# 8-Triazolylpurines: Towards Fluorescent Inhibitors of the MDM2/p53 Interaction

**DOI:** 10.1371/journal.pone.0124423

**Published:** 2015-05-05

**Authors:** Mariell Pettersson, David Bliman, Jimmy Jacobsson, Jesper R. Nilsson, Jaeki Min, Luigi Iconaru, R. Kiplin Guy, Richard W. Kriwacki, Joakim Andréasson, Morten Grøtli

**Affiliations:** 1 Department of Chemistry and Molecular Biology, University of Gothenburg, Gothenburg, Sweden; 2 Department of Chemical and Biological Engineering/Chemistry and Biochemistry, Chalmers University of Technology, Gothenburg, Sweden; 3 Department of Chemical Biology and Therapeutics, St. Jude Children’s Research Hospital, 262 Danny Thomas Place, Memphis, Tennessee, United States of America; 4 Department of Structural Biology, St. Jude Children’s Research Hospital, 262 Danny Thomas Place, Memphis, Tennessee, United States of America; University of East Anglia, UNITED KINGDOM

## Abstract

Small molecule nonpeptidic mimics of α-helices are widely recognised as protein-protein interaction (PPIs) inhibitors. Protein-protein interactions mediate virtually all important regulatory pathways in a cell, and the ability to control and modulate PPIs is therefore of great significance to basic biology, where controlled disruption of protein networks is key to understanding network connectivity and function. We have designed and synthesised two series of 2,6,9-substituted 8-triazolylpurines as α-helix mimetics. The first series was designed based on low energy conformations but did not display any biological activity in a biochemical fluorescence polarisation assay targeting MDM2/p53. Although solution NMR conformation studies demonstrated that such molecules could mimic the topography of an α-helix, docking studies indicated that the same compounds were not optimal as inhibitors for the MDM2/p53 interaction. A new series of 8-triazolylpurines was designed based on a combination of docking studies and analysis of recently published inhibitors. The best compound displayed low micromolar inhibitory activity towards MDM2/p53 in a biochemical fluorescence polarisation assay. In order to evaluate the applicability of these compounds as biologically active and intrinsically fluorescent probes, their absorption/emission properties were measured. The compounds display fluorescent properties with quantum yields up to 50%.

## Introduction

Protein-protein interactions (PPIs) mediate virtually all important biological regulatory pathways [1], and the ability to control and modulate PPIs is therefore of great significance to basic biology, where the controlled disruption of PPI networks is key to understanding network connectivity and function. It is also becoming increasingly clear that the modulation of PPIs offers enormous opportunities in drug discovery for medical diagnostics and treatment.

Designing small molecule inhibitors of PPIs poses a substantial challenge due to PPIs generally shallow interaction sites and large surface area as compared with more traditionally targeted enzyme active sites [2]. However, small regions consisting of a collection of residues that constitute the majority of the free binding energy have been identified and are generally referred to as “hot spots” [3]. These hot spots are often amino acid residues protruding from one face of an α-helix at the interaction surface [4]. A mimetic that reproduces the key interactions of the α-helix should bind to the target binding site of the α-helix.

Tumour protein p53 is crucial in multicellular organisms, where it regulates the cell cycle and functions as a tumour suppressor [5, 6]. All known tumour cells either mutate the p53 gene, or use internal cell p53 modulators like MDM2 and MDMX to disable its function. Releasing functional p53 from inhibition by MDM2 and MDMX should, in principle, provide an efficient, nongenotoxic means of cancer therapy.

The p53 protein binds to MDM2 and MDMX using a short helix with a “hot spot triad” consisting of p53’s Trp23, Leu26, and Phe19 [7]. A number of elegant examples of nonpeptidic α-helix mimetics that inhibit the MDM2/p53 interaction by targeting these hot spots have been published [8]. These inhibitors can be divided into three subcategories: type I, II and III [9]. Type I inhibitors include stabilised oligomers that are designed to mimic the α-helical topography. The second type of inhibitors, functional mimetics, are based on scaffolds that place substituents in the spatial orientation of the parent helix, but the scaffolds themselves are not designed to mimic the α-helix topography. Notable examples of type II inhibitors include the nutlins [10], piperidinones [11] and spiroindolines [12]. These scaffolds vary widely in structure but share the common denominator that they can arrange the substituents in analogy with the *i*, *i*+4 and *i*+7 amino acid side chains of an α-helical structure. In the third category of inhibitors, the nonpeptidic scaffold mimics the topography of an α-helix with substituents protruding in the positions of the *i*, *i*+4 and *i*+7 amino acid side chains of an α-helical structure. Examples from type III include terphenyl [13, 14], and 8-amido-pyrrolopyrimidines[15].

**Fig 1:**
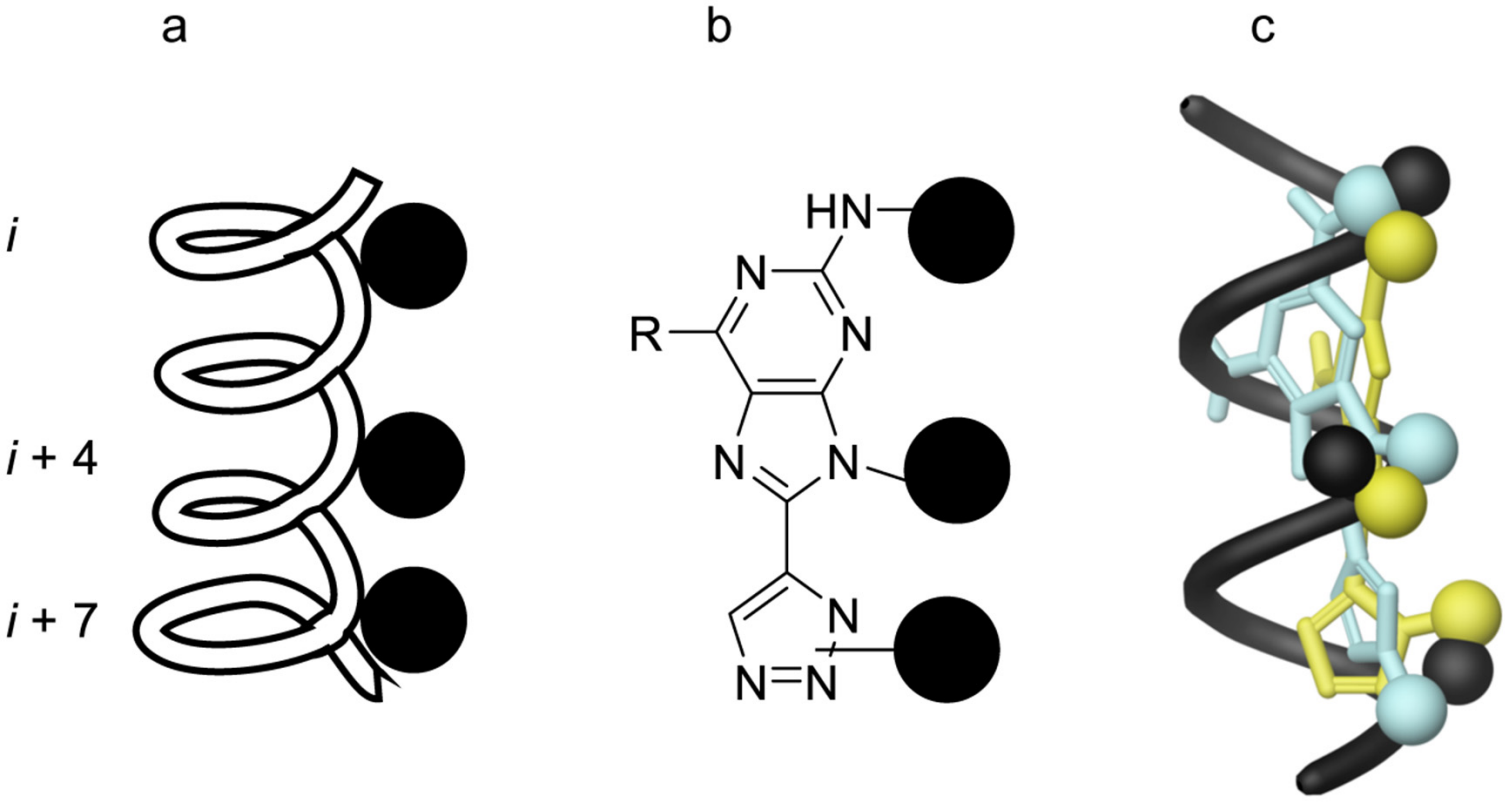
Overview of 8-(triazolyl)purine design. Schematic representation of a) an α-helix with residues present on one face of the helix (*i*, *i*+4 and *i*+7) denoted as black spheres, and b) the 8-triazolyl scaffold with R-groups denoted as black spheres. c) Superimposition of Ala-helix (black), with low energy conformations of 8-(1,4-triazolyl)-purine (turquoise) and 8-(1,5-triazolyl)-purine (yellow).

Purines widely occur in nature [16] and are recognised as privileged structures in drug discovery [17]. We have previously developed 8-(triazolyl) purines as fluorescent base analogues [18, 19] and hypothesised that 8-(triazolyl)purines with substituents in the 2- and 9-positions would project their substituents in analogy with the residues of one face of an α-helix (Fig 1). 7-Deazapurines have previously been reported as inhibitors for MDM2/p53 [15]. Since triazoles are recognised as bioisosters of amide bonds [20–22], we chose to evaluate 8-(triazolyl) purines as α-helix mimetics. Furthermore, given the fluorescent properties of 8-triazolylpurines that were previously reported by our group, we expected 2,9-substituted 8-triazolylpurines to display similar characteristics. Compounds that display both biological activity and intrinsically fluorescent properties would be extremely useful for all applications in which intracellular localisation of the bio-relevant molecule is of interest.

Here, we have evaluated a series of 2,9-substituted 8-(triazolyl)purines as α-helix mimetics. The UV/vis absorption and fluorescence properties of these compounds have also been characterised.

## Results and Discussion

### Type III inhibitors

#### Design

Comparison of low energy conformations of 8-(triazolyl)purine derivatives with an idealised α-helix (Fig 1c) indicated that the scaffold arranges substituents in the *N*2, 9 and on the triazole analogous to the *i*, *i*+4, *i*+7 residues (Fig 1).

The regiochemistry of the triazole can be controlled by the choice of catalyst, which provides a possibility to access compounds with structural diversity. Performing the cyclisation in the presence of a Cu(I) catalyst results in the 1,4-triazole [23]. Changing the catalyst system to Ru(II) results in the 1,5-triazole with high regioselectivity [24]. The 1,5-regioisomer can alternatively be obtained with Me_4_N^+^OH^-^ as catalyst in DMSO [25]. Alkyl- and benzylazides can be obtained from readily available alkylhalides, enabling chemical diversity for the introduction of the R_3_ substituent. The terminal alkyne required for the cyclisation could be accessible through a palladium-catalyzed Sonagashira-type C-C coupling. Activation of the 8-position with a halide would set it up for the C-C coupling. The R_4_ substituent was intended to be introduced by a nucleophilic aromatic substitution (S_N_Ar). The precedence in the literature [26] for this type of reaction in the 6-position of purine derivatives and the commercial availability of 6-chloropurines made this strategy a reasonable choice. Possible strategies for the introduction of substituents in the *N*2 and 9-positions include nucleophilic substitution of alkyl halides, reductive amination (*N*2) or alternatively Mitsunobu-type reactions with primary and secondary alcohols.

#### Synthesis

The *i*, *i+*4 and *i*+7 residues on the α-helix of p53, that are generally recognised as key residues for MDM2 binding, are all hydrophobic (leucine, tryptophan and phenylalanine), which led to the selection of a series of hydrophobic substituents in R_1-3_ for our compounds. The initial route to 2,6,8,9-substituted purines started with alkylation of the 9 position using alkyl or benzyl halide followed by the introduction of a substituent in the *N*2 position via reductive amination. While the alkylation in the 9-position produced reasonable yields using 3-bromo-2-methylpropene (73%), benzyl bromide (59%) and phenethyl bromide (93%) [27], the reductive amination using NaCNBH_3_ or NaBH(OAc)_3_ failed due to the low reactivity of the 2-amino substituent. The major product generated in the reaction was the alcohol product from reduction of the corresponding aldehyde. We then turned to alkylation of the *N*2 position despite expected problems with bis-substitution. As expected, alkylation with aryl halides, potassium iodide and potassium carbonate as base at *N*2 resulted in a mixture of mono- and bis-substitution. Although these species were easily separated by column chromatography, this route failed to generate sufficient quantities of the 2,9-substituted intermediate. It was therefore decided to investigate alternative strategies to achieve these substitutions.

**Fig 2:**
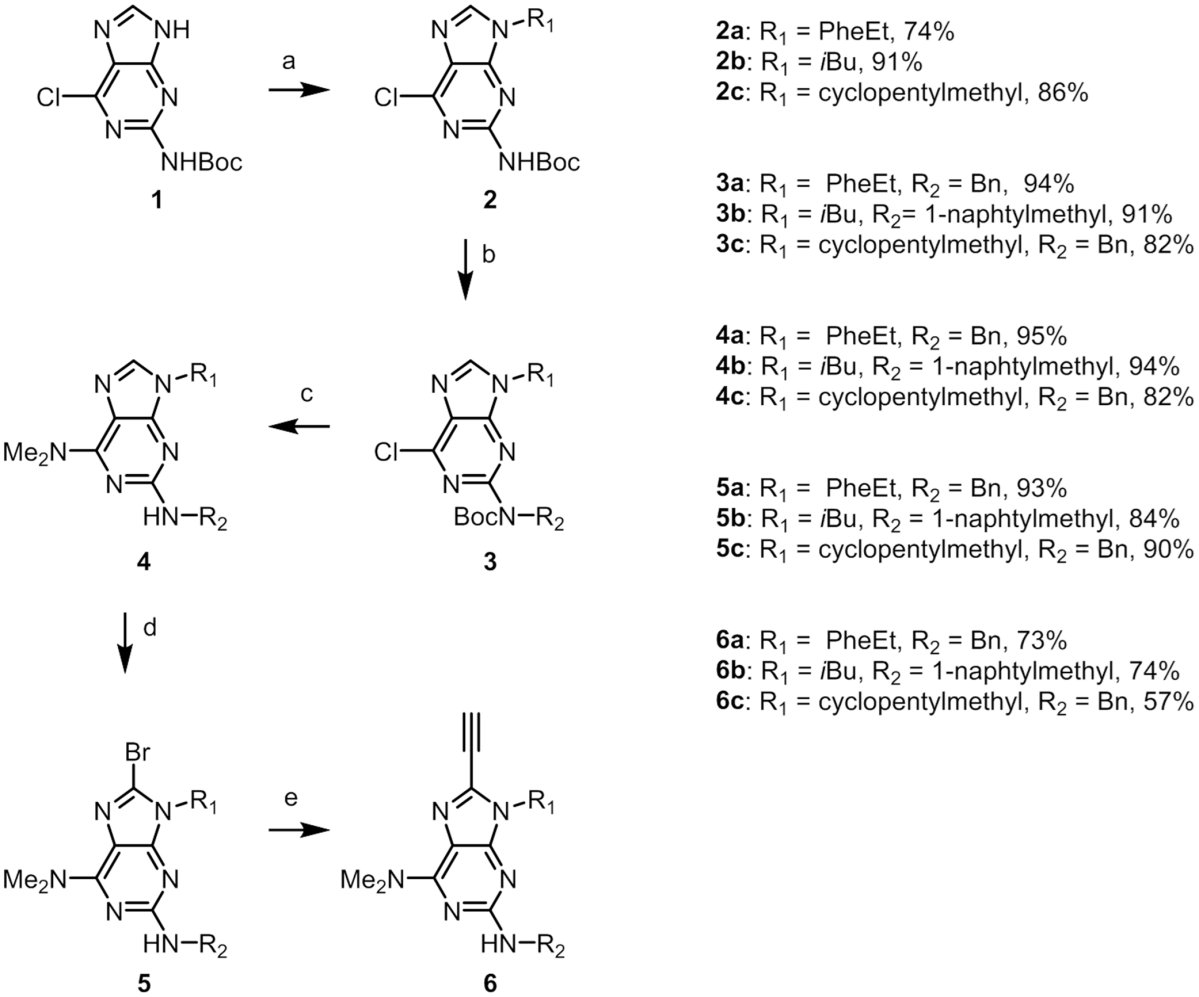
Synthesis of compound 1–6. Reaction conditions: (**a**) R_1_OH, DIAD, Ph_3_P, THF. (**b**) R_2_OH, ADDP, *n*Bu_3_P, THF. (**c**) DMF, MW, 180°C. (**d**) PyBr_3_, CH_2_Cl_2_. (**e**) (i) Pd(II)Cl_2_(PPh_3_)_2_, Cu(I)I, Amberlite IRA-67, TMS-acetylene, MW, 110°C. (ii) PS-F, THF.

Fletcher et al. have published a synthesis of 2,9-substituted guanines starting from 6-chloro-2-amino-purine [28, 29]. The synthesis involves Boc protection in the *N*2-position (**1**) in order to activate it for Mitsunobu coupling (Fig 2). The regioselectivity in the two consecutive Mitsunobu couplings relies on the greater reactivity of the 9-position. This protocol proved successful and allowed us to gain access to **3a-c**. Yields were typically high in both couplings, ranging from 74–91% (**2a-c**) for reactions in the 9-position and 82–94% (**3a-c**) on the exocyclic *N*2-amine.

The chloro substituent in the 6-position of compound **3** is susceptible to nucleophilic aromatic substitution (Fig 2). The dimethylamino substituent was introduced by heating **3a-e** in DMF at 180°C [30]. These reaction conditions also remove the Boc protecting group in the *N*2-position, resulting in **4a-c** in 82–95% yield.

Regarding the next step, activating the 8-position towards C-C-cross coupling by introduction of a bromide, we recently published a procedure for 8-bromination of electron-rich purine derivative using PyBr_3_ in DCM [27]. This procedure afforded **5a-e** in high yields, between 84 and 93%. It is worth mentioning that the bromination is regiospecific and does not react with aromatic substituents.

The terminal alkyne needed for the cyclization was introduced by a Sonogashira-type C-C cross coupling catalysed by PdCl_2_(PPh_3_)_2_ and CuI. Using a solid supported tertiary amine (Amberlite^TM^ IRA67) as base facilitates the workup, and deprotection of the trimethylsilyl group can be carried out on the crude product after filtration to remove the base. Trimethylsilyl removal using polymer supported fluoride (PS-F) in THF afforded **6a-e** in yields of 57–74% over two steps (Fig 2). Here again, the use of a polymer-supported reagent facilitates the workup of the reaction.

**Fig 3:**
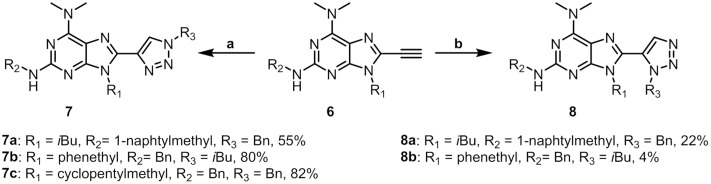
Synthesis of 7a-c and 8a-b. Reaction conditions: (**a**) NaN_3_, R_3_-X, Cu(I)I, NaAsc, DMEDA, DMF. (**b**) Cp*RuCl(PPh_3_)_2_, MW, 120°C, DMF.

1,4-triazoles can be formed with high regioselectivity using a Cu(I) catalyst, a reaction referred to as Copper Catalysed Azide Alkyne Cyclisation (CuAAC). **7a** was obtained by reacting alkyne **6b** with benzyl azide in the presence of CuI, sodium ascorbate and DMEDA at room temperature overnight (Fig 3). To avoid the need to isolate low molecular weight, potentially explosive alkyl azides, isobutyl azide was formed immediately prior to use from isobutyl bromide and sodium azide in DMF. Alkyne, CuI, sodium ascorbate and DMEDA were then added to the azide solution. 1,4-triazolepurines **7a**–**c** were isolated in 55–82% yields (Fig 3). 1,5-Triazoles are accessible by ruthenium-catalysed cyclisation of azides and alkynes [24] and a method for sequential preformation of low molecular weight azides has been reported [31]. Reacting alkyne **6b** with benzyl azide afforded **8a** in 22% yield and when 1,5 cyclization with isobutylazide was attempted, the expected product (**8b**) was isolated in very low yield after extensive purification (Fig 3). The low yield can be attributed, at least in part, to difficulties in separating the product from unidentified by-products.

#### Evaluation against MDM2/p53

Compounds **7a-c** and **8a-b** were evaluated as MDM2 inhibitors in a fluorescence polarisation (FP) assay that measures displacement of a wild-type p53 peptide tagged with a fluorescent probe (Texas Red) bound to MDM2. Unfortunately, the compounds showed no activity at the concentrations tested (up to 47 μM). However, these results prompted us to investigate the solution conformation.

#### Solution conformation analysis

The ability of 8-triazolylpurines to act as α-helix mimetics is dependent on the conformation of the triazole moiety in relation to the purine since this determines the relative positions of the R_1_ and R_3_ substituents. The conformation of **8a** in solution was determined by a combined NMR spectroscopic and computational analysis utilizing the NMR Analysis of Molecular Flexibility (NAMFIS) algorithm [32]. NAMFIS has previously been employed for description of the solution conformations of oligopeptides [33] and small molecules [34], and to evaluate the effect of interstrand hydrogen bonding on β-hairpin stability of cyclic peptides [35]. Experimental proton-proton distances were derived from a NOESY buildup using the initial rate approximation with five mixing times between 80 and 800 ms, ensuring linearity. Theoretically available conformations were predicted using Monte Carlo conformational searches along with Molecular Mechanics Minimisation (MCMM). In order to ensure that the entire conformational space available for the compound was sampled, the conformational search was performed with two different force fields (Amber* and OPLS-2005) and three different solvent models (chloroform, water and octanol). The conformational ensembles were combined and the redundant conformations were removed. Using the NAMFIS algorithm the conformations existing in solution were selected based on the experimentally obtained distances (NOE).

**Fig 4:**
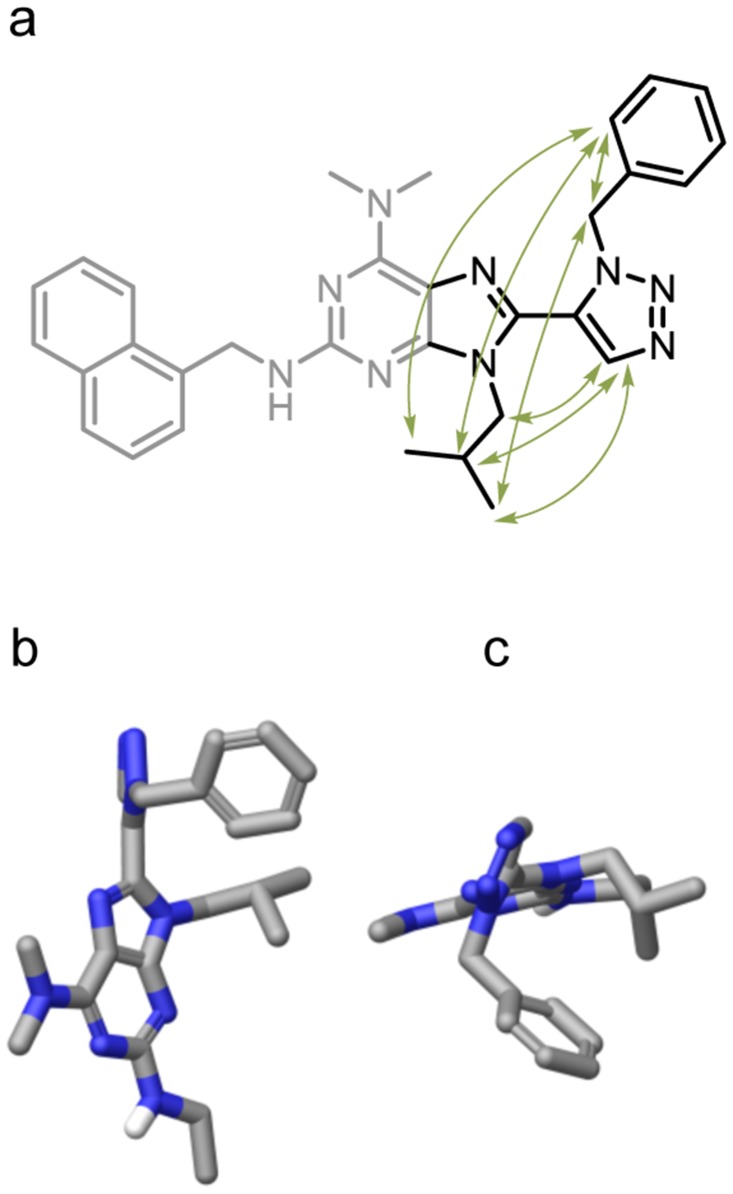
Conformational analysis of 8a. a) NOE correlations (green arrows) in proximity of the triazole ring observed for **8a**. b) Sideview and c) topview of one of the conformations found in the NAMFIS-analysis. (All 6 NAMFIS conformations can be found in Fig. D and E in S1 File).

NAMFIS analysis identified an ensemble of 6 conformations in solution, including conformations that place the substituent in the same spatial orientation as the side chains of an idealised α-helix (Fig 4). The R_2_ substituent was omitted from the analysis since the goal of the conformation analysis was to determine the distance and relative orientation of the R_1_ and R_3_ substituents. The distance and orientation between R_2_ and R_1_ is determined by the rigid and flat purine ring system. An analysis of this part of the molecule would add little or no information about the relative positions of the R_1_ and R_3_ substituents.

**Fig 5:**
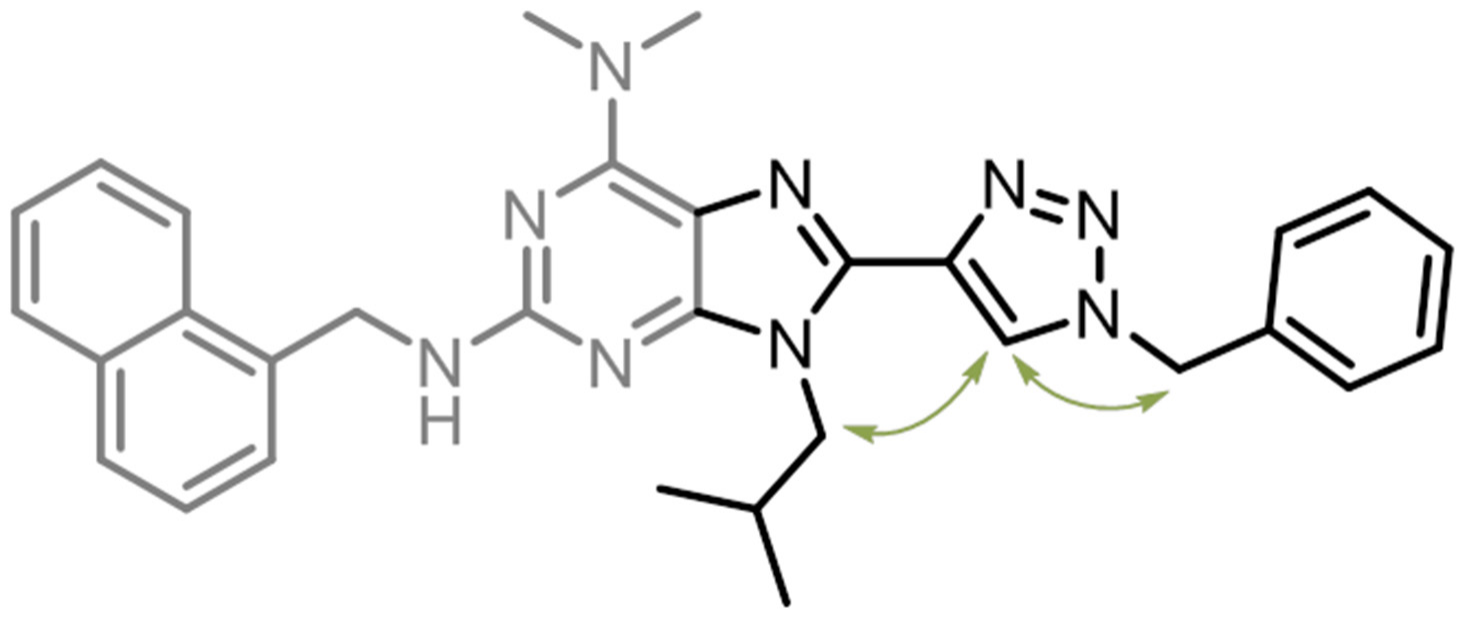
NOE correlations observed for 7a. NOE correlations in proximity of the triazole ring observed for **7a**.

The solution conformations for the 1,4-regioisomer (**7a**) were elucidated using the same method. The experimental distances between protons on the triazole ring and the R_1_ and R_3_ substituents (see Fig 5) were determined by running NOE-buildups with 5 mixing times (80, 200, 400, 600, 800 and 1000 ms). As only two NOE correlations from the triazole H-X are observed for the 1,4-regioisomer (Fig 5), the conformational ensemble could not be reliably determined (overfitting).

The aqueous solubility of compound **7c** was measured and shown to be low (0.3 μM at pH 7.4). Although these compounds are suspected (and supposed) to be lipophilic, the solubility was too low to be practical for biological studies. A more hydrophilic substituent in the 6-position could increase the aqueous solubility of the compounds.

In summary, the solution conformational studies showed that the 8-triazolylpurines scaffold should be able to place the R_1_ and R_3_ substituents in a spatial orientation that mimics the α-helical conformation. The analysis did not, however, reveal why this series was inactive against MDM2/p53 in the FP assay. Since the compounds were inactive as inhibitors against MDM2/p53 interaction at the concentrations evaluated and also showed poor solubility, re-evaluation of the design was undertaken, opting for type II inhibitors.

### Type II inhibitors

#### Design

**Fig 6:**
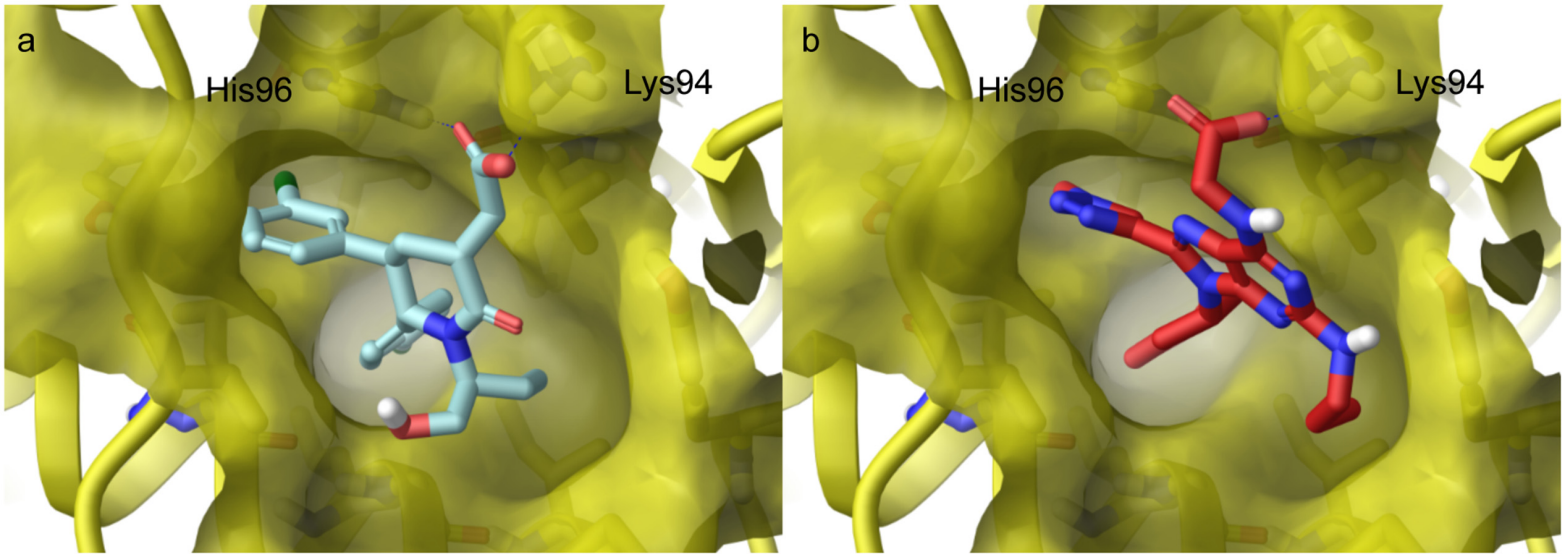
Small molecule inhibitors bound to MDM2. a) Crystal structure of a piperidinone-based inhibitor bound to MDM2 (PDB code: 4HBM)[37] and b) type II inhibitor (R_1_ = indanyl, R_2_ = propyl, R_3_ = methyl and R_4_ = NHCH_2_COOH) docked into the MDM2 binding site.

During our work with the 8-triazolylpurines, new crystal structures of MDM2 co-crystallised with highly potent inhibitors were published [11, 36–38]. Analysis of these and previously published crystal structures [10] of ligand/MDM2 complexes encouraged us to examine two main changes in our design. First, the size of one or two of the hydrophobic substituents should be decreased. Second, several of the published binders of MDM2 have a hydrophilic substituent pointing out towards the solvent/hydrophilic surface of MDM2 enabling hydrogen bonding and/or ionic interactions with the His96 and Lys94 residues of MDM2. The 6-position of our purine derivatives was suitable for the introduction of such functional groups. A series of 8-(triazolyl)purine derivatives were docked into the α-helix binding site of MDM2 (PDB code: 4HBM), using the Schrödinger software package (Glide XP mode), to find a suitable substitution pattern on the 8-(triazolyl)purine ring system. This crystal complex was chosen due to the high affinity and potency of the co-crystallized piperidinones. The models suggest that decreasing the size of the R_3_ substituent to a methyl group, R_2_ to a propyl and R_1_ to an indanyl should improve the fit between the ligand and MDM2. The indanyl is a more rigid isoster of phenethyl. In addition, the introduction of a more hydrophilic group such as an acid or ester could give rise to hydrogen bonding and/or ionic interactions. An example where R_1_ = indanyl, R_2_ = propyl, R_3_ = methyl, and R_4_ = NHCH_2_COOH is shown in Fig 6b. A series of purines with R_1-3_ substituents with varying size and shape were selected for further evaluation of inhibitors. Compound **8a** (type III) was also docked in the grid built from the ligand binding site of MDM2 (PDB code: 4HBM). As expected, the R_1-3_ substituents of a type III inhibitor do not fit into Phe-Trp-Leu-pockets of a crystal structure derived from a type II inhibitor/MDM2 complex.

**Fig 7:**
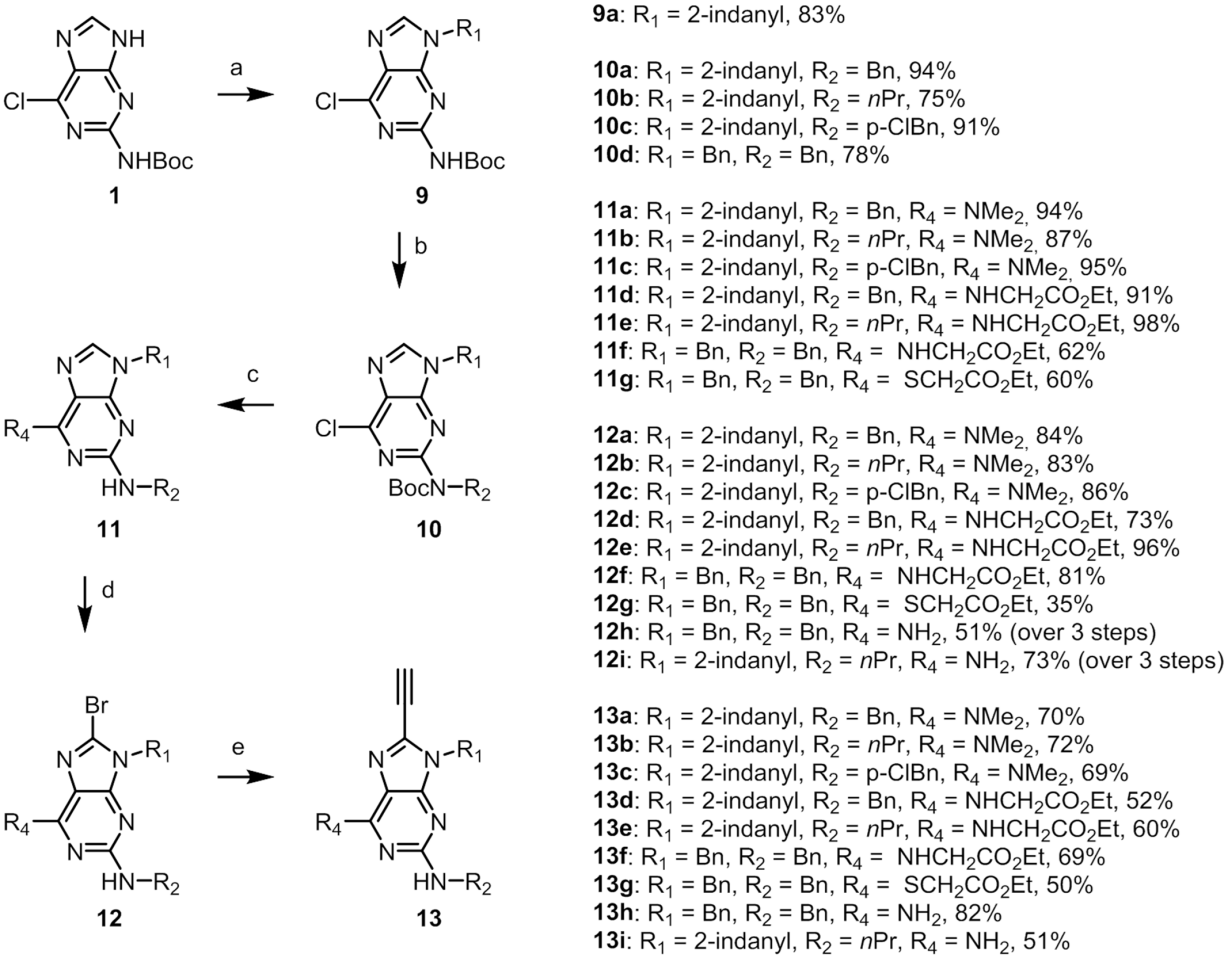
Synthesis of 9–13. Reaction conditions: (**a**) R_1_OH, DIAD, Ph_3_P, THF. (**b**) R_2_OH, ADDP, *n*Bu_3_P, THF. (**c**) R_4_ = NMe_2_, DMF, MW, 180°C. R_2_ = *p*-ClBn, R_4_ = NMe_2_, (i) 5.6 M NMe_2_ in ethanol. (ii) TFA/CH_2_Cl_2_. R_4_ = NHCH_2_CO_2_Et, (i) NH_2_CH_2_CO_2_Et, Et_3_N, ethanol, 100°C. (ii) TFA/CH_2_Cl_2_, R_4_ = SCH_2_CO_2_Et, (i) SHCH_2_CO_2_Et, NaH, toluene, 70°C. (ii) TFA/CH_2_Cl_2_. (**d**) PyBr_3_, CH_2_Cl_2_. For R_4_ = NH_2_, (i) NH_4_OH (aq.), dioxane, 100°C. (ii) TFA/CH_2_Cl_2_ (iii) PyBr_3_, CH_2_Cl_2_. (**e**) (i) Pd(II)Cl_2_(PPh_3_)_2_, Cu(I)I, Amberlite IRA-67, TMS-acetylene, MW, 110°C. (ii) PS-F, THF.

The syntheses of our second series of compounds are shown in Fig 7 Introduction of R_1_ and R_2_ substituents and dimethylamine in the 6-position was performed as described above; **10a-d** and **11a-b** were isolated in 75–94% and 87–94% yield, respectively. When the *N*2-position was substituted with a *p-*chlorobenzyl, milder conditions for the introduction of dimethylamine were used to avoid aromatic substitution in the *p-*chloro position. Running the reaction with dimethylamine in ethanol (5.6 M) at 80°C gave regioselective substitution of the 6-chloro and subsequent Boc removal with TFA in DCM gave **11c** in 95% yield over two steps. For the introduction of an ester in the 6-postion, the 6-chloropurine derivatives were reacted with thioglycol ethylester and glycine ethylester under basic conditions followed by Boc removal (TFA/DCM) to give **11d-g** in 60–98% yields over two steps. Next, treatment of **11a-g** with PyrBr_3_ provided **12a-g** in 35–96% yield, where the lower yields of 35% was obtained for bromination of compound **11g**. Sulphur in the 6-position gives lower yields compared with nitrogen. An alternative to decrease the lipophilicity of these compounds would be to introduce a primary amine in the 6-position. Treatment of the 6-chloroderivatives with ammonium hydroxide gave the 6-aminoderivative. Bromination using PyBr_3_ in DCM did not give the desired product which was assumed to be due to the electron withdrawing effect of the Boc group in the *N*2-position. Removing the Boc group with TFA in DCM prior to the bromination was necessary to facilitate the success of the reaction. The amination, deprotection and bromination sequence can be run conveniently without any intermediate purification to afford **12h** and **12i** in 51 and 73% yield over three steps, respectively.

**Fig 8:**
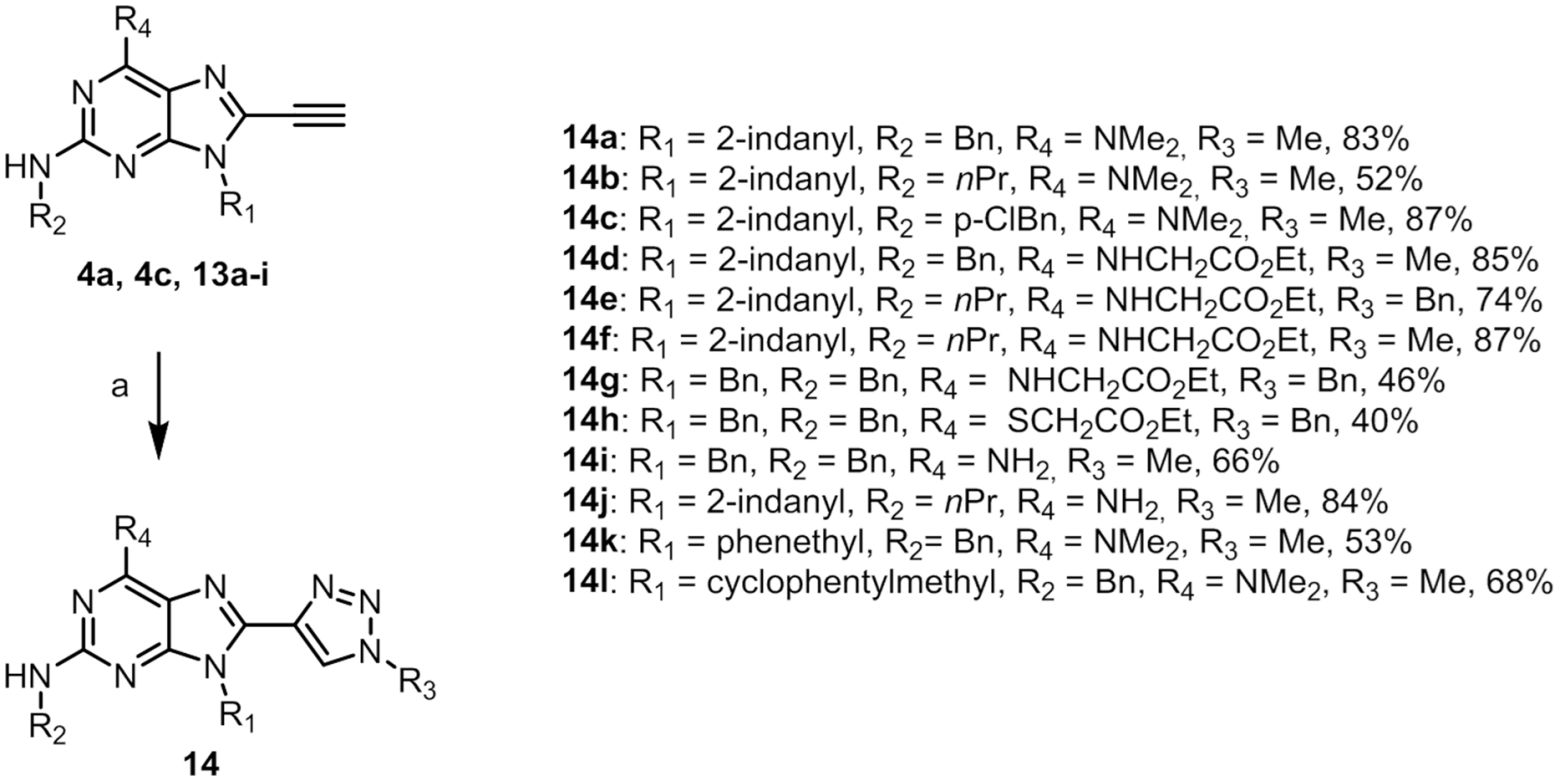
Synthesis of 14a-l. Reaction conditions: (**a**) NaN_3_, R_3_-X, Cu(I)I, NaAsc, DMEDA, DMF.

The Sonsogashira coupling followed the same protocol as above to give **13a-i** (Fig 7). Further, the same protocol for the 1,4-triazole formation was again employed to provide **14a-l** (Fig 8), and as described above for the synthesis of isobutylazide, methylazide was formed *in situ*. The highest yields in the cyclisation were obtained when the azide formation was run in room temperature followed by cyclisation at 60°C. Low yields were isolated if the reaction mixture vial was flushed with nitrogen after azide formation which is assumed to be due to the volatility of methylazide.

**Fig 9:**
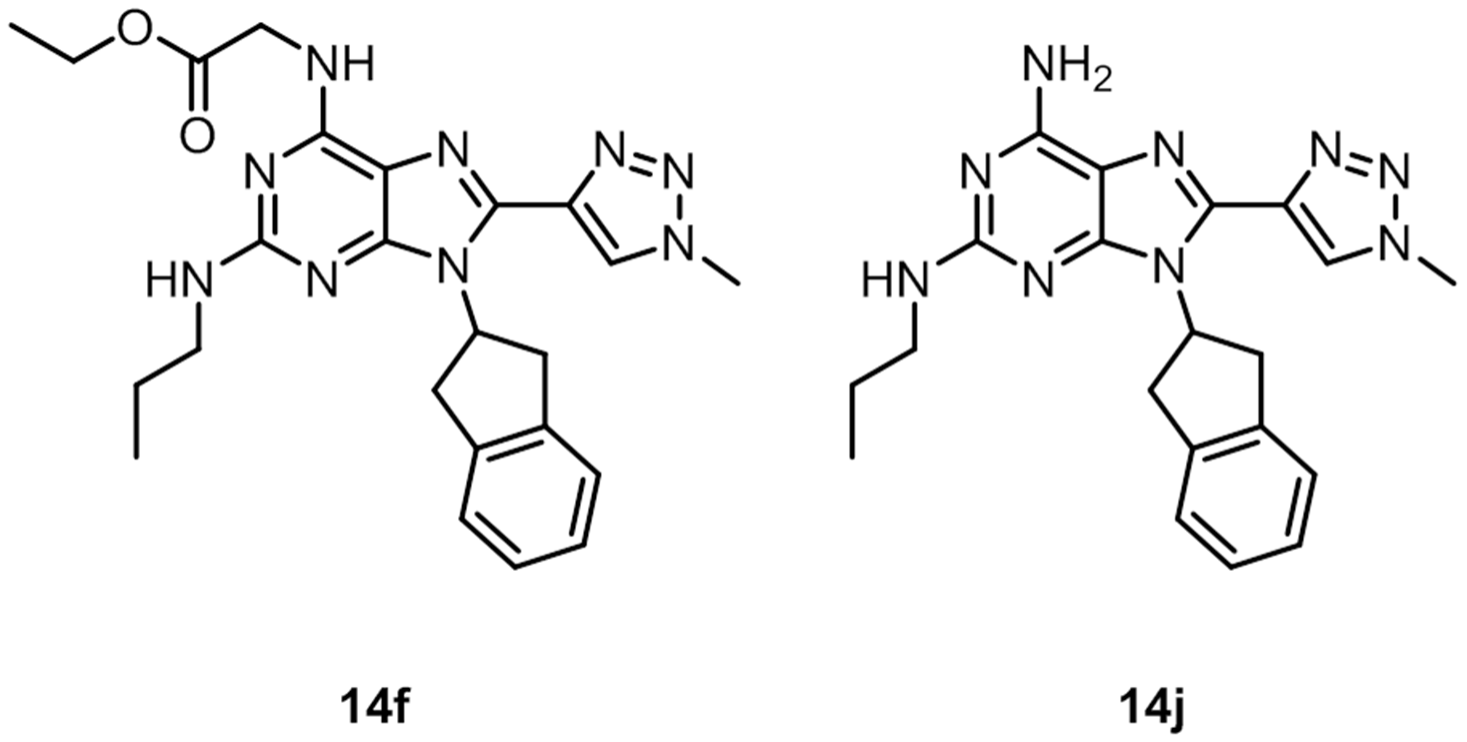
8-(triazolyl)purine inhibitors of MDM2/p53.

These new compounds were also evaluated for their inhibitory effect on MDM2 in a fluorescence polarisation assay. Of the 12 new compounds, 10 were inactive towards p53/MDM2 at concentrations up to 100 μM. Compounds **14f** and **14j** (Fig 9) displayed IC_50_ values of 10 (Fig. P in S1 File) and 56 μM, respectively. Compound **14f** was further evaluated using WaterLOGSY[39] which confirmed binding to MDM2 (Fig. Q in S1 File). These results verify that with this scaffold, compounds with larger substituents in the R_1_ and R_2_-position do not bind to MDM2. This validates the redesign and fits with the observation that many of the published low nM inhibitors of the p53/MDM2 interaction are typically less extended than the model p53 α-helix. One explanation for the low potency observed for the type II 8-(triazolyl)purine derivatives could be that while the methyl on the triazole fits into one of the hydrophobic interaction sites as observed in the docking studies, this fit might bury the polar triazole as well, resulting in a desolvation penalty. Furthermore, the active compounds **14f** and **14j** lack the ability of the ionic interaction formed between the carboxylic acid of the model inhibitor (piperidinones) and the Lys94 residue of MDM2. Recently published docking studies of known p53/MDM2 inhibitors highlight the challenges posed by using docking as a tool for developing inhibitors against this target.[40]

#### Photophysical characterisation

Intrinsically fluorescent α-helix mimetics could be used for imaging and localization of the compounds in cells. We have previously reported the fluorescent properties of 6,9-substituted 8-triazolylpurines [18] and anticipated that the structurally similar 2,6,9-substituted derivatives would have similar behaviour. UV/vis absorption and fluorescence measurements were undertaken to investigate this possibility. The data are presented in Table 1.

**Table 1:** Absorption and fluorescent properties of 8-(triazolyl)purine derivatives in methanol solution.

Entry	Compound	R_1_	R_2_	R_3_	R_4_	λ_abs,max_ (nm)	λ_em,max_ (nm)	*Φ*_*F*_ (%)
1	**14a**	2-indanyl	Bn	1,4-Me	NMe_2_	310	376	2
2	**14k**	phenethyl	Bn	1,4-Me	NMe_2_	315	376	2
3	**7c**	cyclopentylmethyl	Bn	1,4-Bn	NMe_2_	317	377	2
4	**14b**	2-indanyl	*n*Pr	1,4-Me	NMe_2_	311	378	1
5	**14l**	cyclopentylmethyl	Bn	1,4-Me	NMe_2_	316	376	2
6	**14i**	Bn	Bn	1,4-Me	NH_2_	314	375	7
7	**14j**	2-indanyl	*n*Pr	1,4-Me	NH_2_	311	377	4
8	**14d**	2-indanyl	Bn	1,4-Me	NHCH_2_CO_2_Et	311	377	8
9	**14g**	Bn	Bn	1,4-Bn	NHCH_2_CO_2_Et	318	381	10
10	**14e**	2-indanyl	*n*Pr	1,4-Bn	NHCH_2_CO_2_Et	316	382	4
11	**14f**	2-indanyl	*n*Pr	1,4-Me	NHCH_2_CO_2_Et	313	380	5
12	**7a**	1-naphtyl	*i*Bu	1,4-Bn	NMe_2_	318	378	2
13	**7b**	phenethyl	Bn	1,4-*i*Bu	NMe_2_	316	378	5
14	**8a**	1-naphtyl	*i*Bu	1,5-Bn	NMe_2_	322	437	37
15	**8b**	phenethyl	Bn	1,5-*i*Bu	NMe_2_	320	422	51
16	**14h**	Bn	Bn	1,4-Bn	SCH_2_CO_2_Et	340	398	2

λ_abs, max_ and λ_em, max_ are the wavelengths of absorption and emission maxima, respectively, and *Φ*_*F*_ is the fluorescence quantum yield

**Fig 10:**
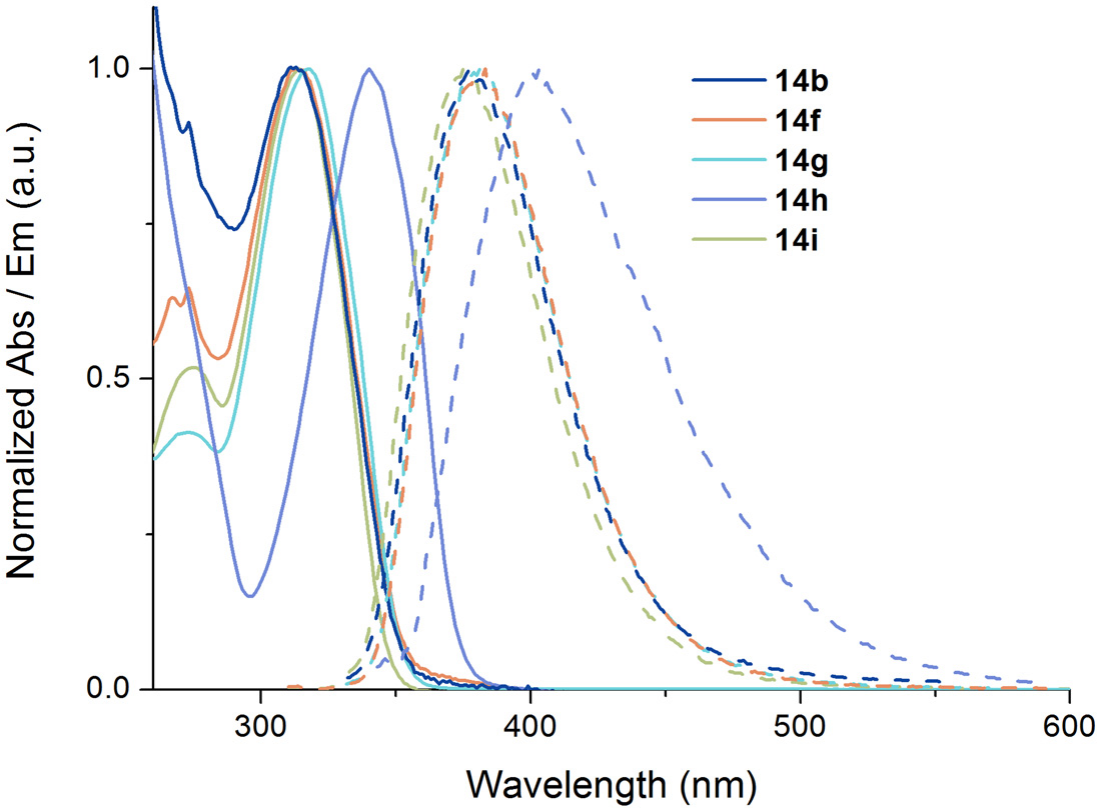
Photophysical characterization of 14b, 14f, 14g, 14h and 14i. Normalised absorption (solid lines) and emission (dashed lines) spectra of a selection of the investigated compounds (the full set of absorption and emission spectra can be found in the SI).

The 8-triazolyl compounds (Table 1, entries 1–15) have absorption maxima in the range 310–322 nm, with the exception of **14h** (entry 16), which bears a sulphur substituent in the 6-position. The absorption maximum of this compound is redshifted to 340 nm. Compounds with a 6-dimethylamino substituent, such as R_4_ and a 1,4-triazole, all have low fluorescence quantum yields between 1 and 2% (entries 1–5,12–13). Changing from tertiary to primary or secondary amines as R_4_ substituent gives higher quantum yield (5–10%). A selection of absorption and emission spectra is shown in Fig 10.

The substitution pattern on the triazole and in the 9 and *N*2 position seems to have little effect on the quantum yield. This is expected since none of the substituents in these positions changes the conjugation of the heterocyclic core. Interestingly, changing the regioisomerism of the triazole from 1,4 to 1,5 (entries 12–15) results in a 10-20-fold increase of the quantum yield. In addition, the Stokes shift increases from 62 to 102 nm (entries 13 and 15). To the best of our knowledge, this is the first time this regioisomeric effect has been observed for fluorescent triazoles and it might have implications in the field of fluorescent base analogs. The increase in quantum yield observed when shifting from the 1,4 to the 1,5-regioisomer could possibly originate from increased steric hindrance and a higher rotational barrier of the purine-triazole bond leading to less rotational flexibility in the excited state, decreasing the rate of non-radiative deactivation. To establish the influence of the triazole on the fluorescent properties, **5a** and **15–16** bearing a proton, bromine, and an alkyne in the 8-position, respectively, were also measured (data not shown). These compounds all displayed very weak fluorescent properties and demonstrate that the 8-triazole substituent is necessary to observe the higher quantum yields.

## Materials and Methods

A representative synthesis example of 6-(ethoxy-carbonyl-methylamino)-9-(2-indanyl)-8-(1-methyl-1H-1,2,3-triazol-4-yl)-2-(1-propylamino)purine (**14f**) is described below.

### 2-Amino-9-*tert*-butoxycarbonyl-6-chloropurine

Following a published procedure[29] with minor modifications, 2-amino-6-chloropurine (2.50 g, 14.7 mmol) and di-*tert*-butyl dicarbonate (3.21 g, 14.7 mmol) were dissolved in anhydrous DMSO (50 ml). The pale yellow solution was cooled in an ice bath with vigorous stirring and the ice bath was removed just as the DMSO started to freeze. DMAP (90 mg, 0.74 mmol) was added and the solution was stirred under nitrogen at room temperature for 8 h. Additional di-*tert*-butyl dicarbonate (220 mg, 1.00 mmol) was added and the reaction mixture was stirred at room temperature overnight. Full consumption of starting material and formation of one new spot was confirmed by TLC (10% methanol in CHCl_3_).*The reaction mixture was* diluted with water (450 ml) and extracted with ethyl acetate (3 x 150 ml). The organic phases were washed with water (5 x 100 ml), dried over Na_2_SO_4_, filtered and evaporated to give the expected product as a white solid (3640 mg, 92%) which was used without further purification in the next step. ^1^H NMR (CDCl_3_): 8.13 (s, 1H), 5.92 (br s, 2H), 1.66 (s, 9H); ^13^C NMR (CDCl_3_): 160.6, 153.4, 152.4, 145.7, 140.2, 125.5, 87.2, 28.0.

### 2-*tert*-Butoxycarbonylamino-6-chloropurine (1)

Sodium hydride (715 mg, 17.9 mmol, 60% mineral oil dispersion) was added to a stirred solution of 2-amino-9-tert-butoxycarbonyl-6-chloropurine, (3490 mg, 12.9 mmol) in dry THF (120 ml) at room temperature under nitrogen. The white suspension was stirred at room temperature for 2 h. Full conversion of starting material was confirmed by TLC (70% ethyl acetate in pentane). The reaction was cooled to 0°C and quenched with brine (5 ml); a white precipitate was observed after the addition. The mixture was then allowed to reach room temperature. The THF was removed under reduced pressure (note: save 10–20 ml THF, to simplify the extraction). CHCl_3_ (200 ml) and distilled water (800 ml) were added (everything did not dissolve). The pH on the aqueous phase was determined to be 14. The phases were separated and extracted with CHCl_3_ (3 x 100 ml, note: wait approximately 10 min between every extraction) and these organic phases were discarded. Sat. NaHCO_3_ (aq.) was added to the aqueous phase, whereupon a white precipitate was observed; the aqueous phase was extracted with CHCl_3_ (3 x 100 ml). The combined organic phases were washed with brine. The solvent was removed under reduced pressure to yield **1** as a white solid in quantitative yield. ^1^H and ^13^C NMR corresponds with previously published results[27]. ^1^H NMR (CDCl_3_): 13.53 (s, 1H), 8.44 (s, 1H), 7.78 (s, 1H), 1.58 (s, 9H); ^13^C NMR (CDCl_3_): 153.3, 151.6, 151.3, 151.0, 145.5, 128.3, 82.4, 28.3.

### General procedure A: Mitsunobu reaction in the 9-position

The alkylation was performed following a published procedure[29] with minor modifications. **1** (1.0 eq.) was dissolved in dry THF under nitrogen in oven-dried round-bottomed flask and alcohol (1.1–2.5 eq.) and PPh_3_ (1.1–2.5 eq.) was added. The nitrogen flow was temporarily removed when solid alcohols were added. When all of the PPh_3_ was dissolved, DIAD (1.0–2.5 eq.), were added drop wise. The reaction was stirred at room temperature under nitrogen, until TLC indicated full consumption of the starting material, unless otherwise noted. The solvent was removed under reduced pressure and the crude product was purified by flash column chromatography or automated flash column chromatography.

### 6-Chloro-9-(2-indanyl)-2-(*tert*-butoxycarbonylamino)purine (9a)

Compound **9a** was synthesised following general procedure A at 0°C for 2 h from **1** (816 mg, 3.03 mmol), 2-indanol (1031 mg, 7.60 mmol), PPh_3_ (1953 mg, 7.44 mmol) and DIAD (1.45 ml, 7.36 mmol) as substrates in dry THF (42 ml). The crude product was purified by flash column chromatography (30–70% ethyl acetate in pentane) to provide **9a** as a white solid (970 mg, 83%). ^1^H NMR (CDCl_3_): 7.73 (s, 1H), 7.53 (br s, 1H), 7.34–7.25 (m, 4H), 5.55 (tt, *J* 7.4, 4.3 Hz, 1H), 3.63 (dd, *J* 16.5, 7.4 Hz, 2H), 3.26 (dd, *J* 16.4, 4.3 Hz, 2H), 1.54 (s, 9H); ^13^C NMR (CDCl_3_): 152.6, 152.3, 151.1, 150.2, 141.9, 139.6, 127.6, 127.5, 124.8, 81.7, 55.0, 40.1, 28.2. HRMS *m/z* [M + H]^+^ calculated for C_19_H_20_ClN_5_O_2_: 386.1384. Found: 386.1351.

### General procedure B: Mitsunobu reaction in the 2-position

The alkylation was performed following a published procedure[29] with minor modifications. The purine was dissolved in dry THF under nitrogen in an oven-dried round-bottomed flask, PBu_3_ (2.4–2.6 eq.), alcohol (2.4–2.6 eq.) and ADDP (2.5–2.6 eq.) was added in that order. The nitrogen flow was temporarily suspended when solid alcohols and ADDP were added. The reaction was stirred at room temperature, until TLC indicated full consumption of the starting material, unless otherwise noted. The obtained white precipitate was filtered off, and washed with THF, and the solvent was removed under reduced pressure. The crude product was purified by flash column chromatography or automated flash column chromatography.

### 6-Chloro-9-(2-indanyl)-2-(N-propyl-tert-butoxycarbonylamino)purine (10b)

Compound **10b** was synthesised following general procedure B from **9a** (2930 mg, 7.59 mmol), *n-*propanol (1.45 ml, 19.40 mmol), PBu_3_ (4.8 ml, 18.26 mmol) and ADDP (4790 mg, 18.99 mmol) in dry THF (144 ml). The reaction was stirred at room temperature for 24 h. The reaction was monitored by TLC (50% ethyl acetate in pentane). The crude product was purified by flash column chromatography (25–100% ethyl acetate in pentane) to provide compound **10b** as a light yellow sticky solid (2450 mg, 75%). Starting material (727 mg) was recovered after column chromatography. ^1^H NMR (CDCl_3_): 7.84 (s, 1H), 7.33–7.24 (m, 4H), 5.45 (tt, *J* 7.6, 5.0 Hz, 1H), 3.89–3.85 (m, 2H), 3.60 (dd, *J* 16.3, 7.5 Hz, 3H), 3.36 (dd, *J* 16.3, 5.0 Hz, 2H), 1.67 (sext, *J* 7.5 Hz, 2H), 1.50 (s, 9H), 0.88 (t, *J* 7.5, 3H); ^13^C NMR (CDCl_3_): 155.3, 154.0, 152.4, 150.5, 142.8, 139.6, 128.4, 127.7, 124.9, 81.4, 55.5, 50.2, 39.8, 28.3, 22.2, 11.4. HRMS *m/z* [M + H]^+^ calculated for C_22_H_26_ClN_5_O_2_: 428.1853. Found: 428.1865.

### 6-(Ethoxy-carbonyl-methylamino)-9-(2-indanyl)-2-(1-propylamino)purine (11e)

The reaction was run in two batches, which were then pooled prior to work-up and purification. **10b** (250 mg, 1.17 mmol) was suspended in ethanol (15 ml, 99.7%) in two oven-dried microwave vials. Glycine ethyl ester hydrochloride (245 mg, 1.76 mmol) and triethyl amine (0.41 ml, 2.94 mmol) were added and the reactions were heated to 100°C whereupon the starting materials dissolved. The reaction was left at 100°C overnight. TLC (5% methanol in CH_2_Cl_2_) indicated full consumption of the starting material. The two batches were pooled and the solvent was removed under reduced pressure. The crude product was dissolved in CH_2_Cl_2_ (13 ml), and TFA (4 ml) was added. The reaction mixture was stirred at room temperature for 3 h, after which full deprotection was confirmed by LCMS. The reaction mixture was cooled on ice and diluted with distilled water (20 ml) and then basified with 6 M NaOH (aq.). The aqueous phase was extracted with CH_2_Cl_2_ (4 x 20 ml). The combined organic phases were washed with brine and dried over MgSO_4_, filtered, and the solvent was removed under reduced pressure. The crude product was purified by automated flash column chromatography (5% methanol in CH_2_Cl_2_) to provide compound **11e** as a light yellow oil/foam (451 mg, 98%). ^1^H NMR (CDCl_3_): 7.31 (s, 1H), 7.30–7.18 (m, 4H), 6.25 (br s, 1H), 5.24–5.31 (m, 1H), 4.86 (t, *J* 5.9 Hz, 1H), 4.31 (d, *J* 5.4 Hz, 2H), 4.20 (q, *J* 7.1 Hz, 2H), 3.50 (dd, *J* 16.2, 7.6 Hz, 2H), 3.38–3.25 (m, 4H), 1.59 (sext, *J* 7.3 Hz, 2H), 1.26 (t, *J* 7.1 Hz, 3H), 0.95 (t, *J* 7.4 Hz, 3H); ^13^C NMR (CDCl_3_): 170.7, 159.6, 154.5, 151.8, 140.5, 135.1, 127.2, 124.7, 114.4, 61.2, 54.2, 43.8, 42.7, 39.8, 23.2, 14.3, 11.7. HRMS *m/z* [M + H]^+^ calculated for C_21_H_25_N_6_O_2_: 395.2195. Found: 395.2195.

### General procedure D: 8-bromination of 2,6,9-substituted purines

Purine (1.0 eq.) was dissolved in dry CH_2_Cl_2_ in oven-dried glassware under nitrogen. The nitrogen flow was temporarily suspended when pyridinium tribromide (PyrBr_3_) (1.1–1.4 eq.) was added in one portion. The reaction mixture was stirred at room temperature under nitrogen until full conversion was observed by TLC or LCMS. The reaction was quenched with 10% Na_2_S_2_O_3_ (aq.); the colour changed from yellow to colorless. The pH was adjusted to 9–12 with 15% NaOH (aq.). The phases were separated and the aqueous phase was extracted with CH_2_Cl_2_ (x 3). The organic phases were pooled, washed with brine, dried over Na_2_SO_4_ and filtered. The solvent was removed under reduced pressure, and the crude product was purified by flash column chromatography.

### 8-Bromo-6-(ethoxy-carbonyl-methylamino)-9-(2-indanyl)-2-(1-propylamino)purine (12e)

Compound **12e** was synthesised following general procedure D from **11e** (418 mg, 1.06 mmol) and PyrBr_3_ (410 mg, 1.21 mmol) in dry CH_2_Cl_2_ (17 ml). The reaction was stirred at room temperature for 5 h, whereupon full consumption of starting material was observed by LCMS. The crude product was purified by automated flash column chromatography (20–30% ethyl acetate in pentane) to provide **12e** as a colorless oil (479 mg, 96%). ^1^H NMR (CDCl_3_): 7.26–7.19 (m, 4H), 5.92 (t, *J* 5.3 Hz, 1H), 5.34 (quin, *J* 9.1 Hz, 1H), 4.77 (t, *J* 5.9 Hz, 1H), 4.30 (d, *J* 5.5 Hz, 2H), 4.23 (q, *J* 7.1 Hz, 2H), 3.96 (dd, *J* 15.5, 9.2 Hz, 2H), 3.26 (dd, *J* 15.4, 9.0 Hz, 2H), 3.14 (q, *J* 6.6 Hz, 2H), 1.48 (sext, *J* 7.4 Hz, 2H), 1.29 (t, *J* 7.1 Hz, 3H), 0.84 (t, *J* 7.4 Hz, 3H); ^13^C NMR (CDCl_3_): 170.5, 158.8, 153.4, 153.1, 140.8, 126.9, 124.5, 122.2, 115.3, 61.4, 56.6, 43.6, 42.6, 37.0, 23.0, 14.4, 11.6. HRMS *m/z* [M + H]^+^ calculated for C_21_H_25_BrN_6_O_2_: 473.1300. Found: 473.1331.

### 6-(Ethoxy-carbonyl-methylamino)-8-ethynyl-9-(2-indanyl)-2-(1-propylamino)purine (13e)

PdCl_2_(PPh_3_)_2_ (28 mg, 0.040 mmol), CuI (31 mg, 0.16 mmol), and amberlite IRA-67 (718 mg, 4.02 mmol) were added to a solution of **12e** (380 mg, 0.803 mmol) in dry THF (12 ml). The vial was capped, nitrogen was bubbled through the reaction mixture and ethynyltrimethylsilane (450 μl, 3.25 mmol) was added. The yellow reaction mixture was heated in a microwave reactor at 110°C for 50 min. Full consumption of **12e** was confirmed by TLC (2% methanol in CHCl_3_). The reaction mixture was filtered through a short plug of silica that was eluted with THF. The solution was concentrated and the volume was adjusted to 8 ml. Polymer-supported fluoride (422 mg) was added and the reaction mixture was stirred under nitrogen at room temperature for 19 h. The polymer was filtered off, washed with THF and CH_2_Cl_2_ and the solvents were removed under reduced pressure. Purification by flash column chromatography (first column eluted with 2% methanol in CH_2_Cl_2_ and second column with 33% ethyl acetate in pentane) provided **13e** as a yellow solid (200 mg, 60%). ^1^H NMR (CDCl_3_): 7.26–7.17 (m, 4H), 6.11 (s, 1H), 5.47 (quin, *J* 9.0 Hz, 1H), 4.84 (t, *J* 5.8 Hz, 1H), 4.31 (br s, 2H), 4.23 (q, *J* 7.1 Hz, 2H), 3.91 (dd, *J* 15.5, 9.3 Hz, 2H), 3.35 (s, 1H), 3.30 (dd, *J* 15.5, 8.8 Hz, 3H), 3.20 (q, *J* 6.7 Hz, 2H), 1.51 (sext, *J* 7.3 Hz, 2H), 1.28 (t, *J* 7.1 Hz, 3H), 0.87 (t, *J* 7.4 Hz, 3H); ^13^C NMR (CDCl_3_): 170.4, 159.9, 154.4, 151.7, 141.0, 129.7, 126.9, 124.5, 114.8, 83.0, 74.3, 61.4, 55.4, 43.6, 42.6, 37.6, 23.1, 14.3, 11.6. HRMS *m/z* [M + H]^+^ calculated for C_23_H_26_N_6_O_2_: 419.2195. Found: 419.2206.

### 6-(Ethoxy-carbonyl-methylamino)-9-(2-indanyl)-8-(1-methyl-1H-1,2,3-triazol-4-yl)-2-(1-propylamino)purine (14f)

Methyl iodide (24 μl, 0.39 mmol) and sodium azide (25 mg, 0.38 mmol) in dry DMF (2 ml) were stirred at room temperature for 3 h in a microwave vial. The formed methyl azide solution was then transferred to a microwave vial containing **13e** (82 mg, 0.20 mmol), sodium ascorbate (12 mg, 0.06 mmol), CuI (7 mg, 0.04 mmol) and DMEDA (5 μl, 0.05 mmol) in dry DMF (1.5 ml). The reaction mixture was heated at 60°C for 3 h and then at room temperature for 15 h. Full consumption of **13e** was confirmed by TLC (10% methanol in CHCl_3_). The reaction mixture was poured into ethyl acetate (25 ml), extracted with water (3 x 20 ml), dried over Na_2_SO_4_ and the solvents were removed under reduced pressure. *Caution*: *Keep aqueous phase slightly basic (0*.*5 M NaOH (aq*.*) was added to the aqueous phase) to avoid hydrazoic acid formation*. Purification by flash column chromatography (ethyl acetate) provided **14f** as a yellow solid (81 mg, 87%). ^1^H NMR (CDCl_3_): 8.11 (br s, 1H), 7.25–7.13 (m, 4H), 6.31 (br s, 1H), 6.16 (br s, 1H), 4.75 (br s, 4.75, 1H), 4.33 (s, 2H), 4.24 (q, *J* 7.1 Hz, 2H),4.14 (s, 3H), 4.05 (dd, *J* 14.9, 8.5 Hz, 2H), 3.32 (dd, *J* 14.8, 8.9 Hz, 2H), 3.12 (q, *J* 6.6 Hz, 2H), 1.46 (q, *J* 7.2 Hz, 1H), 1.30 (t, *J* 7.0 Hz, 3H), 0.81 (t, *J* 7.4 Hz, 3H); ^13^C NMR (CDCl_3_): 170.9, 158.9, 154.4, 153.4, 141.6, 140.9, 139.0, 126.5, 125.4, 124.4, 114.7, 61.2, 56.1, 43.6, 42.5, 37.2, 36.8, 23.0, 14.3, 11.5. *Note*: *Weighting functions were applied to the* ^*1*^ *H NMR spectra to obtain J couplings (sine bell*: *2°*, *sine square*: *88°)*. HRMS *m/z* [M + H]^+^ calculated for C_24_H_29_N_9_O_2_: 476.2522. Found: 476.2485.

## Conclusion

An efficient strategy for the preparation of 2,6,9-substituted 8-triazolylpurines has been developed. The target compounds are obtained in excellent yields by a convenient multistep procedure. Conformation studies by solution phase NMR spectroscopy demonstrated that 2,6,9-substituted 8-triazolylpurines could mimic the topography of an α-helix and could be potentially useful as MDM2/p53 inhibitors. However, our study clearly indicates that this is not sufficient for *in vitro* activity as the first series of compounds were inactive at the tested concentrations in a biochemical fluorescence polarisation assay targeting MDM2/p53. Further iterative design-synthesis-testing cycles using docking as the main design tool resulted in a one micromolar inhibitor (**14f**, 10 μM). Binding of **14f** to MDM2 was confirmed by WaterLOGSY measurements. Furthermore, the 2,6,9-substituted 8-triazolylpurines displayed moderate to high fluorescence quantum yields in the range of 5–51%. The combination of fluorescent properties with inhibitory effect on the MDM2-p53 interaction could potentially be useful for real-time imaging as well as for the development of *in vitro* displacement assays for MDM2/p53. Developments in this direction are currently ongoing in our laboratories.

## Supporting Information

S1 File Describes synthesis and characterisation including Proton (^1^H) and Carbon (^13^C) NMR spectra of all compounds synthesised. In addition, experimental procedures for the biological evaluation and photophysical characterisation are included. A more specific table of contents can be located in this document.
(PDF)
