# Supplementary material for: 8-Triazolylpurines: Towards Fluorescent Inhibitors of the MDM2/p53 Interaction
Source: PLoS One. 2015 May 5;10(5):e0124423. doi: 10.1371/journal.pone.0124423 (PMC4420247; doi:10.1371/journal.pone.0124423)
Supplement: S1 File — In addition, experimental procedures for the biological evaluation and photophysical characterisation are included. A more specific table of contents can be located in this document. (PDF) [file pone.0124423.s001.pdf]

Supporting information

8-Triazolylpurines: Towards Fluorescent Inhibitors of the MDM2/p53 Interaction

Mariell Pettersson[¶1], David Bliman[¶1], Jimmy Jacobsson[1], Jesper R. Nilsson[2], Jaeki Min[3], Luigi Iconaru[4], R. Kiplin Guy[3], Richard W. Kriwacki[4], Joakim Andréasson[2], Morten Grøtli*[1]

[1]Department of Chemistry and Molecular Biology, University of Gothenburg, Gothenburg, Sweden

[2] Department of Chemical and Biological Engineering/ Chemistry and Biochemistry, Chalmers University of Technology, Gothenburg, Sweden.

[3]Department of Chemical Biology and Therapeutics, St. Jude Children's Research Hospital, 262 Danny Thomas Place, Memphis, United States

[4]Department of Structural Biology, St. Jude Children's Research Hospital, 262 Danny Thomas Place, Memphis, United States

[¶]These authors contributed equally to this work.

[*]Corresponding author (grotli@chem.gu.se)

Experimental details, ^{1}H and ^{13}C NMR spectra, absorption and emission measurements, conformational analysis, fluorescence polarisation assay.

Table of Contents

General considerations 5

General procedure A: Mitsunobu reaction in the 9-position 5

General procedure B: Mitsunobu reaction in the 2-position 5

General procedure C: Substitution of chloride with dimethylamine 6

General procedure D: 8-bromination of 2,6,9-substituted purines 6

2-Amino-9-*tert*-butoxycarbonyl-6-chloropurine 6

2-*tert*-Butoxycarbonylamino-6-chloropurine (1) 6

6-Chloro-2-*tert*-butoxycarbonylamino-9-isobutylpurine (2b) 7

2-(*tert*-Butoxycarbonylamino)-6-chloro-9-cyclophentylmethylpurine (2c) 7

6-Chloro-2-(N-napthenyl-*tert*-butoxycarbonylamino)-9-isobutylpurine (3b) 7

2-(N-Benzyl-N-*tert*-butoxycarbonylamino)-6-chloro-9-cyclopentylmethylpurine (3c) 8

6-Dimethylamino-9-isobutyl-2-naphtalenylaminopurine (4b) 8

2-Benzylamino-9-cyclophentylmethyl-6-dimethylaminopurine (4c) 8

8-Bromo-6-dimethylamino-9-isobutyl-6-naphtalenylaminopurine (5b) 8

2-Benzylamino-8-bromo-9-cyclophentylmethyl-6-dimethylaminopurine (5c) 9

2-Benzylamino-6-dimethylamino-8-ethynyl-9-(2-phenethyl)purine (6a) 9

6-Dimethylamino-8-ethynyl-9-isobutyl-6-naphtalenylaminopurine (6b) 9

2-Benzylamino-6-dimethylamino-9-cyclopentylmethyl-8-ethynylpurine (6c) 10

6-Dimethylamino-9-isobutyl-2-naphtalenylamino-8-(1-benzyl-1H-1,2,3-triazol-4-yl)purine (7a) 10

2-Benzylamino-6-dimethylamino-8-(1-isobutyl-1H-1,2,3-triazol-4-yl)-9-phenethylpurine (7b) 11

2-Benzylamino-8-(1-benzyl-1,2,3-triazol-4-yl)-6-dimethylamino-9-cyclopentylmethylpurine (7c) 11

8-(1-Benzyl-1H-1,2,3-triazol-5-yl)-6-dimethylamino-9-isobutyl-2-naphtalenylaminopurine (8a) 12

Benzyl azide[4] 12

6-Chloro-9-(2-indanyl)-2-(*tert*-butoxycarbonylamino)purine (9a) 13

6-Chloro-9-(2-indanyl)-2-(N-benzyl-tert-butoxycarbonylamino)purine (10a) 13

6-Chloro-9-(2-indanyl)-2-(N-propyl-tert-butoxycarbonylamino)purine (10b) 13

2-(N-*p*-Cl-Benzyl-N-*tert*-butoxycarbonylamino)-6-chloro-9-(2-indanyl)purine (10c) 13

9-Benzyl-2-(N-benzyl-*tert*-butoxycarbonylamino)-6-chloro-purine (10d) 14

2-Benzylamino-6-dimethylamino-9-(2-indanyl)purine (11a) 14

6-Dimetylamino-9-(2-indanyl)-2-(1-propylamino)purine (11b) 14

6-Dimethylamino-9-(2-indanyl)-2-(*p*-chloro-benzylamino)purine (11c) 15

2-Benzylamino-6-(ethoxy-carbonyl-methylamino)-9-(2-indanyl)purine (11d) 15

6-(Ethoxy-carbonyl-methylamino)-9-(2-indanyl)-2-(1-propylamino)purine (11e) 16

9-Benzyl-2-benzylamino-6-(ethoxy-carbonyl-methylamino)purine (11f) 16

9-Benzyl-2-benzylamino-6-(methoxy-carbonyl-methyl-thio)purine (11g) 17

2-Benzylamino-8-bromo-6-dimethylamino-9-(2-indanyl)purine (12a) 17

8-Bromo-6-dimetylamino-9-(2-indanyl)-2-(1-propylamino)purine (12b) 17

8-Bromo-6-dimethylamino-9-(2-indanyl)-2-(*p*-chloro-benzylamino)purine (12c) 18

2-Benzylamino-8-bromo-6-(ethoxy-carbonyl-methylamino)-9-(2-indanyl)purine (12d) 18

8-Bromo-6-(ethoxy-carbonyl-methylamino)-9-(2-indanyl)-2-(1-propylamino)purine (12e)............... 18
9-benzyl-2-benzylamino-8-bromo-6-(ethoxy-carbonyl-methylamino)purine (12f)............................. 19
9-Benzyl-2-benzylamino-8-bromo-6-(methoxy-carbonyl-methyl-thio)purine (12g)........................... 19
6-Amino-9-benzyl-2-benzylamino-8-bromopurine (12h) 19
6-Amino-8-bromo-2-(1-propylamino)purine (12i)... 20
2-Benzylamino-6-dimethylamino-8-ethynyl-9-(2-indanyl)purine (13a)... 20
6-Dimetylamino-8-ethynyl-9-(2-indanyl)-2-(1-propylamino)purine (13b)... 21
6-Dimethylamino-8-ethynyl-9-(2-indanyl)-2-(*p*-chloro-benzylamino)purine (13c) 21
2-Benzylamino-6-(ethoxy-carbonyl-methylamino)-8-ethynyl-9-(2-indanyl)purine (13d).................... 22
6-(Ethoxy-carbonyl-methylamino)-8-ethynyl-9-(2-indanyl)-2-(1-propylamino)purine (13e) 22
9-Benzyl-2-benzylamino-6-(ethoxy-carbonyl-methylamino)-8-ethynylpurine (13f) 23
9-Benzyl-2-benzylamino-8-ethynyl-6-(methoxy-carbonyl-methyl-thio)purine (13g) 23
6-Amino-9-benzyl-2-benzylamino-8-ethynylpurine (13h)... 23
6-Amino-8-ethynyl-9-(2-indanyl)-2-(1-propylamino)purine (13i) 24
2-Benzylamino-6-dimethylamino-9-(2-indanyl)-8-(1-methyl-1H-1,2,3-triazol-4-yl)purine (14a)....... 24
6-Dimetylamino-9-(2-indanyl)-8-(1-methyl-1H-1,2,3-triazol-4-yl)-2-(1-propylamino)purine (14b)... 25
6-Dimethylamino-9-(2-indanyl)-8-(1-methyl-1H-1,2,3-triazol-4-yl)-2-(*p*-chloro-benzylamino)purine (14c) 25
2-Benzylamino-6-(ethoxy-carbonyl-methylamino)-9-(2-indanyl)-8-(1-methyl-1,2,3-triazol-4-yl)purine (14d) 26
8-(1-Benzyl-1H-1,2,3-triazol-4-yl)-6-(ethoxy-carbonyl-methylamino)-9-(2-indanyl)-2-(1-propylamino)purine (14e).. 26
6-(Ethoxy-carbonyl-methylamino)-9-(2-indanyl)-8-(1-methyl-1H-1,2,3-triazol-4-yl)-2-(1-propylamino)purine (14f) 26
9-Benzyl-2-benzylamino-8-(1-benzyl-1,2,3-triazol-4-yl)-6-(ethoxy-carbonyl-methylamino)purine (14g) 27
9-Benzyl-2-benzylamino-8-(1-benzyl-1,2,3-triazol-4-yl)-6-(methoxy-carbonyl-methylthio)purine (14h) 28
6-Amino-9-benzyl-2-benzylamino-8-(1-methyl-1H-1,2,3-triazol-4-yl)-purine (14i) 28
6-Amino-9-(2-indanyl)-8-(1-methyl-1H-1,2,3-triazol-4-yl)-2-(1-propylamino)purine (14j)............... 28
2-Benzylamino-6-dimethylamino-8-(1-methyl-1H-1,2,3-triazol-4-yl)-9-phenethylpurine (14k)......... 29
2-Benzylamino-9-cyclophentylmethyl-6-dimethylamino-8-(1-methyl-1H-1,2,3-triazol-4-yl)purine (14l) 29
NOE build-up analysis 31
Computational conformation analysis 33
Ensamble analysis using NAMFIS.. 34
Emission quantum yield determination. 36
Absorption and emisson spectra.. 37
Fluorescence Polarisation (FP) Assay 39
WaterLOGSY measurements 40
Molecular modelling 41

^{1}H and ^{13}C NMR spectra 42
References 101

General considerations

All commercial chemicals were used without prior purification. Polymer supported fluoride (2.0-3.0 mmol/g, 20-50 mesh) was purchased from Sigma Aldrich. Polymer supported base Amberlite IRA-67 (5.6 mmol/g) was purchased from Sigma Aldrich. CH_2Cl_2 was distilled from calcium hydride and THF was distilled from sodium and benzophenone when dry solvents were used. All reactions were monitored by TLC (Merck silica gel 60 F_{254}) and analysed under UV (254 nm). Microwave reactions were performed in a Biotage Initiator reactor with fixed hold time. Column chromatography was performed by flash chromatography (wet-packed silica, 0.04–0.063 mm) or by automated column chromatography on a Biotage SP-4 instrument using pre-packed silica columns. ^1H and ^{13}C NMR spectra were obtained at 400 and 100 MHz respectively, using a Varian 400/54 spectrometer. The solvent peak was used as reference. LCMS analysis was performed on a API SCIEX 150 EX Perkin Elmer ESI-MS (30 eV) connected to a Perkin Elmer gradient pump system and a C8 column (Gemini) using acetonitrile and MilliQ-water with 1% formic acid as mobile phases with a gradient of 5 to 95% acetonitrile over 4 min. Analytical high-performance liquid chromatography (HPLC) analysis was carried out on a Waters separation module 2690 connected to a Waters photodiode array detector 996 using an Atlantis® 5 µm C18 AQ (250×4.6 mm) column. Preparative HPLC was carried out on a Waters 600 controller connected to a Waters 2487 Dual λ Absorbance detector using an Atlantis® Prep T3 5 µm C-18 (250×19 mm) column. HRMS analysis was performed on a Waters LCTp XE mass spectrometer with an Acquity UPLC BEH C18 (pH 10) or an Acquity UPLC CSH C18 (pH 3) column eluting with a gradient of 5-95% acetonitrile in MQ-water.

Abbreviations used: DMEDA for N,N′-Dimethylethylenediamine, DMF for dimethylformamide, $PyrBr_3$ for pyridinium tribromide, ADDP for 1,1'-(azodicarbonyl)dipiperidine, DIAD for diethyl azodicarboxylate, $PyrB_3$ for pyridinium tribromide.

Synthesis of 5a, 4a, 3a and 2a have recently been published and can be found in reference[1].

General procedure A: Mitsunobu reaction in the 9-position

The alkylation was performed following a published procedure[2] with minor modifications. **1** (1.0 eq.) was dissolved in dry THF under nitrogen in oven-dried round-bottomed flask and alcohol (1.1-2.5 eq.) and PPh_3 (1.1-2.5 eq.) was added. The nitrogen flow was temporarily suspended when solid alcohols were added. When all of the PPh_3 was dissolved, DIAD (1.0-2.5 eq.), was added drop wise. The reaction was stirred at room temperature under nitrogen, until TLC indicated full consumption of the starting material, unless otherwise noted. The solvent was removed under reduced pressure and the crude product was purified by flash column chromatography or automated flash column chromatography.

General procedure B: Mitsunobu reaction in the 2-position

The alkylation was performed following a published procedure[2] with minor modifications. The purine was dissolved in dry THF under nitrogen in an oven-dried round-bottomed flask, PBu_3 (2.4-2.6 eq.), alcohol (2.4-2.6 eq.) and ADDP (2.5-2.6 eq.) were added in that order. The nitrogen flow was temporarily removed when solid alcohols and ADDP were added. The reaction was stirred at room temperature, until TLC indicated full consumption of the starting material, unless otherwise noted. The obtained white precipitate was filtered off, washed with THF and the solvent was removed under reduced pressure. The crude product was purified by flash column chromatography or automated flash column chromatography.

General procedure C: Substitution of chloride with dimethylamine

Dimethylamine substitution of 6-chloropurines was performed following a published procedure[3] with modifications. The 6-chloropurines (1.0 eq.) were dissolved in dry DMF in a dry microwave vial without a magnetic stirring bar. The vial was capped and nitrogen was bubbled through the solution. The reaction mixture was heated in a microwave reactor at 180°C until the starting material was consumed according to TLC. The solvent was removed under reduced pressure, co-evaporated with toluene when needed and the crude product was purified by flash column chromatography.

General procedure D: 8-bromination of 2,6,9-substituted purines

Purine (1.0 eq.) was dissolved in dry CH_2Cl_2 in oven-dried glassware under nitrogen. The nitrogen flow was temporarily removed when pyridinium tribromide ($PyrBr_3$) (1.1-1.4 eq.) was added in one portion, the reaction mixture was stirred at room temperature under nitrogen until full conversion was observed by TLC or LCMS. The reaction was quenched with 10% $Na_2S_2O_3$ (aq.); the colour changed from yellow to colourless. The pH was adjusted to 9-12 with 15% NaOH (aq.). The phases were separated and the aqueous phase was extracted with CH_2Cl_2 (x 3). The organic phases were pooled, washed with brine, dried over Na_2SO_4 and filtered. The solvent was removed under reduced pressure and the crude product was purified by flash column chromatography.

2-Amino-9-*tert*-butoxycarbonyl-6-chloropurine

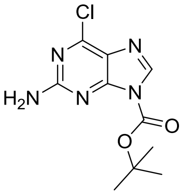

Following a published procedure[2] with minor modifications, 2-amino-6-chloropurine (2.50 g, 14.7 mmol) and di-*tert*-butyl dicarbonate (3.21 g, 14.7 mmol) were dissolved in anhydrous DMSO (50 ml). The pale yellow solution was cooled in an ice bath with vigorous stirring and the ice bath was removed just as the DMSO started to freeze. DMAP (90 mg, 0.74 mmol) was added and the solution was stirred under nitrogen at room temperature for 8 h. Additional *t*bocanhydride (220 mg, 1.00 mmol) was added and the reaction mixture was stirred at room temperature overnight. Full consumption of starting material and formation of one new spot was confirmed by TLC (10% methanol in $CHCl_3$).The reaction mixture was diluted with water (450 ml) and extracted with ethyl acetate (3 x 150 ml). The organic phases were washed with water (5 x 100 ml), dried over Na_2SO_4, filtered and evaporated to give the expected product as a white solid (3640 mg, 92%) which was used without further purification in in the next step. ^1H and ^{13}C NMR corresponds with previously published results[1]. ^1H NMR ($CDCl_3$): 8.13 (s, 1H), 5.92 (br s, 2H), 1.66 (s, 9H); ^{13}C NMR ($CDCl_3$): 160.6, 153.4, 152.4, 145.7, 140.2, 125.5, 87.2, 28.0.

2-*tert*-Butoxycarbonylamino-6-chloropurine (1)

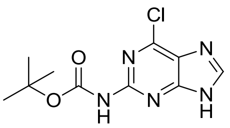

Sodium hydride (715 mg, 17.9 mmol, 60 % mineral oil dispersion) was added to a stirred solution of 1-amino-9-*tert*-butoxycarbonyl-6-chloropurine (3490 mg, 12.9 mmol) in dry THF (120 ml) at room temperature under nitrogen. The white suspension was stirred at room temperature for 2 h. Full conversion of starting material was confirmed by TLC (70% ethyl acetate in pentane). The reaction was cooled to 0°C and quenched with brine (5 ml), a white precipitate was observed after the addition. The mixture was then allowed to reach room temperature. The THF was removed under reduced pressure (note: save 10-20 ml THF, simplifies the extraction). $CHCl_3$ (200 ml) and distilled water (800 ml) were added (everything did not dissolve). The pH on the aqueous phase was determined to 14. The phases were separated and extracted with $CHCl_3$ (3 x 100

ml, note: in between every extraction wait approximately 10 min) and these organic phases were discarded. Sat. $NaHCO_3$ (aq.) was added to the aqueous phase whereupon a white precipitate was observed and the aqueous phase was extracted with $CHCl_3$ (3 x 100 ml). The combined organic phases were washed with brine. The solvent was removed under reduced pressure to yield **1** as a white solid in quantitative yield. ^{1}H and ^{13}C NMR corresponds with previously published results[1]. ^{1}H NMR ($CDCl_3$): 13.53 (s, 1H), 8.44 (s, 1H), 7.78 (s, 1H), 1.58 (s, 9H); ^{13}C NMR ($CDCl_3$): 153.3, 151.6, 151.3, 151.0, 145.5, 128.3, 82.4, 28.3.

6-Chloro-2-*tert*-butoxycarbonylamino-9-isobutylpurine (2b)

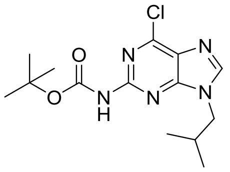

Compound **2b** was synthesised following general procedure A from **1** (1500 mg, 5.56 mmol) isobutyl alcohol (0.55 ml, 5.94 mmol), PPh_3 (1605 mg, 6.12 mmol) and DIAD (1.1 ml, 5.6 mmol) in dry THF (75 ml). Full consumption of starting material was confirmed by TLC (eluting first with 50% ethyl acetate in heptane and then with 10% methanol in $CHCl_3$) after 1.5 h. The crude was purified by flash column chromatography (dryloaded from CH_2Cl_2 on Celite®, 40% ethyl acetate in heptane) to provide **2b** as a white solid (1646 mg, 91%). ^{1}H NMR ($CDCl_3$): 7.90 (s, 1H), 7.65 (s, 1H), 3.99 (d, *J* 7.3 Hz, 2H), 2.24 (m, 1H), 1.50 (s, 9H), 0.91 (d, *J* 6.7 Hz, 6H); ^{13}C NMR ($CDCl_3$): 153.2, 152.4, 151.2, 150.3, 144.6, 127.7, 81.6, 51.5, 28.9, 28.3, 20.0. LRMS *m/z* $[M + H]^+$ calculated for $C_{14}H_{20}ClN_5O_2$: 326.1 Found: 326.7.

2-(*tert*-Butoxycarbonylamino)-6-chloro-9-cyclophentylmethylpurine (2c)

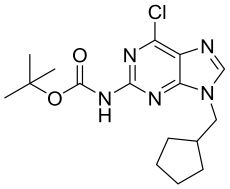

Compound **2c** was synthesised following general procedure A from **1** (910 mg, 3.37 mmol), cyclopentylmethanol (0.38 ml, 3.51 mmol), PPh_3 (964 mg, 3.68 mmol) and DIAD (0.7 ml, 3.55 mmol) dry THF (55 ml). The crude product was purified by automated flash column chromatography (42% ethyl acetate in heptane) to provide **2c** as a white solid (1022 mg, 86%). ^{1}H NMR ($CDCl_3$): 7.95 (s, 1H), 7.43 (s, 1H), 4.13 (d, *J* 7.6 Hz, 2H), 2.57 – 2.37 (m, 1H), 1.83 – 1.64 (m, 4H), 1.64 – 1.56 (m, 2H), 1.55 (s, 9H), 1.37 – 1.17 (m, 2H); ^{13}C NMR ($CDCl_3$): 153.2, 152.4, 151.2, 150.4, 144.3, 127.8, 81.7, 49.1, 40.2, 30.6, 28.4, 25.1. HRMS *m/z* $[M + H]^+$ calculated for $C_{16}H_{22}ClN_5O_2$: 352.1540. Found: 352.1528.

6-Chloro-2-(N-napthenyl-*tert*-butoxycarbonylamino)-9-isobutylpurine (3b)

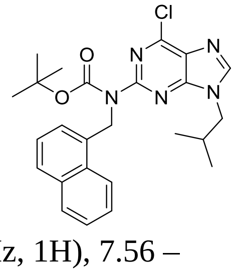

Compound **3b** was synthesised following general procedure B from **2b** (771 mg, 2.37 mmol), 1-naphtylmethanol (936 mg, 5.92 mmol), PBu_3 (1.5 ml, 6.0 mmol) and ADDP (1506 mg, 5.97 mmol) in dry THF (40 ml). The reaction mixture was stirred at room temperature for 19 h. Full consumption of starting material was confirmed by TLC (40% ethyl acetate in heptane). Purification by flash column chromatography (25 to 40% ethyl acetate in hexane) provided **3b** as a colourless foamy sticky solid (1000 mg, 91%). ^{1}H NMR ($CDCl_3$): 8.15 (app d, *J* 8.2 Hz, 1H), 7.89 (s, 1H), 7.82 (app d, *J* 8.1 Hz, 1H), 7.68 (d, *J* 8.2 Hz, 1H), 7.56 – 7.44 (m, 3H), 7.32 (dd, *J* 8.2, 7.2 Hz, 1H), 5.69 (s, 2H), 3.84 (d, *J* 7.2 Hz, 4H), 2.10 – 1.95 (m, 1H), 1.48 (s, 9H), 0.77 (d, *J* 6.7 Hz, 6H); ^{13}C NMR ($CDCl_3$): 155.0, 154.2, 152.7, 150.6, 145.2, 133.7, 133.6, 131.3, 128.8, 128.4, 127.5, 126.1, 125.6, 125.4, 124.7, 123.2, 82.1, 51.6, 49.2, 29.0, 28.3, 19.9. HRMS *m/z* $[M + H]^+$ calculated for $C_{25}H_{28}ClN_5O_2$: 465.201 Found: 466.2029

2-(N-Benzyl-N-*tert*-butoxycarbonylamino)-6-chloro-9-cyclopentylmethylpurine (3c)

Compound **3c** was synthesised following general procedure B from **2c** (791 mg, 2.25 mmol), benzyl alcohol (0.58 ml, 5.6 mmol), PBu_3 (1.4 ml, 5.3 mmol) and ADDP (1420 mg, 5.62 mmol) in dry THF (40 ml). The reaction was stirred overnight at room temperature whereupon full consumption of starting material was confirmed by TLC (50% ethyl acetate in pentane). The crude product was purified by automated flash column chromatography (16% ethyl acetate in pentane) to provide **3c** as a white crystalline solid (859 mg, 92%). ^{1}H NMR ($CDCl_3$): 7.97 (s, 1H), 7.38 – 7.34 (m, 2H), 7.27 – 7.22 (m, 2H), 7.20 – 7.22 (m, 1H), 5.18 (s, 2H), 4.07 (d, *J* 7.6 Hz, 2H), 2.45 – 2.34 (m, 1H), 1.71 – 1.62 (m, 4H), 1.60 – 1.52 (m, 2H), 1.47 (s, 9H), 1.22 (m, 2H); ^{13}C NMR ($CDCl_3$): 155.1, 154.0, 152.7, 150.4, 144.8, 138.6, 128.3, 128.2, 127.6, 127.0, 81.9, 77.2, 51.6, 49.0, 40.3, 30.5, 28.2, 25.0. HRMS *m/z* $[M + H]^+$ calculated for $C_{23}H_{28}ClN_5O_2$: 442.2010. Found: 442.2011.

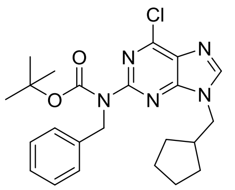

6-Dimethylamino-9-isobutyl-2-naphtalenylaminopurine (4b)

Compound **4b** was synthesised following general procedure C from **3b** (970 mg, 2.08 mmol) in dry DMF (18 ml). The reaction was heated in a microwave reactor at 180 ºC for 50 min. Full consumption of starting material was confirmed by TLC (50% ethyl acetate in heptane). Purification by flash column chromatography (15% methanol in $CHCl_3$) provided **4b** as a pale yellow foam (730 mg, 94%). ^{1}H NMR ($CDCl_3$): 8.23 – 8.16 (m, 1H), 7.90 – 7.83 (m, 1H), 7.77 (d, *J* 8.3, 1H), 7.57 – 7.46 (m, 3H), 7.43 (s, 1H), 7.42 – 7.38 (m, 1H), 5.09 (br s, 3H), 3.82 (d, *J* 7.2 Hz, 2H), 3.44 (br s, 6H), 2.29-2.14 (m, 1H), 0.91 (d, *J* 6.7 Hz, 6H); ^{13}C NMR ($CDCl_3$): 158.8, 155.3, 153.3, 136.4, 135.8, 133.9, 131.9, 128.7, 127.9, 126.2, 126.2, 125.7, 125.5, 124.1, 115.0, 50.7, 44.1, 38.3, 29.0, 20.1. HRMS *m/z* $[M + H]^+$ calculated for $C_{22}H_{26}N_6$: 375.2297 Found: 375.2305

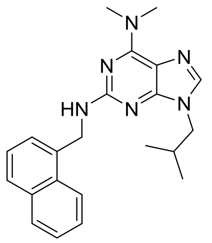

2-Benzylamino-9-cyclophentylmethyl-6-dimethylaminopurine (4c)

Compound **4c** was synthesised following general procedure C from **3c** (355 mg, 0.80 mmol) in dry DMF (10 ml). The crude product was purified by automated flash column chromatography (1% methanol in $CHCl_3$) to provide **3c** as a light yellow oil (230 mg, 83%). ^{1}H NMR ($CDCl_3$): 7.43 (s, 1H), 7.38 – 7.33 (m, 2H), 7.31 – 7.23 (m, 2H), 7.23 – 7.16 (m, 1H), 5.23 (t, *J* 6.0 Hz, 1H), 4.62 (d, *J* 6.0 Hz, 2H), 3.89 (d, *J* 7.6 Hz, 2H), 3.42 (br s, 6H), 2.40 (sept, *J* 7.6 Hz, 1H), 1.75 – 1.58 (m, 4H), 1.57 – 1.46 (m, 2H), 1.29 – 1.17 (m, 2H); ^{13}C NMR ($CDCl_3$): 158.8, 155.1, 153.1, 140.7, 135.9, 128.2, 127.6, 126.7, 114.8, 47.8, 46.0, 40.1, 38.1, 30.3, 25.8. HRMS *m/z* $[M + H]^+$ calculated for $C_{20}H_{27}N_6$: 351.2297. Found: 351.2289.

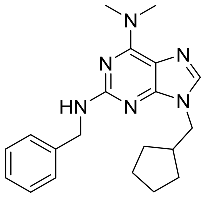

8-Bromo-6-dimethylamino-9-isobutyl-6-naphtalenylaminopurine (5b)

Compound **5b** was synthesised following general procedure D from **4b** (686 mg, 1.83mmol) and $PyrBr_3$ (645 mg, 2.02 mmol) in dry CH_2Cl_2 (35 ml). The reaction mixture was stirred at room temperature for 3 h. Full consumption of starting material was confirmed by TLC (50% ethyl acetate in heptane). Purification by flash column chromatography (10%, then 40% ethyl acetate in hexane) provided **5b** as a white solid (697 mg, 84%). ^{1}H NMR ($CDCl_3$): 8.27 – 8.11 (m, 1H), 7.95 – 7.82 (m, 1H), 7.77 (d, *J* 8.2 Hz, 1H), 7.58 – 7.45 (m,

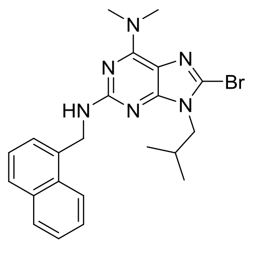

3H), 7.44 – 7.36 (m, 1H), 5.15 (t, *J* 5.5 Hz, 1H), 5.06 (d, *J* 5.5 Hz, 2H), 3.81 (d, *J* 7.4 Hz, 2H), 3.39 (br s, 6H), 2.38 – 2.22 (m, 1H), 0.91 (d, *J* 6.7 Hz, 6H); ^{13}C NMR ($CDCl_3$): 158.5, 154.4, 154.1, 135.6, 133.9, 131.9, 128.7, 128.0, 126.3, 126.2, 125.8, 125.5, 124.0, 121.0, 115.4, 51.0, 44.1, 38.3, 28.6, 20.1. LRMS *m/z* $[M + H]^+$ calculated for $C_{22}H_{25}BrN_6$: 453.1 Found: 453.2.

2-Benzylamino-8-bromo-9-cyclophentylmethyl-6-dimethylaminopurine (5c)

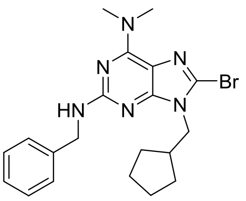

Compound **5c** was synthesised following general procedure D from **4c** (506 mg, 1.44 mmol) and $PyrBr_3$ (608 mg, 1.90 mmol) as the substrates in dry CH_2Cl_2 (35 ml). The reaction was stirred for 2 h at room temperature. The crude was purified by automated flash column chromatography (6-20% ethyl acetate in pentane) to provide **5c** as a colourless oil (556 mg, 90%). ^{1}H NMR ($CDCl_3$): 7.39 – 7.34 (m, 2H), 7.33 – 7.27 (m, 2H), 7.25 – 7.20 (m, 2H), 5.15 (br s, 1H), 4.61 (d, *J* 6.0 Hz, 2H), 3.93 (d, *J* 7.7 Hz, 2H), 3.39 (br s, 6H), 2.50 (sep, *J* 7.6 Hz, 1H), 1.73 – 1.44 (m, 6H), 1.39 – 1.25 (m, 2H); ^{13}C NMR ($CDCl_3$): 158.6, 154.3, 154.1, 140.6, 128.4, 127.7, 126.9, 120.8, 115.4, 48.2, 46.0, 39.9, 38.3, 30.1, 24.9. HRMS *m/z* $[M + H]^+$ calculated for $C_{20}H_{25}BrN_6$: 429.1402. Found: 429.1418.

2-Benzylamino-6-dimethylamino-8-ethynyl-9-(2-phenethyl)purine (6a)

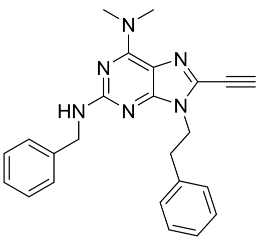

The synthesis of 2-benzylamino-8-bromo-6-dimethylamino-9-phenethylpurine has previously been published[1]. $PdCl_2(PPh_3)_2$ (46 mg, 0.07 mmol), CuI (50 mg, 0.26 mmol) and amberlite IRA-67 (1176 mg, 6.59 mmol) were added to a solution of 2-benzylamino-8-bromo-6-dimethylamino-9-phenethylpurine (594 mg, 1.32 mmol) in dry THF (13 ml). The vial was capped, nitrogen was bubbled through the reaction mixture and ethynyltrimethylsilane (750 µl, 5.42 mmol) was added. The yellow reaction mixture was heated in microwave reactor at 110°C for 50 min, after which full consumption of starting material was confirmed by LCMS. The dark reaction mixture was filtered through a short plug of silica which was eluted with THF. The solvent volume was reduced to 15 ml and polymer supported fluoride (694 mg) was added. The reaction mixture was stirred under nitrogen overnight. Full deprotection was confirmed by TLC (50% ethyl acetate in hexane) and LCMS. The polymer was filtered off, washed with THF and CH_2Cl_2 and the solvents were removed under reduced pressure. Purification by automated flash column chromatography (0-50% ethyl acetate in hexane) provided **6a** as a brown sticky foam (383 mg, 73%) after co-evaporation with DEE (x3). ^{1}H NMR ($CDCl_3$): 7.44 – 7.37 (m, 2H), 7.36 – 7.19 (m, 6H), 7.18 – 7.13 (m, 2H), 5.15 (br t, *J* 5.5 Hz, 1H), 4.65 (d, *J* 5.9 Hz, 2H), 4.37 – 4.28 (m, 2H), 3.43 (s, 6H), 3.32 (s, 1H), 3.14-3.04 (m, 2H); ^{13}C NMR ($CDCl_3$): 159.6, 155.0, 152.6, 140.5, 138.1, 129.0, 128.62 (two carbons, elucidated by gHMBC), 128.46, 127.68, 126.96, 126.70, 115.16, 81.65, 74.01, 46.06, 44.52, 38.42, 35.72. HRMS *m/z* [M + H]$^+$ calculated for $C_{24}H_{24}N_6$: 397.2141. Found: 397.2137.

6-Dimethylamino-8-ethynyl-9-isobutyl-6-naphtalenylaminopurine (6b)

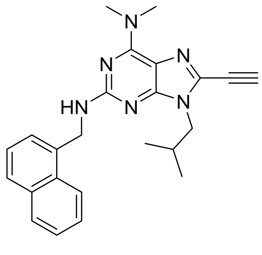

$Pd(PPh_3)_2Cl_2$ (41 mg, 0.06 mmol), CuI (44 mg, 0.23 mmol) and amberlite IRA-67 (1043 mg, 5.84 mmol) were added to a solution of **5b** (528 mg, 1.65 mmol) in dry THF (12 ml). The vial was capped, nitrogen was bubbled through the reaction mixture and ethynyltrimethylsilane (0.65 ml, 4.70 mmol) was added. The yellow reaction mixture was heated in microwave reactor at 110°C for 50 min. Full consumption of starting material was

confirmed by LCMS. The dark reaction mixture was filtered through a short plug of silica which was eluted with THF, the solvent volume was reduced and adjusted to 15 ml and polymer supported fluoride (612 mg) was added. The reaction mixture was stirred under nitrogen at room temperature overnight. Complete deprotection was confirmed by TLC (15% ethyl acetate in hexane). The polymer was filtered off, washed with THF and the solvents were removed under reduced pressure. Purification by automated flash column chromatography (0-10% ethyl acetate in heptane) provided **6b** as a pale brown foam (341 mg, 74%). ^{1}H NMR ($CDCl_3$): 8.21 – 8.14 (m, 1H), 7.90 – 7.84 (m, 1H), 7.80-7.75 (m, 1H), 7.56 – 7.45 (m, 3H), 7.41 (dd, *J* 8.2, 7.0 Hz, 1H), 5.13 (br s, 1H), 5.08 (d, *J* 4.4 Hz, 2H), 3.92 (d, *J* 7.5 Hz, 2H), 3.44 (br s, 6H) 3.38 (s, 1H), 2.41-2.25 (m, 1H), 0.91 (d, *J* 6.7 Hz, 6H); ^{13}C NMR ($CDCl_3$) 159.3, 155.1, 153.0, 135.5, 134.0, 131.8, 129.2, 128.8, 128.0, 126.3 (two carbons, elucidated by HSQC), 125.8, 125.5, 124.0, 115.1, 81.8, 74.6, 50.5, 44.1, 38.5, 28.8, 20.2. HRMS *m/z* $[M + H]^+$ calculated for $C_{24}H_{26}N_6$: 399.2297 Found: 399.2310.

2-Benzylamino-6-dimethylamino-9-cyclopentylmethyl-8-ethynylpurine (6c)

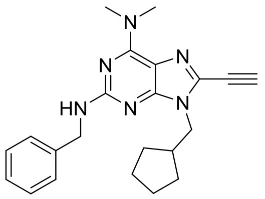

Amberlite IRA-67 (619 mg, 3.47 mmol), CuI (22 mg, 0.12 mmol) and $PdCl_2(PPh_3)_2$ (21 mg, 0.030 mmol) were added to a solution of **5c** (310 mg, 0.722 mmol) in THF (9 ml). The capped microwave vial was bubbled with nitrogen and ethynyltrimethylsilane (0.30 ml, 2.2 mmol) was added. The reaction mixture was heated in the microwave reactor at 110 °C for 50 min. Reaction was monitored by TLC (15% ethyl acetate in pentane). The reaction mixture was filtered through a short plug of silica and eluted with THF. The solvent volume was adjusted to 12 ml, polymer supported fluoride (386 mg) was added and the reaction was stirred at room temperature overnight. Full consumption of starting material was indicated by TLC (15% ethyl acetate in pentane). The polymer was filtered off and washed with CH_2Cl_2 and THF. The solvents were removed and the crude product was purified by automated flash column chromatography (0-15% ethyl acetate in pentane) to provide **6c** as a brown solid (155 mg, 57%). ^{1}H NMR ($CDCl_3$): 7.41 – 7.34 (m, 2H), 7.33 – 7.27 (m, 2H), 7.25 – 7.19 (m, 1H), 5.24 – 5.16 (m, 1H), 4.62 (d, *J* 6.0 Hz, 2H), 4.02 (d, *J* 7.7 Hz, 2H), 3.43 (br s, 6H), 3.38 (s, 1H), 2.53 (sep, *J* 7.5 Hz, 1H), 1.73 – 1.57 (m, 4H), 1.57 – 1.46 (m, 2H), 1.38 – 1.25 (m, 2H); ^{13}C NMR ($CDCl_3$): 159.5, 155.0, 152.9, 140.5, 129.0, 128.4, 127.7, 126.9, 115.1, 81.7, 74.6, 47.7, 46.1, 40.0, 38.5, 30.2, 24.9. HRMS *m/z* $[M + H]^+$ calculated for $C_{22}H_{26}N_6$: 375.2297. Found: 375.2285.

6-Dimethylamino-9-isobutyl-2-naphtalenylamino-8-(1-benzyl-1H-1,2,3-triazol-4-yl)purine (7a)

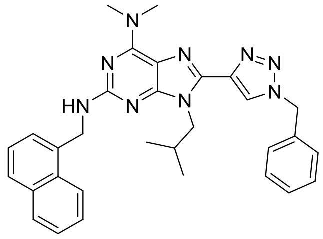

Sodium ascorbate (11 mg, 0.056 mmol), CuI (5 mg, 0.026 mmol), benzyl azide (31 mg, 0.23 mmol) and DMEDA (7 µl, 0.065 mmol) were added in that order to a solution of **6b** (55 mg, 0.14 mmol) in dry DMF (1.5 ml) in a microwave vial. The vial was capped, flushed with nitrogen and stirred at room temperature for 19 h after which full consumption of the purine was confirmed by TLC (50% ethyl acetate in heptane). The reaction mixture was diluted with water (20 ml) which resulted in a milky white precipitation. The aqueous phase was extracted with ethyl acetate (4 x 10 ml), the organic phases were washed with brine, dried over Na_2SO_4 and the solvents were removed under reduced pressure. Purification by automated flash column chromatography (0-50% ethyl acetate in heptane) provided **7a** as a pale green foam (40 mg, 55%). ^{1}H NMR ($CDCl_3$): 8.25 – 8.19 (m, 1H), 8.04 (s, 1H), 7.89 – 7.84 (m, 1H), 7.77 (app d,

J 8.3 Hz, 1H), 7.55 (dd, *J* 7.0, 1. Hz, 1H), 7.53 – 7.45 (m, 2H), 7.44 – 7.29 (m, 6H), 5.57 (s, 2H), 5.11 (s, 2H), 4.49 (d, *J* 7.4 Hz, 2H), 3.43 (br s, 6H), 2.40-2.24 (m, 1H), 0.89 (d, *J* 6.7 Hz, 6H); ^{13}C NMR ($CDCl_3$): 158.6, 155.0, 154.8, 142.0, 137.8, 135.8, 134.3, 133.9, 131.9, 129.3, 129.0, 128.7, 128.4, 127.9, 126.3, 126.2, 125.7, 125.5, 124.1, 123.4, 114.9, 54.4, 50.3, 44.1, 38.3, 29.2, 20.1. HRMS *m/z* $[M + H]^+$ calculated for $C_{31}H_{33}N_9$: 532.2937. Found: 532.2950.

2-Benzylamino-6-dimethylamino-8-(1-isobutyl-1H-1,2,3-triazol-4-yl)-9-phenethylpurine (7b)

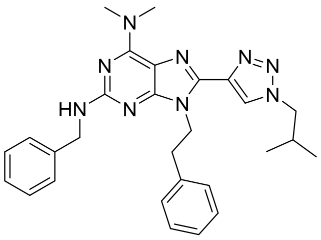

Isobutyl bromide (35 µl, 0.32 mmol) and sodium azide (21 mg, 0.32 mmol) in dry DMF (2 ml) were heated under nitrogen in a microwave reactor at 100 °C for 30 min. The vial was opened and **6a** (61 mg, 0.15 mmol) in dry DMF (1 ml), sodium ascorbate (9.4 mg, 0.05 mmol), CuI (5.2 mg, 0.03 mmol) and DMEDA (4 µl, 0.04 mmol) were added in that order. The vial was recapped, flushed with nitrogen and stirred at room temperature for 22 h. The reaction mixture was diluted with water (20 ml) which gave a white suspension which was extracted with ethyl acetate (4 x 10 ml), the organic phases were washed with water (2 x 10 ml), dried over $MgSO_4$ and solvents were removed under reduced pressure. Purification by automated flash column chromatography (0-100% ethyl acetate in pentane) gave the product as a pale yellow thick oil (61 mg, 80%) which solidified when left at room temperature. ^1H NMR ($CDCl_3$): 7.94 (d, *J* 0.9 Hz, 1H), 7.47 – 7.41 (m, 2H), 7.37 – 7.29 (m, 2H), 7.28 – 7.11 (m, 6H), 5.11 (br s, 1H), 4.89 – 4.79 (m, 2H), 4.68 (d, *J* 5.9 Hz, 2H), 4.20 (d, *J* 7.2 Hz, 2H), 3.46 (br s, 6H), 3.18 – 3.06 (m, 2H), 2.36 – 2.21 (m, 1H), 1.00 (d, *J* 6.7 Hz, 6H); ^{13}C NMR ($CDCl_3$): 159.0, 155.0, 154.3, 141.1, 140.7, 138.9, 137.5, 129.3, 128.5, 128.4, 127.8, 126.9, 126.3, 123.6, 115.0, 57.8, 46.2, 44.9, 38.3, 36.2, 29.8, 20.0. HRMS *m/z* $[M + H]^+$ calculated for $C_{28}H_{33}N_9$: 496.2937. Found: 496.2961.

2-Benzylamino-8-(1-benzyl-1,2,3-triazol-4-yl)-6-dimethylamino-9-cyclopentylmethylpurine (7c)

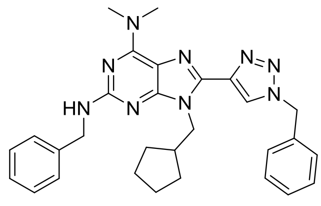

Benzyl bromide (35 µl, 0.30 mmol) and sodium azide (21 mg, 0.32 mmol) in dry DMF (0.7 ml) were heated in a microwave reactor at 100 °C for 40 min. The resulting benzyl azide solution was transferred to a microwave vial containing **6c** (52 mg, 0.14 mmol), sodium ascorbate (11 mg, 0.056 mmol), CuI (6 mg, 0.03 mmol) and DMEDA (7 µl, 0.07 mmol) in dry DMF (1.5 ml). The reaction mixture was stirred at room temperature for overnight. The reaction was monitored by TLC (30% ethyl acetate in pentane). The reaction mixture was then diluted with water (20 ml) and extracted with ethyl acetate (4 x 20 ml). *Caution: Keep aqueous phase slightly basic (0.5 M NaOH (aq.) was added to the aqueous phase) to avoid hydrazoic acid formation.* The combined organic phases were washed with brine and dried over Na_2SO_4. The solvents were removed by co-evaporation with toluene and the crude was purified by automated flash column chromatography (25-30% ethyl acetate in pentane) to provide **7c** as a pale yellow solid (58 mg, 82%). ^1H NMR ($CDCl_3$): 8.01 (s, 1H), 7.44 – 7.16 (m, 10H), 5.55 (s, 2H), 5.12 (br s, 1H) 4.63 (d, *J* 5.9 Hz, 2H), 4.56 (d, *J* 7.7 Hz, 2H), 3.41 (br s, 6H), 2.52 (sep, *J* 7.5 Hz, 1H), 1.67 – 1.49 (m, 4H), 1.48 – 1.36 (m, 2H), 1.35 – 1.23 (m, 2H); ^{13}C NMR ($CDCl_3$): 158.8, 155.0, 154.7, 142.0, 140.8, 137.6, 134.4, 129.3, 129.0, 128.4, 128.4, 127.8, 126.9, 123.4, 115.0, 54.4, 47.7, 46.2, 40.5, 38.3, 30.1, 25.0. HRMS *m/z* $[M + H]^+$ calculated for $C_{29}H_{33}N_9$: 508.2937. Found: 508.2927.

8-(1-Benzyl-1H-1,2,3-triazol-5-yl)-6-dimethylamino-9-isobutyl-2-naphtalenylaminopurine (8a)

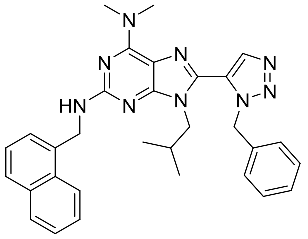

A solution of **6b** (86 mg, 0.22 mmol) and benzyl azide (52 mg, 0.39 mmol) in dry DMF (1 ml) were added to a suspension of Cp*RuCl(PPh_3)$_2$ (17 mg, 0.021 mmol) in dry DMF (1 ml) in a microwave vial. The vial was capped, flushed with nitrogen (5 min) and heated in a microwave reactor at 110ºC for 60 min. Full consumption of starting material was confirmed by TLC (50% ethyl acetate in heptane) and LCMS. The solvent was removed under reduced pressure and co-evaporated with toluene (x 3). The crude product was purified by flash column chromatography (25-100 % ethyl acetate in heptane), then precipitated from water and acetonitrile which resulted in compound **8a** as a pale yellow solid (25 mg, 22%). ^{1}H NMR ($CDCl_3$): 8.24 – 8.13 (m, 1H), 7.93 (s, 1H), 7.90 – 7.84 (m, 1H), 7.78 (d, *J* 8.3 Hz, 1H), 7.53 (d, *J* 7.0 Hz, 1H), 7.51 – 7.46 (m, 2H), 7.23 – 7.18 (m, 3H), 7.15 – 7.10 (m, 2H), 6.02 (s, 2H), 5.23(br s, 1H), 5.08 (d, *J* 5.5 Hz, 2H), 3.80 (d, *J* 7.6 Hz, 2H), 3.45 (br s, 6H), 1.81 (m, 1H), 0.60 (d, *J* 6.7 Hz, 6H); ^{13}C NMR ($CDCl_3$): 158.9, 155.2, 154.2, 135.6, 135.4, 133.9, 133.5, 133.3, 131.8, 128.8, 128.7, 128.2, 128.1, 127.8, 127.7, 126.3, 126.2, 125.8, 125.5, 123.9, 115.3, 53.1, 50.1, 44.0, 38.5, 28.7, 19.8. HRMS *m/z* [M + H]$^+$ calculated for $C_{31}H_{33}N_9$: 531.2859. Found: 532.2950

2-Benzylamino-6-dimethylamino-8-(1-isobutyl-1H-1,2,3-triazol-5-yl)-9-phenethylpurine (8b)

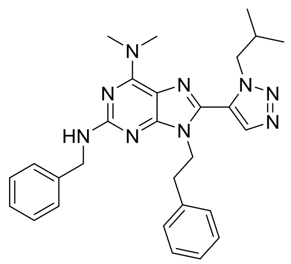

Isobutyl bromide (24 µl, 0.22 mmol) and sodium azide (14 mg, 0.22 mmol) in DMF (1.5 ml) were heated under nitrogen in a microwave reactor at 100°C for 30 min. **6a** (42 mg, 0.11 mmol) was added and to the solution was added a suspension of Cp*RuCl(PPh_3)$_2$ (8.5 mg, 0.01 mmol) in DMF (1 ml). The vial was recapped, flushed with nitrogen (4 min) and heated in a microwave reactor at 110 °C for 5 h. Full consumption of alkyne was observed by TLC (pentane:EtOAc 2:1). Solvent was removed under reduced pressure. Flash column chromatography (the crude was dryloaded on Celite from ethyl acetate, 33 to100% ethyl acetate in hexane) followed by preparative column chromatography (C-18 column, 50 to 100% MeCN in water buffered with 0.1% TFA) provided **8b** (2 mg, 4%). ^{1}H NMR ($CDCl_3$): 7.50 (s, 1H), 7.46 – 7.40 (m, 2H), 7.38 – 7.29 (m, 2H), 7.25 – 7.17 (m, 3H), 7.03 – 6.96 (m, 2H), 5.21 (br s, 1H), 4.69 (d, *J* 6.0 Hz, 2H), 4.34 (d, *J* 7.3 Hz, 2H), 4.30 (t, *J* 7.4 Hz, 2H), 3.46 (br s, 6H), 3.07 (t, *J* 7.3 Hz, 2H), 2.28 – 2.14 (m, 1H), 0.85 (d, *J* 6.7 Hz, 6H); ^{13}C NMR ($CDCl_3$): 159.2, 155.1, 153.8, 140.4, 137.5, 133.5, 132.8, 128.8, 128.7, 128.4, 127.6, 127.2, 127.0, 126.9, 115.5, 56.3, 46.0, 44.8, 38.4, 35.3, 29.2, 19.8. . HRMS *m/z* [M + H]$^+$ calculated for $C_{28}H_{33}N_9$: 496.2937. Found: 496.2932.

Benzyl azide[4]

NaN_3 (131 mg, 2.02 mmol), MQ-water (2 ml) and benzyl bromide (120 µl, 1.01 mmol) were added in that order to a 2-5 ml microwave vial which was capped, flushed with nitrogen and heated in a microwave reactor at 120°C for 40 min. The reaction mixture was allowed to cool to ambient temperature and was then extracted with DEE (3 x 5 ml) (extraction was performed with glass pipette in the reaction vial). The collected organic phases were dried over Na_2SO_4, gravity filtrated in to a plastic falcon vial, and the solvents were removed under reduced pressure to provide benzyl azide as a clear liquid (110 mg, 82%). ^{1}H NMR ($CDCl_3$)

corresponded to the published data and the product was used in the cyclization reactions without further purification). ^{1}H NMR ($CDCl_3$): 7.43-7.30 (m, 5H), 4.35 (s, 2H).

6-Chloro-9-(2-indanyl)-2-(*tert*-butoxycarbonylamino)purine (9a)

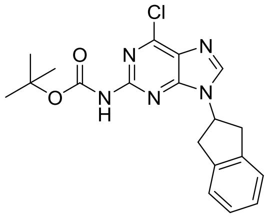

Compound **9a** was synthesised following general procedure A at 0 °C for 2 h from **1** (816 mg, 3.03 mmol), 2-indanol (1031 mg, 7.60 mmol), PPh_3 (1953 mg, 7.44 mmol) and DIAD (1.45 ml, 7.36 mmol) as substrates in dry THF (42 ml). The crude product was purified by flash column chromatography (30-70 % ethyl acetate in pentane) to provide **9a** as a white solid (970 mg, 83%). ^{1}H NMR ($CDCl_3$): 7.73 (s, 1H), 7.53 (br s, 1H), 7.34 – 7.25 (m, 4H), 5.55 (tt, *J* 7.4, 4.3 Hz, 1H), 3.63 (dd, *J* 16.5, 7.4 Hz, 2H), 3.26 (dd, *J* 16.4, 4.3 Hz, 2H), 1.54 (s, 9H); ^{13}C NMR ($CDCl_3$): 152.6, 152.3, 151.1, 150.2, 141.9, 139.6, 127.6, 127.5, 124.8, 81.7, 55.0, 40.1, 28.2. HRMS *m/z* $[M + H]^+$ calculated for $C_{19}H_{20}ClN_5O_2$: 386.1384. Found: 386.1351.

6-Chloro-9-(2-indanyl)-2-(N-benzyl-tert-butoxycarbonylamino)purine (10a)

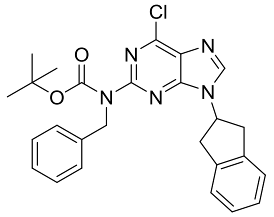

Compound **10a** was synthesised following general procedure B from **9a** (426 mg, 1.10 mmol), benzyl alcohol (0.29 ml, 2.75 mmol), PBu_3 (0.73 ml, 2.76 mmol) and ADDP (698 mg, 2.77 mmol) in dry THF (25 ml). The reaction was stirred overnight at room temperature. The crude product was purified by automated flash column chromatography (28% ethyl acetate in pentane) to provide **10a** as a white crystalline solid (496 mg, 94%). ^{1}H NMR ($CDCl_3$): 7.82 (s, 1H), 7.37 – 7.34 (m, 2H), 7.31 – 7.15 (m, 7H), 5.39 (tt, *J* 7.5, 5.2 Hz, 1H), 5.18 (s, 2H), 3.55 (dd, *J* 16.3, 7.5 Hz, 2H), 3.31 (dd, *J* 16.2, 5.2 Hz, 2H), 1.48 (s, 9H); ^{13}C NMR ($CDCl_3$): 154.9, 154.0, 152.3, 150.5, 142.8, 139.6, 138.6, 128.4, 128.3, 127.7, 127.6, 127.0, 124.9, 82.0, 55.6, 51.6, 39.6, 28.2. HRMS *m/z* $[M + H]^+$ calculated for $C_{26}H_{26}ClN_5O_2$: 476.1853. Found: 476.1867.

6-Chloro-9-(2-indanyl)-2-(N-propyl-tert-butoxycarbonylamino)purine (10b)

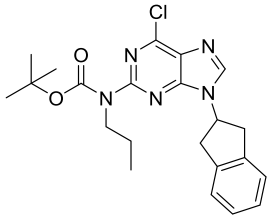

Compound **10b** was synthesised following general procedure B from **9a** (2930 mg, 7.59 mmol), *n*-propanol (1.45 ml, 19.40 mmol), PBu_3 (4.8 ml, 18.26 mmol) and ADDP (4790 mg, 18.99 mmol) in dry THF (144 ml). The reaction was stirred at room temperature for 24 h. The reaction was monitored by TLC (50% ethyl acetate in pentane). The crude product was purified by flash column chromatography (25-100% ethyl acetate in pentane) to provide compound **10b** as a light yellow sticky solid (2450 mg, 75%). Starting material (727 mg) was recovered after column chromatography. ^{1}H NMR ($CDCl_3$): 7.84 (s, 1H), 7.33 – 7.24 (m, 4H), 5.45 (tt, *J* 7.6, 5.0 Hz, 1H), 3.89 – 3.85 (m, 2H), 3.60 (dd, *J* 16.3, 7.5 Hz, 3H), 3.36 (dd, *J* 16.3, 5.0 Hz, 2H), 1.67 (sext, *J* 7.5 Hz, 2H), 1.50 (s, 9H), 0.88 (t, *J* 7.5, 3H); ^{13}C NMR ($CDCl_3$): 155.3, 154.0, 152.4, 150.5, 142.8, 139.6, 128.4, 127.7, 124.9, 81.4, 55.5, 50.2, 39.8, 28.3, 22.2, 11.4. HRMS *m/z* $[M + H]^+$ calculated for $C_{22}H_{26}ClN_5O_2$: 428.1853. Found: 428.1865.

2-(N-*p*-Cl-Benzyl-N-*tert*-butoxycarbonylamino)-6-chloro-9-(2-indanyl)purine (10c)

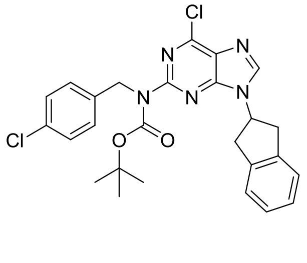

Compound **10c** was synthesised following general procedure B in dry THF at 0 °C for 1.3 h, then at room temperature overnight from **9a** (636 mg, 1.65 mmol), 4-chlorobenzylalcohol (589 mg, 4.13 mmol), PBu_3

(1.1 ml, 4.2 mmol) and ADDP (1040 mg, 4.11 mmol) in dry THF (40 ml). The reaction was monitored by TLC (30% ethyl acetate in pentane). The crude was purified by flash column chromatography (30% ethyl acetate in hexane) to provide **10c** as a white solid (765 mg, 91%). ^{1}H NMR ($CDCl_3$): 7.83 (s, 1H), 7.30 – 7.25 (m, 6H), 7.22 – 7.19 (m, 2H), 5.42 – 5.34 (m, 1H), 5.11 (s, 2H), 3.53 (dd, *J* 16.2, 7.6 Hz, 2H), 3.32 (dd, *J* 16.2, 5.3 Hz, 2H), 1.48 (s, 9H); ^{13}C NMR ($CDCl_3$): 154.7, 153.9, 152.4, 150.6, 143.0, 139.6, 137.1, 132.9, 129.1, 128.6, 128.5, 127.8, 124.9, 82.3, 55.7, 51.0, 39.7, 28.3. HRMS *m/z* $[M + H]^+$ calculated for $C_{26}H_{25}Cl_2N_5O_2$: 510.1463. Found: 510.1439.

9-Benzyl-2-(N-benzyl-*tert*-butoxycarbonylamino)-6-chloro-purine (10d)

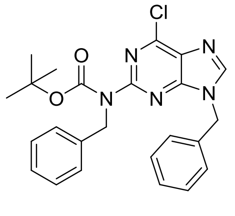

Benzyl alcohol (1.15 ml, 11.1 mmol) was added to a stirred solution of **1** (1.00 g, 3.72 mmol) in dry THF (50 ml) followed by addition of PBu_3 (2.80 ml, 11.2 mmol) under nitrogen at room temperature. The septum was removed and ADDP (2.82 g, 11.2 mmol) was added, the septum with nitrogen inlet was quickly replaced and the reaction mixture was stirred at room temperature. The orange colour faded within 20 min to a pale yellow. Precipitation formed after 1 h. The reaction mixture was stirred at room temperature for 5 h after which time all starting material was consumed as observed by TLC (10% methanol in $CHCl_3$). The precipitate was filtered off and washed with small portions of cold THF (3 x 2 ml). The solvent was removed under reduced pressure, the crude was re-dissolved in ethyl acetate (180 ml), washed with sat. $NaHCO_3$ (aq., 3 x 30 ml), brine (30 ml) and dried over Na_2SO_4. The solvents were removed under reduced pressure and the crude was purified by automated flash column chromatography (0-25% ethyl acetate in pentane) to provide **10d** as a pale yellow solid (1300 mg, 78%). ^{1}H NMR ($CDCl_3$): 7.95 (s, 1H), 7.39 – 7.30 (m, 5H), 7.29 – 7.17 (m, 5H), 5.31 (s, 2H), 5.20 (s, 2H), 1.46 (s, 9H); ^{13}C NMR ($CDCl_3$): 155.4, 154.0, 152.6, 150.7, 144.4, 138.6, 134.8, 129.3, 129.3, 128.9, 128.4, 128.2, 127.7, 127.1, 82.1, 51.6, 47.9, 28.3. HRMS *m/z* $[M + H]^+$ calculated for $C_{24}H_{24}ClN_5O_2$: 450.1697. Found: 450.1707.

2-Benzylamino-6-dimethylamino-9-(2-indanyl)purine (11a)

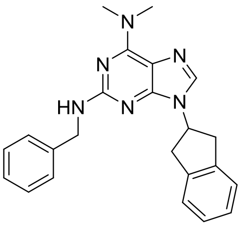

Compound **11a** was synthesised following general procedure C from **9a** (473 mg, 1.0 mmol) as substrate in dry DMF (15 ml). The crude product was purified by automated flash column chromatography (20-30 % ethyl acetate in pentane) to provide **11a** as a colourless oil (362 mg, 95%). ^{1}H NMR ($CDCl_3$): 7.40 – 7.36 (m, 2H), 7.35 (s, 1H), 7.33 – 7.19 (m, 7H), 5.37 – 5.24 (m, 2H), 4.64 (d, *J* 6.0 Hz, 2H), 3.47 (dd, *J* 16.3, 7.7 Hz, 2H), 3.45 (br s, 6H), 3.30 (dd, *J* 16.1, 5.6 Hz, 2H); ^{13}C NMR ($CDCl_3$): 158.7, 155.1, 152.7, 140.6, 140.4, 133.6, 128.2, 127.6, 127.1, 126.7, 124.6, 115.0, 54.1, 46.0, 39.5, 38.1. HRMS *m/z* $[M + H]^+$ calculated for $C_{23}H_{24}N_6$: 385.2141. Found: 385.2123.

6-Dimetylamino-9-(2-indanyl)-2-(1-propylamino)purine (11b)

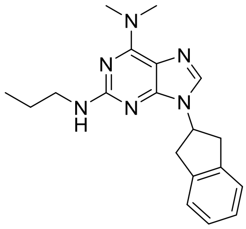

The reaction was run in two batches. **10b** (511 mg, 1.19 mmol) and (200 mg, 0.47 mmol) were dissolved in a dry DMF (14 ml and 12 ml respectively) in two dry microwave vials. The reactions were flushed with nitrogen and then heated in a microwave reactor at 180°C for 60 min. The reactions were monitored by TLC (50% ethyl acetate in pentane). The solvents were removed under reduced pressure, co-evaporating with toluene (x 3). Trace amounts of starting material was still present after purification by automated flash column chromatography (10-30% ethyl acetate in pentane) and (20% ethyl acetate in

pentane). The product could not be separated from the starting material. The obtained mixed fractions from the two batches were pooled, dissolved in dry DMF (15 ml) and heated in the microwave at 180 °C for additional 75 min. TLC (50% ethyl acetate in pentane) showed full consumption of the starting material. The solvent was removed under reduced pressure and the crude product was purified by automated flash column chromatography (15-20% ethyl acetate in pentane) to provide compound **11b** as an colourless oil (318 mg, 57%). ^{1}H NMR ($CDCl_3$): 7.30 (s, 1H), 7.28 – 7.19 (m, 4H), 5.31 (tt, *J* 7.5, 5.4 Hz, 1H), 4.74 (t, *J* 5.9 Hz, 1H), 3.50 (dd, *J* 16.1, 7.5 Hz, 3H), 3.43 (br s, 6H), 3.39 – 3.34 (m, 2H), 3.28 (dd, *J* 16.1, 5.4 Hz, 2H), 1.62 (sext, *J* 7.4 Hz, 2H), 0.96 (t, *J* 7.4 Hz, 3H); ^{13}C NMR ($CDCl_3$): 159.2, 155.2, 152.9, 140.5, 133.4, 127.2, 124.7, 114.8, 54.0, 43.8, 39.8, 38.1, 23.3, 11.7. HRMS *m/z* $[M + H]^+$ calculated for $C_{19}H_{24}N_6$: 337.2140. Found: 337.2154.

6-Dimethylamino-9-(2-indanyl)-2-(*p*-chloro-benzylamino)purine (11c)

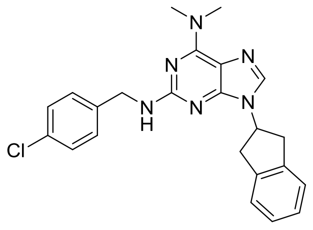

To **10c** (743 mg, 1.46 mmol) was added 5.6 M dimethylamine in ethanol (15 ml, 230 mmol) and the reaction mixture was heated in a microwave reactor at 80°C for 20 min. TLC (30% ethyl acetate in pentane) indicated complete conversion of starting material and the solution was bubbled with nitrogen to remove excess dimethylamine prior to removal of the solvents under reduced pressure. The crude was dissolved in CH_2Cl_2 (10 ml) and TFA (4 ml, 50 mmol) was added. TLC (50% ethyl acetate in pentane) indicated full conversion of starting material after 4 h, the solution was bubbled with nitrogen to remove excess TFA and the solvent was removed under reduced pressure. The crude was dissolved in CH_2Cl_2 (30 ml), water was added (10 ml) and the solution basified by addition of 6 M NaOH (aq.). The phases were separated, the aqueous phase was extracted with CH_2Cl_2 (4 x 40 ml) and the combined organic phases were washed with brine and dried over Na_2SO_4. The solvent was removed under reduced pressure and the crude product purified by flash column chromatography (28% ethyl acetate in pentane) to provide **11c** as an off white solid (579 mg, 95%). ^{1}H NMR ($CDCl_3$): 7.35 (s, 1H), 7.28 – 7.18 (m, 8H), 5.47 (t, *J* 6.1 Hz, 1H), 5.25 (tt, *J* 7.5, 5.8 Hz, 1H), 4.54 (d, *J* 6.1 Hz, 2H), 3.43 (dd, *J* 16.1, 7.5 Hz, 2H), 3.42 (br s, 6H), 3.29 (dd, *J* 16.0, 5.8 Hz, 2H); ^{13}C NMR ($CDCl_3$): 158.5, 155.0, 152.6, 140.3, 139.2, 133.7, 132.1, 128.7, 128.2, 127.0, 124.5, 115.1, 54.2, 45.2, 39.3, 38.1. HRMS *m/z* $[M + H]^+$ calculated for $C_{23}H_{23}ClN_6$: 419.1751. Found: 419.1763.

2-Benzylamino-6-(ethoxy-carbonyl-methylamino)-9-(2-indanyl)purine (11d)

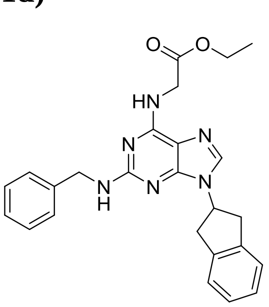

Glycine ethyl ester hydrochloride (191 mg, 1.37 mmol) and triethylamine (0.32 ml, 2.3 mmol) were added to **10a** (218 mg, 0.458 mmol) in ethanol (18 ml). The reaction mixture was heated at 100°C overnight. Full consumption of starting material was confirmed by TLC (60% ethyl acetate in pentane). The solvent was removed under reduced pressure, the crude product was dissolved in CH_2Cl_2 (7 ml) and TFA (3 ml, 40 mmol) was added. The reaction mixture was stirred at room temperature for 4 h. Full consumption of starting material was confirmed by TLC (60% ethyl acetate in pentane), the reaction mixture was cooled on ice, basified by addition of 3 M NaOH (aq.) and diluted with distilled water (20 ml) and CH_2Cl_2 (35 ml). The aqueous phase was extracted with CH_2Cl_2 (4 x 35 ml) and the combined organic phases were washed with brine and dried over Na_2SO_4. The solvent was removed under reduced pressure and purification by automated flash column chromatography (2.5% methanol in CH_2Cl_2) provided **11d** as a white solid (184 mg, 91%). ^{1}H NMR ($CDCl_3$): 7.37 – 7.20 (m, 9H), 6.72 (br s, 1H), 5.51 (br s, 1H),

5.25 (tt, *J* 7.5, 5.6 Hz, 1H), 4.59 (d, *J* 5.9 Hz, 2H), 4.29 (br s, 2H), 4.16 (q, *J* 7.1 Hz, 2H), 3.46 (dd, *J* 16.1, 7.6 Hz, 2H), 3.30 (dd, *J* 16.1, 5.6 Hz, 2H), 1.24 (t, *J* 7.1 Hz, 3H); ^{13}C NMR ($CDCl_3$): 170.7, 159.2, 154.6, 151.6, 140.4, 140.3, 135.2, 128.3, 127.6, 127.1, 126.7, 124.6, 114.6, 61.0, 54.3, 45.9, 42.6, 39.5, 14.2. HRMS *m/z* $[M + H]^+$ calculated for $C_{25}H_{26}N_6O_2$: 443.2195. Found: 443.2210.

6-(Ethoxy-carbonyl-methylamino)-9-(2-indanyl)-2-(1-propylamino)purine (11e)

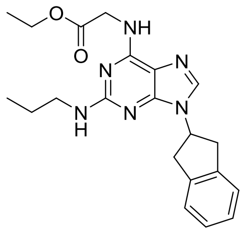

The reaction was run in two batches, which was then pooled prior to work-up and purification. **10b** (250 mg, 1.17 mmol) was suspended in ethanol (15 ml, 99.7%) in two oven-dried microwave vials. Glycine ethyl ester hydrochloride (245 mg, 1.76 mmol) and triethyl amine (0.41 ml, 2.94 mmol) were added and the reactions were heated to 100ºC, whereupon the starting materials dissolved. The reaction was left at 100ºC overnight. TLC (5% methanol in CH_2Cl_2) indicated full consumption of the starting material. The two batches were pooled and the solvent was removed under reduced pressure. The crude product was dissolved in CH_2Cl_2 (13 ml) and TFA (4 ml) was added. The reaction mixture was stirred at room temperature for 3 h after which full deprotection was confirmed by LCMS. The reaction mixture was cooled on ice and diluted with distilled water (20 ml) and then basified with 6 M NaOH (aq.). The aqueous phase was extracted with CH_2Cl_2 (4 x 20 ml), the combined organic phases were washed with brine and dried over $MgSO_4$, filtered and the solvent was removed under reduced pressure. The crude product was purified by automated flash column chromatography (5% methanol in CH_2Cl_2) to provide compound **11e** as a light yellow oil/foam (451 mg, 98%). ^{1}H NMR ($CDCl_3$): 7.31 (s, 1H), 7.30 – 7.18 (m, 4H), 6.25 (br s, 1H), 5.24 – 5.31 (m, 1H), 4.86 (t, *J* 5.9 Hz, 1H), 4.31 (d, *J* 5.4 Hz, 2H), 4.20 (q, *J* 7.1 Hz, 2H), 3.50 (dd, *J* 16.2, 7.6 Hz, 2H), 3.38 – 3.25 (m, 4H), 1.59 (sext, *J* 7.3 Hz, 2H), 1.26 (t, *J* 7.1 Hz, 3H), 0.95 (t, *J* 7.4 Hz, 3H); ^{13}C NMR ($CDCl_3$): 170.7, 159.6, 154.5, 151.8, 140.5, 135.1, 127.2, 124.7, 114.4, 61.2, 54.2, 43.8, 42.7, 39.8, 23.2, 14.3, 11.7. HRMS *m/z* $[M + H]^+$ calculated for $C_{21}H_{25}N_6O_2$: 395.2195. Found: 395.2195.

9-Benzyl-2-benzylamino-6-(ethoxy-carbonyl-methylamino)purine (11f)

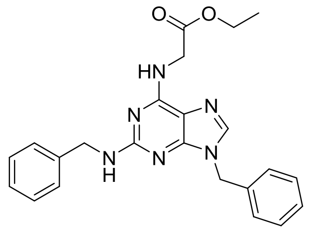

K_2CO_3 (350 mg, 2.53 mmol) and glycine ethyl ester hydrochloride (353 mg, 2.53 mmol) were added to a solution of **10d** (380 mg, 0.845 mmol) in acetonitrile (19 ml, dried over molecular sieves). The solution was heated at 70 °C for 3 days after which full consumption of starting material was confirmed by TLC (50% ethyl acetate in pentane). The reaction mixture was diluted with water (20 ml) and extracted with CH_2Cl_2 (5 x 20 ml). The combined organic phases were washed with brine and dried over Na_2SO_4. The solvents were removed under reduced pressure, the crude was re-dissolved in CH_2Cl_2 (6 ml) and TFA (2 ml, 26 mmol) was added. The reaction mixture was stirred at room temperature for 2 h. Full conversion was confirmed by TLC (50% ethyl acetate in pentane). The reaction mixture was diluted with water (10 ml), basified (pH 12) with 6 M NaOH (aq.) and the aqueous phase was extracted with CH_2Cl_2 (4 x 20 ml). The combined organic phases were washed with brine and dried over Na_2SO_4. The solvent was removed under reduced pressure and purification by automated flash column chromatography (65-70% ethyl acetate in pentane) provided **11f** as a white solid (217 mg, 62%). ^{1}H NMR ($CDCl_3$): 7.41 (s, 1H, H8), 7.36 – 7.19 (m, 10H), 6.15 (br s, 1H), 5.25 (br s, 1H), 5.16 (s, 2H), 4.61 (d, *J* 5.9 Hz, 2H), 4.30 (br s, 2H), 4.17 (q, *J* 7.4 Hz, 2H), 1.24 (t, *J* 7.4 Hz, 3H); ^{13}C NMR ($CDCl_3$): 170.6, 159.6, 154.6, 152.0, 140.4, 137.2, 136.5, 128.9, 128.5, 128.1, 128.0, 127.7,

127.0, 114.5, 61.3, 46.7, 46.0, 14.3. HRMS *m/z* $[M + H]^+$ calculated for $C_{23}H_{23}N_6O_2$: 417.2039. Found: 417.2044.

9-Benzyl-2-benzylamino-6-(methoxy-carbonyl-methyl-thio)purine (11g)

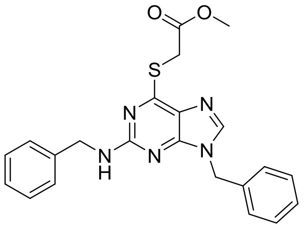

A solution of **10d** (452 mg, 26.0 mmol) and methyl thioglycolate (0.275 ml, 3.01 mmol) in dry toluene (13 ml) were added to NaH (125 mg, 3.13 mmol, 60% in mineral oil) in dry toluene (15 ml). The flask was fitted with a reflux condenser and heated at 70°C overnight. The reaction was monitored by TLC (40% ethyl acetate in pentane). The reaction was quenched with sat. $NaHCO_3$ (aq., 5 ml) and diluted with water (20 ml). The aqueous phase was extracted with CH_2Cl_2 (6 x 15 ml) and the combined organic phases were washed with brine and dried over Na_2SO_4. The solvents were removed under reduced pressure, the crude was re-dissolved in CH_2Cl_2 (6 ml) and TFA (2 ml, 26.0 mmol) was added. The reaction mixture was stirred at room temperature for 5 h after which full consumption of starting material was confirmed by LC-MS. The reaction mixture was diluted with water (15 ml) and CH_2Cl_2 (10 ml) and basified by addition of 6 M NaOH (aq.). The aqueous phase was extracted with CH_2Cl_2 (6 x 15 ml) and the combined organic phases were washed with brine and dried over Na_2SO_4. The solvent was removed under reduced pressure and purification by automated flash column chromatography (50-65% ethyl acetate in pentane) provided **11g** as a colourless oil (252 mg, 60%). ^1H NMR ($CDCl_3$): 7.58 (s, 1H), 7.36 – 7.15 (m, 10H), 5.64 (t, *J* 6.0 Hz, 1H), 5.14 (s, 2H), 4.64 (d, *J* 6.0 Hz, 2H), 4.00 (s, 2H), 3.64 (s, 3H); ^{13}C NMR ($CDCl_3$): 169.7, 158.7, 158.6, 151.1, 139.7, 139.6, 135.8, 128.8, 128.4, 128.1, 127.7, 127.3, 126.9, 124.8, 52.5, 46.6, 45.7, 30.6. HRMS *m/z* $[M + H]^+$ calculated for $C_{22}H_{21}N_5O_2S$: 420.1494. Found: 420.1495.

2-Benzylamino-8-bromo-6-dimethylamino-9-(2-indanyl)purine (12a)

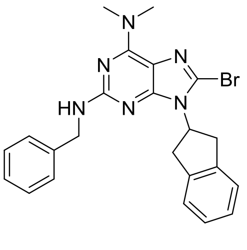

Compound **12a** was synthesised following general procedure D from **11a** (124 mg, 0.323 mmol) $PyBr_3$ (157 mg, 0.491 mmol) in dry CH_2Cl_2 (12 ml). The reaction was stirred overnight at room temperature. Full consumption of starting material was confirmed by TLC (50% ethyl acetate in pentane). Deviation from general procedure, the reaction was quenched with 10% $Na_2S_2O_3$ (20 ml), diluted with water (20 ml) and CH_2Cl_2 (20 ml). The reaction was basified via addition of 6 M NaOH (aq.) and the aqueous phase was extracted with CH_2Cl_2 (4 x 40 ml). The crude product was purified by automated flash column chromatography (7% ethyl acetate in pentane) to provide **12a** as a white solid (125 mg, 84%). ^1H NMR ($CDCl_3$): 7.47 – 6.99 (m, 9H), 5.36 (q, *J* 9.1 Hz, 1H), 5.15 (t, *J* 6.1 Hz, 1H), 4.41 (d, *J* 6.1 Hz, 2H), 3.97 (dd, *J* 15.4, 9.4 Hz, 2H), 3.41 (br s, 6H), 3.22 (dd, *J* 15.4, 8.9 Hz, 2H); ^{13}C NMR ($CDCl_3$): 157.9, 154.13, 154.10, 140.9, 140.4, 128.3, 127.6, 126.8, 126.7, 124.5, 120.5, 116.0, 56.5, 45.9, 38.3, 36.6. HRMS *m/z* $[M + H]^+$ calculated for $C_{23}H_{23}BrN_6$: 463.1246. Found: 463.1230.

8-Bromo-6-dimetylamino-9-(2-indanyl)-2-(1-propylamino)purine (12b)

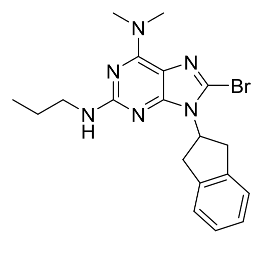

Compound **12b** was synthesised following general procedure D from **11b** (303 mg, 0.90 mmol) and $PyrBr_3$ (355 mg, 1.11 mmol) in dry CH_2Cl_2 (16 ml). The reaction was stirred at room temperature overnight. Full consumption of starting material was confirmed by TLC (20% ethyl acetate in pentane). The crude product was purified by automated flash column chromatography (10% ethyl acetate in pentane) to provide compound **12b** as

a colourless oil/foam (326 mg, 87%). ^{1}H NMR ($CDCl_3$): 7.31 – 7.22 (m, 4H), 5.43 (quin, *J* 9.1 Hz, 1H), 4.77 (t, *J* 5.6 Hz, 1H), 4.04 (dd, *J* 15.5, 9.2 Hz, 2H), 3.45 (br s, 6H), 3.30 (dd, *J* 15.5, 9.0 Hz, 2H), 3.21 (q, *J* 6.7 Hz, 2H), 1.54 (sext, *J* 7.4 Hz, 2H), 0.89 (t, *J* 7.4 Hz, 3H); ^{13}C NMR ($CDCl_3$): 158.2, 154.2, 154.1, 140.9, 126.7, 124.4, 120.1, 115.6, 56.2, 43.6, 38.2, 36.8, 23.1, 11.6. HRMS *m/z* $[M + H]^+$ calculated for $C_{19}H_{23}BrN_6$: 415.1246. Found: 415.1259.

8-Bromo-6-dimethylamino-9-(2-indanyl)-2-(*p*-chloro-benzylamino)purine (12c)

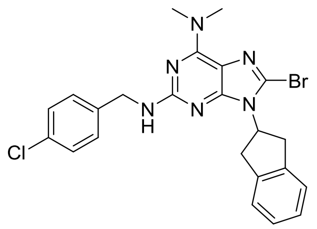

Compound **12c** was synthesised following general procedure D from **11c** (567 mg, 1.36 mmol) and $PyrBr_3$ (650 mg, 2.03 mmol) in dry CH_2Cl_2 (50 ml). The reaction was stirred overnight at room temperature. TLC (22% ethyl acetate in pentane) indicated full consumption of the starting material.). The crude was purified by automated flash column chromatography (7% ethyl acetate in pentane) to provide **12c** as a white solid (580 mg, 86%). ^{1}H NMR ($CDCl_3$): 7.25 – 7.18 (m, 4H), 7.15 – 7.05 (m, 4H), 5.31 (quin, *J* 9.2 Hz, 1H), 5.10 (br s, 1H), 4.33 (d, *J* 6.1 Hz, 2H), 3.87 (dd, *J* 12.2, 4.2 Hz, 2H), 3.38 (br s, 6H), 3.17 (dd, *J* 15.4, 8.8 Hz, 2H); ^{13}C NMR ($CDCl_3$): 157.7, 154.2, 154.0, 140.9, 139.1, 132.3, 128.8, 128.4, 126.9, 124.5, 120.8, 116.2, 56.7, 45.3, 38.4, 36.6. HRMS *m/z* $[M + H]^+$ calculated for $C_{23}H_{22}BrClN_6$: 497.0856. Found: 497.0863.

2-Benzylamino-8-bromo-6-(ethoxy-carbonyl-methylamino)-9-(2-indanyl)purine (12d)

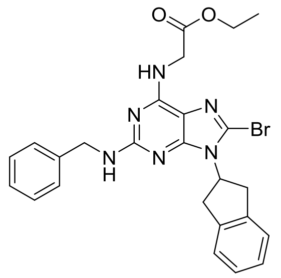

Compound **12d** was synthesised following general procedure D from **11d** (206 mg, 0.466 mmol) and $PyrBr_3$ (233 mg, 0.739 mmol) in CH_2Cl_2 (20 ml). Full consumption of starting material was confirmed by TLC (50% ethyl acetate in pentane) after 2 h. Purification by flash column chromatography (22% ethyl acetate in pentane) provided **12d** as a white solid (177 mg, 73%). ^{1}H NMR ($CDCl_3$): 7.27 – 7.14 (m, 9H), 6.25 (br s, 1H), 5.38 (br s, 1H), 5.33 (quin, *J* 9.1, Hz, 1H), 4.38 (d, *J* 5.2 Hz, 2H), 4.30 (br s, 2H), 4.21 (q, *J* 7.1 Hz, 2H), 3.94 (dd, *J* 15.4, 9.4 Hz, 2H), 3.22 (dd, *J* 15.4, 8.8 Hz, 2H), 1.28 (t, *J* 7.1 Hz, 3H); ^{13}C NMR ($CDCl_3$): 170.5, 158.3, 153.5, 152.8, 140.7, 140.1, 128.2, 127.5, 126.8, 126.7, 124.4, 122.4, 115.4, 61.2, 56.8, 45.8, 42.6, 36.7, 14.3. HRMS *m/z* $[M + H]^+$ calculated for $C_{25}H_{25}BrN_6O_2$: 521.1300. Found: 521.1316.

8-Bromo-6-(ethoxy-carbonyl-methylamino)-9-(2-indanyl)-2-(1-propylamino)purine (12e)

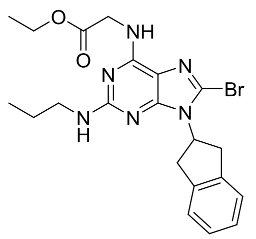

Compound **12e** was synthesised following general procedure D from **11e** (418 mg, 1.06 mmol) and $PyrBr_3$ (410 mg, 1.21 mmol) in dry CH_2Cl_2 (17 ml). The reaction was stirred at room temperature for 5 h whereupon full consumption of starting material was observed by LCMS. The crude product was purified by automated flash column chromatography (20-30% ethyl acetate in pentane) to provide **12e** as a colourless oil (479 mg, 96%). ^{1}H NMR ($CDCl_3$): 7.26 – 7.19 (m, 4H), 5.92 (t, *J* 5.3 Hz, 1H), 5.34 (quin, *J* 9.1 Hz, 1H), 4.77 (t, *J* 5.9 Hz, 1H), 4.30 (d, *J* 5.5 Hz, 2H), 4.23 (q, *J* 7.1 Hz, 2H), 3.96 (dd, *J* 15.5, 9.2 Hz, 2H), 3.26 (dd, *J* 15.4, 9.0 Hz, 2H), 3.14 (q, *J* 6.6 Hz, 2H), 1.48 (sext, *J* 7.4 Hz, 2H), 1.29 (t, *J* 7.1 Hz, 3H), 0.84 (t, *J* 7.4 Hz, 3H); ^{13}C NMR ($CDCl_3$): 170.5, 158.8, 153.4, 153.1, 140.8, 126.9, 124.5, 122.2, 115.3, 61.4, 56.6, 43.6, 42.6, 37.0, 23.0, 14.4, 11.6. HRMS *m/z* $[M + H]^+$ calculated for $C_{21}H_{25}BrN_6O_2$: 473.1300. Found: 473.1331.

9-benzyl-2-benzylamino-8-bromo-6-(ethoxy-carbonyl-methylamino)purine (12f)

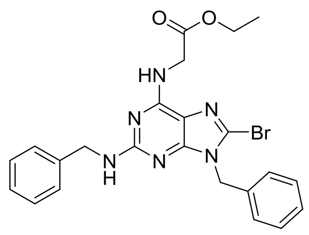

Compound **12f** was synthesised following general procedure D from **11f** (220 mg, 0.528 mmol) and $PyrBr_3$ (254 mg, 0.794 mmol) in CH_2Cl_2 (22 ml). The reaction was stirred at room temperature overnight and full conversion of starting material was confirmed by TLC (30% ethyl acetate in pentane). The aqueous phase was extracted with CH_2Cl_2 (6 x 15 ml). Purification by automated flash column chromatography (25-35% ethyl acetate in pentane) provided **12f** as a white solid (212 mg, 81%). ^1H NMR ($CDCl_3$): 7.37 – 7.21 (m, 10H), 6.25 (t, *J* 5.4 Hz, 1H), 5.49 (s, 1H), 5.19 (s, 2H), 4.62 (d, *J* 6.0 Hz, 2H), 4.30 (s, 2H), 4.19 (q, *J* 7.1 Hz, 2H), 1.26 (t, *J* 7.1 Hz, 3H); ^{13}C NMR ($CDCl_3$): 170.4, 159.3, 153.4, 153.1, 140.1, 135.9, 128.7, 128.4, 128.0, 127.9, 127.5, 126.9, 122.2, 114.8, 61.3, 46.9, 45.8, 42.7, 14.2. HRMS *m/z* $[M + H]^+$ calculated for $C_{23}H_{23}BrN_6O_2$: 495.1144. Found: 495.1157.

9-Benzyl-2-benzylamino-8-bromo-6-(methoxy-carbonyl-methyl-thio)purine (12g)

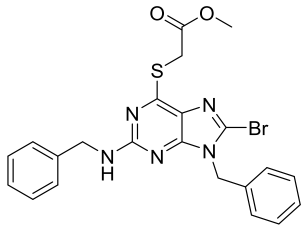

Compound **12g** was synthesised following general procedure D from **11g** (234 mg, 0.558 mmol) and $PyrBr_3$ (276 mg, 0.862 mmol) in CH_2Cl_2 (23 ml). The reaction was monitored by TLC (35% ethyl acetate in pentane) and quenched after 15 h. The aqueous phase was extracted with CH_2Cl_2 (7 x 15 ml). Purification by flash column chromatography (25-35% ethyl acetate in pentane) provided **12g** as a colourless sticky solid (96 mg, 35%). ^1H NMR ($CDCl_3$): 7.34 – 7.22 (m, 10H), 5.48 (br s, 1H), 5.21 (s, 2H), 4.63 (d, *J* 6.0 Hz, 2H), 3.99 (s, 2H), 3.67 (s, 3H); ^{13}C NMR ($CDCl_3$): 169.6, 158.4, 157.9, 152.2, 139.4, 135.3, 128.8, 128.6, 128.2, 128.0, 127.5, 127.2, 126.3, 125.3, 52.7, 47.2, 45.9, 30.8. HRMS *m/z* $[M + H]^+$ calculated for $C_{22}H_{20}BrN_5O_2S$: 498.0599. Found: 498.0602.

6-Amino-9-benzyl-2-benzylamino-8-bromopurine (12h)

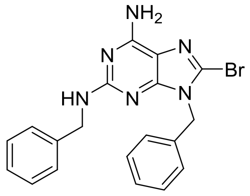

A solution of NH_4OH (aq. 28%, 7.5 ml) was added to a solution of **10d** (703 mg, 1.56 mmol) in 1,4-dioxane (7.5 ml) in a 10-20 ml microwave vial. The vial was capped, flushed with nitrogen and heated in a microwave reactor at 100°C for 1 h. Excess NH_3 was bubbled off with nitrogen and the solvents were removed by evaporation on high vacuum. The white solid was taken up in CH_2Cl_2 (4 ml), TFA (4 ml) was added and the reaction mixture was stirred at room temperature under nitrogen. Full consumption of the starting material and one new major spot was observed by TLC (10% methanol in $CHCl_3$) after 1 h. 3 M NaOH (aq.) was added to basify, CH_2Cl_2 (200 ml) was added (the mixture was stirred until the white precipitation that had formed on basification was dissolved) followed by water (50 ml) and the phases were separated. The aqueous phase was extracted with CH_2Cl_2, the organic phases pooled, dried over Na_2SO_4 and the solvents were removed under reduced pressure. The white crude was suspended in CH_2Cl_2 (40 ml) and transferred to a three necked round-bottomed flask equipped with a nitrogen inlet. The mixture was heated at 40 °C to dissolve the material and $PyrBr_3$ (999 mg, 3.12 mmol) was added. The reaction was heated at 40 °C for 3 h and then at room temperature for 1 h after which TLC indicated full consumption of starting material (10% methanol in $CHCl_3$). The reaction was quenched with $Na_2S_2O_3$ (10% aq.), basified with 0.5 M NaOH (aq.) to pH 10-11 and diluted with $CHCl_3$ (300 ml). Water was added and the phases were separated, the aqueous phase extracted with $CHCl_3$ (2 x 50 ml), the organic phases were collected and the solvents were removed under reduced pressure. The

crude was purified by flash column chromatography (2% methanol in $CHCl_3$) to provide **12h** as a white solid (329 mg, 51% over 3 steps). ^{1}H NMR ($CDCl_3$): 7.38 – 7.21 (m, 10H), 5.35 (br s, 2H), 5.32 (t, *J* 5.9 Hz, 1H), 5.21 (s, 2H), 4.63 (d, *J* 6.0 Hz, 2H); ^{13}C NMR ($CDCl_3$): 159.4, 154.4, 153.6, 140.0, 135.8, 128.8, 128.6, 128.2, 128.1, 127.6, 127.1, 123.0, 114.6, 47.2, 45.9. HRMS *m/z* $[M + H]^+$ calculated for $C_{19}H_{17}BrN_6$: 409.0776. Found: 409.0779.

6-Amino-8-bromo-2-(1-propylamino)purine (12i)

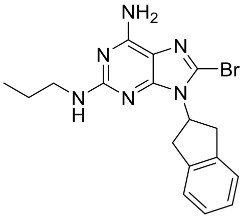

A solution of NH_4OH (aq. 28%, 7 ml) was added to a solution of **10b** in 1,4-dioxane (7 ml) in a 10-20 ml microwave vial. The vial was capped, flushed with nitrogen and heated in a microwave reactor at 100ºC for 1 h. Excess NH_3 was bubbled off with nitrogen and solvents were removed under reduced pressure. The resulting white solid was taken up in CH_2Cl_2 (4 ml), TFA (4 ml) was added and the reaction mixture was stirred at room temperature under nitrogen for 1 h. Full consumption of starting material was observed by TLC (10% methanol in $CHCl_3$). The solution was concentrated by passing a stream of nitrogen over the solution. Water (50 ml) was added and the suspension was basified by addition of 3 M NaOH (aq.). The aqueous phase was extracted with CH_2Cl_2 (3 x 100 ml), the organic phases were pooled, dried over Na_2SO_4 and the solvents were removed under reduced pressure. The white crude was dissolved in CH_2Cl_2 (30 ml) and transferred to a three necked round bottom flask equipped with a nitrogen inlet. $PyrBr_3$ (747 mg, 2.34 mmol) was added, the reaction mixture was stirred at room temperature for 15 min and then at 40 ºC for 26 h (precipitation formed after 3 min at room temperature and dissolved when heated). The reaction mixture was cooled to room temperature, quenched with 10% $Na_2S_2O_3$ (aq.), basified with 0.5 M NaOH (aq.) to pH 10-11 and diluted with CH_2Cl_2 (150 ml) and water (50 ml). The phases were separated and the aqueous phase was extracted with CH_2Cl_2 (2 x 50 ml). The pooled organic phases were washed with brine, dried over Na_2SO_4 and the solvents were removed under reduced pressure. Starting material was still present after purification by automated flash column chromatography (2% methanol in $CHCl_3$). The obtained mixed fractions were pooled and the solvents were removed under reduced pressure. The obtained solid was re-dissolved in dry CH_2Cl_2 (30 ml), $PyrBr_3$ (450 mg, 1.41 mmol) was added and the reaction mixture was stirred at room temperature for 19 h after which one major spot was observed on TLC (10% methanol in $CHCl_3$). The reaction was quenched with 10% $Na_2S_2O_3$ (aq.), basified with 0.5 M NaOH (aq.) to pH 9-10 and diluted with CH_2Cl_2 (150 ml) and water (50 ml). The phases were separated and the aqueous phase was extracted with CH_2Cl_2 (2 x 50 ml). The pooled organic phases were washed with brine, dried over Na_2SO_4 and the solvents were removed under reduced pressure. Purification by flash column chromatography (2% methanol in $CHCl_3$) provided **12i** as an off white solid (328 mg, 73% over three steps). ^{1}H NMR ($CDCl_3$): 7.26 – 7.18 (m, 4H), 5.37 (quin, *J* 9.0 Hz, 1H), 5.30 (br s, 2H), 4.78 (t, *J* 5.5 Hz, 1H), 3.95 (dd, *J* 15.5, 9.0 Hz, 2H), 3.29 (dd, *J* 15.5, 8.9 Hz, 2H), 3.12 (q, *J* 7.1 Hz, 2H), 1.47 (sext, *J* 7.3 Hz, 2H), 0.83 (t, *J* 7.4 Hz, 3H); ^{13}C NMR ($CDCl_3$): 158.9, 154.5, 153.5, 140.8, 126.9, 124.5, 122.9, 114.9, 56.7, 43.6, 37.1, 23.0, 11.5. HRMS *m/z* $[M + H]^+$ calculated for $C_{17}H_{19}BrN_6$: 387.0933. Found: 387.0920.

2-Benzylamino-6-dimethylamino-8-ethynyl-9-(2-indanyl)purine (13a)

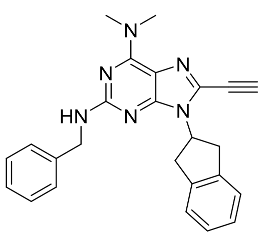

Amberlite IRA-67 (232 mg, 1.30 mmol), CuI (10 mg, 0.053 mmol) and $Pd(PPh_3)_2Cl_2$ (7.5 mg, 0.011 mmol) were added to a solution of **12a** (117 mg, 0.253 mmol) in THF (2 ml). The vial was capped, the reaction mixture was bubbled with nitrogen again and ethynyltrimethylsilane (0.11 ml, 0.80

mmol) was added. The vial was heated at 115°C for 50 min. TLC (35% ethyl acetate in pentane) showed only trace amounts of starting material and the reaction mixture was filtered through a short plug of silica and eluted with THF. The solvent volume was adjusted to 5 ml and polymer supported fluoride was added (134 mg). TLC (35% ethyl acetate in pentane) the following morning indicated full conversion of starting material. The polymers were filtered off and washed with a few ml of CH_2Cl_2 and THF, the solvents were removed under reduced pressure. The crude product was purified by flash column chromatography (12-20% ethyl acetate in pentane) to provide **13a** as a brown solid (72 mg, 70%). ^{1}H NMR ($CDCl_3$): 7.26 – 7.20 (m, 9H), 5.51 (quin, *J* 9.1 Hz, 1H), 5.19 (br t, *J* 6.1 Hz, 1H), 4.47 (d, *J* 6.1 Hz, 2H), 3.93 (dd, *J* 15.4, 9.4 Hz, 2H), 3.45 (br s, 6H), 3.35 (s, 1H), 3.27 (dd, *J* 15.4, 8.9 Hz, 2H); ^{13}C NMR ($CDCl_3$): 159.0, 155.0, 152.8, 141.0, 140.4, 128.31 (two carbons), 127.6, 126.77 (two carbons), 124.5, 115.6, 82.8, 74.6, 55.2, 45.9, 38.4, 37.3. HRMS *m/z* $[M + H]^+$ calculated for $C_{25}H_{24}N_6$: 409.2140. Found: 409.2137.

6-Dimetylamino-8-ethynyl-9-(2-indanyl)-2-(1-propylamino)purine (13b)

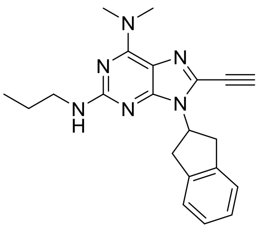

$PdCl_2(PPh_3)_2$ (26 mg, 0.037 mmol), CuI (28 mg, 0.15 mmol) and amberlite IRA-67 (646 mg, 3.62 mmol) was added to a solution of **12b** (299 mg, 0.72 mmol) in dry THF (5 ml) in an oven-dried microwave vial. The vial was capped, nitrogen gas was bubbled through the reaction mixture and ethynyltrimethylsilane (400 µl, 2.89 mmol) was added. The yellow reaction mixture was heated at 115°C for 1 h. The obtained dark reaction mixture was filtered through a short plug of Celite®. The solvent volume was reduced to 20 ml by evaporation and polymer supported fluoride (378 mg) was added. The reaction mixture was stirred at room temperature overnight. The polymer was filtered off, washed with THF and CH_2Cl_2 and the solvents were removed under reduced pressure. The crude product was purified by flash column chromatography (10% ethyl acetate in pentane) to provide **13b** as a yellow oil (188 mg, 72%). ^{1}H NMR ($CDCl_3$): 7.28 – 7.16 (m, 4H), 5.51 (quin, *J* 9.1 Hz, 1H), 4.72 (br s, 1H), 3.92 (dd, *J* 15.5, 9.3 Hz, 2H), 3.45 (br s, 6H), 3.33 (s, 1H), 3.29 (dd, *J* 15.5, 9.1 Hz, 2H), 3.22 (q, *J* 6.6 Hz, 3H), 1.53 (sext, *J* 7.4 Hz, 3H), 0.88 (t, *J* 7.4 Hz, 3H); ^{13}C NMR ($CDCl_3$): 159.4, 155.0, 153.0, 141.1, 128.0, 126.7, 124.4, 115.3, 82.8, 74.7, 54.9, 43.6, 38.4, 37.4, 23.2, 11.6. HRMS *m/z* $[M + H]^+$ calculated for $C_{21}H_{24}N_6$: 361.2140. Found: 361.2122.

6-Dimethylamino-8-ethynyl-9-(2-indanyl)-2-(*p*-chloro-benzylamino)purine (13c)

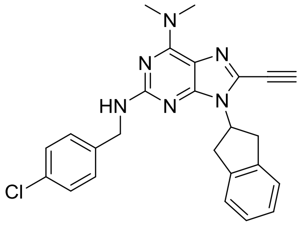

Amberlite IRA-67 (475 mg, 2.66 mmol), CuI (20 mg, 0.11 mmol) and $Pd(PPh_3)_2Cl_2$ (16 mg, 0.023 mmol) were added to a solution of **12c** (262 mg, 0.526 mmol) in dry THF (4.5 ml). The vial was capped, the reaction mixture was bubbled with nitrogen again and ethynyltrimethylsilane (0.22 ml, 1.6 mmol) was added. The reaction mixture was heated in a microwave reactor at 110°C for 50 min. TLC (20% ethyl acetate in pentane) indicated full conversion of starting material. The reaction mixture was filtered through a short plug of silica and eluted with THF. The solvent volume was adjusted to 5 ml, polymer supported fluoride (282 mg) was added and the reaction mixture was stirred at room temperature overnight. Full consumption of starting material was confirmed by LCMS. The polymer was filtered off and washed with CH_2Cl_2 and THF. The solvents were removed under reduced pressure and the crude product was purified by flash column chromatography (91% ethyl acetate in pentane) to provide **13c** as a brown solid (160 mg, 69%). ^{1}H NMR ($CDCl_3$): 7.25 – 7.18 (m, 4H), 7.17 – 7.08 (m, 4H), 5.47 (quin, *J* 9.1 Hz,

1H), 5.21 (t, *J* 5.8 Hz, 1H), 4.38 (d, *J* 5.8 Hz, 2H), 3.86 (dd, *J* 15.4, 9.5 Hz, 2H), 3.43 (br s, 6H), 3.34 (s, 1H) 3.23 (dd, *J* 15.4, 8.8 Hz, 2H); ^{13}C NMR ($CDCl_3$): 158.8, 155.1, 152.7, 141.0, 139.1, 132.3, 128.8, 128.5, 128.4, 126.8, 124.5, 115.7, 82.7, 74.4, 55.4, 45.3, 38.5, 37.2. HRMS *m/z* $[M + H]^+$ calculated for $C_{25}H_{23}ClN_6$: 443.1751. Found: 443.1762.

2-Benzylamino-6-(ethoxy-carbonyl-methylamino)-8-ethynyl-9-(2-indanyl)purine (13d)

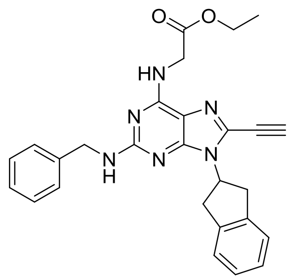

Amberlite IRA-67 (980 mg, 5.49 mmol), CuI (42 mg, 0.22 mmol) and $PdCl_2(PPh_3)_2$ (31 mg, 0.044 mmol) were added to a solution of **12d** (575 mg, 1.10 mmol) in dry THF (12 ml). The vial was capped, nitrogen was bubbled through the reaction mixture and ethynyltrimethylsilane (0.46 ml, 3.3 mmol) was added. The reaction mixture was heated at 115°C for 50 min. Full consumption of starting material was confirmed by TLC (50% ethyl acetate in pentane). The reaction mixture was filtered through a short plug of silica and eluted with THF. The solvent volume was adjusted to 10 ml, polymer supported fluoride (557 mg) was added and the reaction mixture was stirred at room temperature overnight. Full deprotection was confirmed by TLC (50% ethyl acetate in pentane), the polymer was filtered off and washed with CH_2Cl_2 and THF. The solvents were removed under reduced pressure and purification by automated flash column chromatography (22-25% ethyl acetate in pentane) provided **13d** as a brown solid (266 mg, 52%). ^{1}H NMR ($CDCl_3$): 7.26 – 7.16 (m, 9H), 6.46 (t, *J* 5.2 Hz, 1H), 5.47 (quin, *J* 9.2 Hz, 1H), 5.39 (br s, 1H) 4.43 (d, *J* 5.0 Hz, 2H), 4.30 (broad s, 2H), 4.20 (q, *J* 7.1 Hz), 3.90 (dd, *J* 15.5, 9.4 Hz, 2H), 3.36 (s, 1H), 3.27 (dd, *J* 15.5, 8.7 Hz, 2H), 1.26 (t, *J* 7.1 Hz, 3H); ^{13}C NMR ($CDCl_3$): 170.4, 159.4, 154.5, 151.4, 140.8, 140.1, 129.9, 128.2, 127.6, 126.78, 126.78, 124.4, 115.0, 83.0, 74.1, 61.2, 55.6, 45.8, 42.6, 37.3, 14.2. HRMS *m/z* $[M + H]^+$ calculated for $C_{27}H_{26}N_6O_2$: 467.2195. Found: 467.2216.

6-(Ethoxy-carbonyl-methylamino)-8-ethynyl-9-(2-indanyl)-2-(1-propylamino)purine (13e)

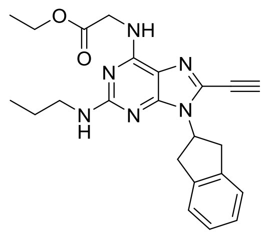

$PdCl_2(PPh_3)_2$ (28 mg, 0.040 mmol), CuI (31 mg, 0.16 mmol), amberlite IRA-67 (718 mg, 4.02 mmol) were added to a solution of **12e** (380 mg, 0.803 mmol) in dry THF (12 ml). The vial was capped, nitrogen was bubbled through the reaction mixture and ethynyltrimethylsilane (450 µl, 3.25 mmol) was added. The yellow reaction mixture was heated in a microwave reactor at 110 °C for 50 min. Full consumption of **12e** was confirmed by TLC (2% methanol in $CHCl_3$). The reaction mixture was filtered through a short plug of silica which was eluted with THF, the solution was concentrated and the volume was adjusted to 8 ml. Polymer supported fluoride (422 mg) was added and the reaction mixture was stirred under nitrogen at room temperature for 19 h. The polymer was filtered off, washed with THF and CH_2Cl_2 and the solvents were removed under reduced pressure. Purification by flash column chromatography (first column eluted with 2% methanol in CH_2Cl_2 and second column with 33% ethyl acetate in pentane) provided **13e** as a yellow solid (200 mg, 60%). ^{1}H NMR ($CDCl_3$): 7.26 – 7.17 (m, 4H), 6.11 (s, 1H), 5.47 (quin, *J* 9.0 Hz, 1H), 4.84 (t, *J* 5.8 Hz, 1H), 4.31 (br s, 2H), 4.23 (q, *J* 7.1 Hz, 2H), 3.91 (dd, *J* 15.5, 9.3 Hz, 2H), 3.35 (s, 1H), 3.30 (dd, *J* 15.5, 8.8 Hz, 3H), 3.20 (q, *J* 6.7 Hz, 2H), 1.51 (sext, *J* 7.3 Hz, 2H), 1.28 (t, *J* 7.1 Hz, 3H), 0.87 (t, *J* 7.4 Hz, 3H); ^{13}C NMR ($CDCl_3$): 170.4, 159.9, 154.4, 151.7, 141.0, 129.7, 126.9, 124.5, 114.8, 83.0, 74.3, 61.4, 55.4, 43.6, 42.6, 37.6, 23.1, 14.3, 11.6. HRMS *m/z* $[M + H]^+$ calculated for $C_{23}H_{26}N_6O_2$: 419.2195. Found: 419.2206.

9-Benzyl-2-benzylamino-6-(ethoxy-carbonyl-methylamino)-8-ethynylpurine (13f)

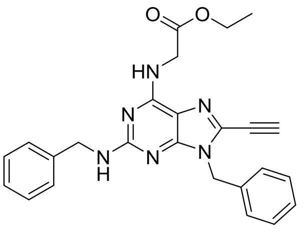

Amberlite IRA-67 (356 mg, 1.99 mmol), CuI (16 mg, 0.084 mmol) and $PdCl_2(PPh_3)_2$ (14 mg, 0.020 mmol) were added to a solution of **12f** (198 mg, 0.400 mmol) in dry THF (1.5 ml). The vial was capped, nitrogen was bubbled through the reaction mixture and ethynyltrimethylsilane (0.17 ml, 1.2 mmol) was added. The reaction mixture was heated at 115°C for 50 min, whereupon full conversion of starting material was confirmed by TLC (30% ethyl acetate in pentane). The reaction mixture was filtered through a short plug of silica and eluted with THF. The solvent volume was adjusted to 6 ml, polymer supported fluoride (203 mg) was added and the reaction mixture was stirred at room temperature overnight. Full deprotection was confirmed by TLC (30% ethyl acetate in pentane), the polymer was filtered off and washed with CH_2Cl_2 and THF. The solvents were removed under reduced pressure and purification by automated flash column chromatography (25% ethyl acetate in pentane) provided **13f** as a sticky brown solid (203 mg, 69%). ^{1}H NMR ($CDCl_3$): 7.38 – 7.21 (m, 10H), 6.34 (t, *J* 5.4 Hz, 1H), 5.43 (br s, 1H), 5.27 (s, 2H), 4.63 (d, *J* 6.0 Hz, 2H), 4.29 (br s, 2H), 4.18 (q, *J* 7.1 Hz, 2H), 3.40 (s, 1H), 1.25 (t, *J* 7.1 Hz, 3H); ^{13}C NMR ($CDCl_3$): 170.3, 160.2, 154.5, 151.5, 140.1, 136.4, 130.0, 128.6, 128.5, 128.3, 127.9, 127.6, 127.0, 114.6, 82.5, 74.0, 61.3, 46.5, 45.9, 42.6, 14.3. HRMS *m/z* $[M + H]^+$ calculated for $C_{25}H_{24}N_6O_2$: 441.2039. Found: 441.2017.

9-Benzyl-2-benzylamino-8-ethynyl-6-(methoxy-carbonyl-methyl-thio)purine (13g)

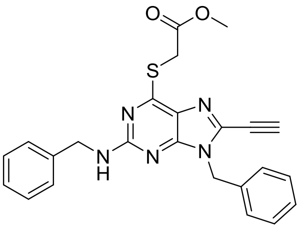

Amberlite IRA-67 (160 mg, 0.896 mmol), CuI (7.5 mg, 0.039 mmol) and $PdCl_2(PPh_3)_2$ (7.5 mg, 0.011 mmol) were added to a solution of **12g** (90 mg, 0.18 mmol) in dry THF (1.5 ml). The vial was capped, nitrogen was bubbled through the reaction mixture and ethynyltrimethylsilane (0.075 ml, 0.54 mmol) was added. The vial was heated at 115°C for 50 min, trace amounts of starting material could be observed by TLC (30% ethyl acetate in pentane). The reaction mixture was filtered through a short plug of silica and eluted with THF. The solvent volume was adjusted to 3 ml, polymer supported fluoride (94 mg) was added and the reaction mixture was stirred at room temperature overnight. Full deprotection was confirmed by TLC (30% ethyl acetate in pentane). The polymer was filtered off and washed with CH_2Cl_2 and THF. The solvents were removed under reduced pressure and the crude was passed through a silica column (20% ethyl acetate in pentane) which resulted in the isolation of a brown solid (40 mg, 50%). ^{1}H and ^{13}C NMR confirmed that **13g** was the major compound with impurities and the material was used in the next step without further purification. ^{1}H NMR ($CDCl_3$): 7.40 – 7.22 (m, 10H), 5.50 (br s, 1H), 5.29 (s, 2H), 4.65 (d, *J* 6.0 Hz, 2H), 4.00 (s, 2H), 3.68 (s, 3H), 3.45 (s, 1H); ^{13}C NMR ($CDCl_3$): 169.7, 159.8, 159.2, 150.8, 139.4, 135.9, 132.7, 128.8, 128.7, 128.2, 128.1, 127.5, 127.3, 124.7, 83.8, 73.5, 52.7, 46.7, 45.9, 30.8. HRMS *m/z* $[M + H]^+$ calculated for $C_{24}H_{21}N_5O_2S$: 444.1494. Found: 444.1487.

6-Amino-9-benzyl-2-benzylamino-8-ethynylpurine (13h)

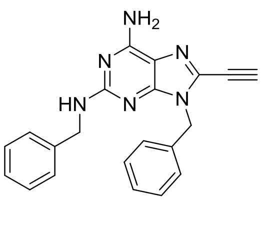

$PdCl_2(PPh_3)_2$ (26 mg, 0.04 mmol), CuI (28 mg, 0.15 mmol) and amberlite IRA-67 (646 mg, 3.62 mmol) were added to a solution of **12h** (296 mg, 0.72 mmol) in dry THF (5 ml). The vial was capped, nitrogen was bubbled through the reaction mixture and ethynyltrimethylsilane (400 µL, 2.89 mmol) was added. The reaction mixture was heated in a microwave reactor at 110°C for 50 min, after which full consumption of starting material was

confirmed by LCMS. The reaction mixture was filtered through a short plug of silica which was eluted with THF. The solvent volume was reduced to 15 ml and polymer supported fluoride (380 mg) was added. The reaction mixture was stirred at room temperature for 14 h. Full deprotection was confirmed by TLC (10% methanol in $CHCl_3$) and LCMS. The polymer was filtered off, washed with THF and CH_2Cl_2 and the solvents were removed under reduced pressure. The crude product was passed through a silica column (0-5% methanol in $CHCl_3$) which provided **13h** as a yellow solid (211 mg, 82%). The isolated material contained unidentified impurities and was used without further purification in the next step. ^{1}H NMR ($CDCl_3$): 7.37 – 7.23 (m, 8H), 5.45 (br s, 2H), 5.34 (t, *J* 5.9 Hz, 1H), 5.27 (s, 2H), 4.65 (d, *J* = 5.6 Hz, 2H), 3.42 (s, 1H); ^{13}C NMR ($CDCl_3$): 159.9, 155.4, 152.0, 139.8, 136.3, 130.7, 128.8, 128.6, 128.4, 128.1, 127.6, 127.2, 114.2, 82.8, 73.8, 46.7, 45.9. HRMS *m/z* $[M + H]^+$ calculated for $C_{21}H_{18}N_6$: 355.1671. Found: 355.1683.

6-Amino-8-ethynyl-9-(2-indanyl)-2-(1-propylamino)purine (13i)

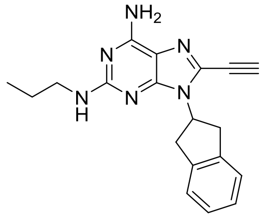

$PdCl_2(PPh_3)_2$ (22 mg, 0.031 mmol), CuI (22 mg, 0.12 mmol), amberlite IRA-67 (543 mg, 3.04 mmol) were added to a solution of **12i** (235 mg, 0.607 mmol) in dry THF (5 ml). The vial was capped, nitrogen was bubbled through the reaction mixture and ethynyltrimethylsilane (340 µl, 2.46 mmol) was added. The yellow reaction mixture was heated in a microwave reactor at 110°C for 50 min. Full consumption of **12i** was confirmed by TLC (10% methanol in $CHCl_3$). The dark reaction mixture was filtered through a short plug of silica which was eluted with THF. The volume was adjusted to 7 ml and polymer supported fluoride (320 mg) was added. The reaction mixture was stirred at room temperature under nitrogen for 24 h. Full consumption of the starting material was confirmed by TLC (10% methanol in $CHCl_3$) after 24 h. The polymer was removed by filtration, washed with THF and CH_2Cl_2 and the solvents were removed under reduced pressure. The crude was purified by flash column chromatography (3% methanol in CH_2Cl_2) to provide **13i** as a yellow solid (103 mg, 51%). ^{1}H NMR ($CDCl_3$): 7.26 – 7.18 (m, 4H), 5.48 (quin, *J* 9.0 Hz, 1H), 5.28 (br s, 2H), 4.78 (t, *J* 5.8 Hz, 1H), 3.91 (dd, *J* 15.6, 9.1 Hz, 1H),3.36 (s, 1H), 3.33 (dd, *J* 15.9, 8.8 Hz, 2H), 3.19 (q, *J* 6.6 Hz, 2H), 1.51 (sext, *J* 7.3 Hz, 2H), 0.87 (t, *J* 7.4 Hz, 3H); ^{13}C NMR ($CDCl_3$): 160.1, 155.7, 152.2, 141.0, 130.2, 126.9, 124.5, 114.5, 83.1, 74.1, 55.4, 43.6, 37.6, 23.0, 11.6. HRMS *m/z* $[M + H]^+$ calculated for $C_{19}H_{20}N_6$: 333.1827. Found: 333.1819.

2-Benzylamino-6-dimethylamino-9-(2-indanyl)-8-(1-methyl-1H-1,2,3-triazol-4-yl)purine (14a)

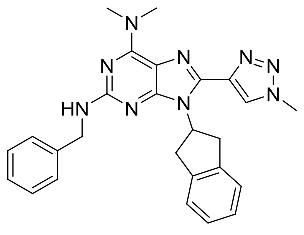

Methyl iodide (23 µl, 0.37 mmol) and sodium azide (24 mg, 0.37 mmol) in dry DMF (2 ml) were stirred at room temperature for 3 h in a sealed microwave vial. The resulting methyl azide solution was transferred to a microwave vial containing **13a** (67 mg, 0.16 mmol), sodium ascorbate (10 mg, 0.051 mmol), CuI (7 mg, 0.04 mmol) and DMEDA (4.5 µl, 0.042 mmol) in dry DMF (1 ml). The reaction mixture was heated at 60°C for 3 h. Full consumption of starting material was confirmed by TLC (20% ethyl acetate in pentane). The reaction mixture was diluted with water (20 ml), ethyl acetate (20 ml) and basified via addition of 0.5 M NaOH (aq.). *Caution! The aqueous phase should be kept slightly basic to avoid hydrazoic acid formation.* The aqueous phase was extracted with ethyl acetate (20 ml) and the combined organic phases were washed with brine, dried over Na_2SO_4 and the solvent was removed under reduced pressure. The crude product was purified by flash column chromatography (50% ethyl acetate in pentane) to provide **14a** as a

colourless oil (63 mg, 83%). ^{1}H NMR ($CDCl_3$): 8.04 (s, 1H), 7.24 – 7.14 (m, 9H), 6.33 (quin, *J* 9.1 Hz, 1H), 5.14 (br s, 1H), 4.38 (d, *J* 6.1 Hz, 2H), 4.11 (s, 3H), 4.07 (dd, *J* 15.2, 8.8 Hz, 2H), 3.46 (br s, 6H), 3.26 (dd, *J* 15.2, 8.8 Hz, 2H); ^{13}C NMR ($CDCl_3$): 157.9, 155.1, 154.4, 141.6, 141.4, 140.6, 137.5, 128.2, 127.7, 126.6, 126.4, 125.0, 124.4, 115.7, 55.9, 45.9, 38.3, 37.0, 36.8. HRMS *m/z* $[M + H]^+$ calculated for $C_{26}H_{27}N_9$: 466.2467. Found: 466.2453.

6-Dimetylamino-9-(2-indanyl)-8-(1-methyl-1H-1,2,3-triazol-4-yl)-2-(1-propylamino)purine (14b)

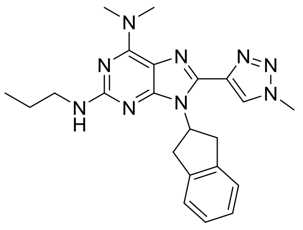

Methyl iodide (0.021 ml, 0.34 mmol) and sodium azide (22 mg, 0.34 mmol) in dry DMF (2 ml) were stirred at room temperature for 3 h in an oven-dried microwave vial. The methyl azide solution was transferred to a microwave vial containing **13b** (60 mg, 0.17 mmol), sodium ascorbate (10 mg, 0.05 mmol), CuI (7 mg, 0.04 mmol) and DMEDA (4 µl, 0.03 mmol) in dry DMF (1.5 ml). The microwave vial was capped and the mixture was heated at 60°C for 3 h. The mixture was allowed to reach room temperature and stirred for 91 h. The reaction was monitored by TLC (50% ethyl acetate in pentane). The reaction mixture was added to ethyl acetate (20 ml), extracted with 0.5 M NaOH (aq., 3 x 20 ml), the organic phase was dried over $MgSO_4$ and solvents were removed under reduced pressure. The crude product was purified by automated flash column chromatography (40% ethyl acetate in pentane) to provide compound **14b** as a beige foam (36 mg, 52%). ^{1}H NMR ($CDCl_3$): 8.04 (s, 1H), 7.23 – 7.14 (m, 4H), 6.32 (quin, *J* 9.1 Hz, 1H), 4.67 (t, *J* 5.9 Hz, 1H), 4.13 (s, 3H), 4.10 (dd, *J* 15.3, 9.2 Hz, 1H), 3.46 (s, 6H), 3.30 (dd, *J* 15.3, 8.9 Hz, 2H), 3.13 (q, *J* 6.7 Hz, 2H), 1.48 (sext, *J* 7.3 Hz, 2H), 0.81 (t, *J* 7.4 Hz, 3H); ^{13}C NMR ($CDCl_3$): 158.4, 155.1, 154.5, 141.7, 141.5, 137.2, 126.3, 124.9, 124.3, 115.4, 55.6, 43.6, 38.1, 37.0, 36.7, 23.1, 11.5. HRMS *m/z* $[M + H]^+$ calculated for $C_{22}H_{27}N_9$: 418.2467. Found: 418.2454.

6-Dimethylamino-9-(2-indanyl)-8-(1-methyl-1H-1,2,3-triazol-4-yl)-2-(*p*-chloro-benzylamino)purine (14c)

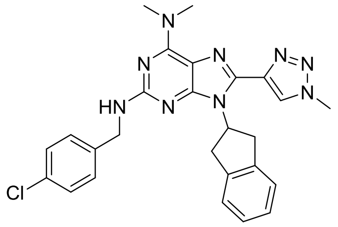

Methyl iodide (41 µl, 0.66 mmol) and sodium azide (43 mg, 0.66 mmol) in dry DMF (3 ml) were stirred at room temperature for 3 h in a sealed microwave vial. The resulting methyl azide solution was transferred to a microwave vial containing **13c** (140 mg, 0.316 mmol), sodium ascorbate (19 mg, 0.096 mmol), CuI (12 mg, 0.063 mmol) and DMEDA (8.5 µl, 0.079 mmol) in dry DMF (1.5 ml). The reaction mixture was heated at 60°C for 3 h. Complete conversion of starting material was indicated by TLC (50% ethyl acetate in pentane). The reaction mixture was diluted with water (25 ml), ethyl acetate (20 ml) and basified via addition of 0.5 M NaOH (aq.). *Caution! The aqueous phase should be kept slightly basic to avoid hydrazoic acid formation.* The aqueous phase was extracted with ethyl acetate (2 x 20 ml) and the combined organic phases were washed with brine and dried over Na_2SO_4. The solvent was removed under reduced pressure and the crude purified via flash column chromatography (45% ethyl acetate in pentane) to provide **14c** as pale yellow solid (138 mg, 87%). ^{1}H NMR ($CDCl_3$): 8.05 (s, 1H), 7.21 – 7.16 (m, 4H), 7.11 – 7.03 (m, 4H), 6.34 (quin, *J* 9.1 Hz, 1H), 5.30 (br s, 1H), 4.29 (d, *J* 6.0 Hz, 2H), 4.09 (s, 3H), 4.02 (dd, *J* 15.2, 9.4 Hz, 2H), 3.44 (br s, 6H), 3.25 (dd, *J* 15.2, 8.8 Hz, 2H); ^{13}C NMR ($CDCl_3$): 157.7, 155.0, 154.2, 141.5, 141.3, 139.3, 137.5, 132.0, 128.8, 128.1, 126.4, 125.0, 124.4, 115.8, 55.8, 45.2, 38.2, 36.8, 36.7. HRMS *m/z* $[M + H]^+$ calculated for $C_{26}H_{26}ClN_9$: 500.2078. Found: 500.2081.

2-Benzylamino-6-(ethoxy-carbonyl-methylamino)-9-(2-indanyl)-8-(1-methyl-1,2,3-triazol-4-yl)purine (14d)

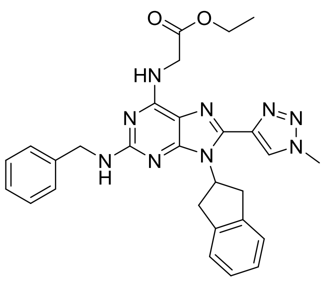

Methyl iodide (22 µl, 0.35 mmol) and sodium azide (23 mg, 0.35 mmol) in dry DMF (2 ml) were stirred at room temperature for 3 h in a microwave vial. The methyl azide solution was transferred to a microwave vial containing **13d** (80 mg, 0.17 mmol), sodium ascorbate (11 mg, 0.056 mmol), CuI (7 mg, 0.04 mmol) and DMEDA (5 µl, 0.05 mmol) in dry DMF (1 ml). The reaction mixture was heated at 60°C for 3 h. Full consumption of **13d** was confirmed by TLC (50% ethyl acetate in pentane). The reaction mixture was diluted with water (20 ml) and ethyl acetate (20 ml). The aqueous phase was extracted with ethyl acetate (20 ml) and the combined organic phases were washed with brine and dried over Na_2SO_4. *Caution: Keep aqueous phase slightly basic (0.5 M NaOH (aq.) was added to the aqueous phase) to avoid hydrazoic acid formation.* The solvents were removed under reduced pressure and purification by flash column chromatography (80% ethyl acetate in pentane) provided **14d** as an off white solid (76 mg, 85%). ^{1}H NMR ($CDCl_3$): 8.13 (s, 1H), 7.23 – 7.13 (m, 9H), 6.63 (t, *J* 5.3 Hz, 1H), 6.39 (quin, *J* 9.0 Hz, 1H), 5.33 (br s, 1H), 4.36 (d *J* 5.6 Hz, 2H), 4.21 (br s, 2H), 4.16 (q, *J* 14.3, 7.1 Hz, 2H), 4.06 (s, 3H), 4.03 (dd, 15.2, 9.3 Hz, 2H), 3.28 (dd, *J* 15.2, 8.8 Hz, 2H), 1.25 (t, *J* 7.1 Hz, 3H); ^{13}C NMR ($CDCl_3$): 170.8, 158.4, 154.4, 153.2, 141.5, 140.9, 140.3, 139.1, 128.3, 127.7, 126.7, 126.5, 125.4, 124.4, 115.1, 61.2, 56.1, 45.9, 42.5, 37.1, 36.8, 14.3. HRMS *m/z* $[M + H]^+$ calculated for $C_{28}H_{29}N_9O_2$: 524.2522. Found: 524.2546.

8-(1-Benzyl-1H-1,2,3-triazol-4-yl)-6-(ethoxy-carbonyl-methylamino)-9-(2-indanyl)-2-(1-propylamino)purine (14e)

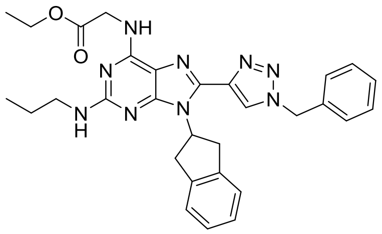

Benzyl bromide (82 mg, 0.196 mmol) and sodium azide (26 mg, 0.400 mmol) in dry DMF (2 ml) were heated in a microwave reactor at 100°C for 30 min. The resulting benzyl azide solution was transferred to a microwave vial containing **13e** (82 mg, 0.196 mmol), sodium ascorbate (12 mg, 0.061 mmol), CuI (7 mg, 0.04 mmol) and DMEDA (5 µl, 0.05 mmol) in DMF (1.5 ml). The reaction mixture was stirred at room temperature for 19 h. Full consumption of **13e** was confirmed by TLC (50% ethyl acetate in pentane). The reaction mixture was poured in to ethyl acetate (40 ml), extracted with water (3 x 20 ml), dried over Na_2SO_4 and the solvents were removed under reduced pressure. *Caution: Keep aqueous phase slightly basic (0.5 M NaOH (aq.) was added to the aqueous phase) to avoid hydrazoic acid formation.* The crude product was purified by flash column chromatography (50% ethyl acetate in pentane) to provide **14e** as a pale yellow solid (80 mg, 74%). ^{1}H NMR ($CDCl_3$): 8.05 (s, 1H), 7.41 – 7.29 (m, 5H), 7.25 – 7.15 (m, 4H), 6.40 (quin, *J* 9.0 Hz, 1H), 6.14 (s, 1H), 5.54 (s, 2H), 4.81 (t, *J* 5.9 Hz, 1H), 4.27 (d, *J* 5.2 Hz, 2H), 4.20 (q, *J* 7.1 Hz, 2H), 4.07 (dd, *J* 15.3, 9.1 Hz, 2H), 3.33 (dd, *J* 15.3, 8.9 Hz, 2H), 3.11 (q, *J* 6.4 Hz, 2H), 1.46 (sext, *J* 7.3 Hz, 2H), 1.26 (t, *J* 7.1 Hz, 3H), 0.81 (t, *J* 7.4 Hz, 3H); ^{13}C NMR ($CDCl_3$): 170.7, 158.8, 154.3, 153.4, 141.6, 141.4, 138.9, 134.2, 129.3, 129.0, 128.5, 126.5, 124.4, 124.1, 114.9, 61.2, 56.0, 54.5, 43.6, 42.6, 37.3, 23.0, 14.3, 11.6. HRMS *m/z* $[M + H]^+$ calculated for $C_{30}H_{33}N_9O_2$: 552.2835. Found: 552.2856.

6-(Ethoxy-carbonyl-methylamino)-9-(2-indanyl)-8-(1-methyl-1H-1,2,3-triazol-4-yl)-2-(1-propylamino)purine (14f)

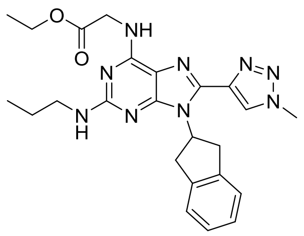

Methyl iodide (24 µl, 0.39 mmol) and sodium azide (25 mg, 0.38 mmol) in dry DMF (2 ml) were stirred at room temperature for 3 h in a microwave vial. The formed methyl azide solution was then transferred to a microwave vial containing **x** (82 mg, 0.20 mmol), sodium ascorbate (12 mg, 0.06 mmol), CuI (7 mg, 0.04 mmol) and DMEDA (5 µl, 0.05 mmol) in dry DMF (1.5 ml). The reaction mixture was heated at 60°C for 3 h and then at room temperature for 15 h. Full consumption of **13e** was confirmed by TLC (10% methanol in $CHCl_3$). The reaction mixture was poured in to ethyl acetate (25 ml), extracted with water (3 x 20 ml), dried over Na_2SO_4 and the solvents were removed under reduced pressure. *Caution: Keep aqueous phase slightly basic (0.5 M NaOH (aq.) was added to the aqueous phase) to avoid hydrazoic acid formation.* Purification by flash column chromatography (ethyl acetate) provided **14e** as a yellow solid (81 mg, 87%). ^{1}H NMR ($CDCl_3$): 8.11 (br s, 1H), 7.25-7.13 (m, 4H), 6.31 (br s, 1H), 6.16 (br s, 1H), 4.75 (br s, 4.75, 1H), 4.33 (s, 2H), 4.24 (q, *J* 7.1 Hz, 2H),4.14 (s, 3H), 4.05 (dd, *J* 14.9, 8.5 Hz, 2H), 3.32 (dd, *J* 14.8, 8.9 Hz, 2H), 3.12 (q, *J* 6.6 Hz, 2H), 1.46 (q, *J* 7.2 Hz, 1H), 1.30 (t, *J* 7.0 Hz, 3H), 0.81 (t, *J* 7.4 Hz, 3H); ^{13}C NMR ($CDCl_3$): 170.9, 158.9, 154.4, 153.4, 141.6, 140.9, 139.0, 126.5, 125.4, 124.4, 114.7, 61.2, 56.1, 43.6, 42.5, 37.2, 36.8, 23.0, 14.3, 11.5. *Note: Weighting functions were applied to the ^{1}H NMR* spectrum *to obtain J couplings (sine bell: 2°, sine square: 88°).* HRMS *m/z* $[M + H]^+$ calculated for $C_{24}H_{29}N_9O_2$: 476.2522. Found: 476.2485.

9-Benzyl-2-benzylamino-8-(1-benzyl-1,2,3-triazol-4-yl)-6-(ethoxy-carbonyl-methylamino)purine (14g)

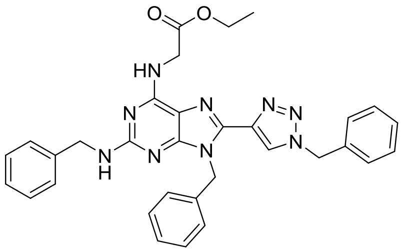

Benzyl bromide (60 µl, 0.51 mmol) and sodium azide (32 mg, 0.49 mmol) in dry DMF (1 ml) were stirred at 105 °C for 1 h. The benzyl azide solution was transferred to a microwave vial containing **13f** (106 mg, 0.241 mmol), sodium ascorbate (19 mg, 0.096 mmol), CuI (9 mg, 0.05 mmol) and DMEDA (10.5 µl, 0.0974 mmol) in dry DMF (1.6 ml). The reaction mixture was stirred at room temperature overnight. The reaction was monitored by TLC (30% ethyl acetate in pentane) which indicated full consumption of the starting material. The reaction mixture was diluted with water (20 ml), basified (pH ~ 9) with sat. $NaHCO_3$ (aq.) and extracted with ethyl acetate (4 x 20 ml and 2 x 10 ml). *Caution: Keep aqueous phase slightly basic (0.5 M NaOH (aq.) was added to the aqueous phase) to avoid hydrazoic acid formation.* The combined organic phases were washed with brine and dried over Na_2SO_4. The solvents were removed by co-evaporation with toluene and the crude material was purified by preparative HPLC (50-100% acetonitrile in water with 0.1% TFA). The solvents were removed under reduced pressure, the product was dissolved in CH_2Cl_2 (25 ml) and washed with 5% $NaHCO_3$ (2 x 15 ml), brine and dried over Na_2SO_4. After removal of the solvents under reduced pressure, **14g** was isolated as a yellow solid (64 mg, 46%). ^{1}H NMR ($CDCl_3$): 7.96 (s, 1H), 7.39 – 7.12 (m, 15H), 6.25 (br s, 1H), 5.86 (s, 2H), 5.49 (s, 2H), 5.37 (br s, 1H), 4.64 (d, *J* 6.0 Hz, 2H), 4.26 (d, *J* 5.4 Hz, 2H), 4.16 (q, *J* 7.1 Hz, 2H), 1.23 (t, *J* 7.1 Hz, 3H); ^{13}C NMR ($CDCl_3$): 170.6, 159.5, 154., 153.4, 141.1, 140.3, 138.9, 137.6, 134.2, 129.2, 129.0, 128.5, 128.4, 128.3, 128.3, 127.7, 127.4, 127.0, 123.7, 114.4, 61.3, 54.4, 46.5, 46.0, 42.7, 14.3. HRMS *m/z* $[M + H]^+$ calculated for $C_{32}H_{31}N_9O_2$: 574.2679. Found: 574.2666.

9-Benzyl-2-benzylamino-8-(1-benzyl-1,2,3-triazol-4-yl)-6-(methoxy-carbonyl-methylthio)purine (14h)

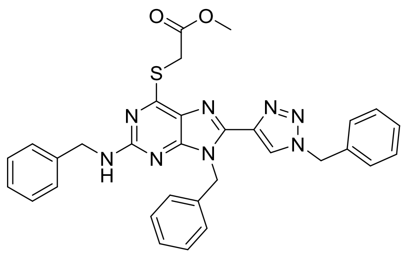

Benzyl bromide (20 µl, 0.17 mmol) and sodium azide (13 mg, 0.20 mmol) in dry DMF (0.5 ml) were stirred at 105 °C for 1 h. The benzyl azide solution was transferred to a microwave vial containing **13g** (37 mg, 0.083 mmol), sodium ascorbate (7 mg, 0.04 mmol), CuI (3 mg, 0.02 mmol) and DMEDA (5 µl, 0.05 mmol) in dry DMF (1 ml). The reaction mixture was stirred at room temperature overnight. Full consumption of **13g** was confirmed by TLC (10% ethyl acetate in pentane). The reaction mixture was diluted with water (10 ml) and ethyl acetate (10 ml) and basified (pH ~ 9) with sat. $NaHCO_3$ (aq.). The aqueous phase was extracted with ethyl acetate (5 x 10 ml) and the combined organic phases were washed with brine and dried over Na_2SO_4. *Caution: Keep aqueous phase slightly basic (0.5 M NaOH (aq.) was added to the aqueous phase) to avoid hydrazoic acid formation.* The solvents were removed by co-evaporation with toluene and the crude material was purified by preparative HPLC (50-100% acetonitrile in water with 0.1% TFA). The solvents were removed under reduced pressure, the product was dissolved in CH_2Cl_2 (15 ml) and washed with 5% $NaHCO_3$ (2 x 5 ml), brine and dried over Na_2SO_4. After removal of the solvents under reduced pressure, **14h** was isolated as a yellow solid (19 mg, 40%). ^1H NMR ($CDCl_3$): 8.13 (s, 1H), 7.41 – 7.23 (m, 12H), 7.20 – 7.16 (m, 3H), 5.94 (s, 2H), 5.55 (s, 2H), 5.46 (t, *J* 6.0 Hz, 1H), 4.68 (d, *J* 5.8 Hz, 2H), 4.03 (s, 2H), 3.67 (s, 3H); ^{13}C-NMR ($CDCl_3$): 169.8, 158.6, 157.9, 152.9, 141.6, 140.8, 139.7, 137.1, 133.9, 129.4, 129.2, 128.7, 128.5, 128.5, 128.3, 127.6, 127.6, 127.3, 125.0, 124.8, 54.6, 52.7, 46.8, 46.0, 30.8. HRMS *m/z* $[M + H]^+$ calculated for $C_{31}H_{28}N_8O_2S$: 577.2134. Found: 577.2169.

6-Amino-9-benzyl-2-benzylamino-8-(1-methyl-1H-1,2,3-triazol-4-yl)-purine (14i)

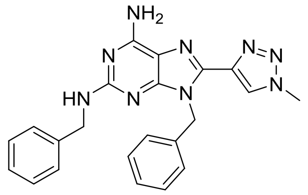

Methyl iodide (19 µl, 0.30 mmol) and sodium azide (20 mg, 0.31 mmol) in dry DMF (2 ml) were stirred at room temperature for 3 h. The resulting methyl azide solution was transferred to a microwave vial containing **13h** (53 mg, 0.15 mmol), sodium ascorbate (9 mg, 0.05 mmol), CuI (6 mg, 0.03 mmol) and DMEDA (4 µl, 0.04 mmol) in dry DMF (1.2 ml). The reaction mixture was heated at 60°C for 3 h. The reaction was monitored by TLC (eluting first with 50% ethyl acetate in pentane and then with 10% methanol in $CHCl_3$). Ethyl acetate (20 ml) was added and the organic phase was washed with water (2 x 20 ml), dried over Na_2SO_4 and the solvents were removed under reduced pressure. *Caution! The aqueous phase should be kept slightly basic to avoid hydrazoic acid formation.* Purification by preparative HPLC (14 ml/min, 50–100% MeCN in H_2O, 0.1% TFA) provided **14i** as a white solid (41 mg, 66%). ^1H NMR (Methanol-d_4): 8.41 (s, 1H), 7.39 – 7.33 (m, 2H), 7.31 – 7.12 (m, 8H), 5.83 (s, 2H), 4.65 (s, 2H), 4.17 (s, 3H); ^{13}C NMR (Methanol-d_4): 153.4 (elucidated by gHMBC), 152.9, 151.8, 143.7, 140.2, 139.7, 137.7, 129.62, 129.59, 128.83, 128.81, 128.7, 128.4, 127.3, 112.6, 47.9, 46.1, 37.2. HRMS *m/z* $[M + H]^+$ calculated for $C_{22}H_{21}N_9$: 412.1998. Found: 412.1989.

6-Amino-9-(2-indanyl)-8-(1-methyl-1H-1,2,3-triazol-4-yl)-2-(1-propylamino)purine (14j)

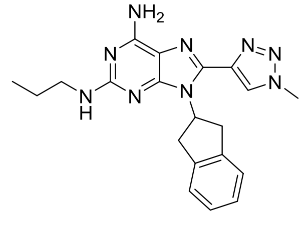

Methyl iodide (27 µl, 0.43 mmol) and sodium azide (27 mg, 0.42 mmol) were stirred in dry DMF (2 ml) at room temperature for 3 h. The resulting methyl azide solution was transferred to a vial containing **13i** (72 mg, 0.22 mmol) sodium ascorbate (13 mg, 0.066 mmol), CuI (9 mg, 0.047

mmol) and DMEDA (6 µl, 0.06 mmol) in dry DMF (1.5 ml). The reaction mixture was heated at 60ºC for 3 h. Consumption of **13i** was confirmed by TLC (10% methanol in $CHCl_3$). The reaction mixture was poured in to ethyl acetate (25 ml), the organic phase was extracted with water (3 x 20 ml), diluted with ethyl acetate (20 ml), dried over Na_2SO_4 and the solvents were removed to provide the crude product as a yellow solid (71 mg, 82%). *Caution: Keep aqueous phase slightly basic, 0.5 M NaOH (aq.) was added to the aqueous phase, to avoid hydrazoic acid formation*. A portion of the crude product (50 mg) was purified by automated flash column chromatography (0-3% methanol in CH_2Cl_2) to provide **14j** as a white solid (38 mg, 62%). ^{1}H NMR ($CDCl_3$): 8.08 (s, 1H), 7.26 – 7.14 (m, 4H), 6.31 (quin, *J* 8.9 Hz, 1H), 5.24 (s, 2H), 4.74 (br t, *J* 5.3 Hz, 2H), 4.16 (s, 3H), 4.05 (dd, *J* 15.4, 8.9 Hz, 2H), 3.35 (dd, *J* 15.4, 8.9 Hz, 2H), 3.09 (q, *J* 6.6 Hz, 2H), 1.46 (sext, *J* 7.4 Hz, 2H), 0.81 (t, *J* 7.4 Hz, 3H); ^{13}C NMR ($CDCl_3$): 158.8, 155.3, 153.8, 141.4, 141.0, 139.4, 126.4, 125.0, 124.3, 114.5, 55.9, 43.5, 37.3, 36.9, 22.9, 11.4. HRMS *m/z* $[M + H]^+$ calculated for $C_{20}H_{23}N_9$: 390.2154. Found: 390.2163.

2-Benzylamino-6-dimethylamino-8-(1-methyl-1H-1,2,3-triazol-4-yl)-9-phenethylpurine (14k)

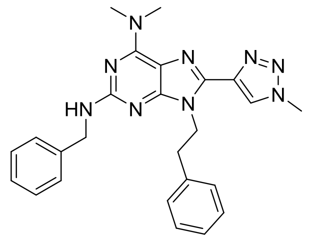

Methyl iodide (18 µl, 0.29 mmol) and sodium azide (18 mg, 0.29 mmol) in DMF (2 ml) were stirred at room temperature for 3 h. The resulting methyl azide solution was transferred to a microwave vial containing **5a** (56 mg, 0.14 mmol), sodium ascorbate (8.5 mg, 0.043 mmol), CuI (5.6 mg, 0.03 mmol) and DMEDA (4 µl, 0.04 mmol) in DMF (1.5 ml). The reaction mixture was heated at 60°C for 3 h. The reaction was monitored by TLC (50% ethyl acetate in heptane). Ethyl acetate (20 ml) was added and the organic phase was washed with water (3 x 20 ml), dried over Na_2SO_4 and the solvents were removed under reduced pressure. *Caution! The aqueous phase should be kept slightly basic to avoid hydrazoic acid formation.* Purification by flash column chromatography (dry loaded from Celite®, 50% ethyl acetate in pentane) provided **14k** as a beige solid (34 mg, 53%). ^{1}H NMR ($CDCl_3$): 7.90 (s, 1H), 7.47 – 7.40 (m, 2H), 7.37 – 7.32 (m, 2H), 7.28 – 7.13 (m, 1H), 5.18 (t, *J* 6.0 Hz, 1H), 4.86 – 4.78 (m, 2H), 4.68 (d, *J* 5.8 Hz, 2H), 4.13 (s, 3H), 3.46 (s, 6H), 3.15 – 3.06 (m, 2H); ^{13}C NMR ($CDCl_3$): 159.0, 155.0, 154.2, 141.4, 141.4, 140.8, 138.8, 137.3, 129.3, 128.5, 128.3, 127.8, 126.9, 126.3, 124.3, 115.1, 46.2, 44.8, 38.3, 36.8, 36.2. HRMS *m/z* $[M + H]^+$ calculated for $C_{25}H_{27}N_9$: 454.2468. Found: 454.2448.

2-Benzylamino-9-cyclophentylmethyl-6-dimethylamino-8-(1-methyl-1H-1,2,3-triazol-4-yl)purine (14l)

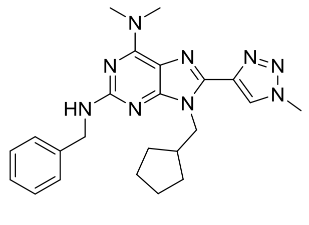

Methyl iodide (14 µl, 0.23 mmol) and sodium azide (14 mg, 0.22 mmol) in dry DMF (2 ml) were stirred at room temperature for 3 h. The resulting methyl azide solution was transferred to a microwave vial containing **6c** (40 mg, 0.11 mmol), CuI (4 mg, 0.02 mmol), sodium ascorbate (14 mg, 0.035 mmol), DMEDA (3 µl, 0.03 mmol) in dry DMF (2 ml). The reaction mixture was heated at 60ºC for 3 h and then at room temperature overnight. Full conversion of starting material was confirmed by TLC (30% ethyl acetate in pentane). The reaction mixture was diluted with ethyl acetate (30 ml) and water (20 ml). The aqueous phase was extracted with ethyl acetate (3 x 20 ml) and the combined organic phases were washed with brine and dried over Na_2SO_4. The solvent was removed under reduced pressure. The crude product was purified by automated flash column chromatography (35% ethyl acetate in pentane) to provide **14l** as a

brown solid (31 mg, 67%). ^{1}H NMR ($CDCl_3$): 8.08 (s, 1H), 7.41 – 7.39 (m, 2H), 7.36 – 7.26 (m, 2H), 7.25 – 7.19 (m, 1H), 5.13 (br s, 1H), 4.65 (d, *J* 5.9 Hz, 2H), 4.58 (d, *J* 7.7 Hz, 2H), 4.15 (s, 3H), 3.45 (br s, 6H), 2.49 (sep, *J* 7.6 Hz, 1H), 1.65 – 1.48 (m, 4H), 1.47 – 1.37 (m, 2H), 1.35 – 1.21 (m, 2H); ^{13}C NMR ($CDCl_3$): 158.8, 155.0, 154.6, 141.9, 140.7, 137.6, 128.4, 127.8, 126.9, 124.5, 115.0, 47.7, 46.1, 40.5, 38.3, 36.9, 30.1, 25.0. HRMS *m/z* $[M + H]^+$ calculated for $C_{23}H_{29}N_9$: 432.2624. Found: 432.2630.

NOE build-up analysis

All NOESY spectra were recorded on Varian MR 400 MHz spectrometer. Compounds **8a** and **7a** (Fig. S1) were dissolved in DMSO-d$_6$ in a concentration of 0.011 M and 0.012M respectively. Chemical shifts are reported in parts per million with the solvent residual signal used as internal standard: DMSO-d$_6$ [δ_H 2.50, δ_C 39.52]. The NOE-build up was performed with six mixing times ranging from 80-1000 ms. The relaxation delay was set to 2.0 s, 16 scans were recorded with 4096 points in the direct and 128 points in the indirect dimension. Distances were calculated with the reference distance of 2.508 Å, measured for aromatic ortho protons. The NOE peak intensities were calculated using normalisation of both crosspeaks and both diagonalpeaks ([crosspeak$_1$ × crosspeak$_2$]/[diagonalpeak$_1$ × diagonalpeak$_2$])$^{0.5}$. At least three mixing times giving a linear (r^2>0.95) initial NOE rate were used to determine σ_{ij} build-up rates according to the equation $r_{ij}=r_{ref}(\sigma_{ref}/\sigma_{ij})^{(1/6)}$, where r_{ij} is the distance between protons i and j in Ångström and σ_{ij} is the normalized intensity obtained from NOESY experiments. Calculated distances are shown in Table S1 and S2, for **8a** and **7a**, respectively. In addition, build-up curves for **8a** and **7a** are shown in Fig. S2 and S3, respectively.

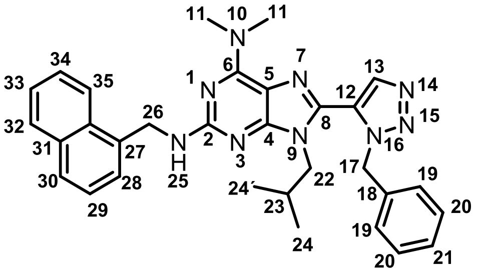

8a

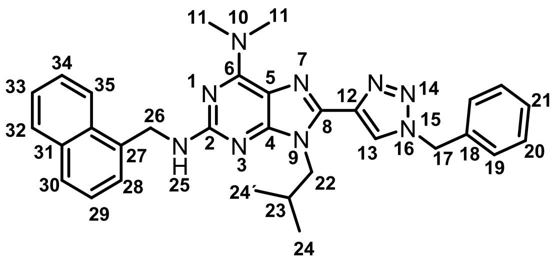

7a

Fig. A. Structure and numbering of **8a** and **7a**.

Table A. Interproton distances (Å) for **8a** as derived from NOE build up measurement.

	**Chemical shift**		**Normalised peak area/mixing time**								
**Interaction**	**f1 (ppm)**	**f2 (ppm)**	**80 ms**	**100 ms**	**200 ms**	**400 ms**	**600 ms**	**800 ms**	**σ**	**r^2**	**r_{ij} (Å)**
13-22	8.36	3.89	0.004802	0.006058	0.012057	0.022961	0.035101	0.048465	6.00E-05	1.0	2.23
13-24	8.36	0.45	0.001037	0.001171	0.002055	0.003365	0.005178	0.00708	9.00E-06	1.0	3.07
17-24	5.97	0.45	0.000114	0.000169	0.000366		0.001045	0.001318	2.00E-06	1.0	3.94
13-23	8.36	1.59	0.001559	0.001856	0.003235	0.007118	0.011413	0.014062	2.00E-05	1.0	2.68
19-24	7.05	0.45	0.000353	0.000376	0.00057	0.001157	0.001608	0.002094	3.00E-06	0.98	3.68
19-23	7.05	1.59		0.000202	0.000701		0.001957	0.00292	3.00E-06	0.99	3.68
19-17	7.05	5.97	0.003309	0.004058	0.008346	0.016767	0.026033	0.035605	4.00E-05	1.0	2.39
28-29	7.41	7.79				0.009347	0.017407	0.026815	3.00E-05	0.95	2.508

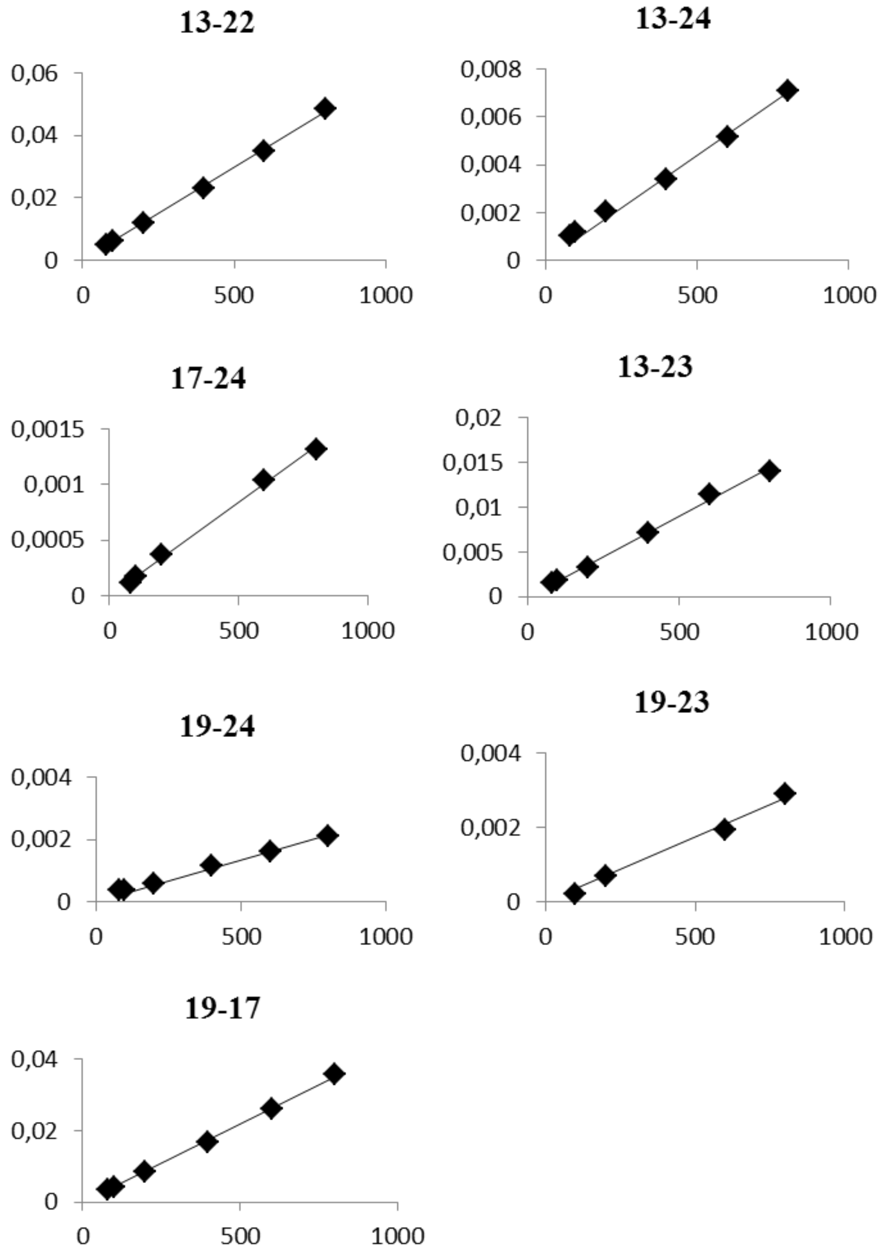

Fig. B. Build-curves for **8a**.

Table B. Interproton distances (Å) for **7a** as derived from NOE build up measurement.

	Chemical shift		Normalised peak area/mixing time								
**Interaction**	**f1 (ppm)**	**f2 (ppm)**	**80 ms**	**200 ms**	**400 ms**	**600 ms**	**800 ms**	**1000 ms**	**σ**	**r^2**	**r_{ij} (Å)**
13-17	8.70	5.67	0.001246	0.002817	0.005731	0.009072	0.011637	0.014852	1.00E-05	1	3.16
13-22	8.70	4.29	0.000494	0.000967	0.002	0.002827	0.004012	0.005758	5.00E-06	0.98	3.55
30-29	7.92	7.79		0.004172	0.015076	0.024697	0.031862	0.038384	4.00E-05	0.98	2.508

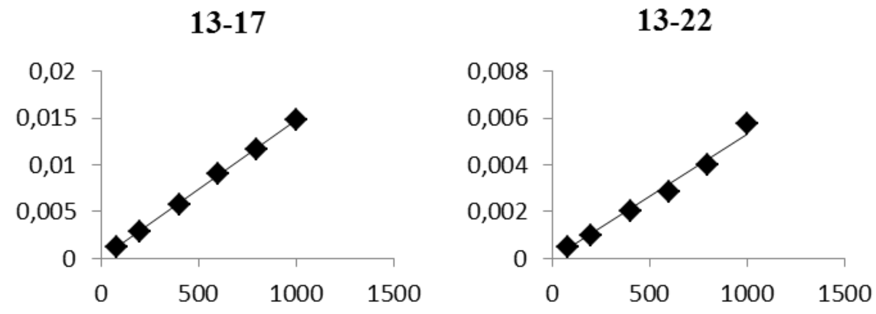

Fig. C. Build up curves for **7a**.

Computational conformation analysis

All computational studies were performed using the software MacroModel[5]. Conformational searches for **8a** and **7a** were performed twice, using the OPLS-2005 and the Amber* force fields with several different solvation models. For OPLS-2005, water, chloroform and octanol were used and for Amber*, the water solvation model was used. The energy window for saving structures was set to 42 kJ/mol and RMSD cut off was set to 1-2 Å (1 Å for OPLS-2005 and water solvation for **8a**, otherwise 2 Å). The torsional sampling mode (MCMM) was employed with 10000 steps per rotable bond. PRCG was used as the minimization method with a maximum of 2500 iterations. For **8a** and **7a** 963 and 711 conformations were obtained, respectively. The ensembles derived from calculations with different force fields and/or solvent models were combined followed by elimination of redundant conformations by comparison of the heavy atom coordinates applying a RMSD cutoff 2.5 Å. For **8a** and **7a**, 59 and 42 unique conformations were obtained, respectively.

Ensamble analysis using NAMFIS

Following a previously described protocol[6], ensemble calculation analysis was performed by fitting the experimentally measured distances to the computationally predicted conformations. Methyl, eqvivalent methylene and eqvivalent ortho protons were treated accoridng to the equation d= $\{[(d_1)^{-6}+ (d_2)^{-6} + (d_3)^{-6}+ (d_n)^{-6}]/n^{-1/6}\}$, where n is the number of eqvivalnet protons e.g. for a methyl group n = 3[7]. The experimental obtained distances is presented in Tabel S3 and S4 for **8a**.

Table C. Result of the NAMFIS analysis of **8a**.

Msigma% = 0.594D+02

**Conformation**[x]	**% population**
**1**	**14**
**2**	**15**
**3**	**5**
**4**	**34**
**5**	**15**
**6**	**16**

[x]The structures of the main conformers are shown in Fig. S4 and S5.

Table D. Comparison of the experimentally observed and the NAMFIS determined interatomic distances (Å) for **8a**.

**H-H distances**	**Experimental (Å)**	**Calculated (Å)**
13-22	2.2	3.0
13-24	3.1	3.5
17-24	3.9	4.1
13-23	2.7	2.9
19-24	3.7	3.8
23-19	3.7	3.8
19-17	2.4	2.8
	**RMSD**	0.37

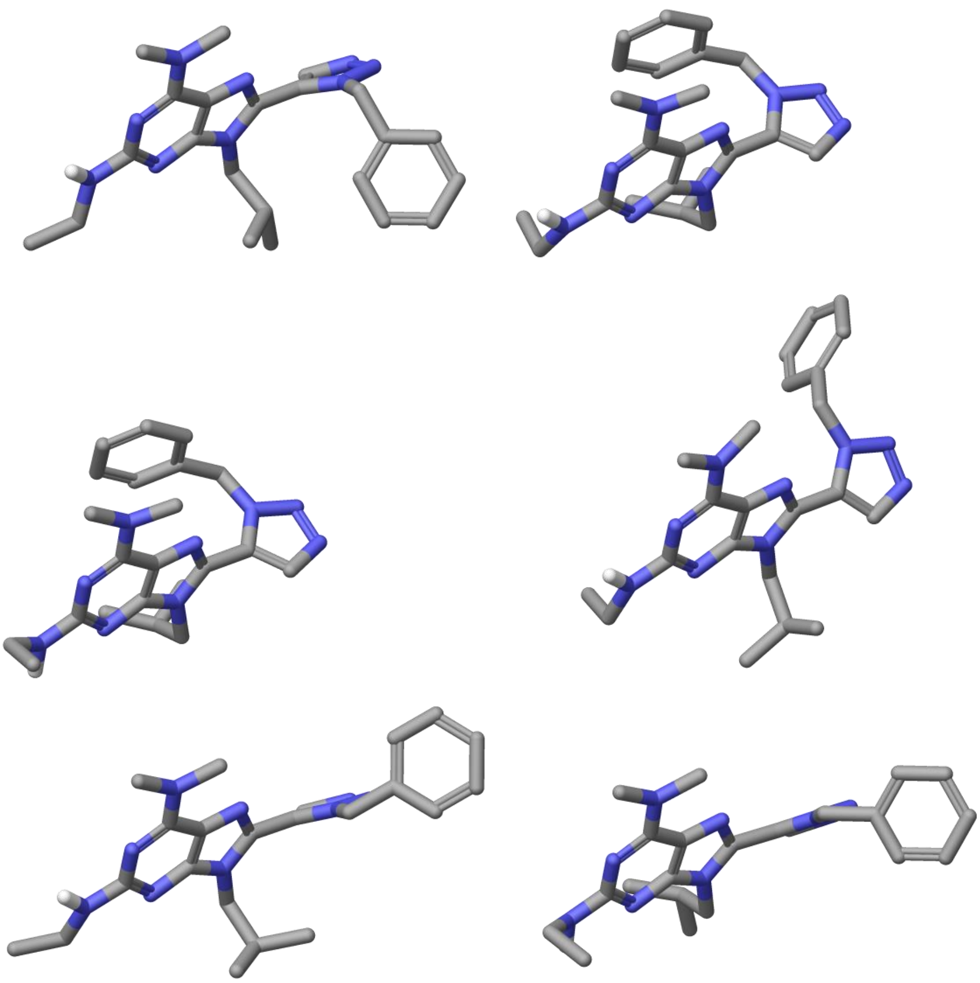

Fig. D. Solution conformations of **8a**, identified by the NAMFIS-analysis. Conformations with the highest probabilities are shown: Top left **1 (14%)**, top right **2 (15%)**, middle left **3 (5%)**, middle right **4 (34%)**, bottom left **5 (15%)** and bottom left **6 (16%)**. The non-polar hydrogens and the naphtyl substituent have been removed in these Fig.s due to clarity.

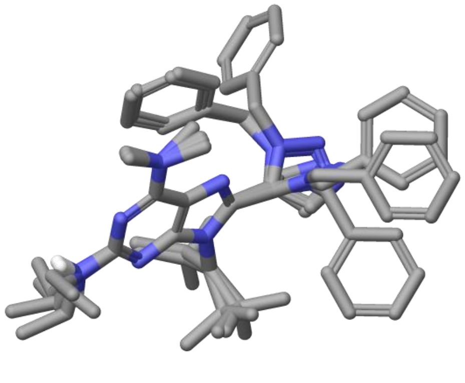

Fig. E. Superimposition of the most probable conformations (1-6), indicating the available conformational space of **8a**.

Table E. Result of the NAMFIS analysis using conformations 1-6 of **8a.**

Msigma% = 0.593D+02

Conformation[x]	% population
**1**	**14**
**2**	**11**
**3**	**10**
**4**	**35**
**5**	**14**
**6**	**16**

[x]The structures of the main conformers are shown in Fig. S4 and S5.

Table F. Comparison of the experimentally observed and the NAMFIS determined interatomic distances (Å) using conformation 1-6 of **8a**.

H-H distances	Experimental (Å)	Calculated (Å)
13-22	2.2	3.0
13-24	3.1	3.5
17-24	3.9	4.1
13-23	2.7	2.9
19-24	3.7	3.8
23-19	3.7	3.8
19-17	2.4	2.8
	**RMSD**	0.37

Emission quantum yield determination.

Emission quantum yields in methanol were determined at room temperature in a 1 cm quartz cuvette (V = 3.0 ml). Excitation wavelength was λ_{exc} = 300 nm for all compounds except **14h** for which λ_{exc} = 317 nm was used. For each compound, Raman corrected emission spectra of samples with $A \sim$ 0.08, 0.06, and 0.04 were recorded. The slope of the linear regression for integrated emission peak area (IA) *vs.* absorption (A) for the samples was referenced to that of fluorescent standard 2-aminopyridine in 0.05 M H_2SO_4 ($\Phi_{f,std}$ = 0.60) to give the sample quantum yield as;

$$\Phi_{f,sample} = \Phi_{f,std} \times \frac{\left(\frac{dIA}{dA}\right)_{sample}}{\left(\frac{dIA}{dA}\right)_{std}} \times \frac{\eta_{MeOH}^2}{\eta_{water}^2}$$

where η_{MeOH} and η_{water} is the refractive index for methanol (1.333) and water (1.328), respectively. Absorption spectra were recorded on a Cary 5000 UV/Vis spectrometer. Emission spectra were recorded on a Horiba Spex fluorolog 3.

Absorption and emission spectra

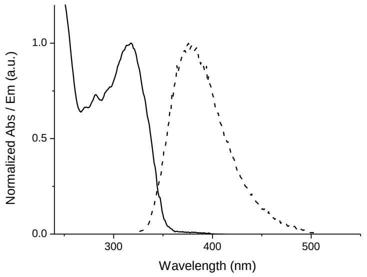

Fig. F.. Normalized absorption (full lines) and emission (dashed lines) spectra of **7a**.

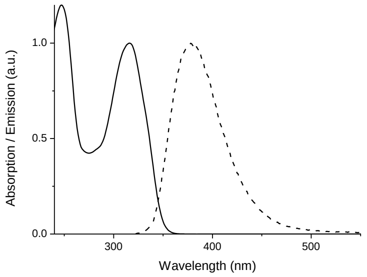

Fig. G. Normalized absorption (full lines) and emission (dashed lines) spectra of **7b**.

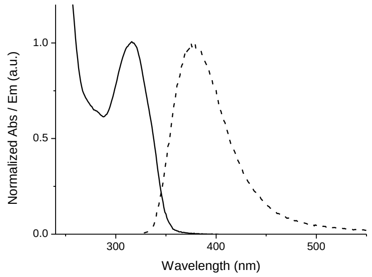

Fig. H. Normalized absorption (full lines) and emission (dashed lines) spectra of **7c**.

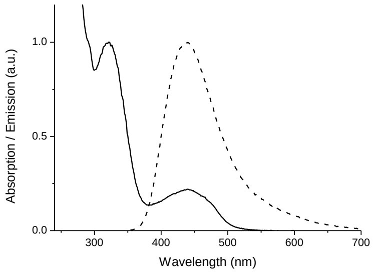

Fig. I. Normalized absorption (full lines) and emission (dashed lines) spectra of **8a**.

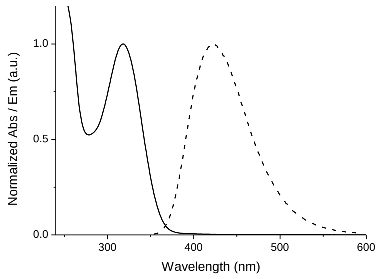

Fig. J. Normalized absorption (full lines) and emission (dashed lines) spectra of **8b**.

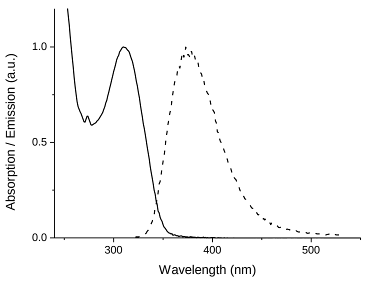

Fig. K. Normalized absorption (full lines) and emission (dashed lines) spectra of **14a**.

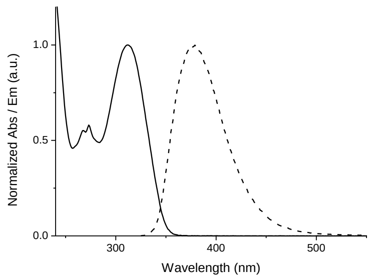

Fig. L. Normalized absorption (full lines) and emission (dashed lines) spectra of **14d**.

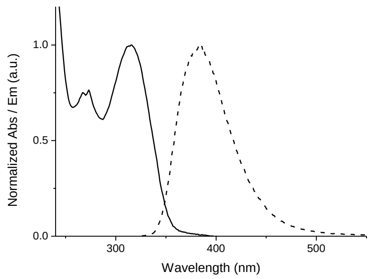

Fig. M. Normalized absorption (full lines) and emission (dashed lines) spectra of **14e**.

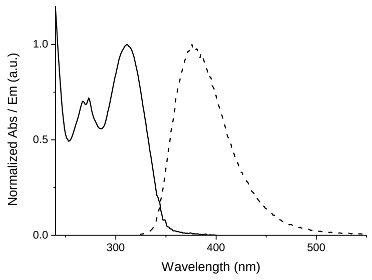

Fig. N. Normalized absorption (full lines) and emission (dashed lines) spectra of **14j**.

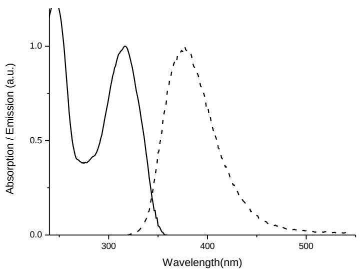

Fig. O. Normalized absorption (full lines) and emission (dashed lines) spectra of **14l**.

Fluorescence Polarisation (FP) Assay

The fluorescence polarisationpolarisation (FP) assay was performed in black 384-well microtiter plates at a final volume of 20 µL per well. Assay plates were prepared by addition of 20 µL of assay buffer (10 mM Tris (pH 8.0), 42.5 mM NaCl, and 0.0125% Tween-20) containing 1 µM GST-MDM2 (1 – 188) and Texas Red labelled wild-type p53 peptide (15 nM, amino acids 15 – 29: GSGSSQETFSDLWKLLPEN) to each well using a WellMate instrument (Matrix). For testing, a dilution series of small molecules (spanning 10 mM to 0.5 µM in DMSO, in 1:3-dilution) was added by direct addition to the assay plate by Biomek FX lab automation workstation (Beckman Coulter, Inc., Fullerton, CA) using pin transfer (100ss pins, V&P scientific) giving a test dilution series spanning 130 µM to 6.6 nM with a final concentration of 1.3 % DMSO. The assay mixture was incubated for 1 hour at room temperature and the fluorescence polarisation signal was measured on an EnVision multilabel plate reader fitted with a 555-nm excitation filter, 632-nm static and polarized filters, and a Texas Red FP dichroic mirror. Unlabelled WT-p53 peptide was used as a positive control, and DMSO was used as a negative control. Technical triplicate data was normalized to the positive (100% inhibition) and negative (0% inhibition) controls on the corresponding row of the 384-well plate (the percentage inhibition = 100 × (sample result – negative control)/(positive control mean – negative control)). Two to seven independent experiments of normalized data were combined into a data set, and then fit using a non-linear regression in GraphPad Prism with the formula log(inhibitor) vs. response – Variable slope (four parameters). Additionally the residuals of the curve fit were plotted to determine the fit of the theoretical curve. IC50 and 95% confidence intervals (CI) were determined from these graphs.

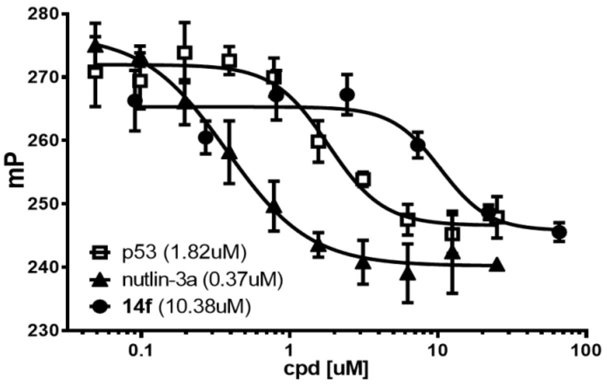

Fig. P. Example of fluorescence polarisation-based MDM2 binding assay. Inhibition curves for Nutlin-3a (▲) the natural p53 peptide (□) which was used as positive controls and for **14f** (●).

WaterLOGSY measurements

All NMR experiments were performed at 298 K (25 °C) using a Bruker Avance 600 MHz spectrometer equipped with TCI cryogenic gradient probe. Compounds (20 mM stock solution in DMSO-D_6) were added to buffer (25mM HEPES, pH7.2, 150 mM NaCl, 2mM TCEP) or buffer containing MDM2 protein (5 µM) to give final compound concentration of 200 µM. One-dimensional (1D) ^{1}H- and WaterLOGSY (water-ligand observed via gradient spectroscopy)[8] NMR spectra were recorded for compounds, protein, and compounds plus protein, respectively. NMR spectra were processed using Bruker Topspin software.

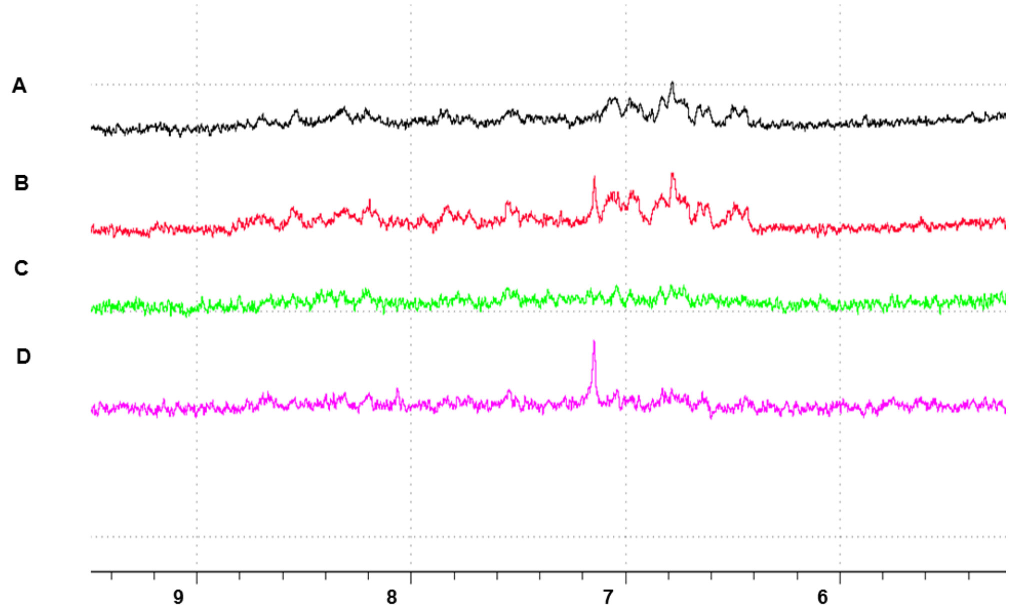

A. Black: protein, no compound – ^{1}H
B. Red: protein + **14f** – ^{1}H
C. Green: protein, no compound – WaterLOGSY
D. Magenta: protein + **14f** – WaterLOGSY

Fig. Q. WaterLOGSY experiments. 1D spectra and WaterLOGSY spectra of the MDM2 protein in the absence of the compound (A and C) and in the presence of **14f** (B and D). The positive signal around 7.2 ppm of the compound on spectra D demonstrates an interaction between **14f** and the MDM2 protein.

Solubility

Solubility assay was carried out on Biomek FX lab automation workstation (Beckman Coulter, Inc., Fullerton, CA) using µSOL Evolution software (pION Inc., Woburn, MA). The detailed method is described as following. 10 µL of 10 mM compound stock (in DMSO) was added to 190 µL 1-propanol to make a reference stock plate. 5 µL from this reference stock plate was mixed with 70 µL 1-propanol and 75 µL citrate phosphate buffered saline (isotonic) to make the reference plate, and the UV spectrum (250 nm – 500 nm) of the reference plate was read. 6 µL of 10 mM test compound stock was added to 594 µL buffer in a 96-well storage plate and mixed. The storage plate was sealed and incubated at room temperature for 18 hours. The suspension was then filtered through a 96-well filter plate (pION Inc., Woburn, MA). 75 µL filtrate was mixed with 75 µL 1-propanol to make the sample plate, and the UV spectrum of the sample plate was read. Calculation was carried out by µSOL Evolution

software based on the AUC (area under curve) of UV spectrum of the sample plate and the reference plate. All compounds were tested in triplicates.

Molecular modelling

Conformational analysis was performed using MacroModel[9] in Maestro. The conformational search was performed using the OPLS-2005 force field with water as solvation model. The energy window for saving structures was set to 21 kJ/mol and a maximum atom deviation threshold of 0.5 Å. The torsional sampling mode (MCMM) was employed with 10000 steps per rotable bond. PRCG was used as the minimization method with a maximum of 10000 iterations. Protein crystal complexes were retrieved from the PDB database and prepared with the protein preparation wizard workflow in Maestro using default settings (minimization with OPLS2005 as forcefield converging heavy atoms to RMSD of 0.30Å)[10]. Grids for docking were prepared using Glide[11] in maestro. Grids were centred around the co-crystallized ligands with a box size of 20 Å. Prior to docking, all ligands were prepared using Ligprep[12] in maestro with OPLS_2005 as forcefield. Epik was used to generate possible ionization states at pH 7 ±2. Docking was performed in Glide with flexible ligands and added Epik penalties. Docking of structures generated by Ligprep was performed in Precision (XP) mode. Crystal complexes were compared using the protein structure alignment in Maestro. Alignments were made with protein backbone atoms and with all heavy atoms.

^{1}H and ^{13}C NMR spectra

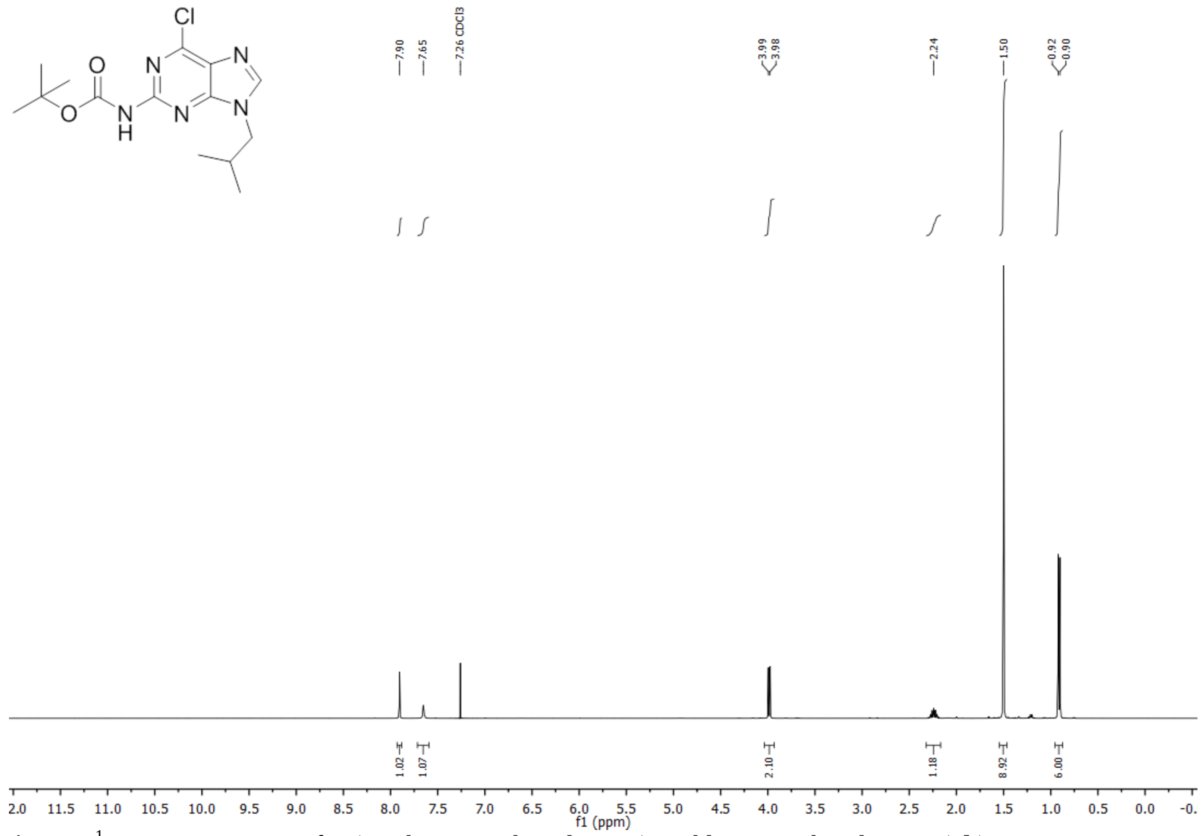

Fig. A1. ^{1}H NMR spectrum of 2-(*tert*-butoxycarbonylamino)-6-chloro-9-isobutylpurine (**2b**).

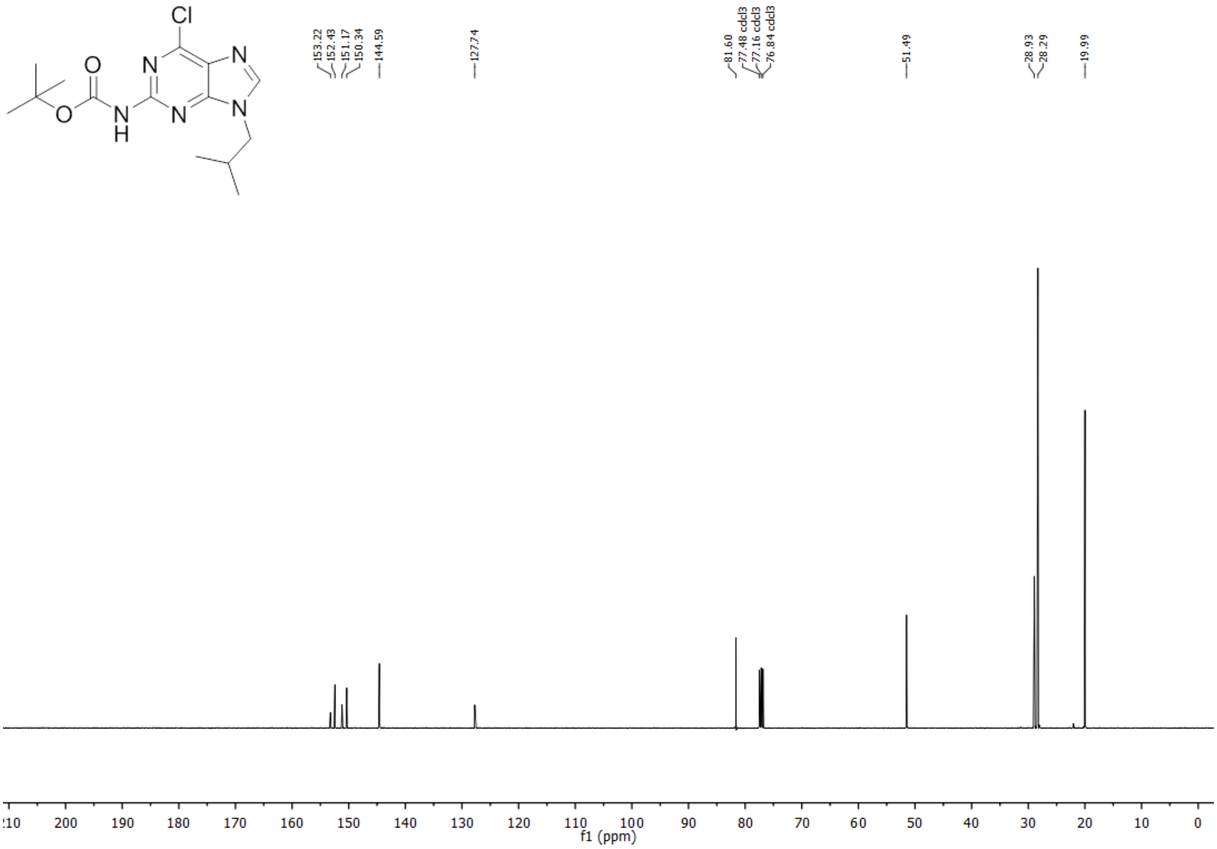

Fig. A2. ^{13}C NMR spectrum of 2-(*tert*-butoxycarbonylamino)-6-chloro-9-isobutylpurine (**2b**).

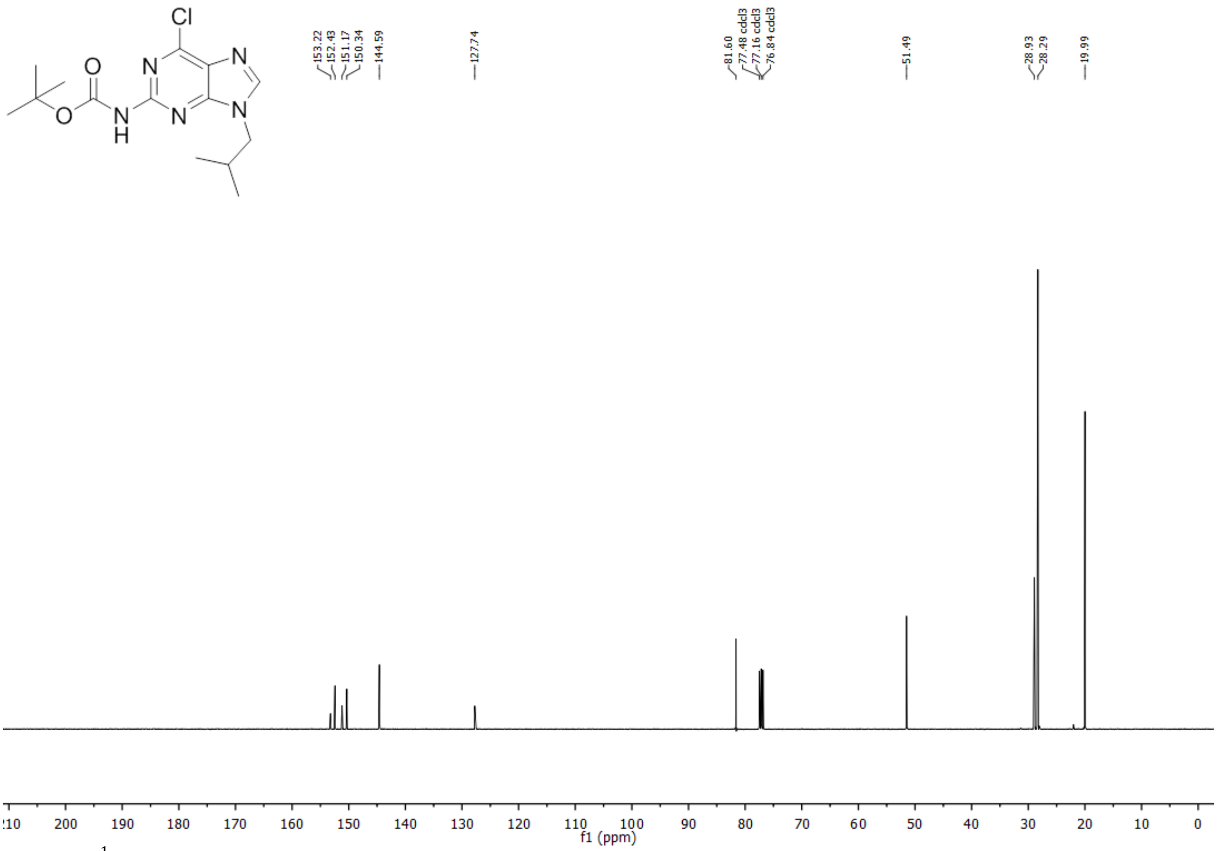

Fig. A3. ^{1}H NMR spectrum of 2-(*tert*-butoxycarbonylamino)-6-chloro-9-cyclophentylmethylpurine (**2b**).

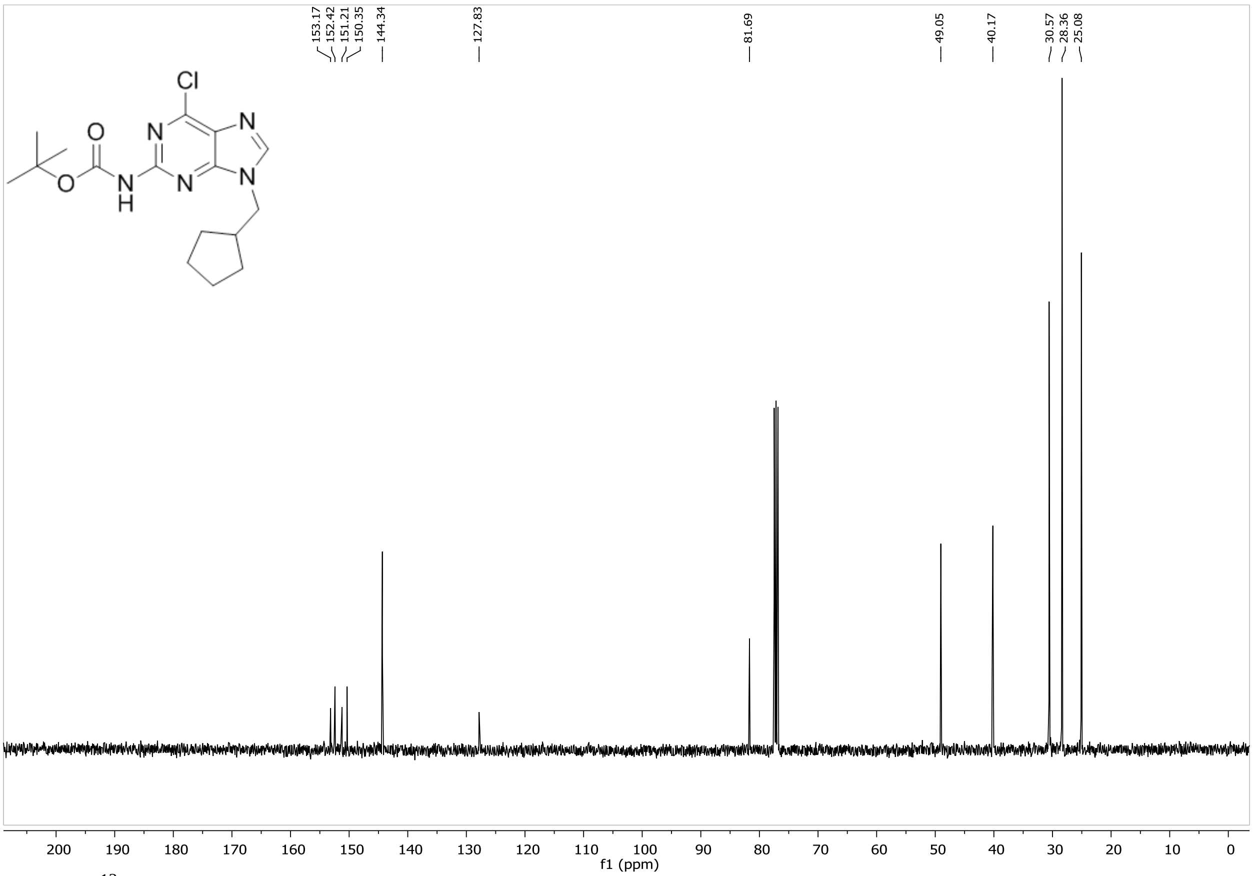

Fig. A4. ^{13}C NMR spectrum of 2-(*tert*-butoxycarbonylamino)-6-chloro-9-cyclophentylmethylpurine (**2c**).

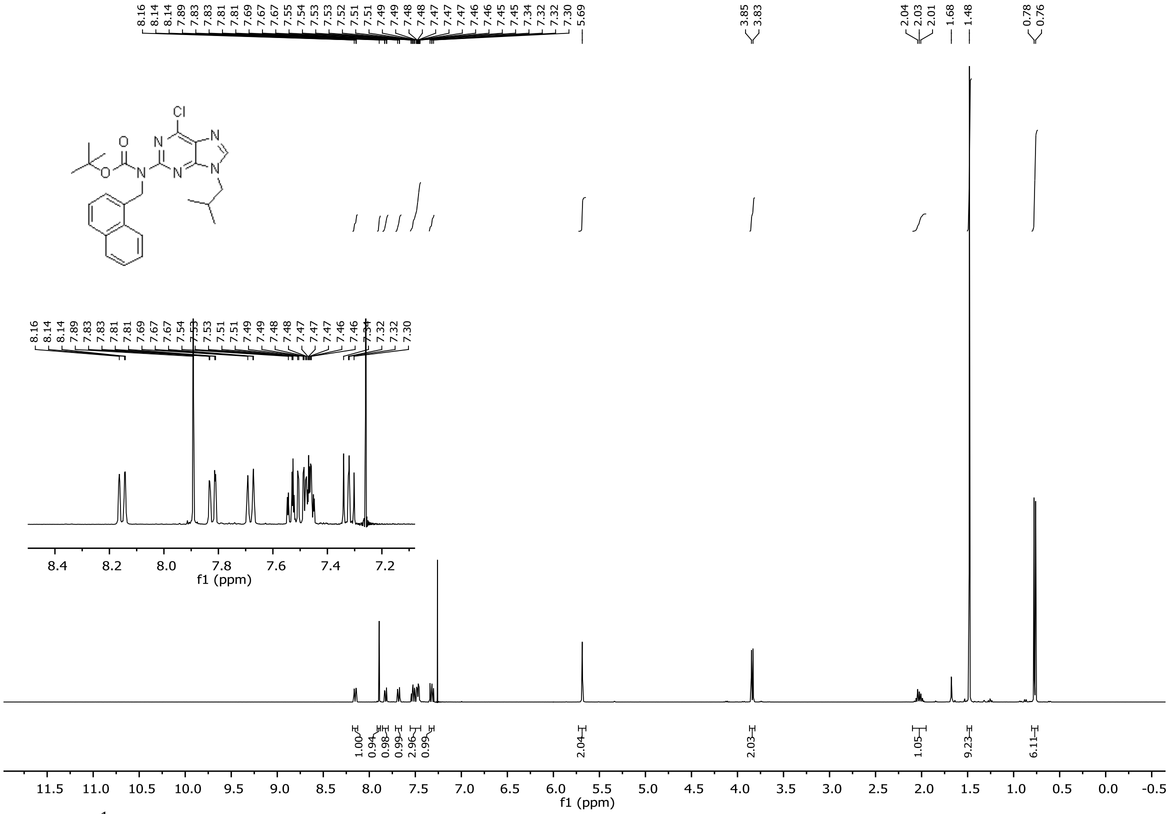

Fig. A5. ^{1}H NMR spectrum of 6-chloro-2-(N-napthenyl-*tert*-butoxycarbonylamino)-9-isobutylpurine (**3b**).

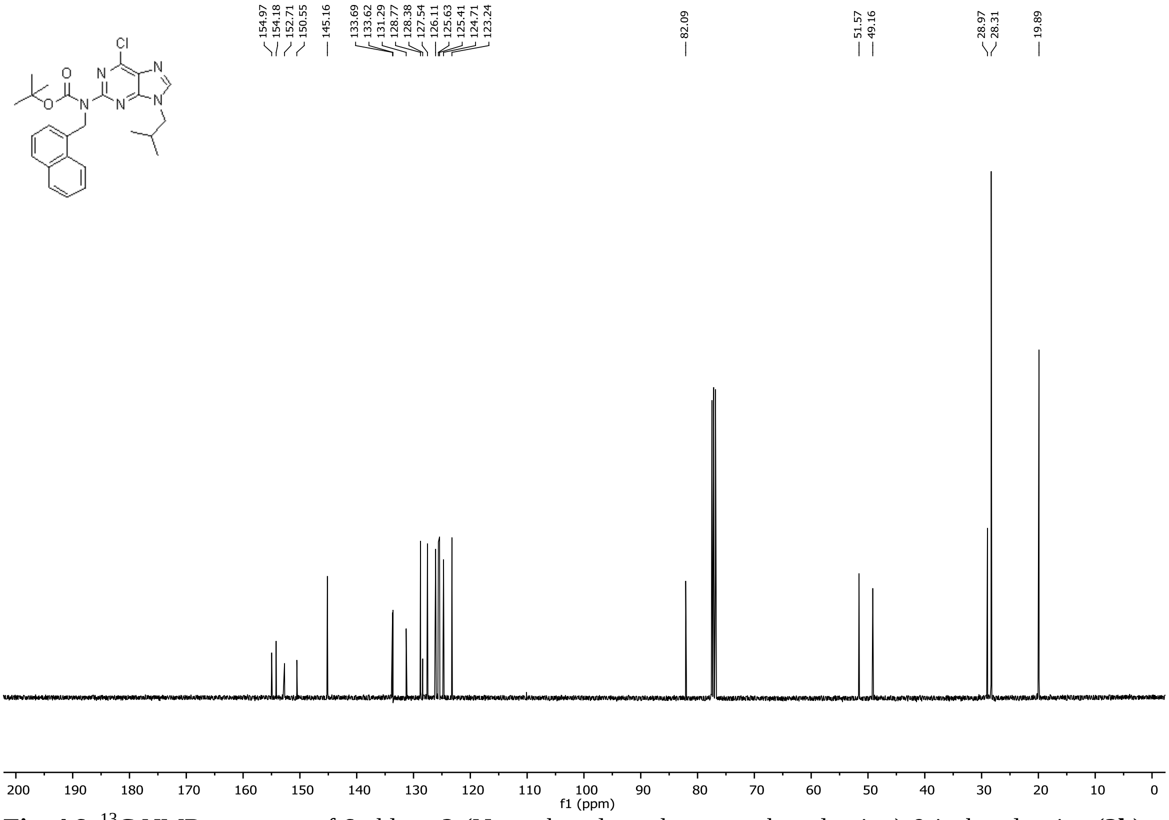

Fig. A6. ^{13}C NMR spectrum of 6-chloro-2-(N-napthenyl-*tert*-butoxycarbonylamino)-9-isobutylpurine (**3b**).

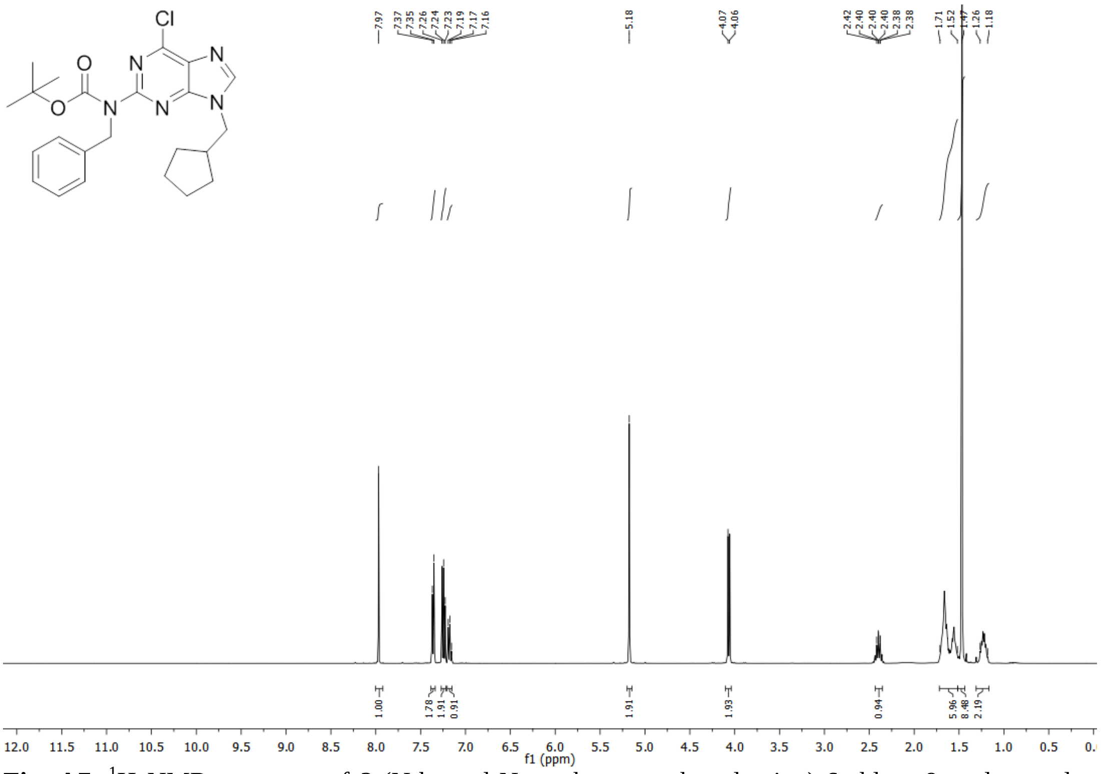

Fig. A7. ^{1}H NMR spectrum of 2-(N-benzyl-N-*tert*-butoxycarbonylamino)-6-chloro-9-cyclopentylmethylpurine (**3c**).

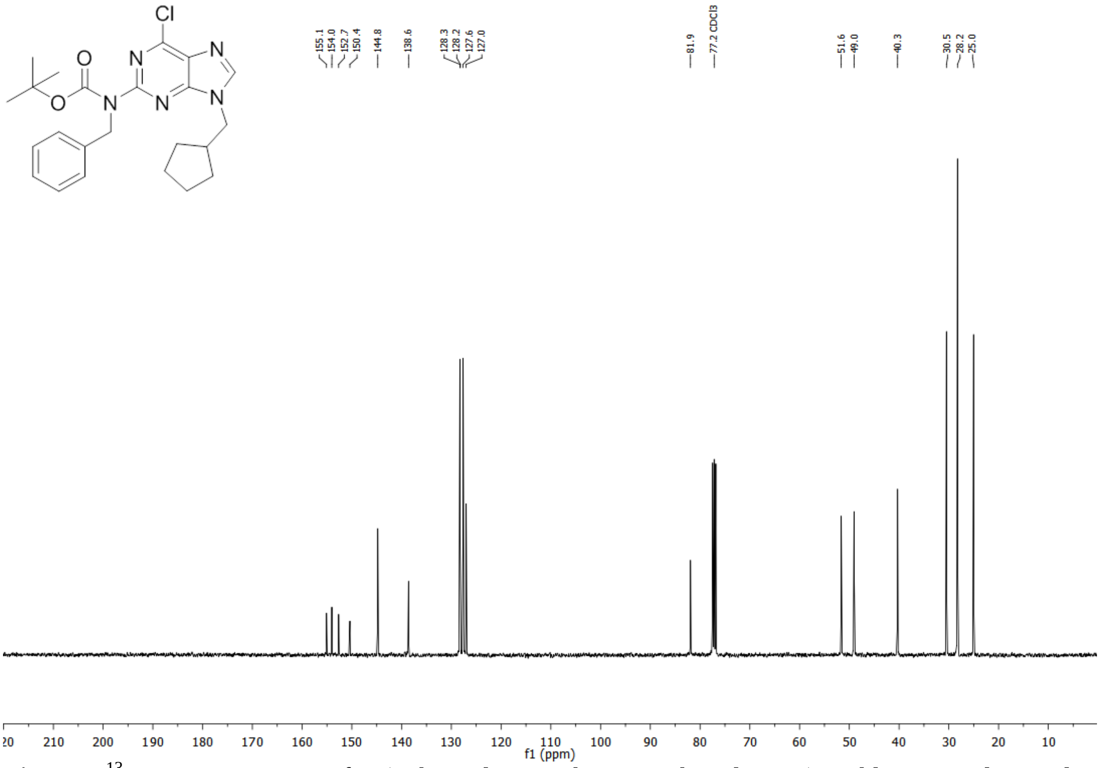

Fig. A8. ^{13}C NMR spectrum of 2-(N-benzyl-N-*tert*-butoxycarbonylamino)-6-chloro-9-cyclopentylmethylpurine (**3c**).

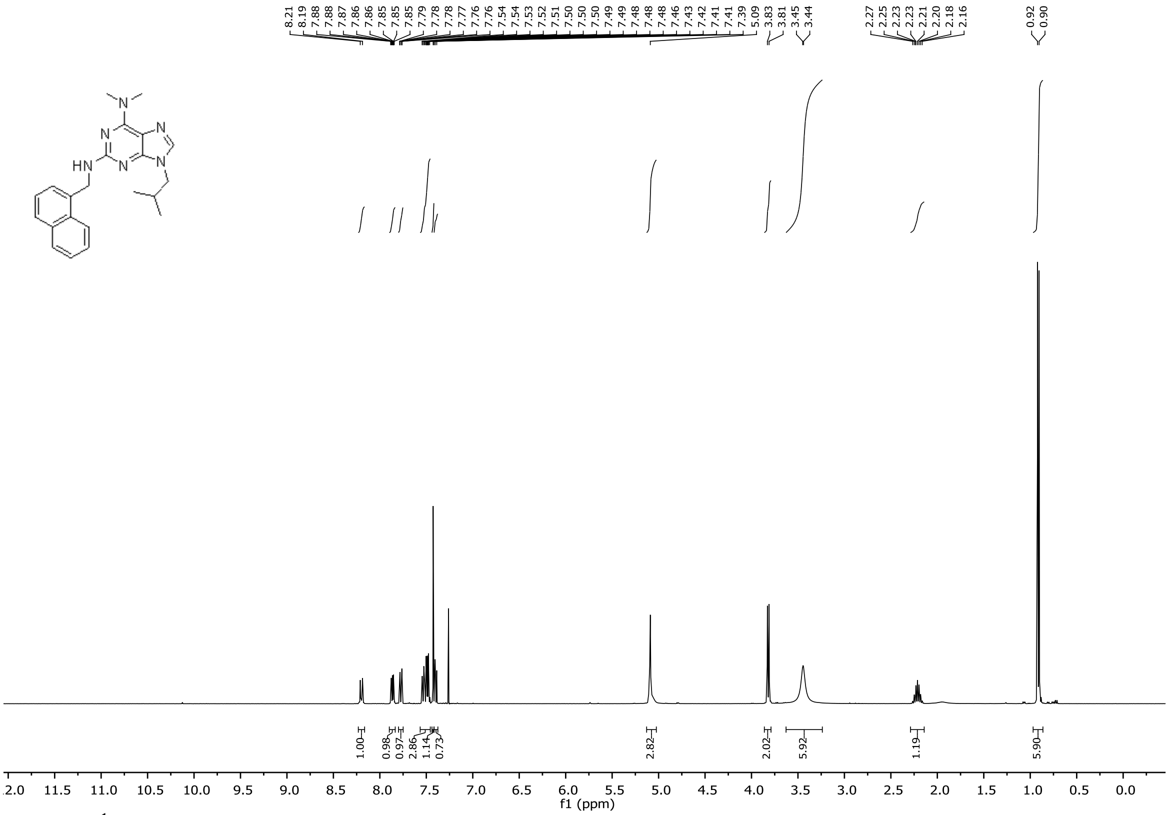

Fig. A9. ^{1}H NMR spectrum of 6-dimethylamino-9-isobutyl-2-naphtalenylaminopurine (**4b**).

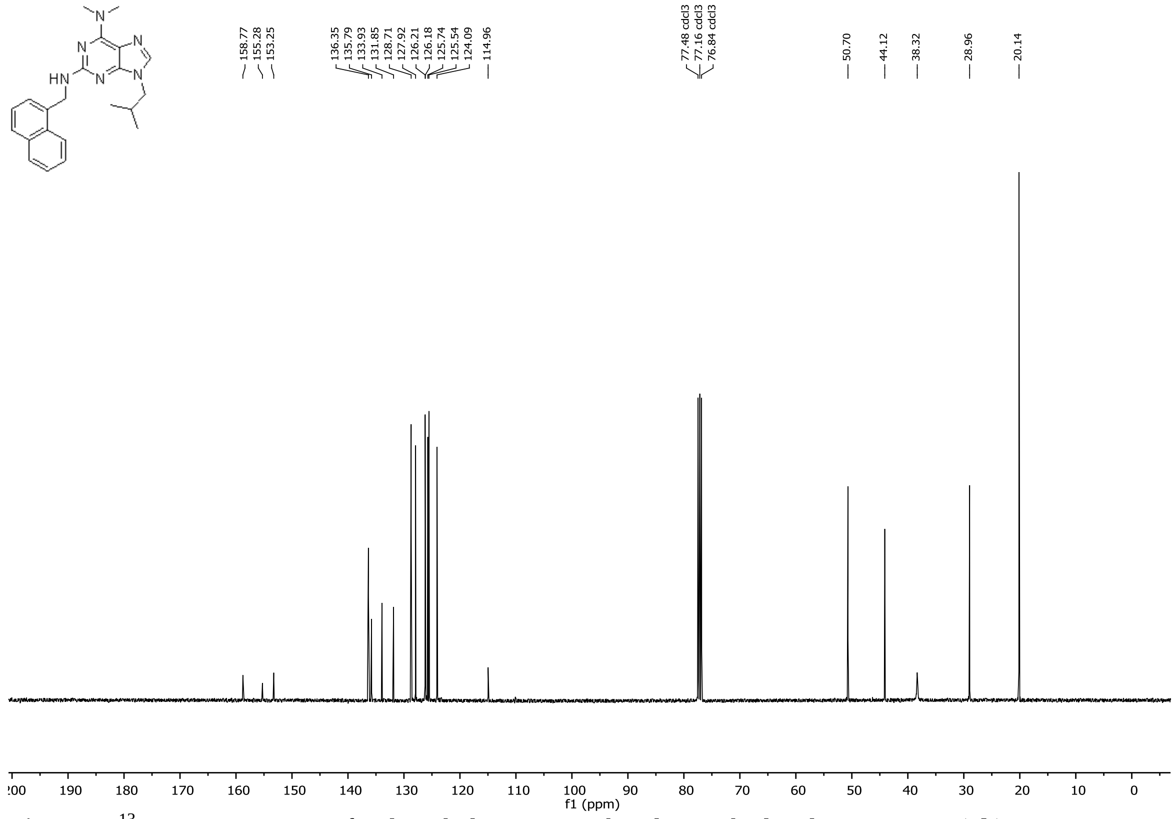

Fig. A10. ^{13}C NMR spectrum of 6-dimethylamino-9-isobutyl-2-naphtalenylaminopurine (**4b**).

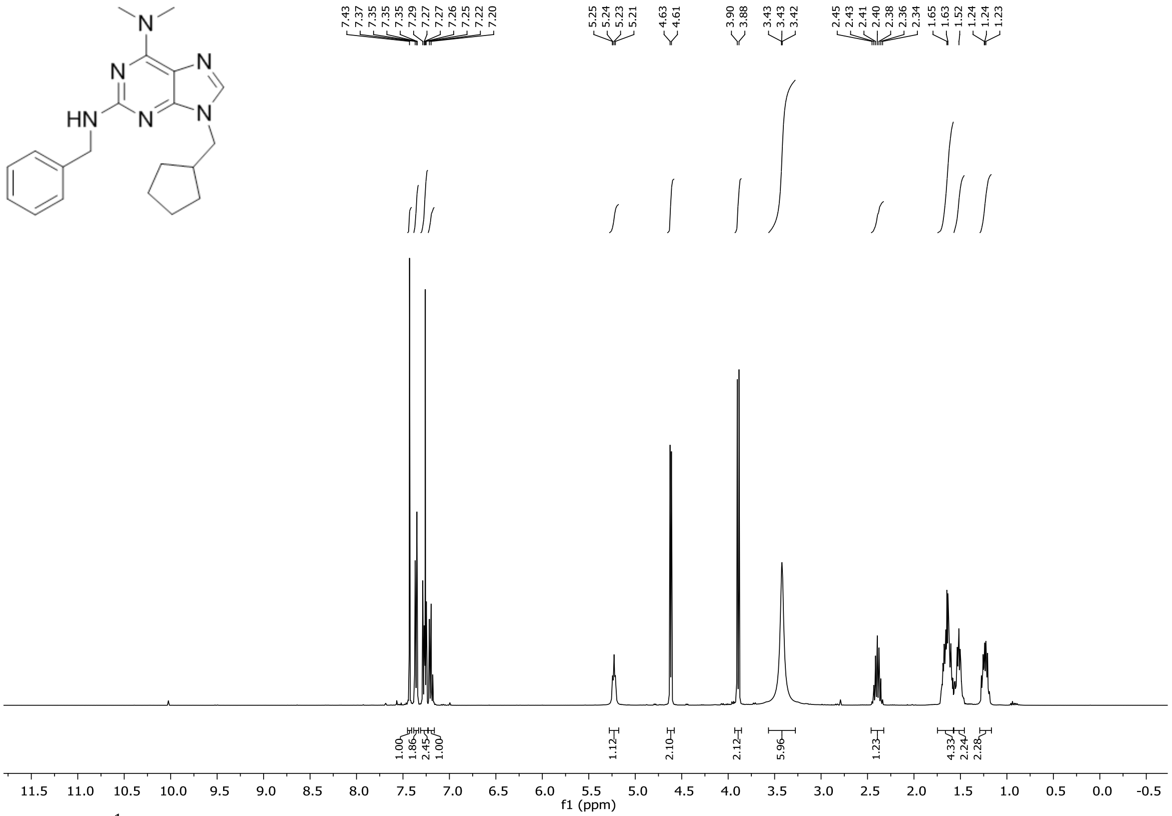

Fig. A11. ^{1}H NMR spectrum of 2-benzylamino-9-cyclophentylmethyl-6-dimethylamino-purine (**4c**)

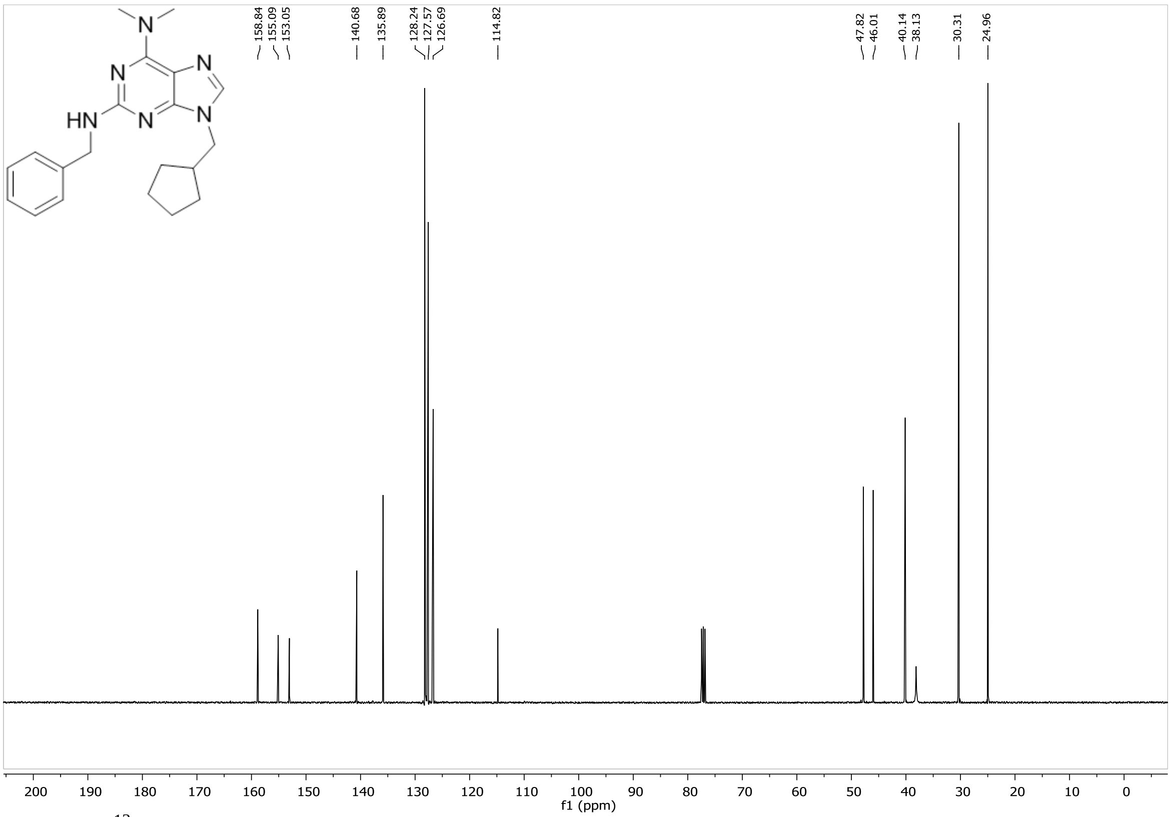

Fig. A12. ^{13}C NMR spectrum of 2-benzylamino-9-cyclophentylmethyl-6-dimethylamino-purine (**4c**)

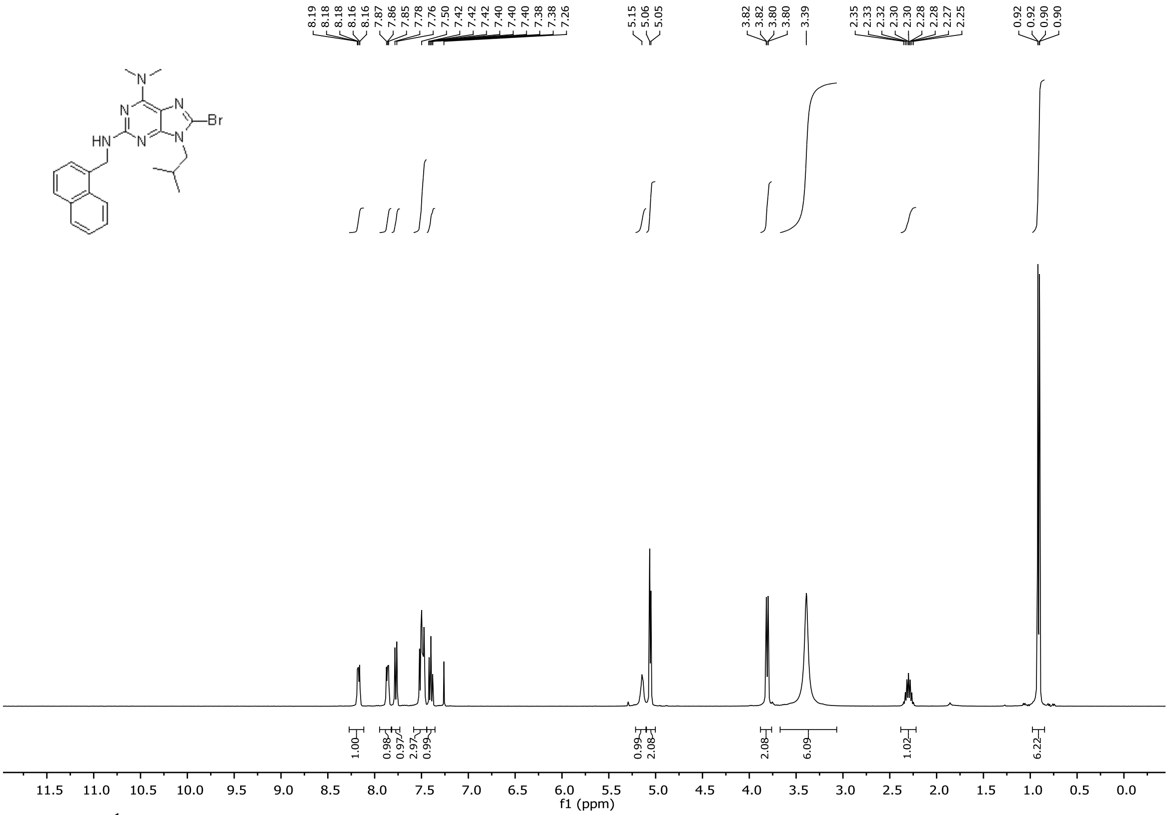

Fig. A13. ^{1}H NMR spectrum of 8-bromo-6-dimethylamino-9-isobutyl-6-naphtalenylaminopurine (**5b**).

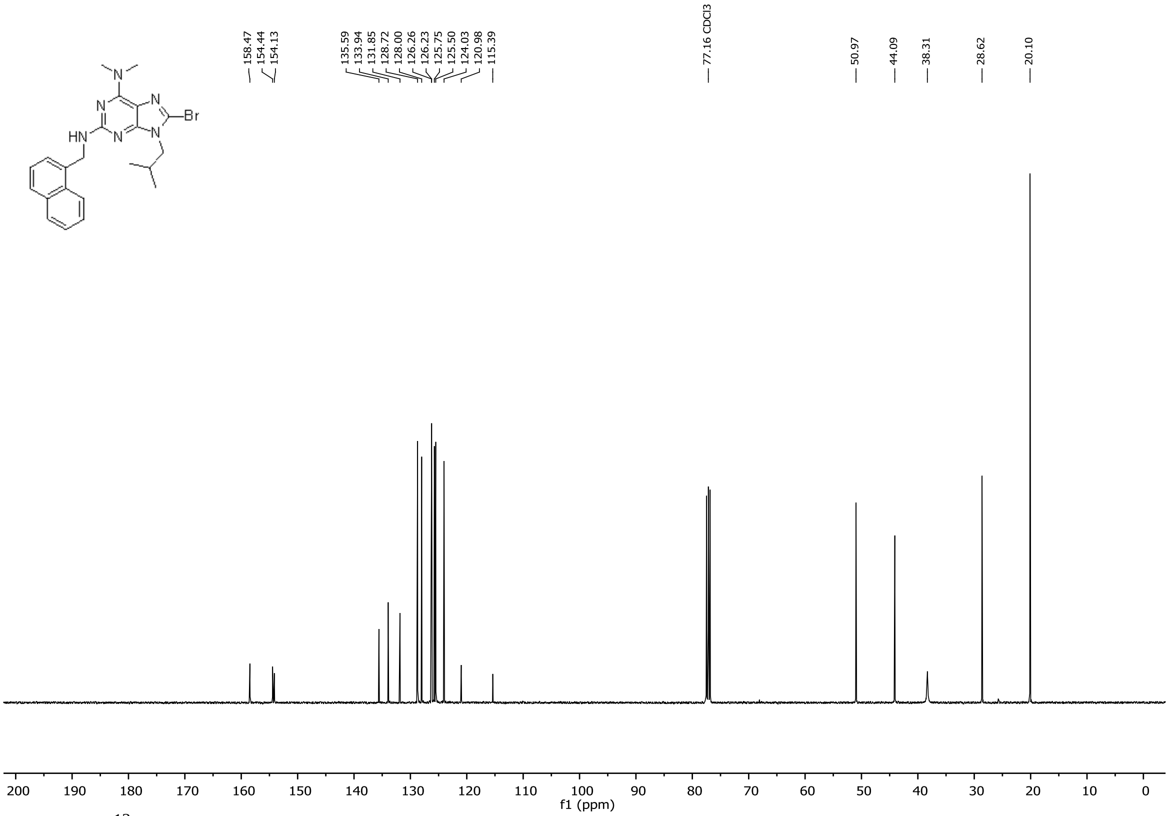

Fig. A14. ^{13}C NMR spectrum of 8-bromo-6-dimethylamino-9-isobutyl-6-naphtalenylaminopurine (**5b**).

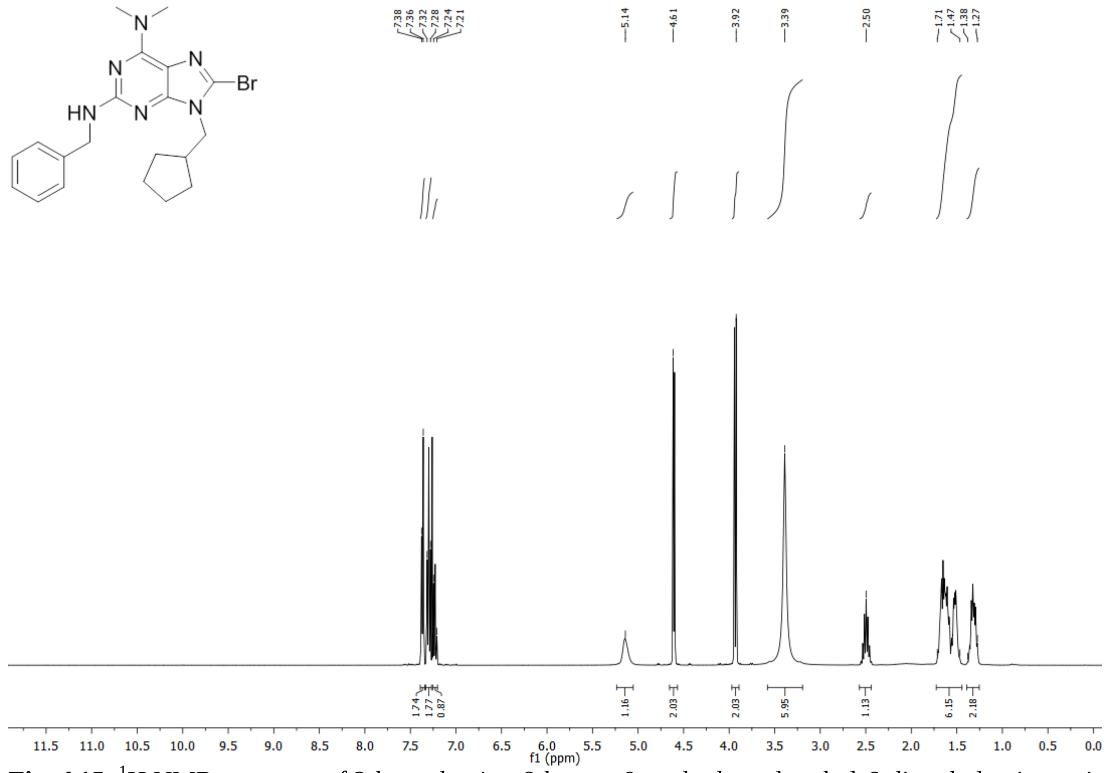

Fig. A15. ^{1}H NMR spectrum of 2-benzylamino-8-bromo-9-cyclophentylmethyl-6-dimethylaminopurine (**5c**).

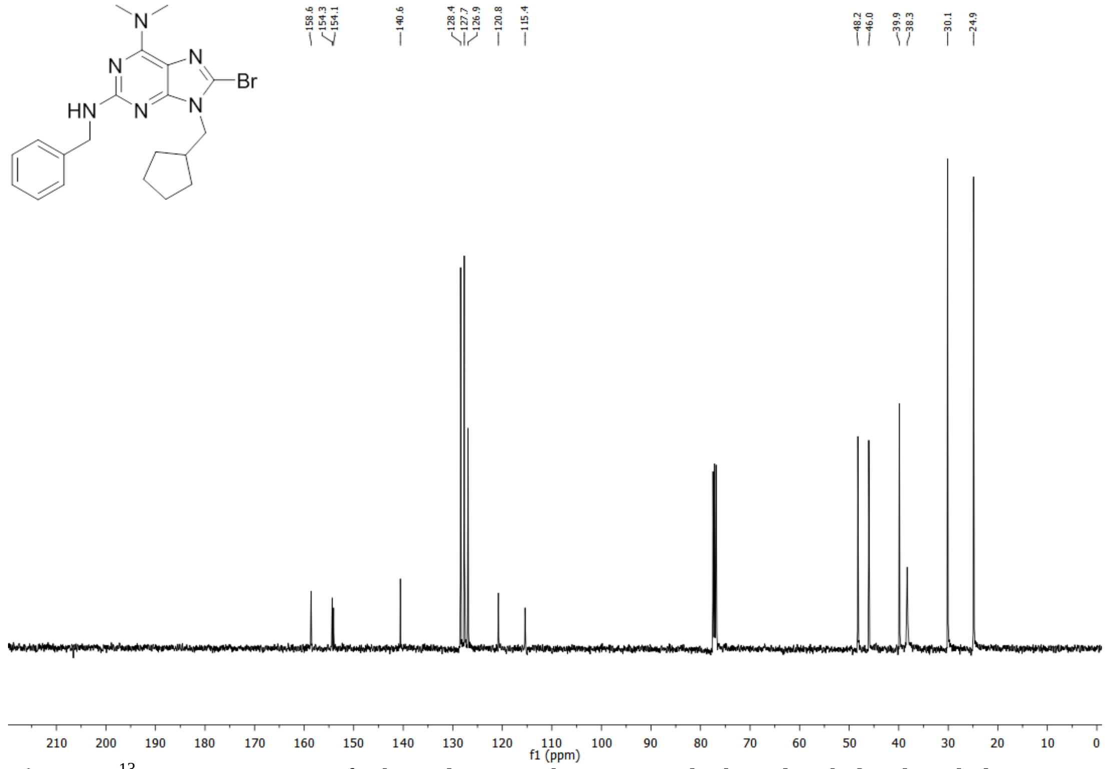

Fig. A16. ^{13}C NMR spectrum of 2-benzylamino-8-bromo-9-cyclophentylmethyl-6-dimethylaminopurine (**5c**).

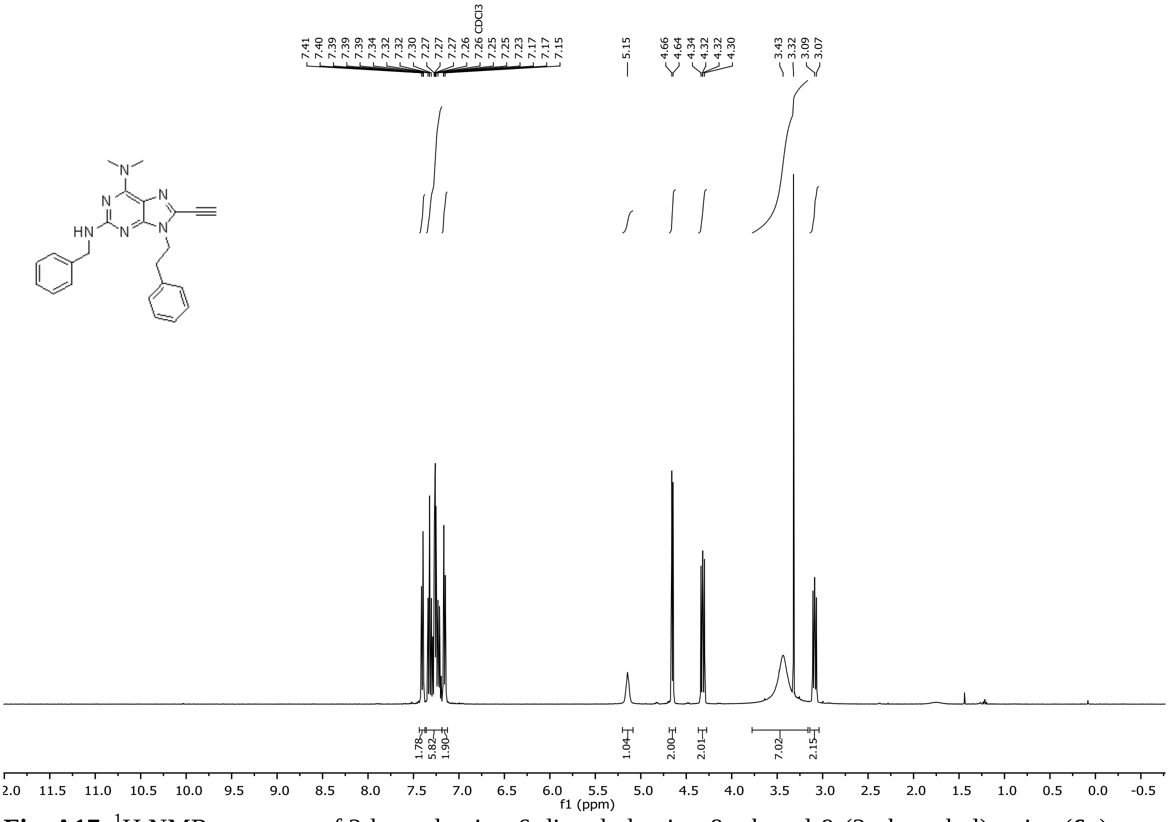

Fig. A17. ^{1}H NMR spectrum of 2-benzylamino-6-dimethylamino-8-ethynyl-9-(2-phenethyl)purine (**6a**).

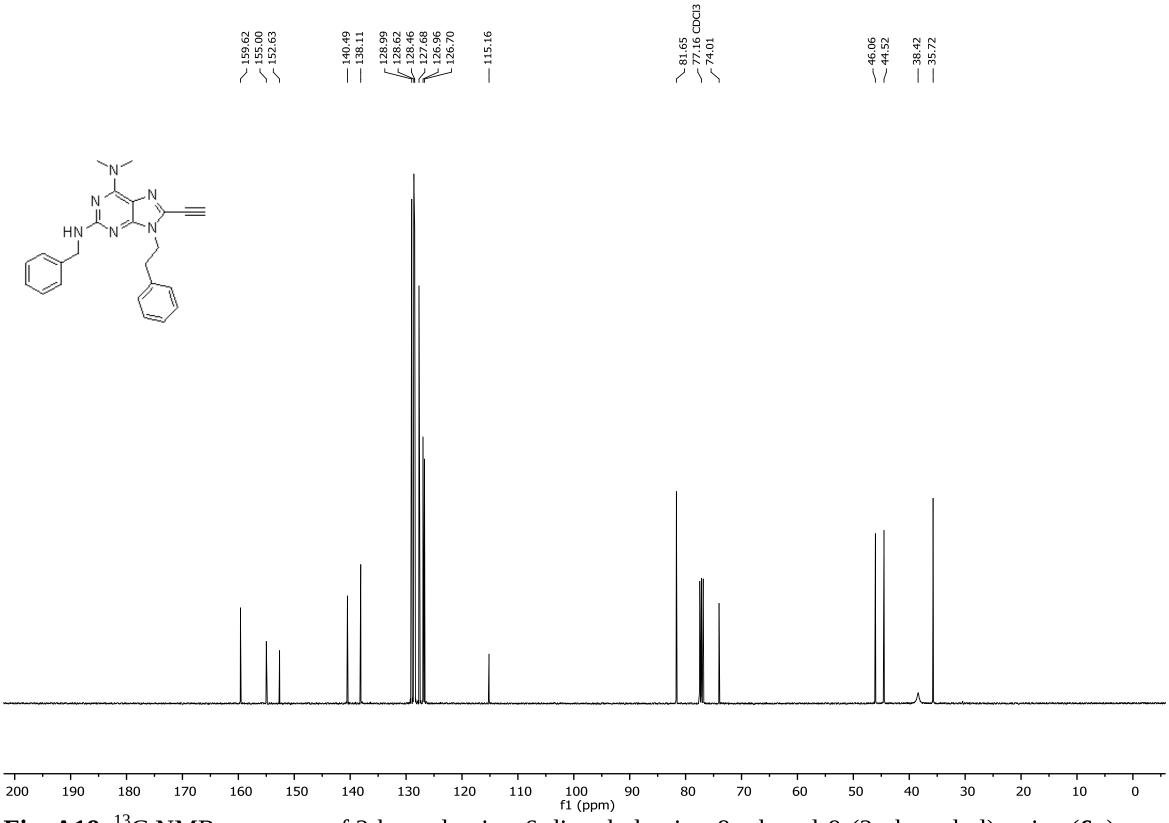

Fig. A18. ^{13}C NMR spectrum of 2-benzylamino-6-dimethylamino-8-ethynyl-9-(2-phenethyl)purine (**6a**).

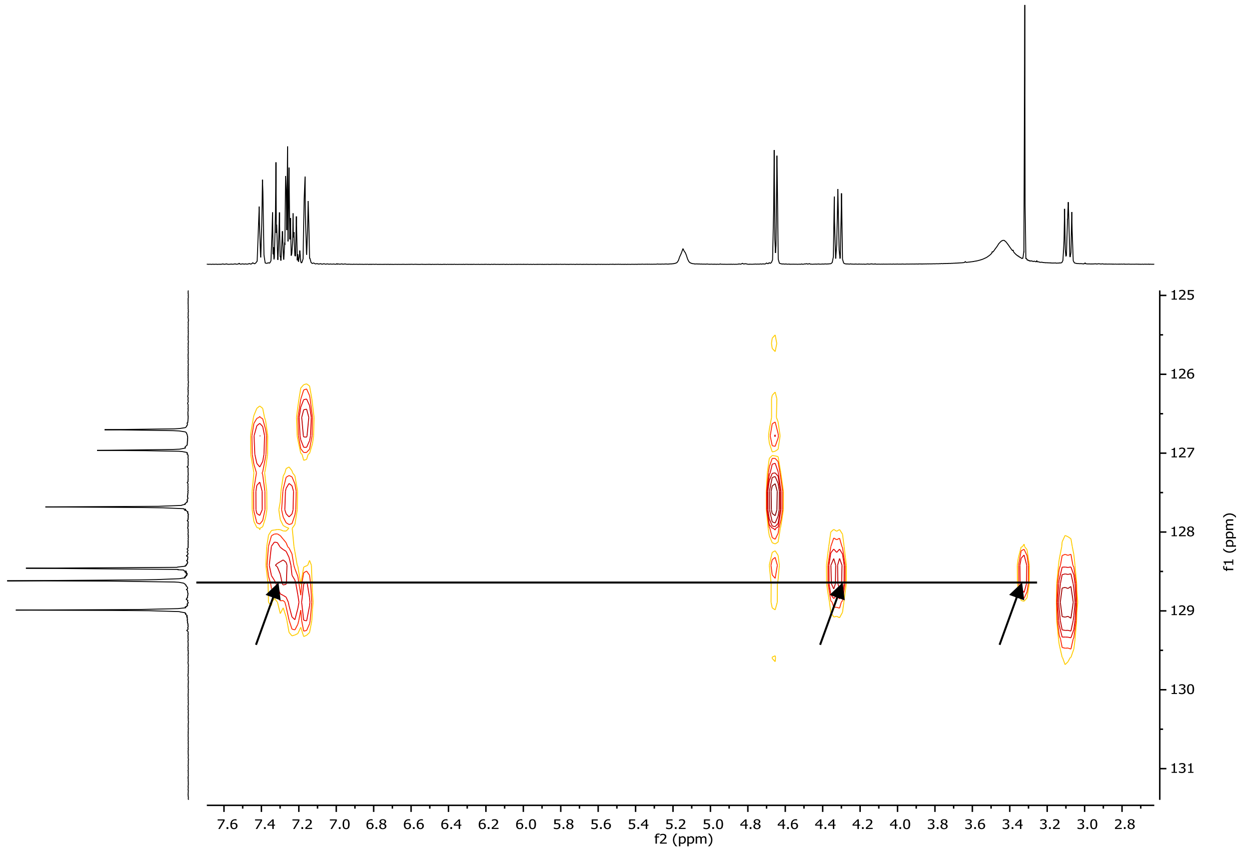

Fig. A19. Detail from gHMBC for **6a**.

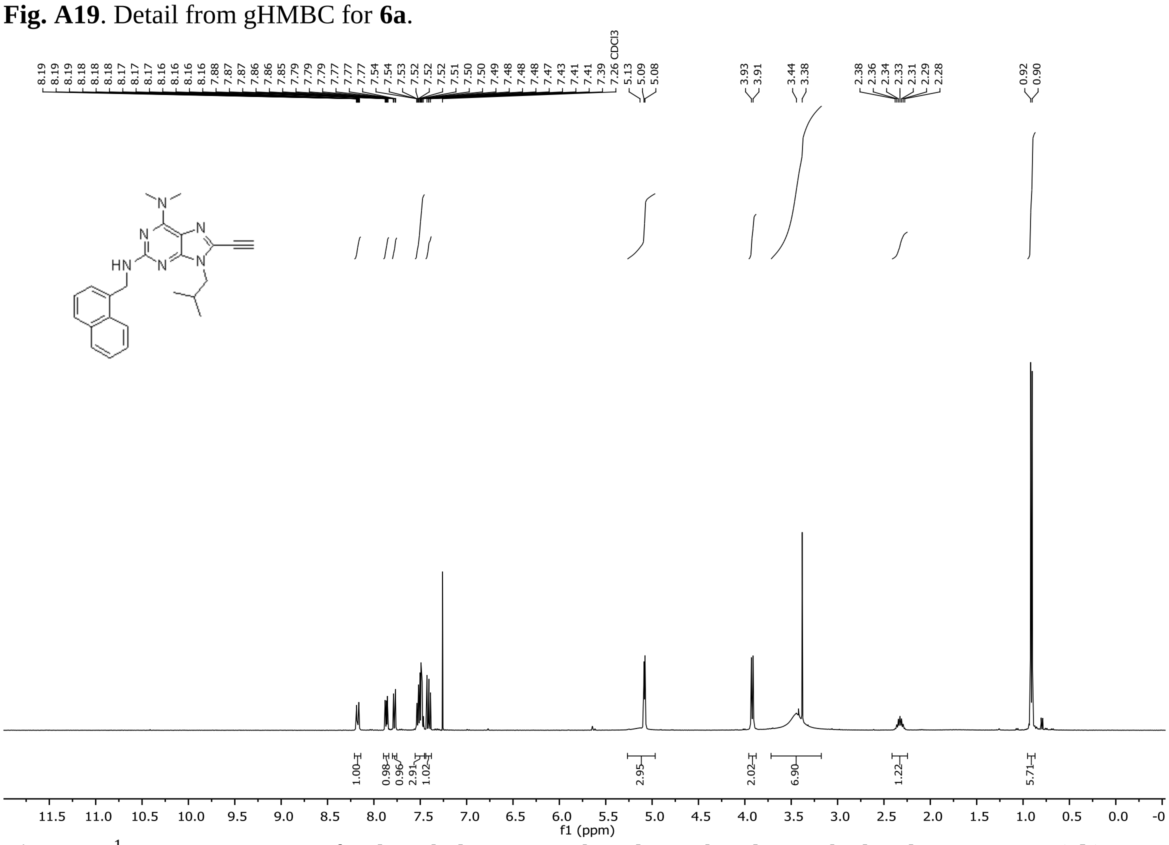

Fig. A20. ^{1}H NMR spectrum of 6-dimethylamino-8-ethynyl-9-isobutyl-6-naphtalenylaminopurine (**6b**).

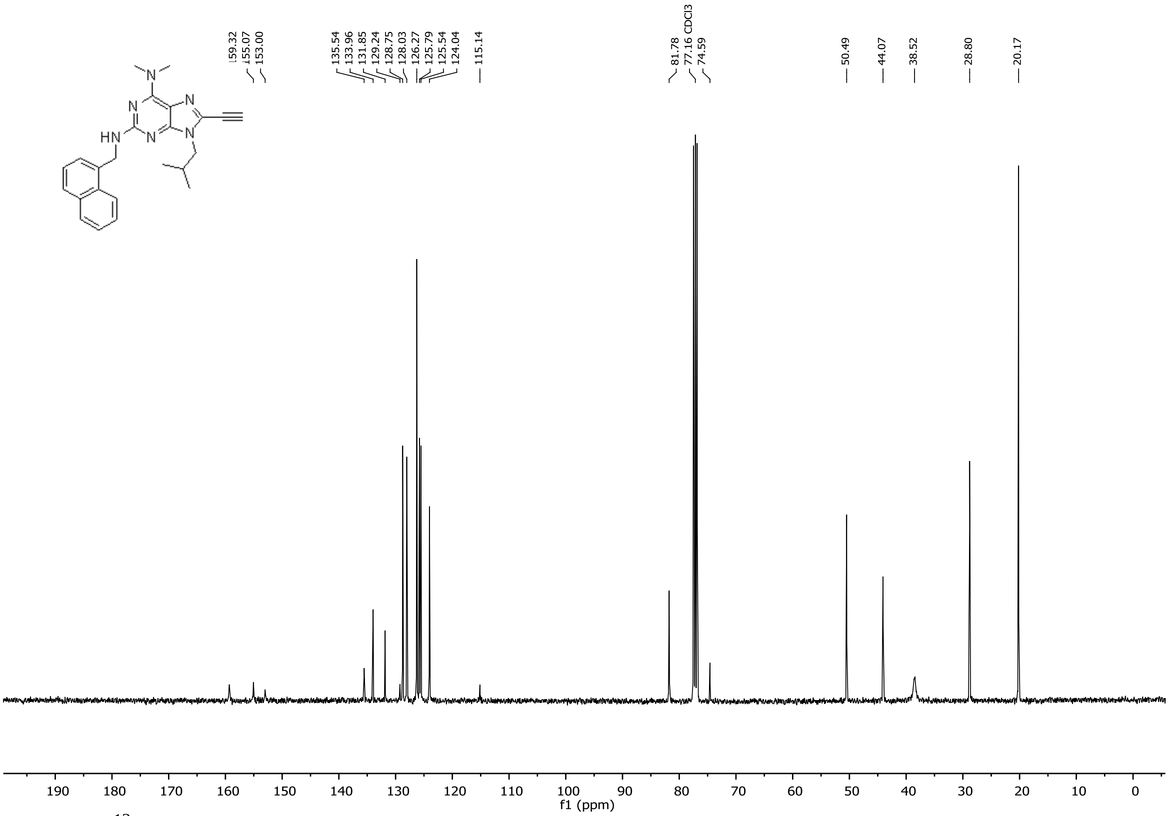

Fig. A21. ^{13}C NMR spectrum of 6-dimethylamino-8-ethynyl-9-isobutyl-6-naphtalenylaminopurine (**6b**).

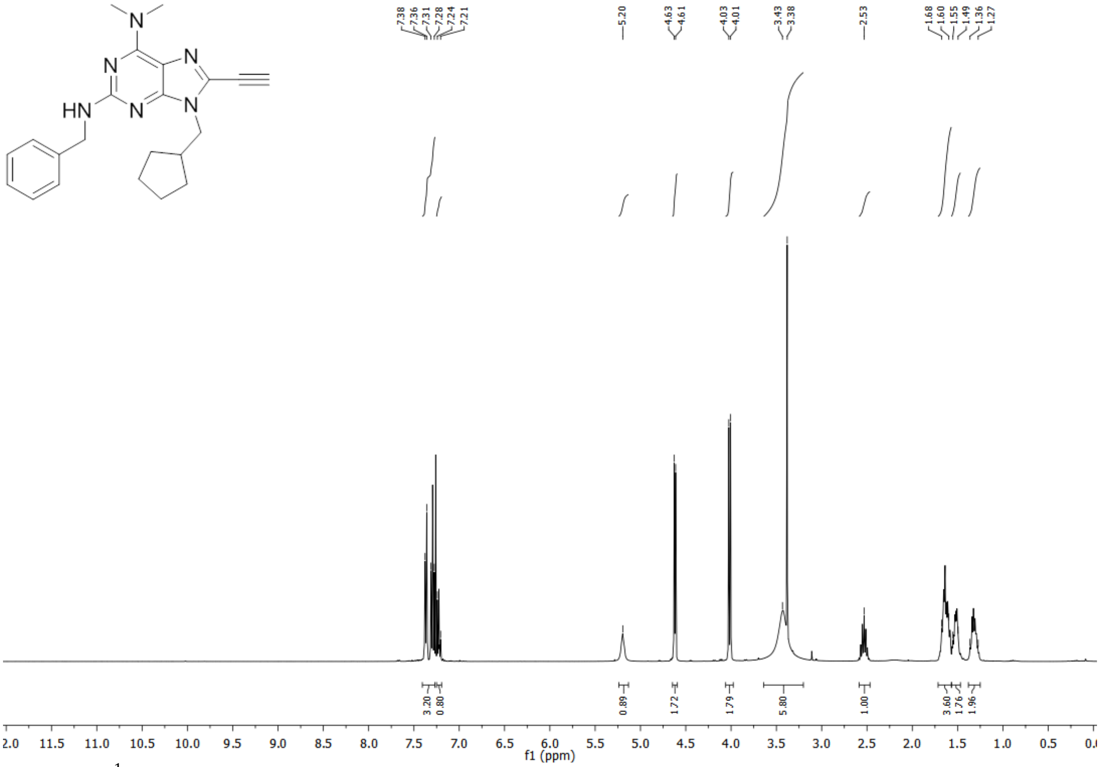

Fig. A22. ^{1}H NMR spectrum of 2-benzylamino-6-dimethylamino-9-cyclopentylmethyl-8-ethynylpurine (**6c**).

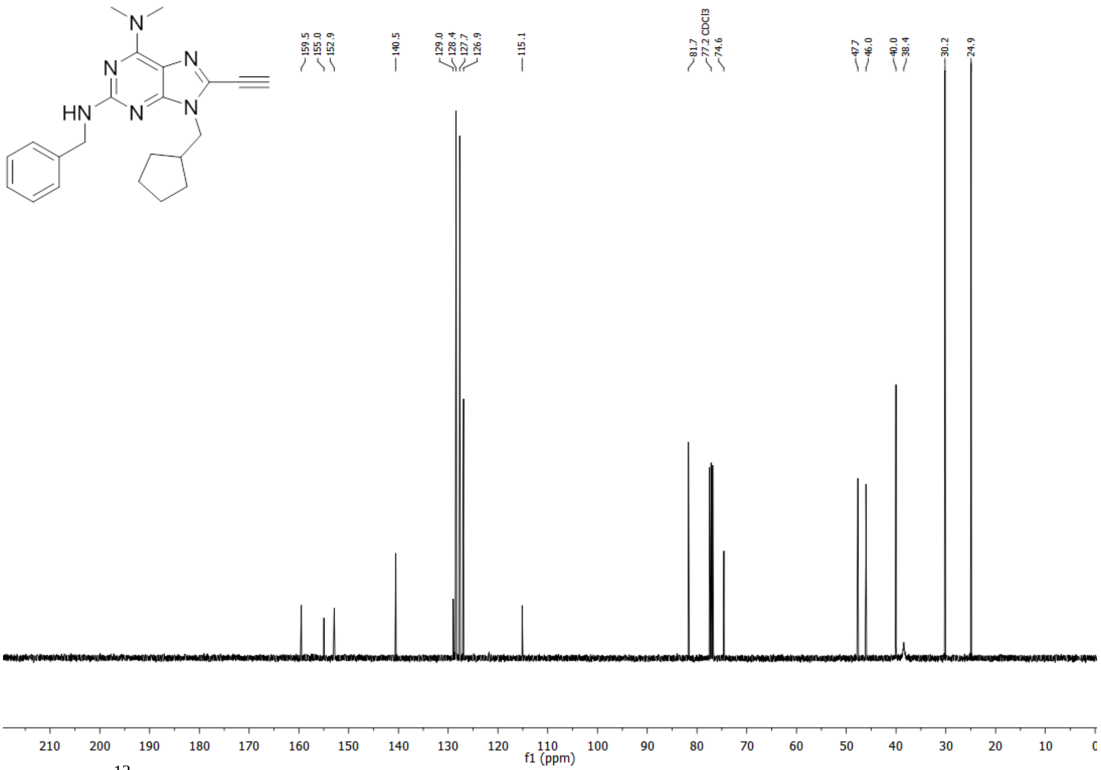

Fig. A23. ^{13}C NMR spectrum of 2-benzylamino-6-dimethylamino-9-cyclopentylmethyl-8-ethynylpurine (**6c**).

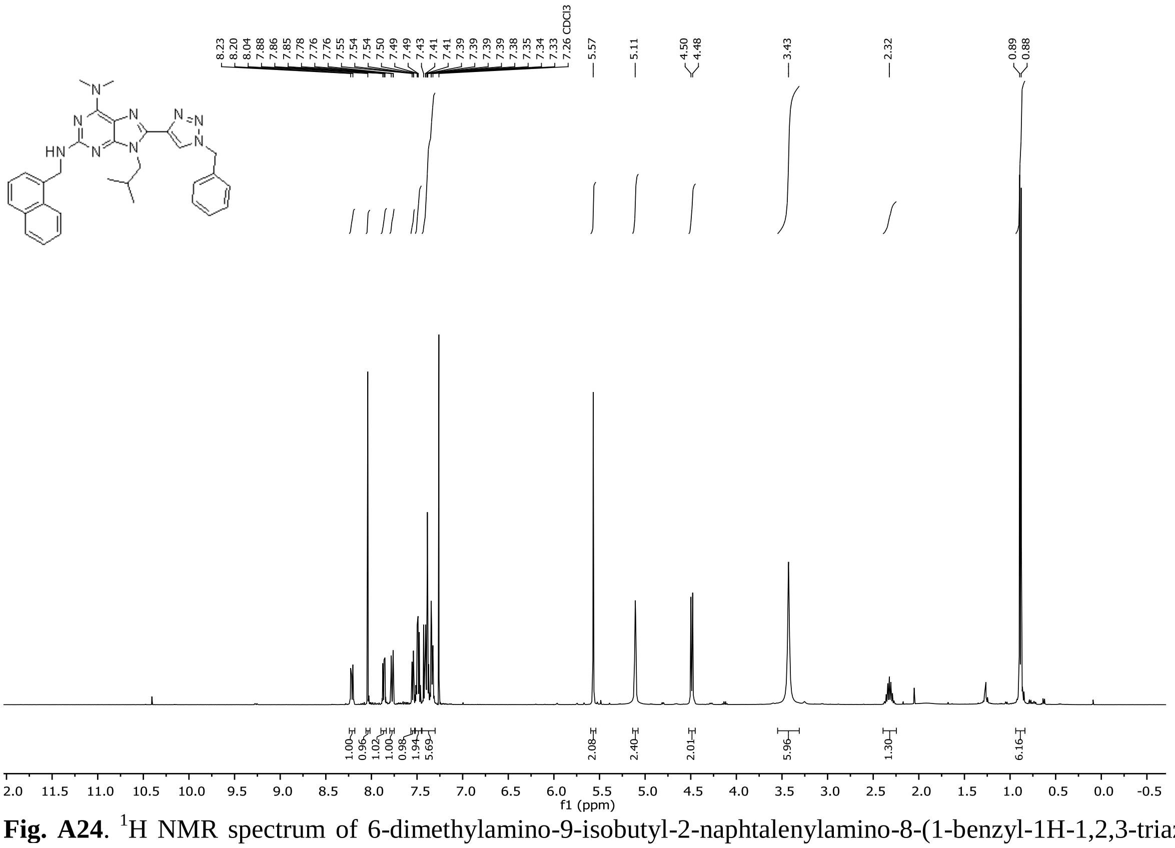

Fig. A24. ^{1}H NMR spectrum of 6-dimethylamino-9-isobutyl-2-naphtalenylamino-8-(1-benzyl-1H-1,2,3-triazol-4-yl)purine (**7a**).

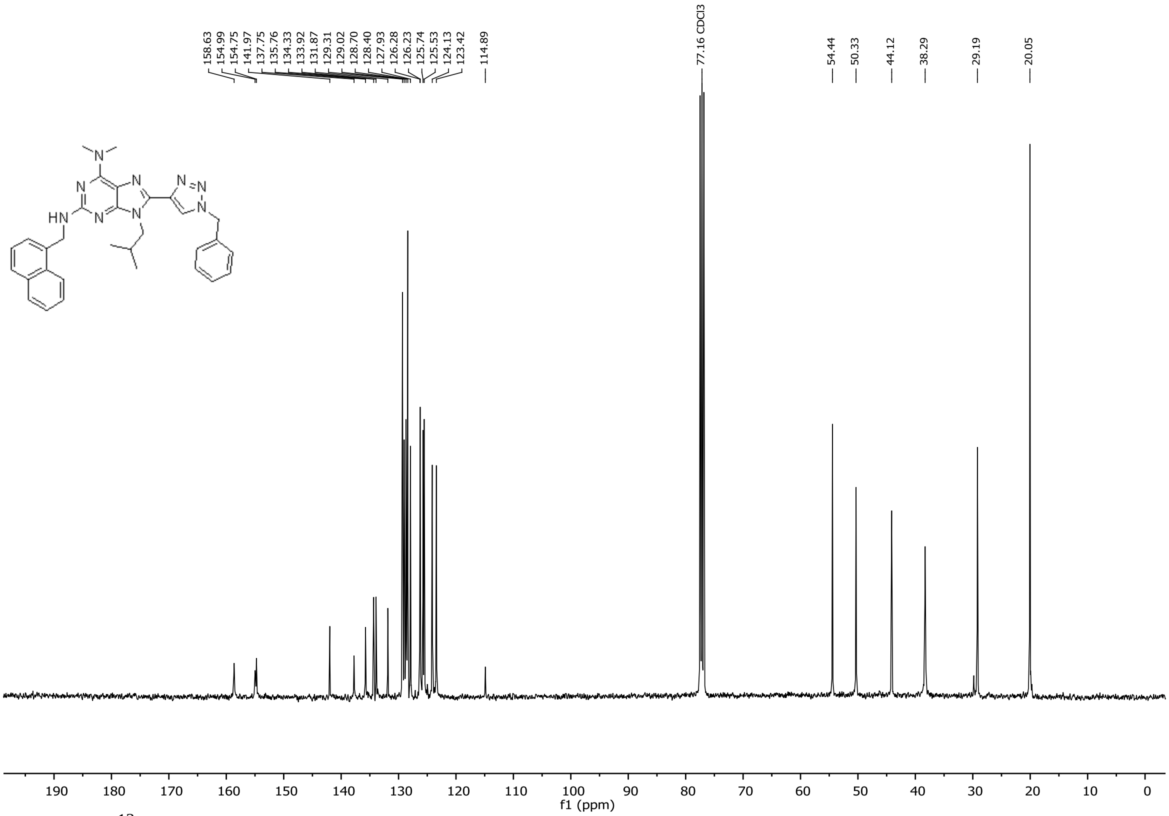

Fig. A25. ^{13}C NMR spectrum of 6-dimethylamino-9-isobutyl-2-naphtalenylamino-8-(1-benzyl-1H-1,2,3-triazol-4-yl)purine (**7a**).

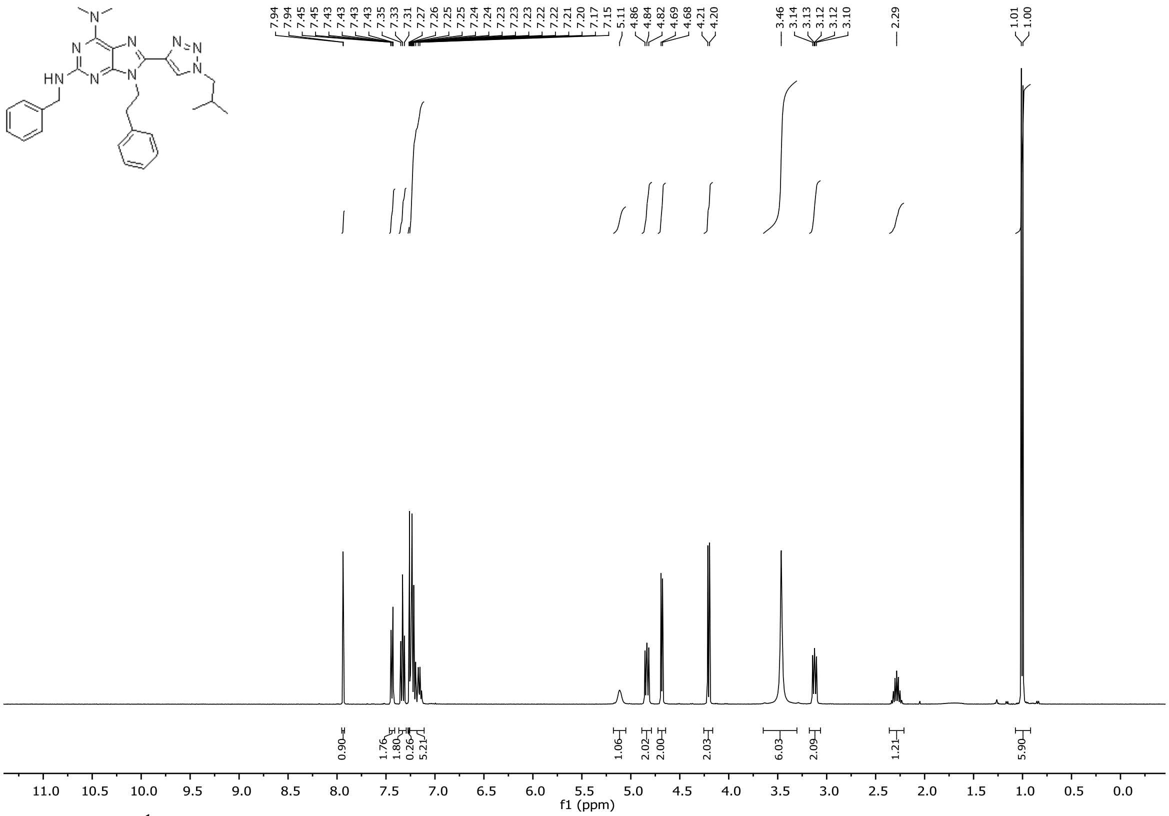

Fig. A26. ^{1}H NMR spectrum of 2-benzylamino-6-dimethylamino-8-(1-isobutyl-1H-1,2,3-triazol-4-yl)-9-phenethylpurine (**7b**).

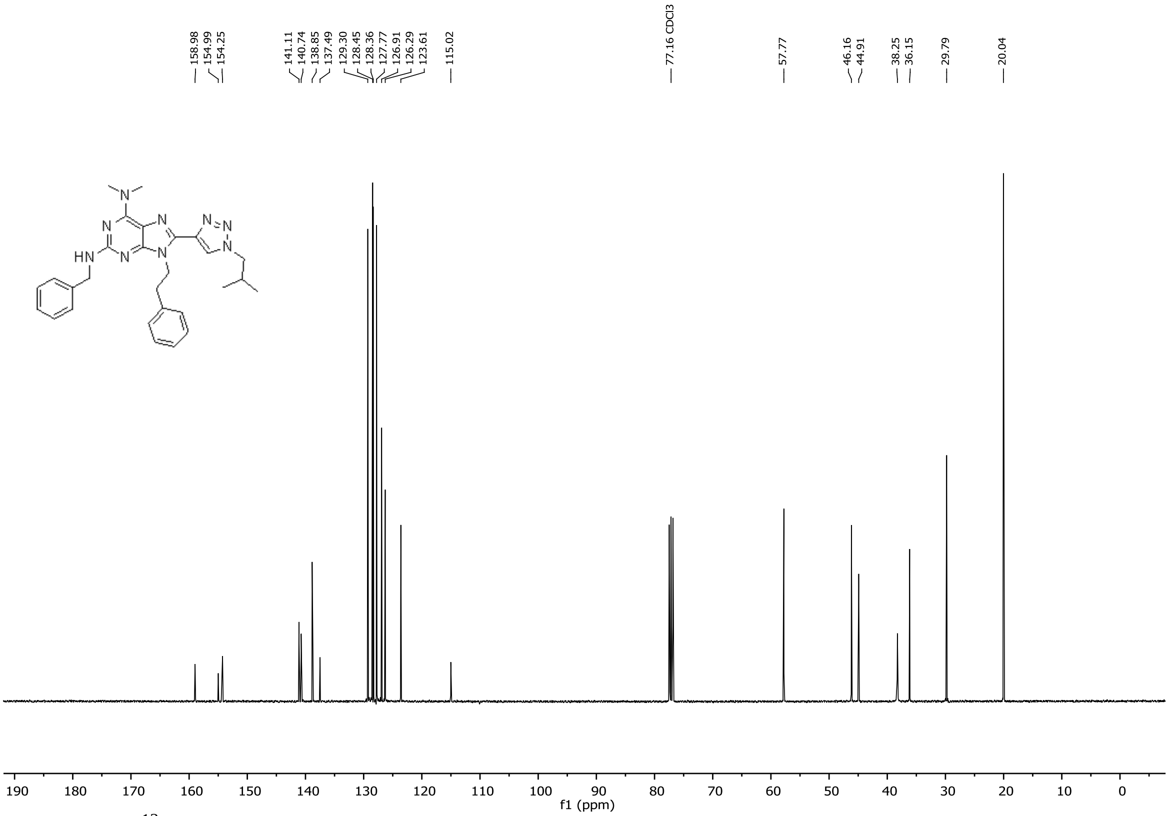

Fig. A27. ^{13}C NMR spectrum of 2-benzylamino-6-dimethylamino-8-(1-isobutyl-1H-1,2,3-triazol-4-yl)-9-phenethylpurine (**7b**).

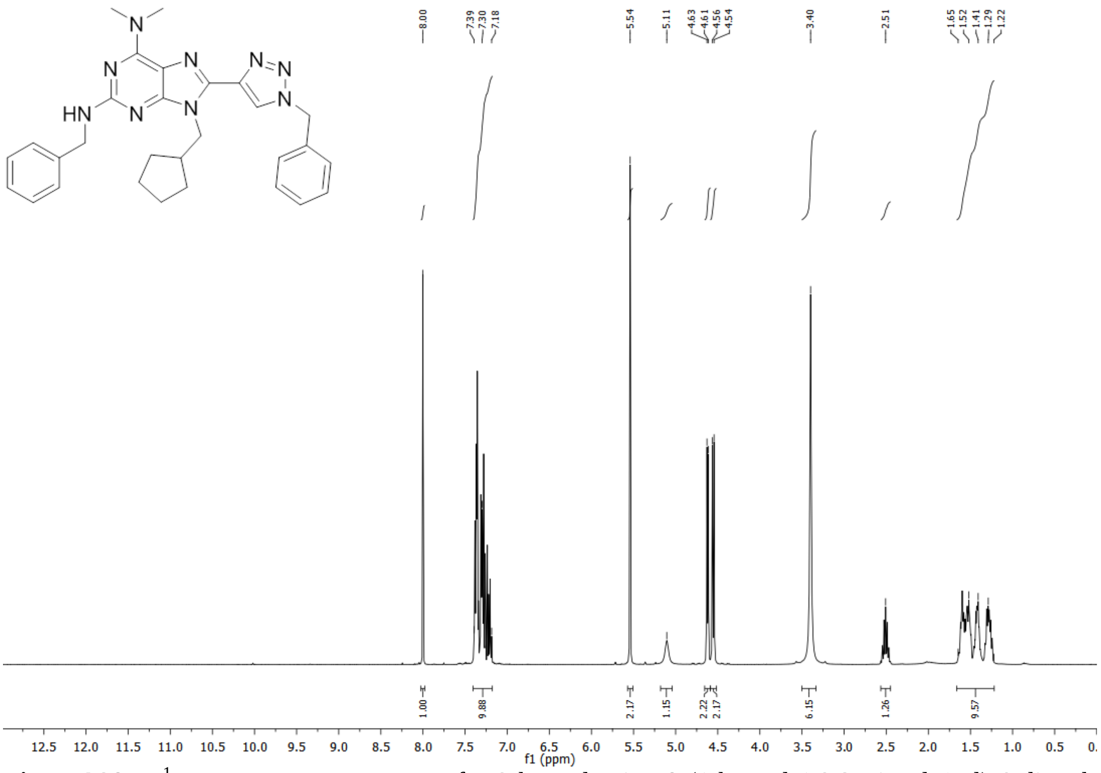

Fig. A28. ^{1}H NMR spectrum of 2-benzylamino-8-(1-benzyl-1,2,3-triazol-4-yl)-6-dimethylamino-9-cyclopentylmethylpurine (**7c**).

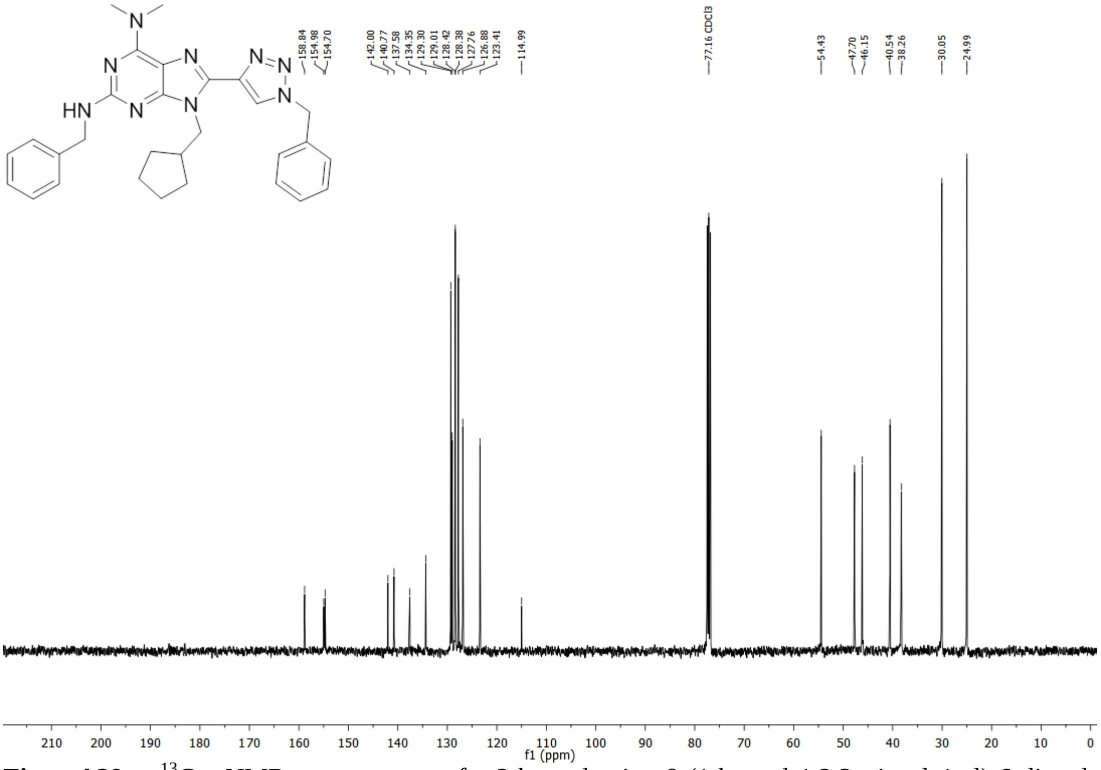

Fig. A29. ^{13}C NMR spectrum of 2-benzylamino-8-(1-benzyl-1,2,3-triazol-4-yl)-6-dimethylamino-9-cyclopentylmethylpurine (**7c**).

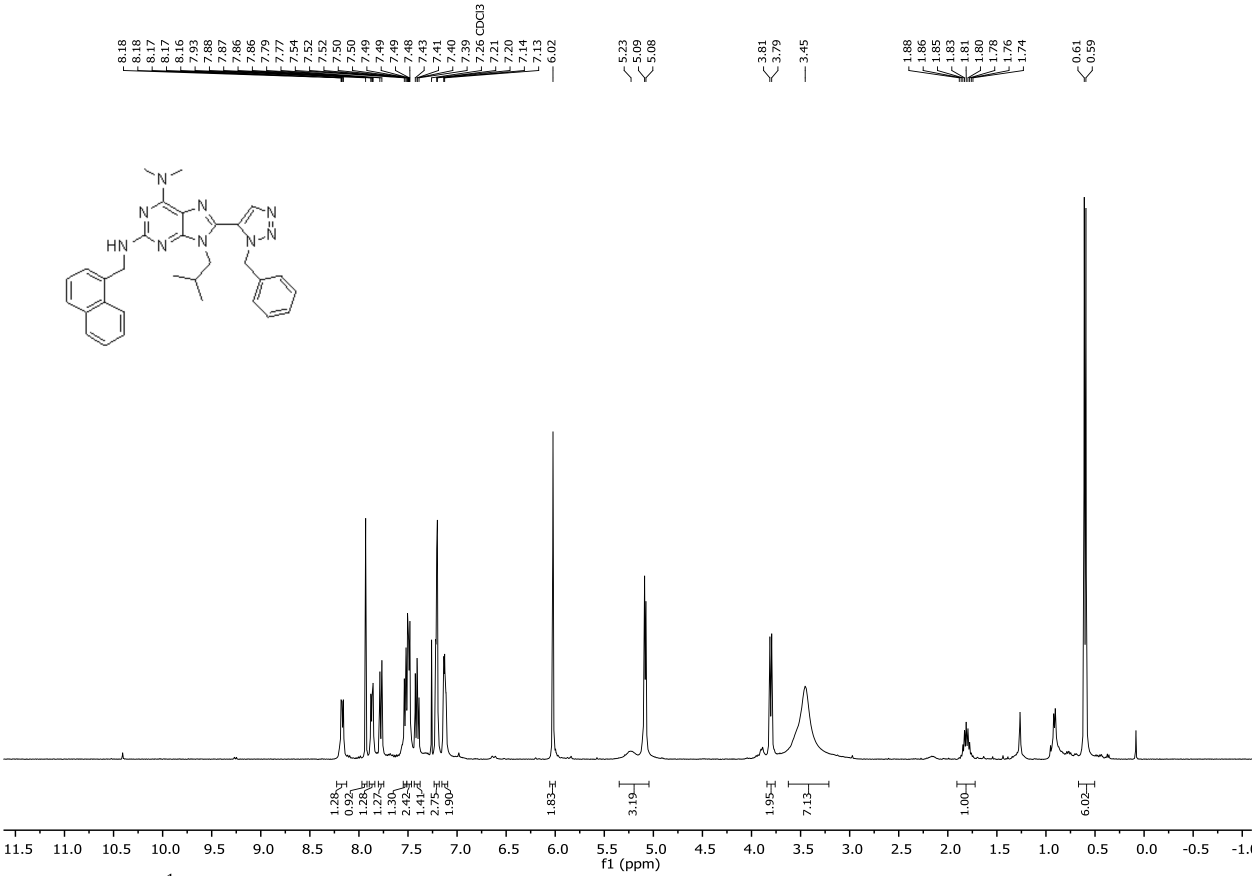

Fig. A30. ^{1}H NMR spectrum of 8-(1-benzyl-1H-1,2,3-triazol-5-yl)-6-dimethylamino-9-isobutyl-2-naphtalenylaminopurine (**8a**).

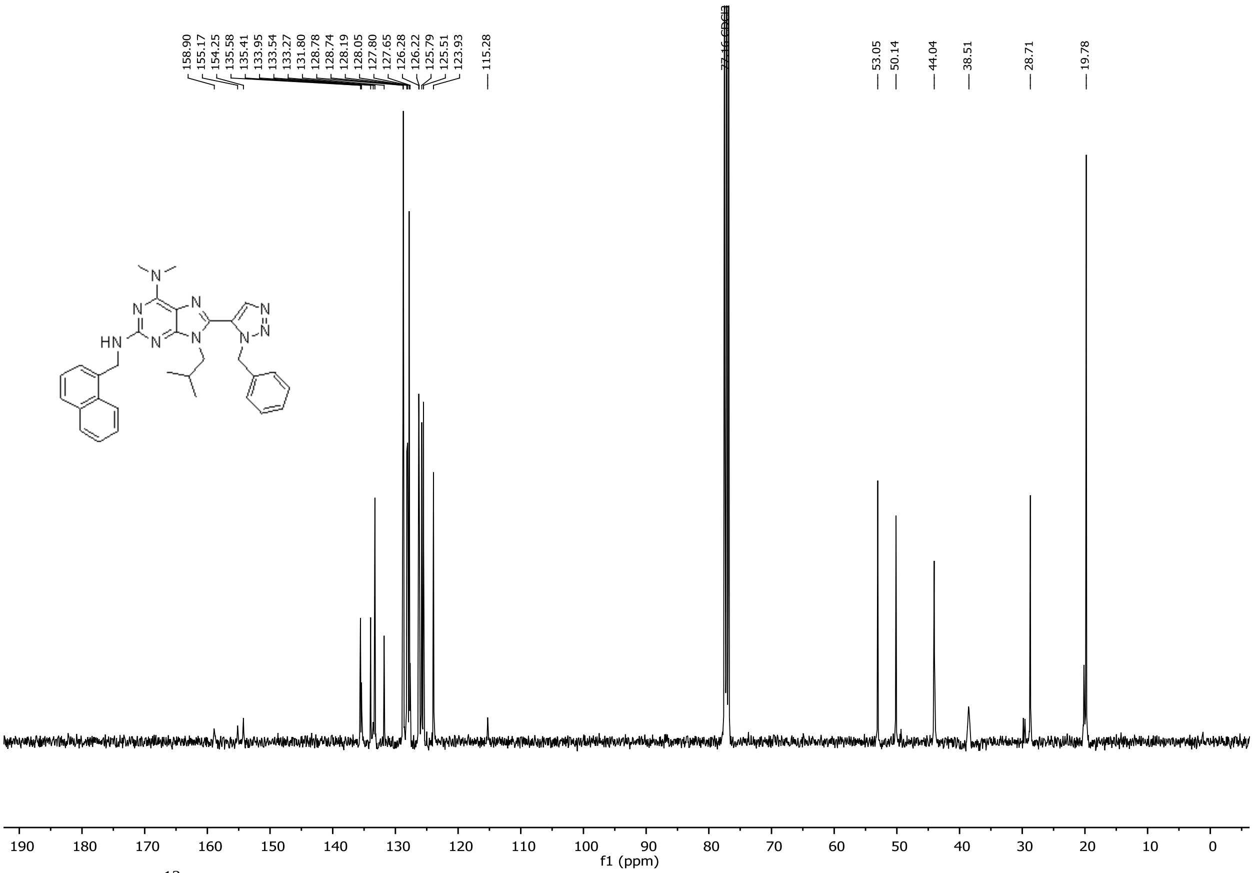

Fig. A31. ^{13}C NMR spectrum of 8-(1-benzyl-1H-1,2,3-triazol-5-yl)-6-dimethylamino-9-isobutyl-2-naphtalenylaminopurine (**8a**).

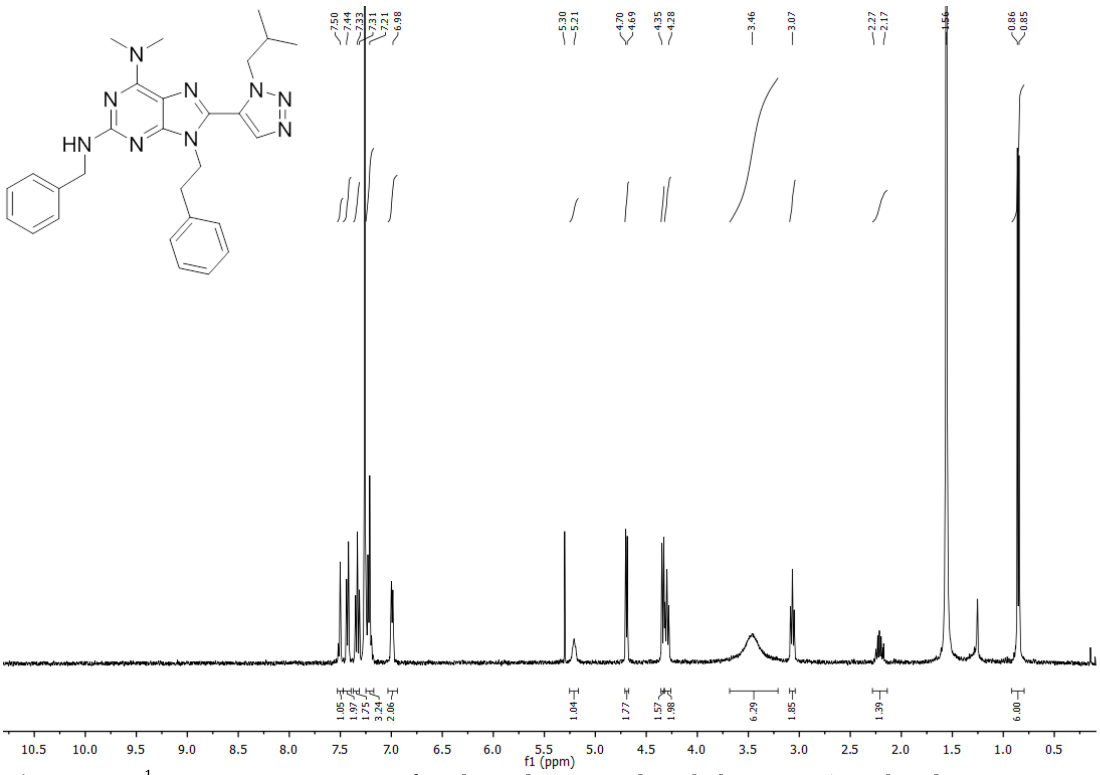

Fig. A32. ^{1}H NMR spectrum of 2-benzylamino-6-dimethylamino-8-(1-isobutyl-1H-1,2,3-triazol-5-yl)-9-phenethylpurine (**8b**).

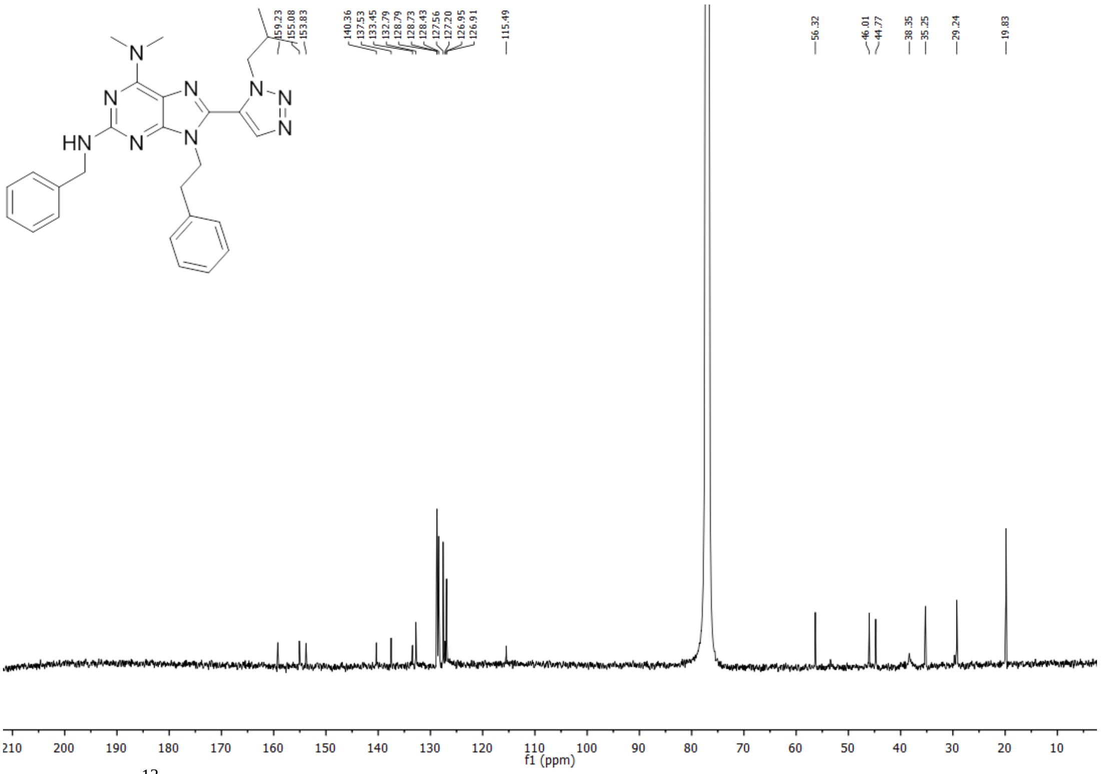

Fig. A33. ^{13}C NMR spectrum of 2-benzylamino-6-dimethylamino-8-(1-isobutyl-1H-1,2,3-triazol-5-yl)-9-phenethylpurine (**8b**).

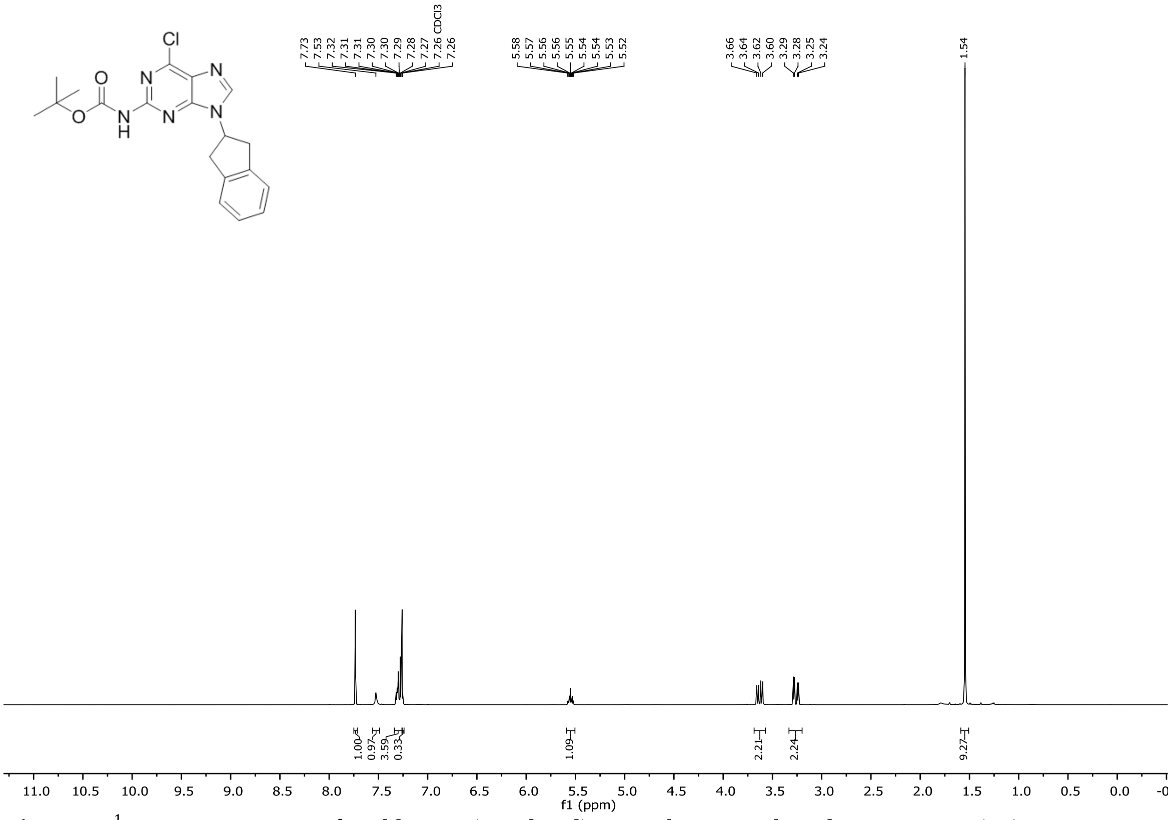

Fig. A34. ^{1}H NMR spectrum of 6-chloro-9-(2-indanyl)-2-*tert*-butoxycarbonylaminopurine (**9a**).

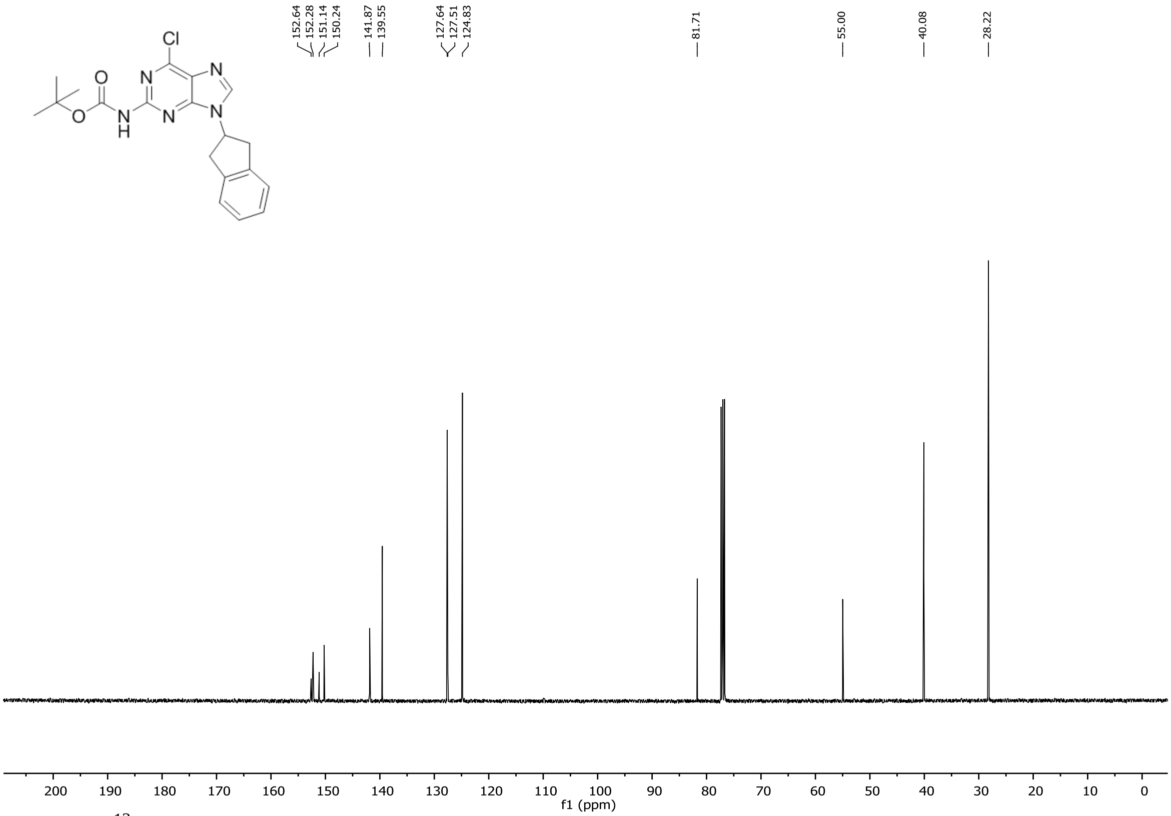

Fig. A35. ^{13}C NMR spectrum of 6-chloro-9-(2-indanyl)-2-*tert*-butoxycarbonylaminopurine (**9a**).

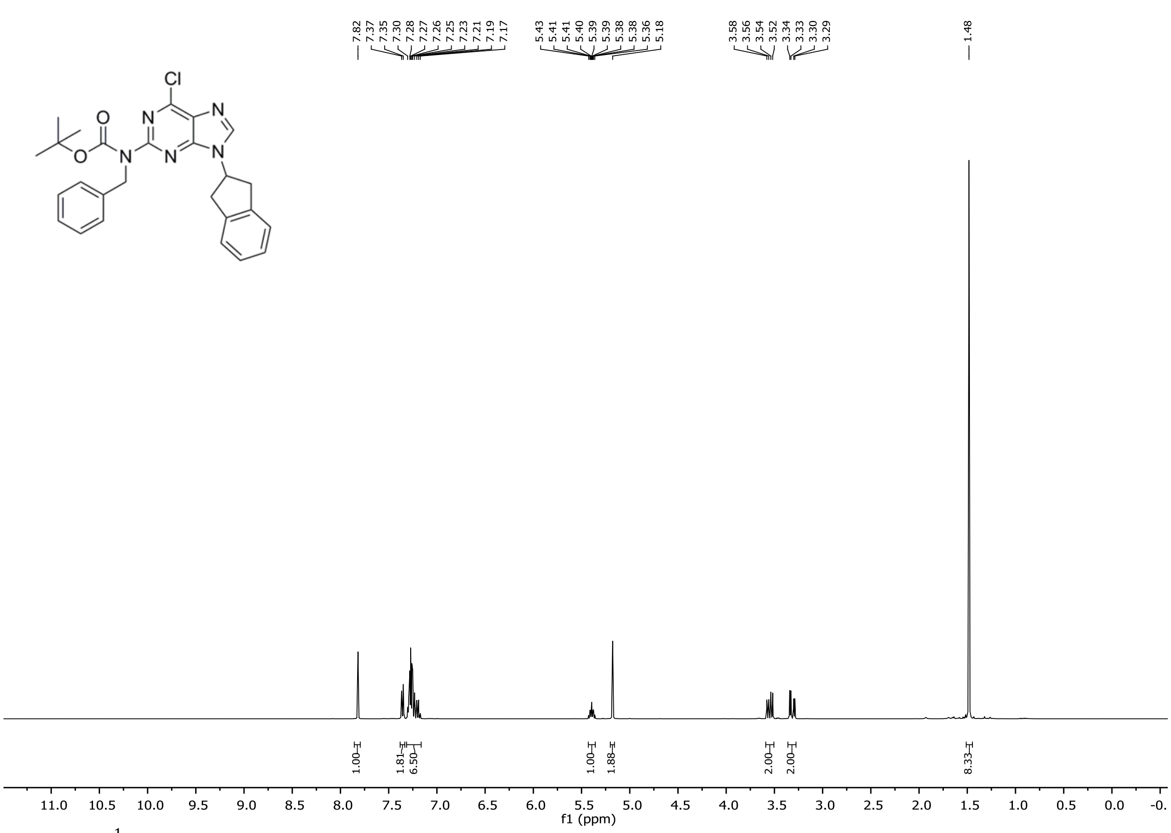

Fig. A36. ^{1}H NMR spectrum of 6-chloro-9-(2-indanyl)-2-(N-benzyl tert-butoxycarbonylamino)-purine (**10a**).

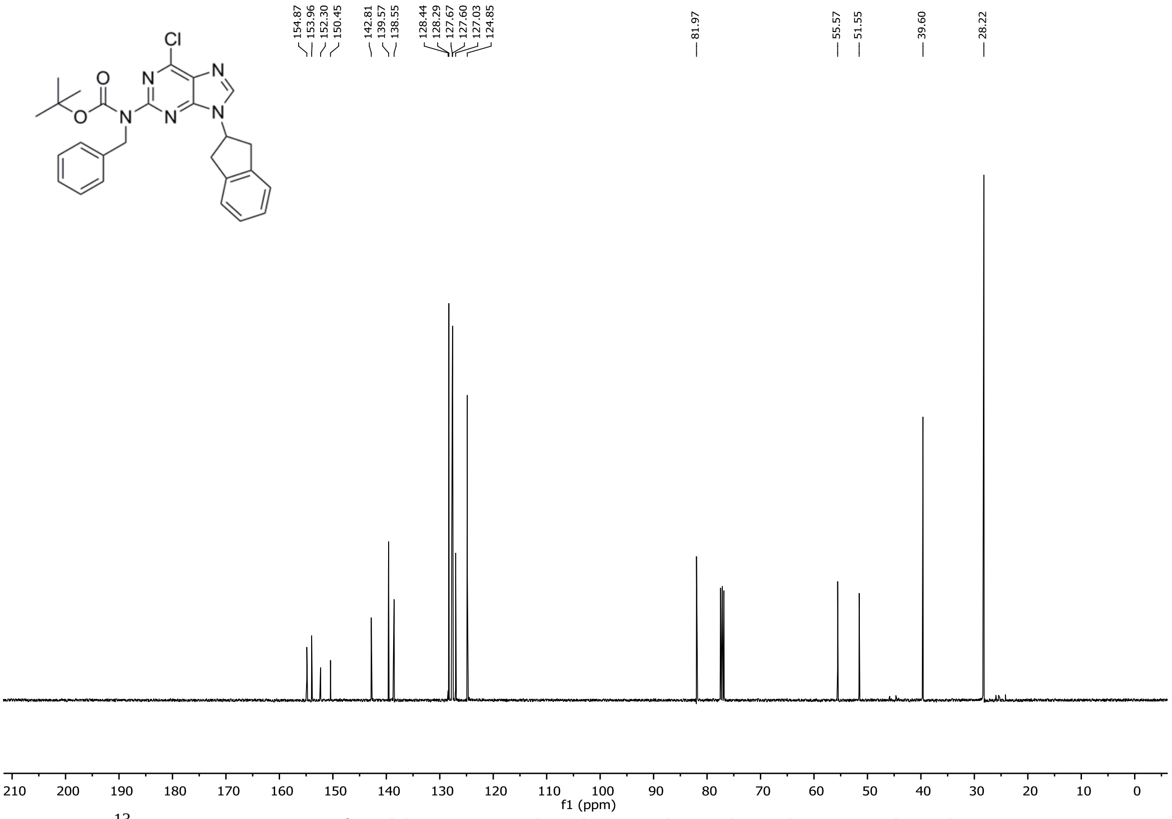

Fig. A37. ^{13}C NMR spectrum of 6-chloro-9-(2-indanyl)-2-(N-benzyl tert-butoxycarbonylamino)-purine (**10a**).

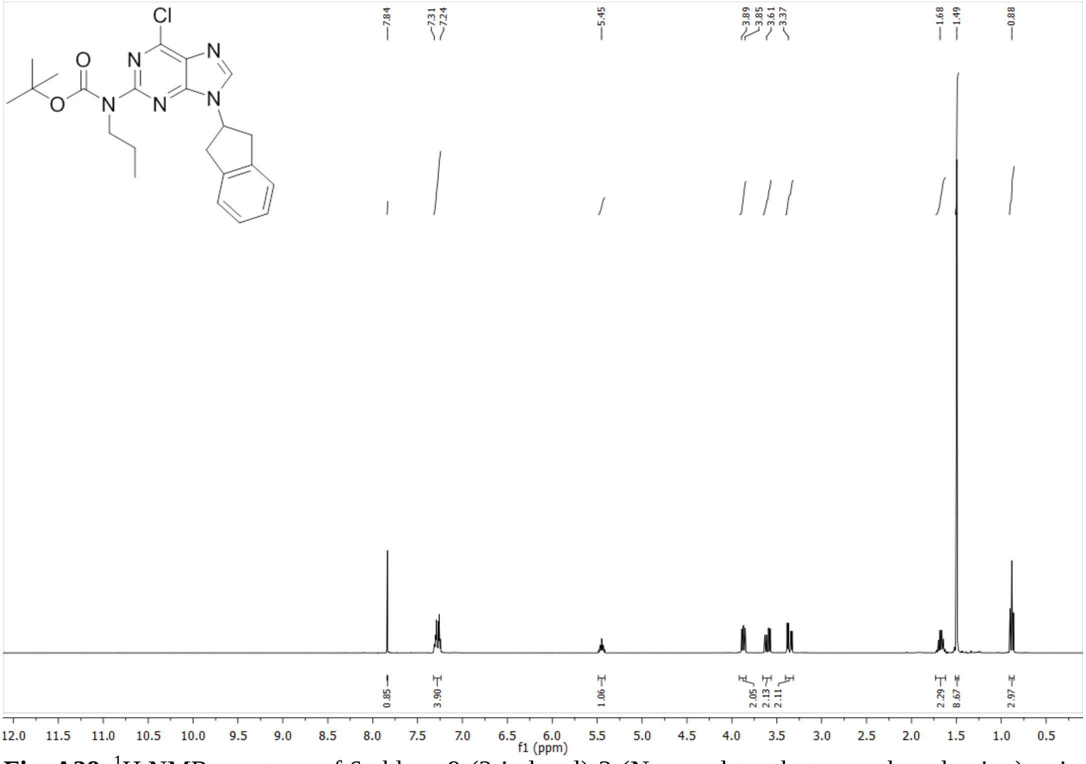

Fig. A38. ^{1}H NMR spectrum of 6-chloro-9-(2-indanyl)-2-(N-propyl-tert-butoxycarbonylamino)purine (**10b**).

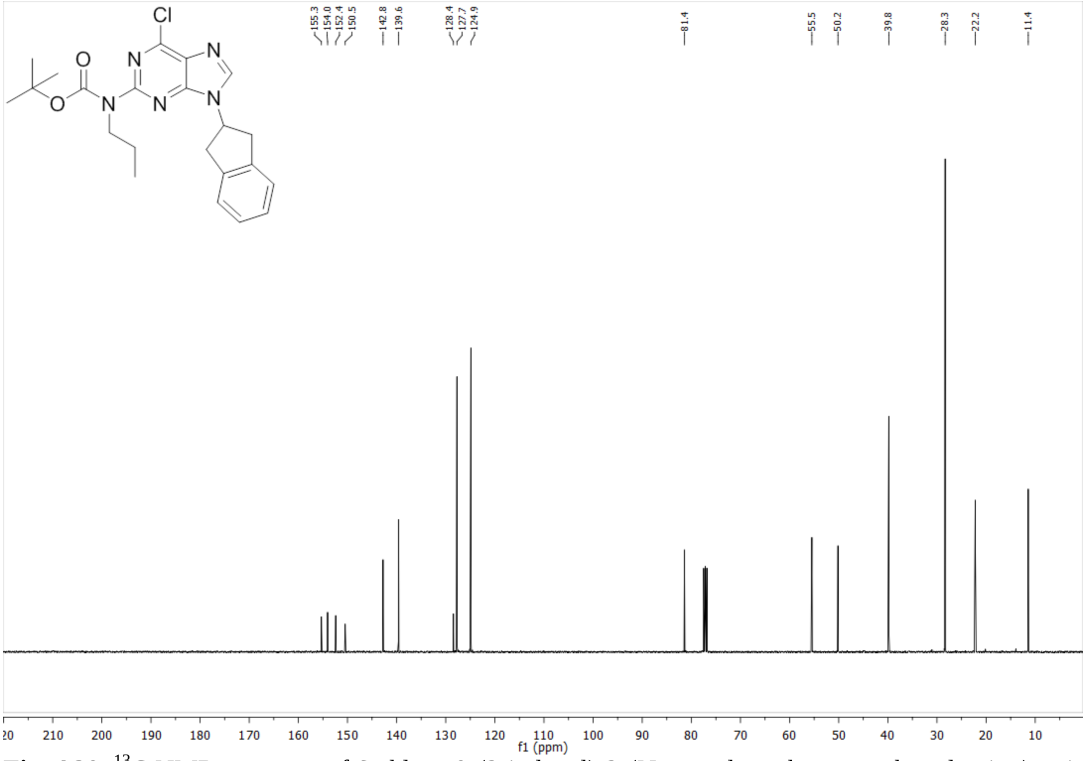

Fig. A39. ^{13}C NMR spectrum of 6-chloro-9-(2-indanyl)-2-(N-propyl-tert-butoxycarbonylamino)purine (**10b**).

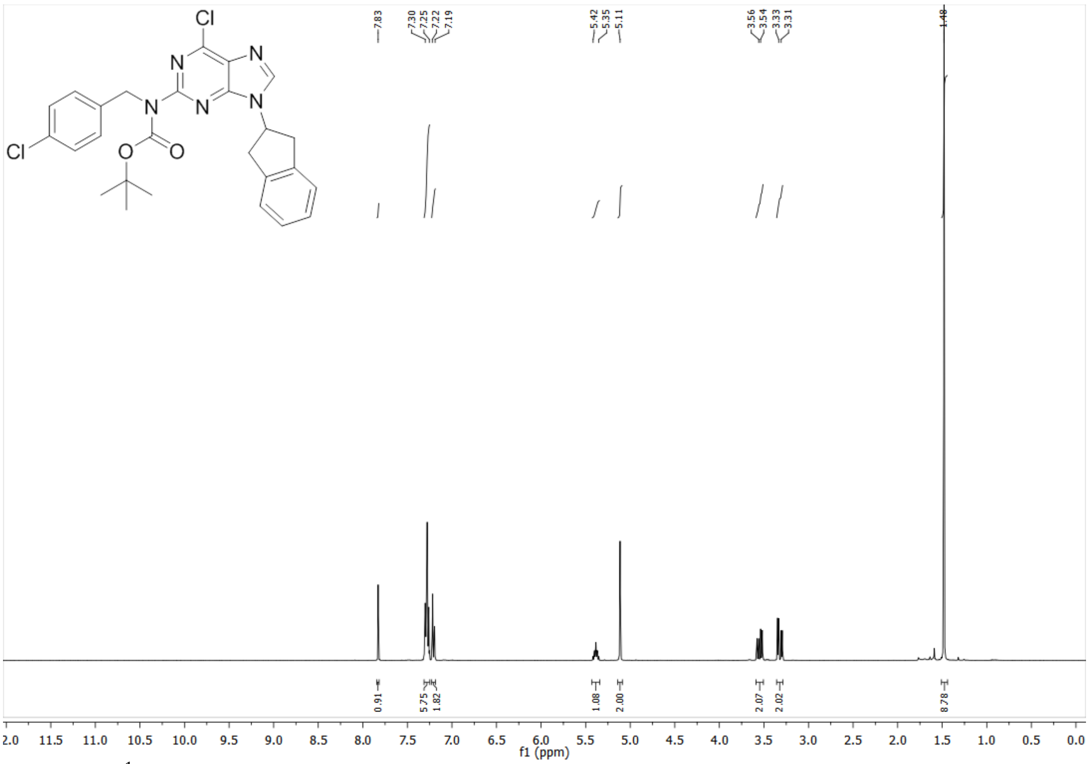

Fig. A40. ^{1}H NMR spectrum of 2-(N-*p*-Cl-benzyl-N-*tert*-butoxycarbonylamino)-6-chloro-9-(2-indanyl)purine (**10c**).

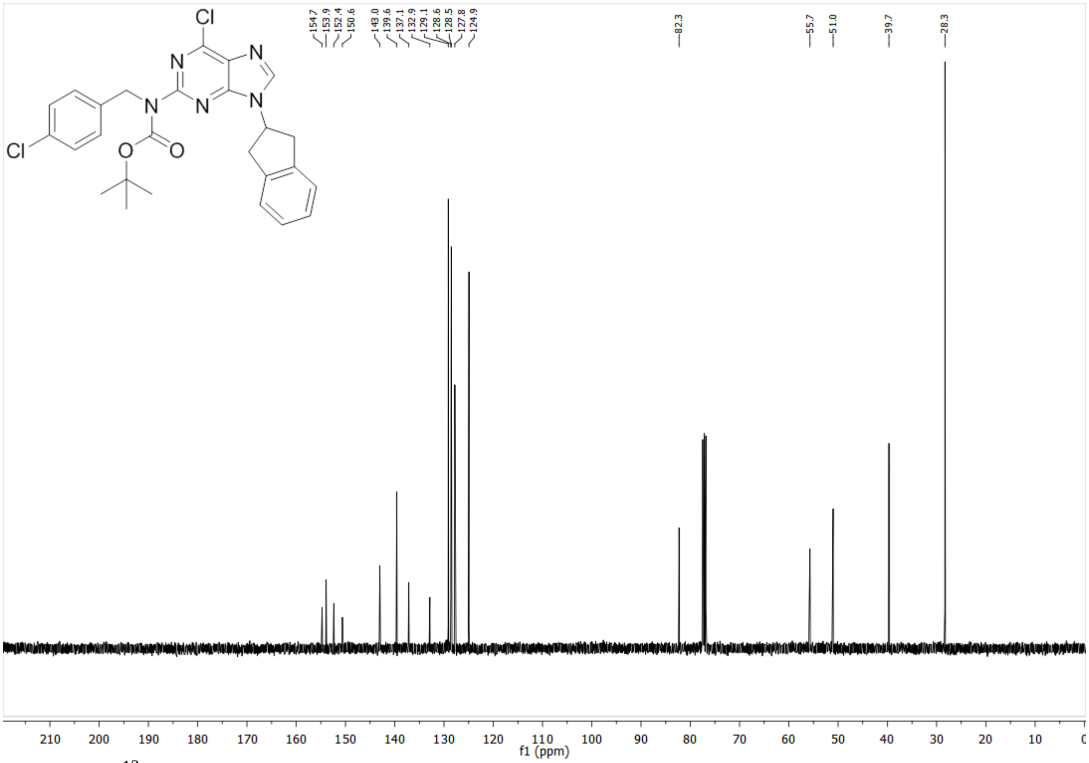

Fig. A41. ^{13}C NMR spectrum of 2-(N-*p*-Cl-benzyl-N-*tert*-butoxycarbonylamino)-6-chloro-9-(2-indanyl)purine (**10c**).

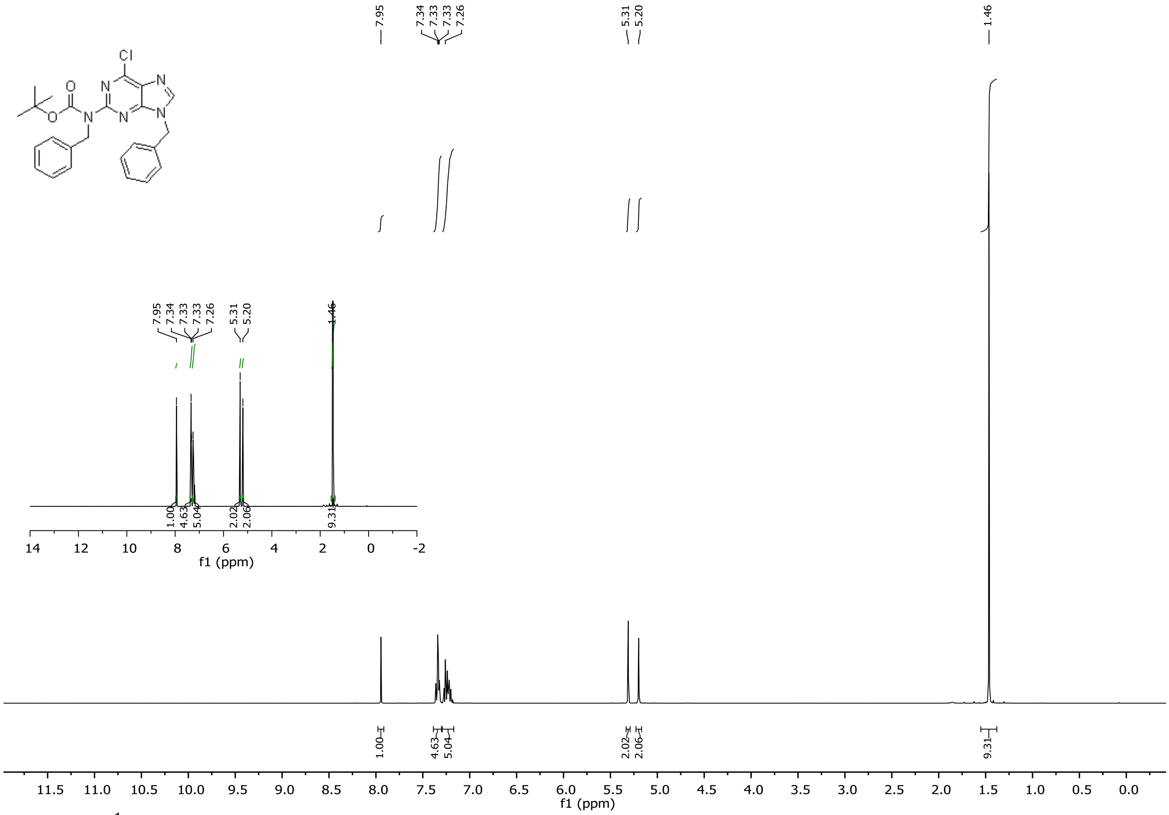

Fig. A42. ^{1}H NMR spectrum of 9-benzyl-2-(N-benzyl-*tert*-butoxycarbonylamino)-6-chloro-purine (**10d**).

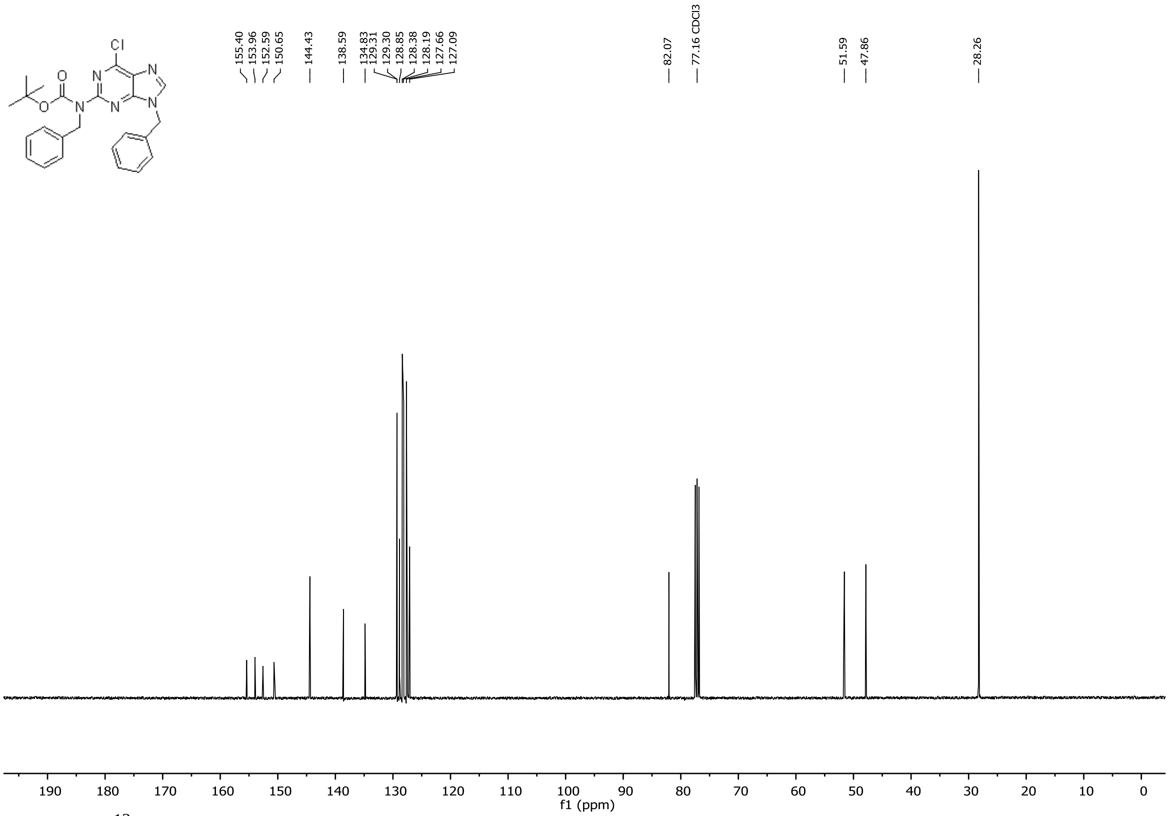

Fig. A43. ^{13}C NMR spectrum of 9-benzyl-2-(N-benzyl-*tert*-butoxycarbonylamino)-6-chloro-purine (**10d**).

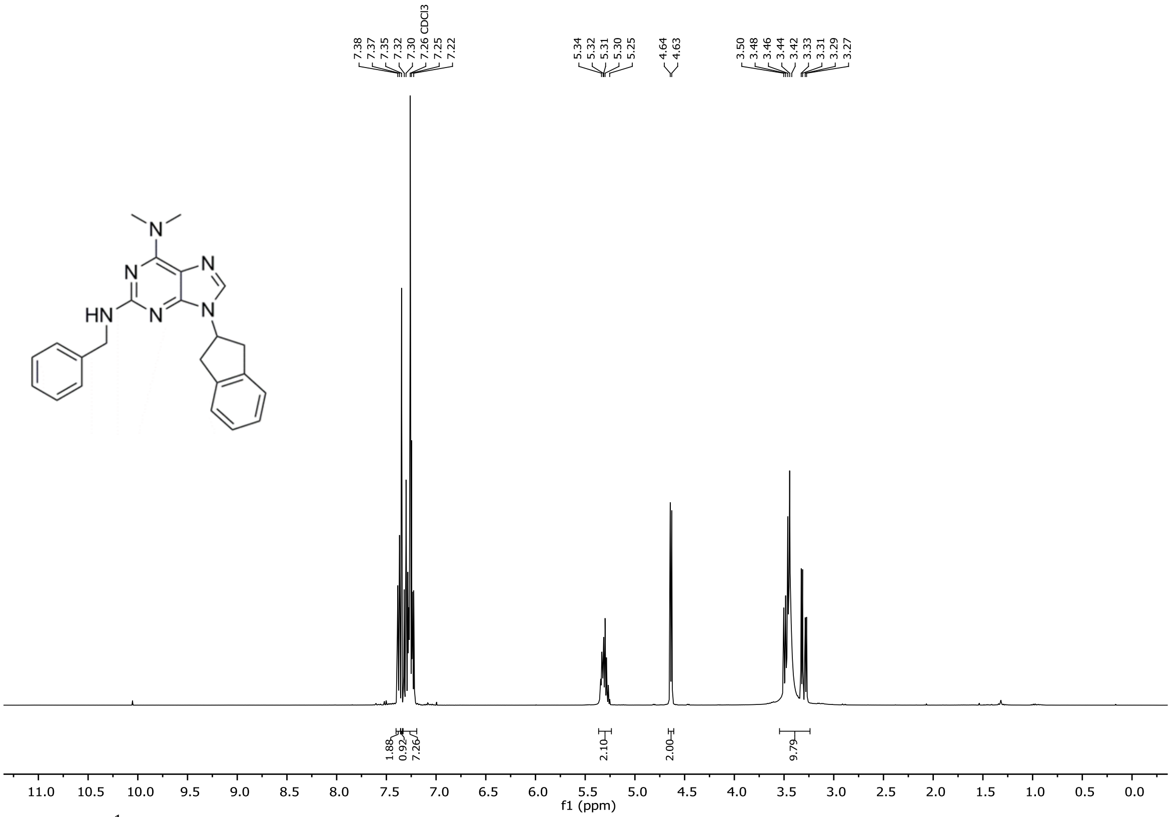

Fig. A44. ^{1}H NMR spectrum of 2-benzylamino-6-dimethylamino-9-(2-indanyl)-purine (**11a**).

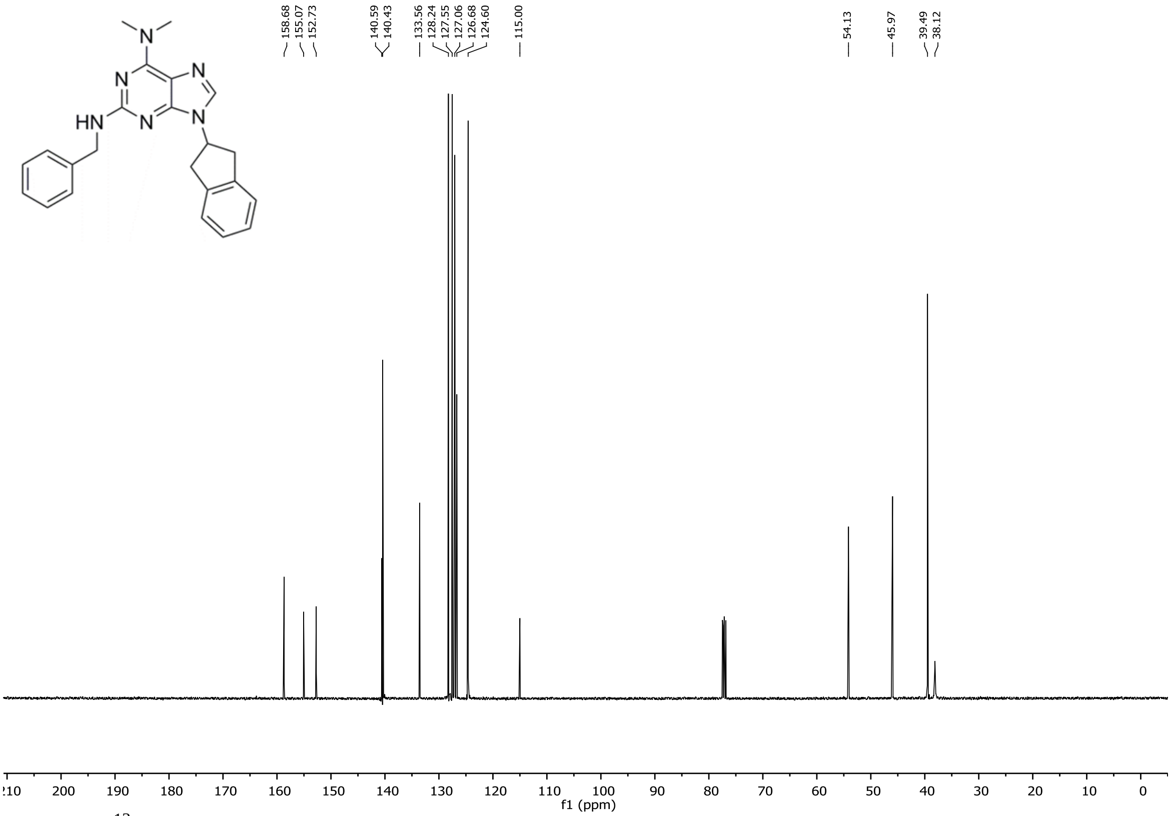

Fig. A45. ^{13}C NMR spectrum of 2-benzylamino-6-dimethylamino-9-(2-indanyl)-purine (**11a**).

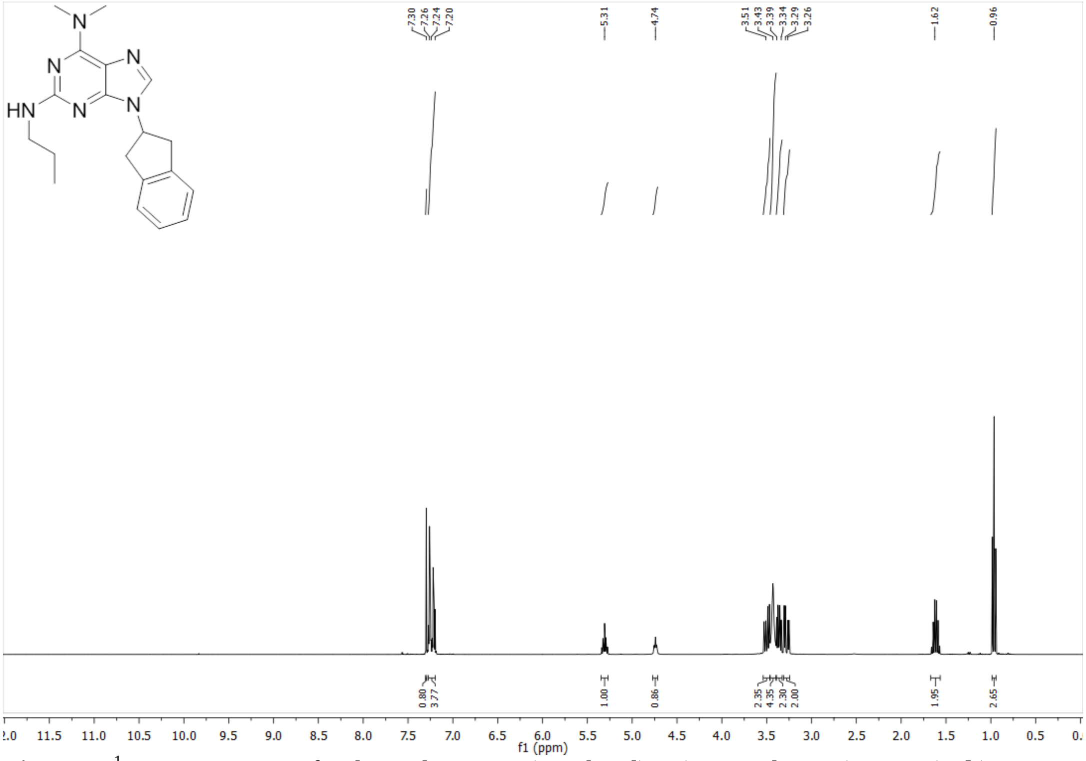

Fig. A46. ^{1}H NMR spectra of 6-dimetylamino-9-(2-indanyl)-2-(1-propylamino)purine (**11b**).

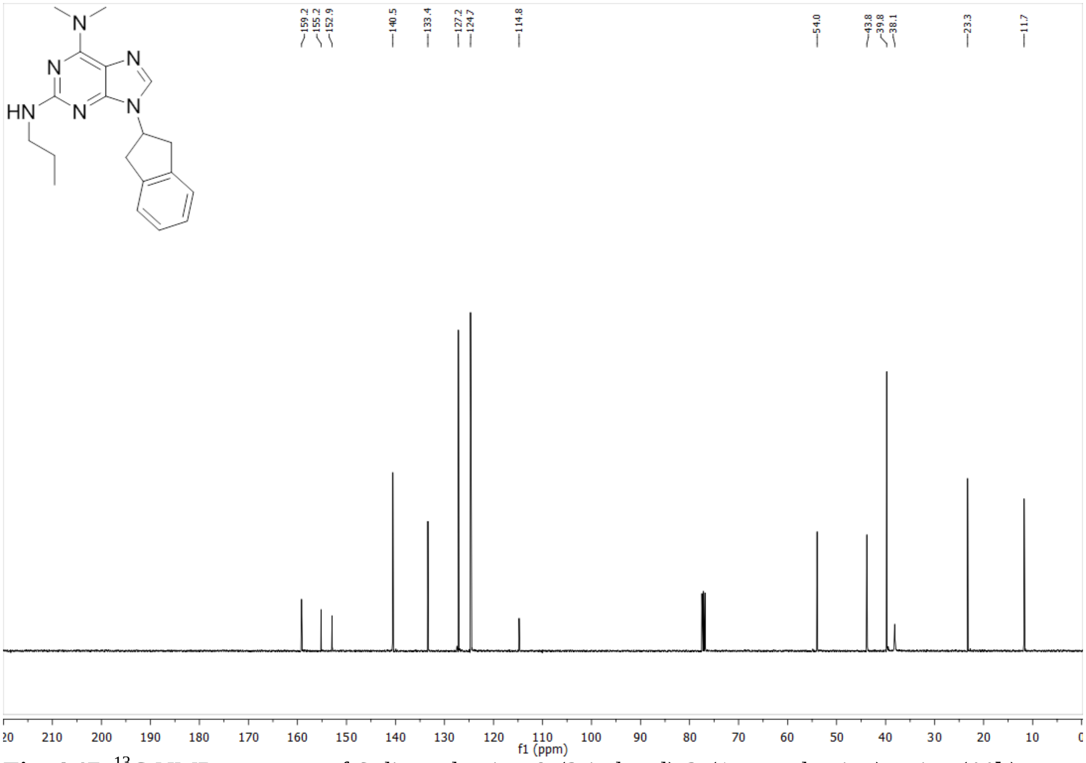

Fig. A47. ^{13}C NMR spectrum of 6-dimetylamino-9-(2-indanyl)-2-(1-propylamino)purine (**11b**).

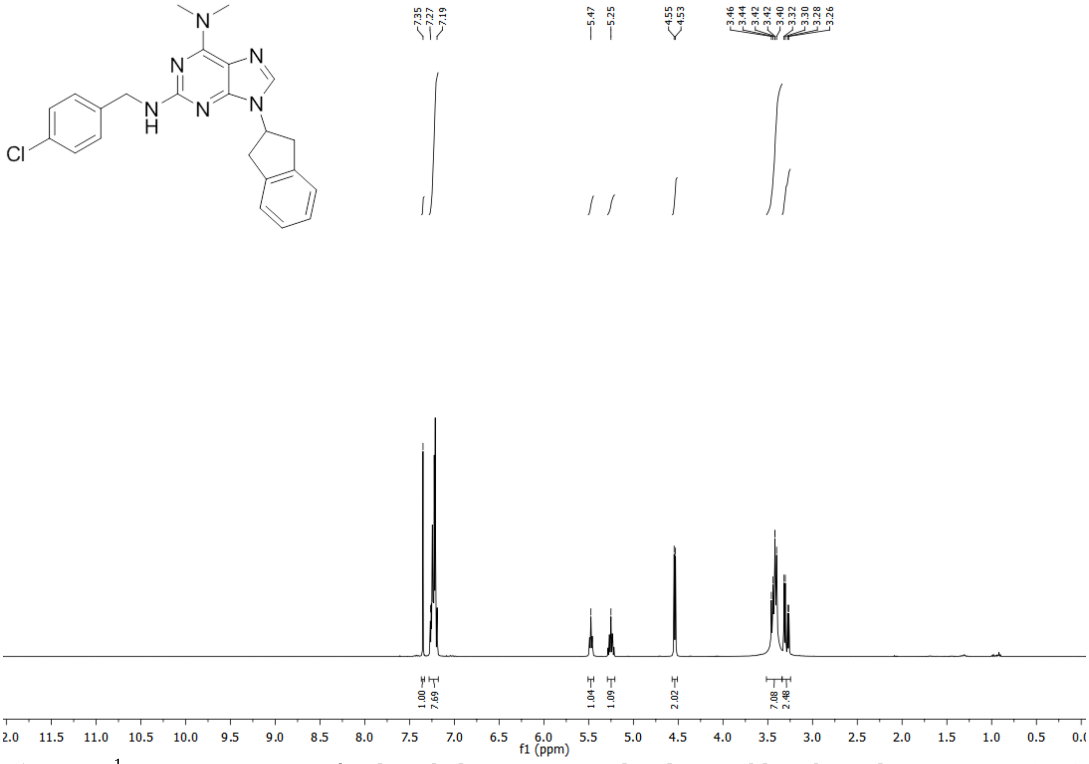

Fig. A48. ^{1}H NMR spectrum of 6-dimethylamino-9-(2-indanyl)-2-*p*-chloro-benzylaminopurine (**11c**).

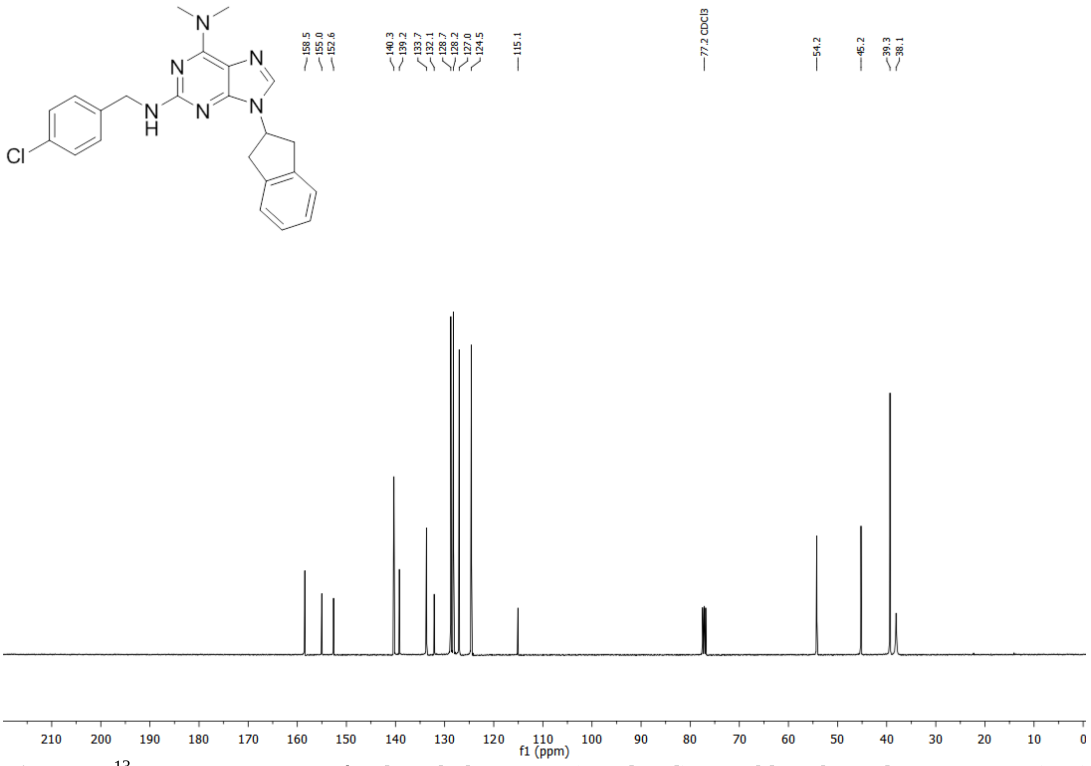

Fig. A49. ^{13}C NMR spectrum of 6-dimethylamino-9-(2-indanyl)-2-*p*-chloro-benzylaminopurine (**11c**).

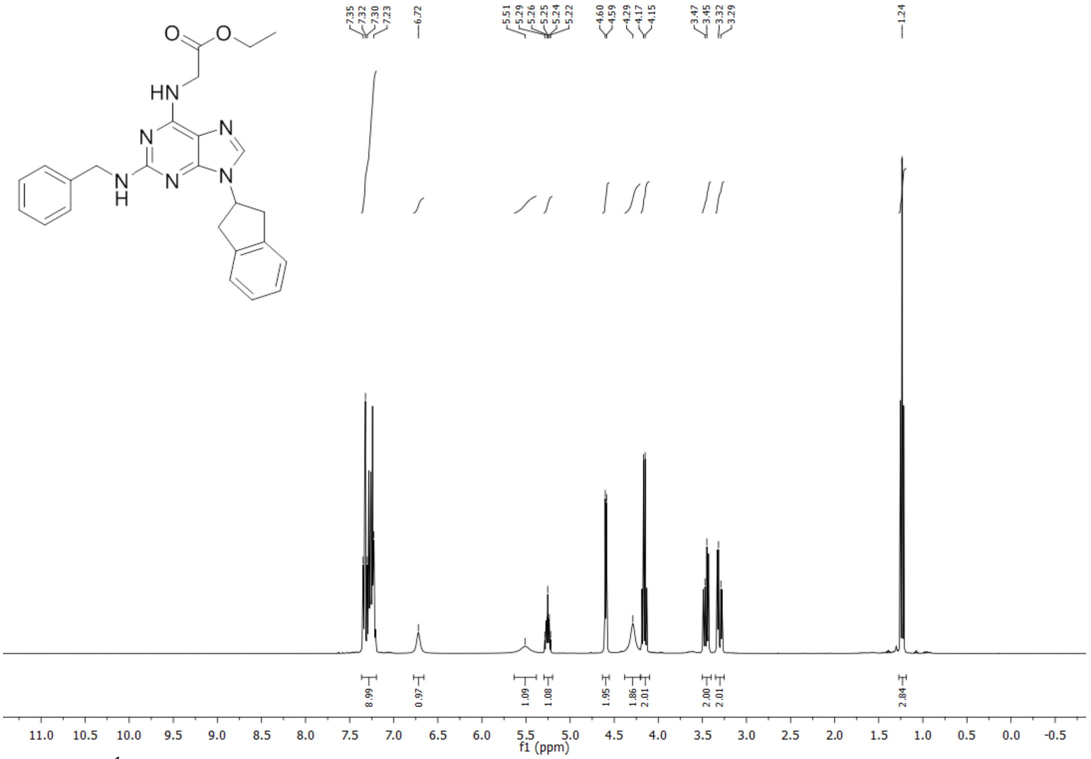

Fig. A50. ^{1}H NMR spectrum of 2-benzylamino-6-(ethoxy-carbonyl-methylamino)-9-(2-indanyl)-purine (**11d**).

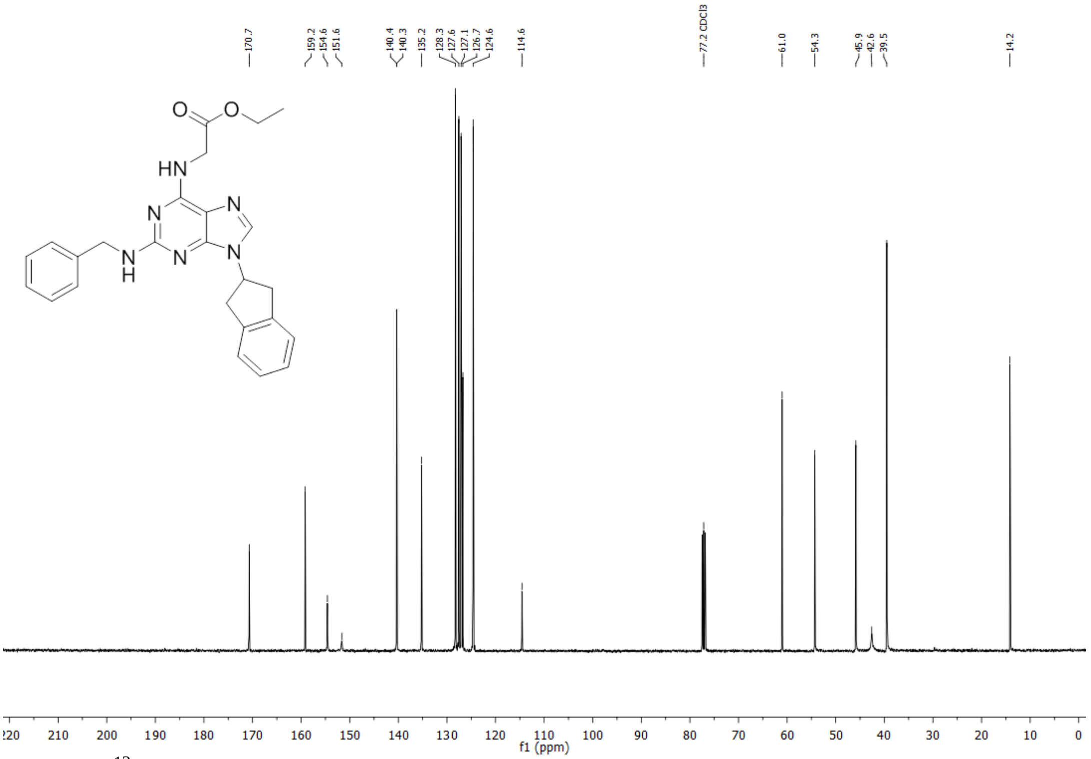

Fig. A51. ^{13}C NMR spectrum of 2-benzylamino-6-(ethoxy-carbonyl-methylamino)-9-(2-indanyl)-purine (**11d**).

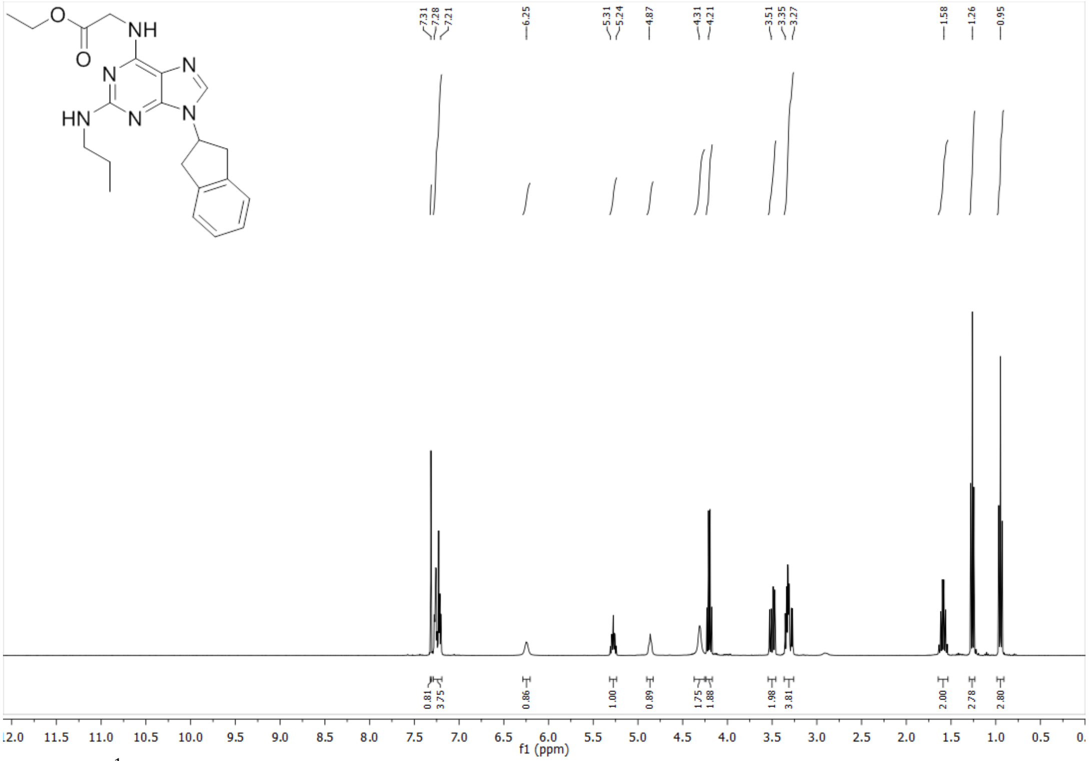

Fig. A52. ^{1}H NMR spectrum of 6-(ethoxy-carbonyl-methylamino)-9-(2-indanyl)-2-(1-propylamino)purine (**11e**).

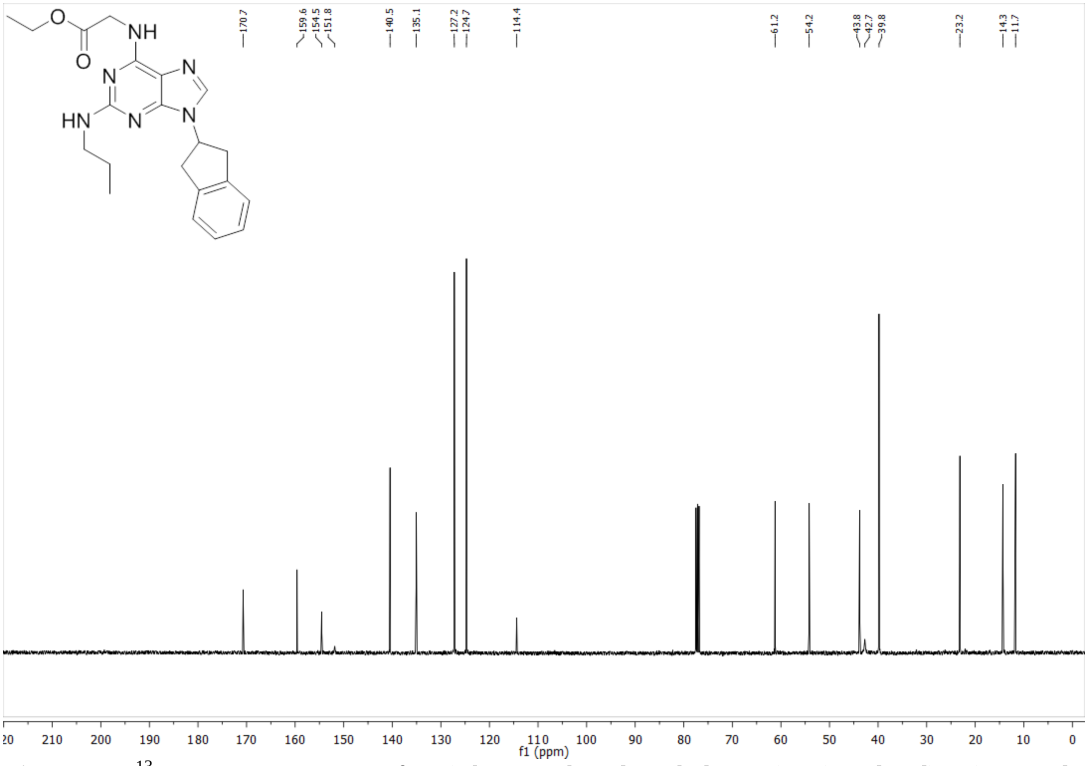

Fig. A53. ^{13}C NMR spectrum of 6-(ethoxy-carbonyl-methylamino)-9-(2-indanyl)-2-(1-propylamino)purine (**11e**).

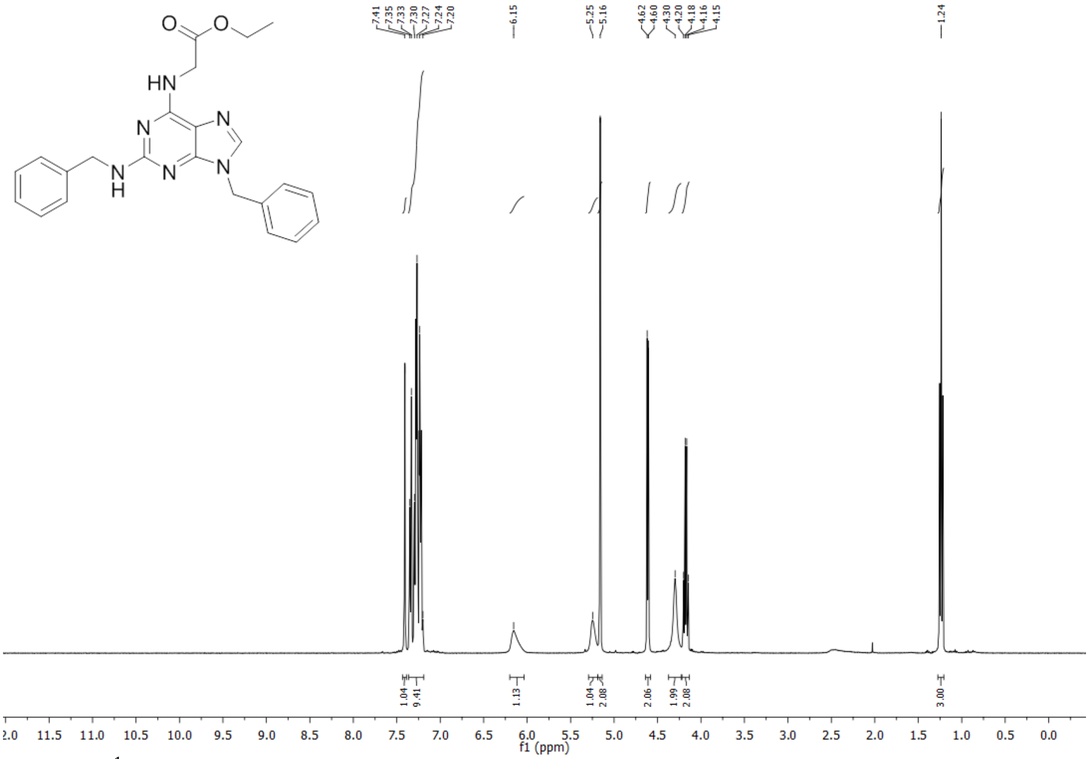

Fig. A54. ^{1}H NMR spectrum of 9-benzyl-2-benzylamino-6-(ethoxy-carbonyl-methylamino)-purine (**11f**).

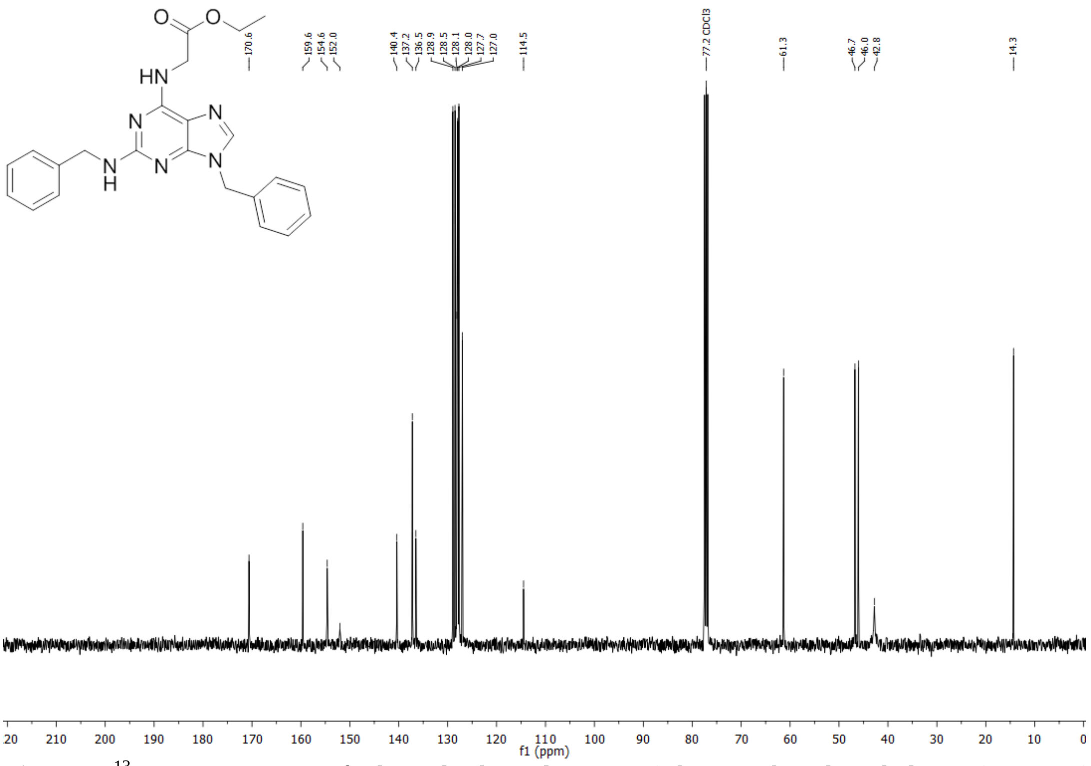

Fig. A55. ^{13}C NMR spectrum of 9-benzyl-2-benzylamino-6-(ethoxy-carbonyl-methylamino)-purine (**11f**).

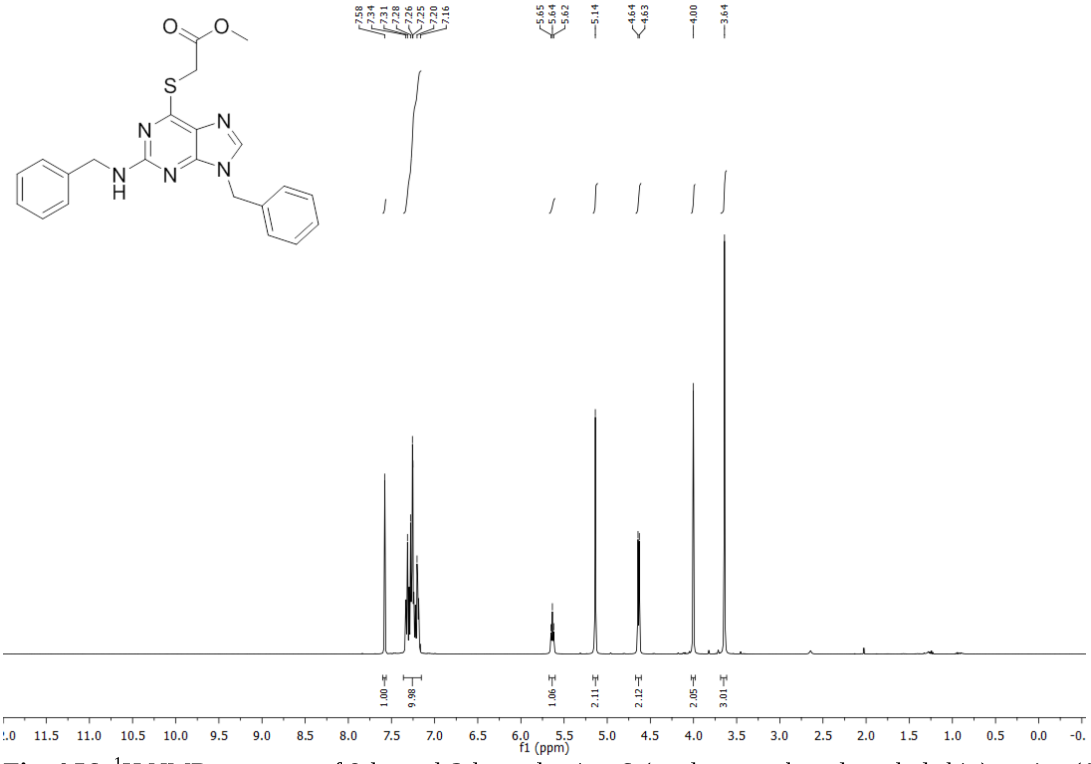

Fig. A56. ^{1}H NMR spectrum of 9-benzyl-2-benzylamino-6-(methoxy-carbonyl-methyl-thio)-purine (**11g**).

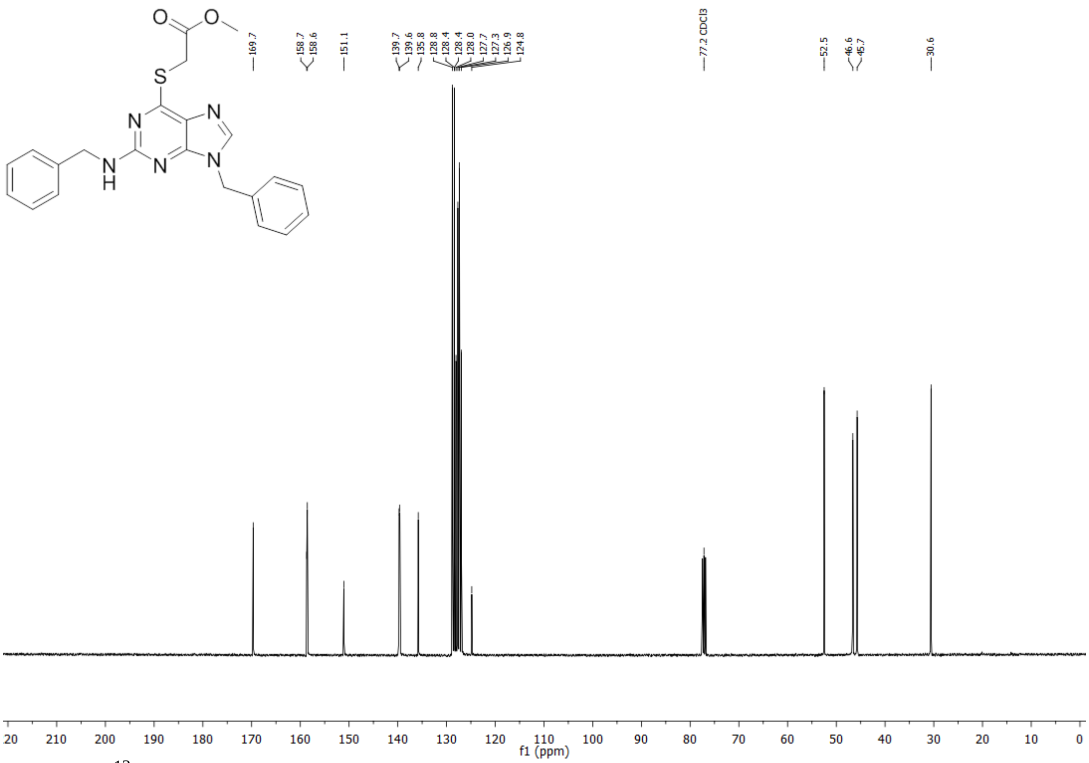

Fig. A57. ^{13}C NMR spectrum of 9-benzyl-2-benzylamino-6-(methoxy-carbonyl-methyl-thio)-purine (**11g**).

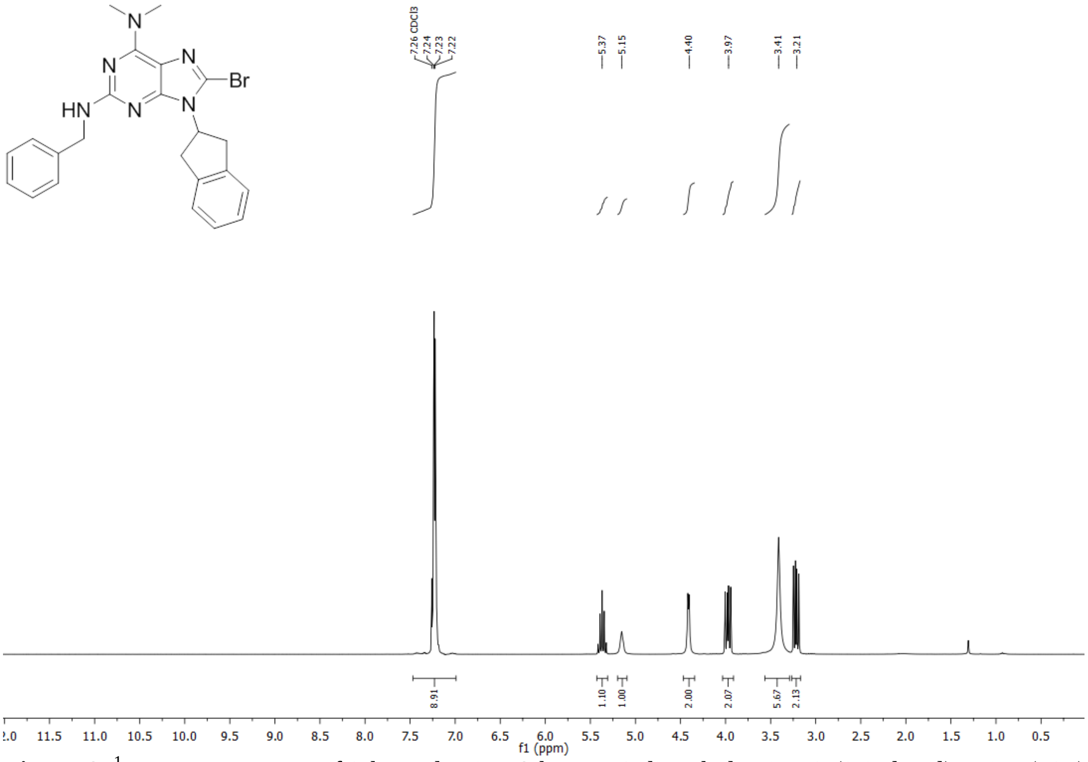

Fig. A58. ^{1}H NMR spectrum of 2-benzylamino-8-bromo-6-dimethylamino-9-(2-indanyl)purine (**12a**).

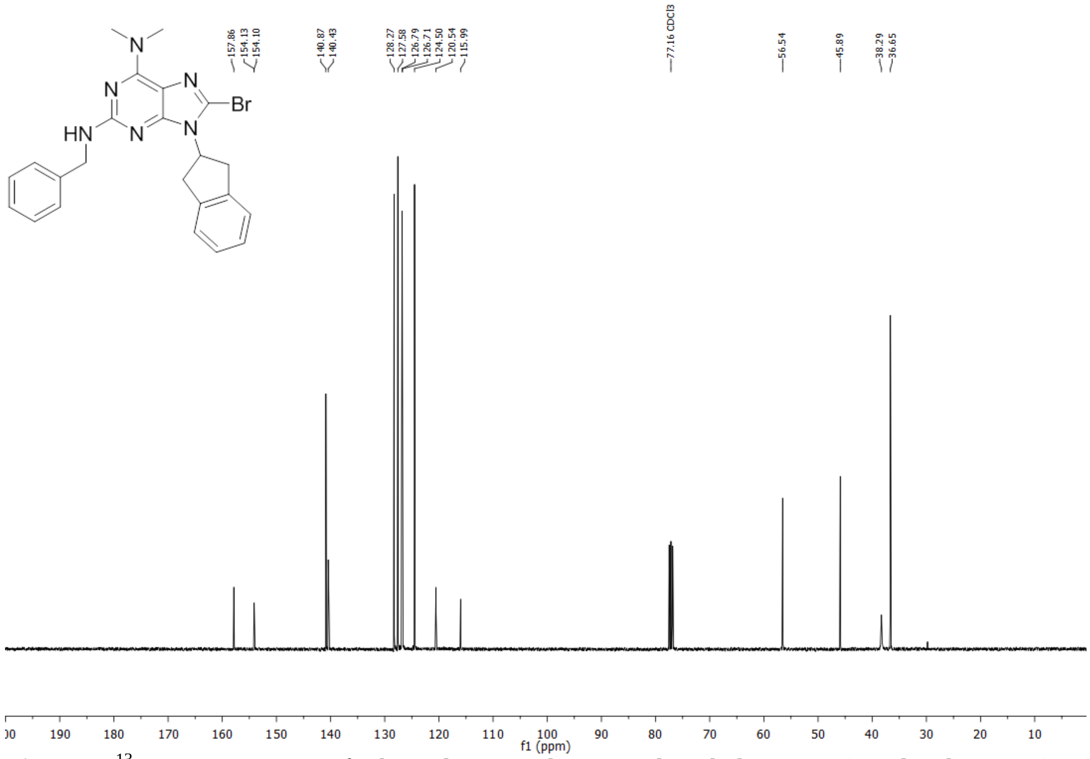

Fig. A59. ^{13}C NMR spectrum of 2-benzylamino-8-bromo-6-dimethylamino-9-(2-indanyl)purine (**12a**).

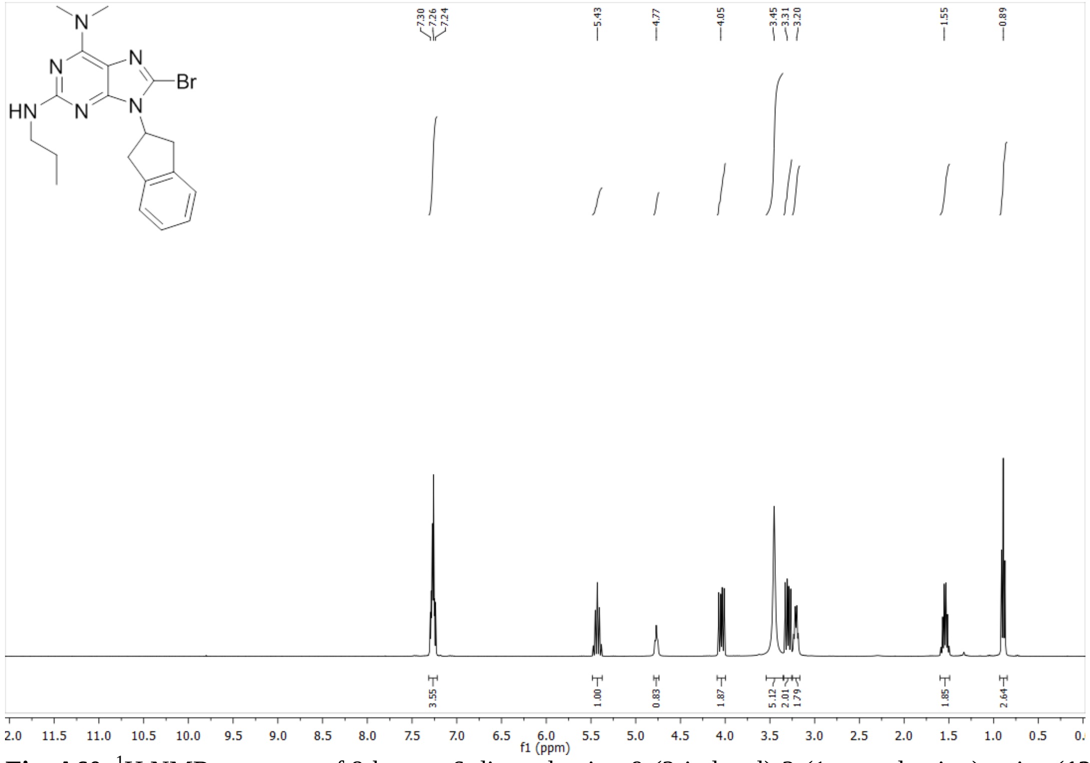

Fig. A60. ^{1}H NMR spectrum of 8-bromo-6-dimetylamino-9-(2-indanyl)-2-(1-propylamino)purine (**12b**).

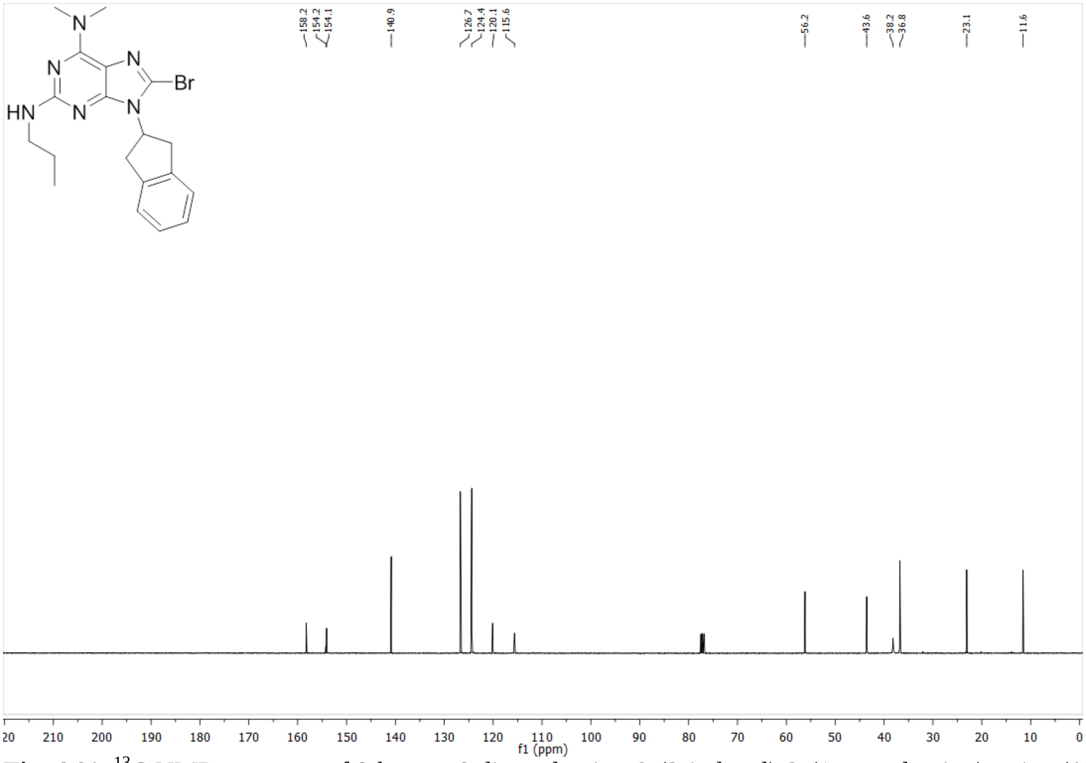

Fig. A61. ^{13}C NMR spectrum of 8-bromo-6-dimetylamino-9-(2-indanyl)-2-(1-propylamino)purine (**12b**).

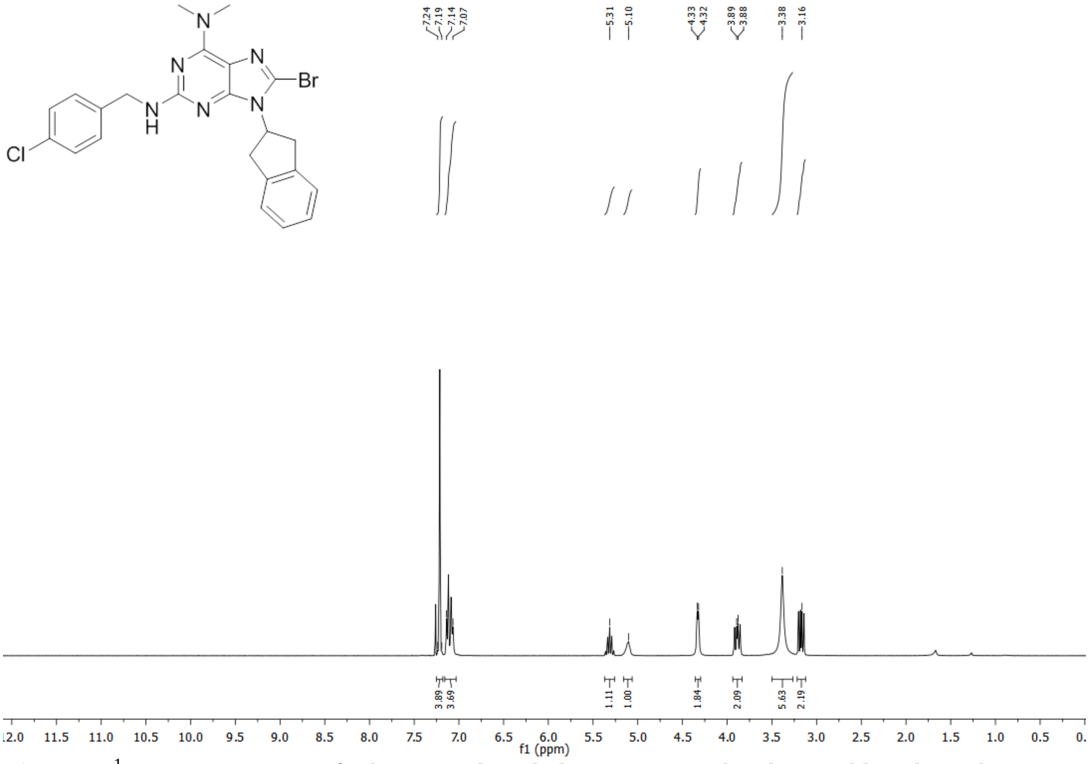

Fig. A62. ^{1}H NMR spectrum of 8-bromo-6-dimethylamino-9-(2-indanyl)-2-*p*-chloro-benzylaminopurine (**12c**).

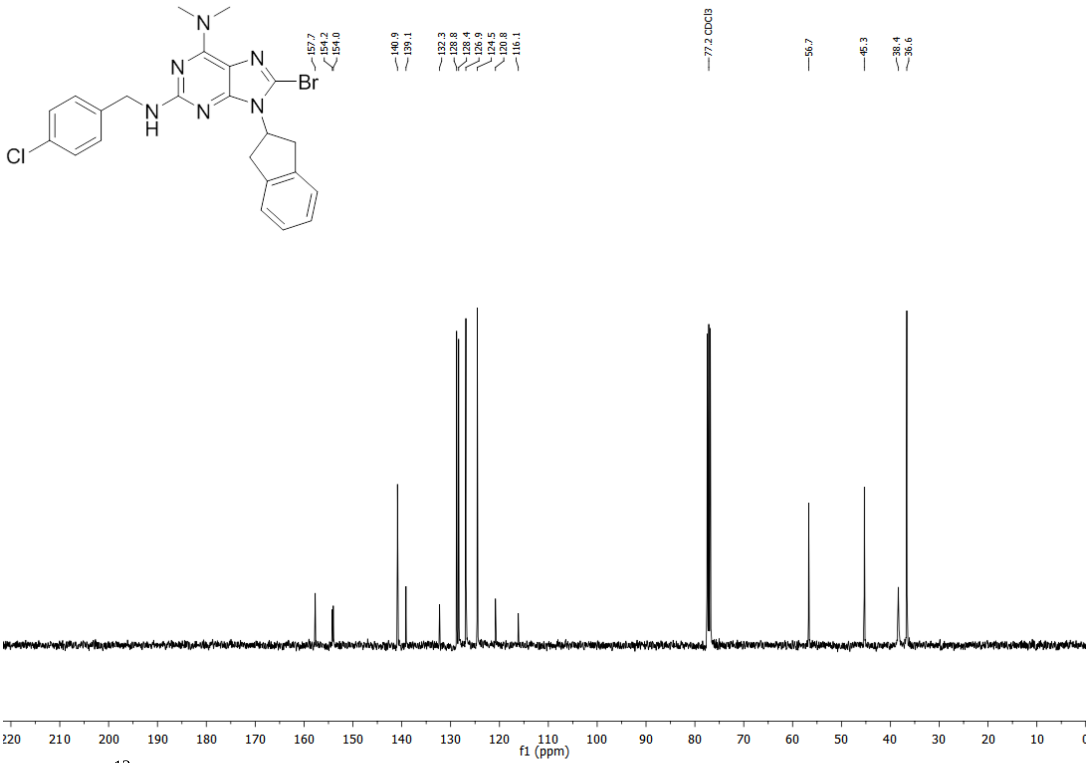

Fig. A63. ^{13}C NMR spectrum of 8-bromo-6-dimethylamino-9-(2-indanyl)-2-*p*-chloro-benzylaminopurine (**12c**).

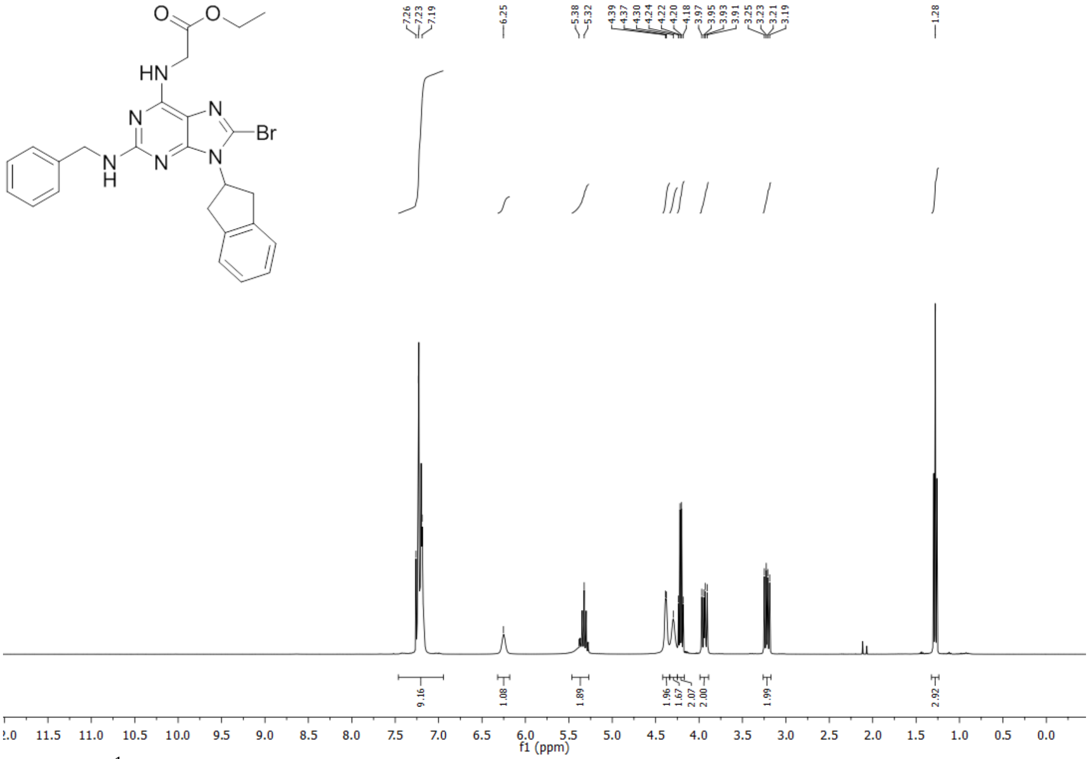

Fig. A64. ^{1}H NMR spectrum of 2-benzylamino-8-bromo-6-(ethoxy-carbonyl-methylamino)-9-(2-indanyl)-purine (**12d**).

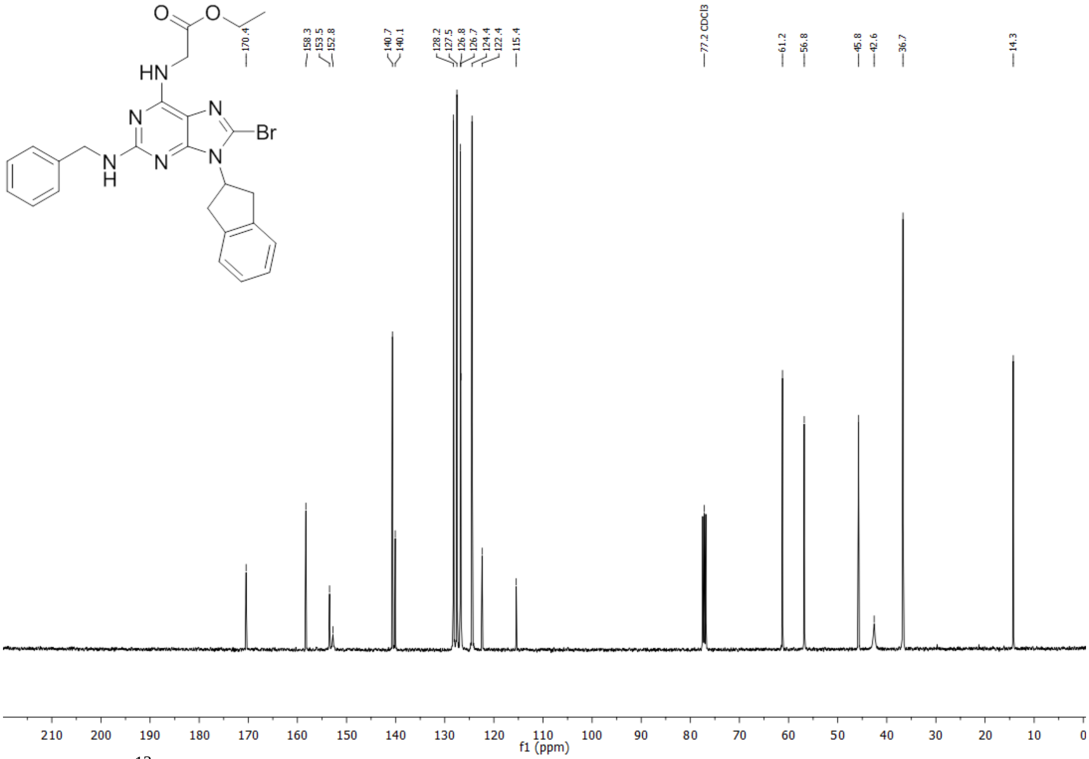

Fig. A65. ^{13}C NMR spectrum of 2-benzylamino-8-bromo-6-(ethoxy-carbonyl-methylamino)-9-(2-indanyl)-purine (**12d**).

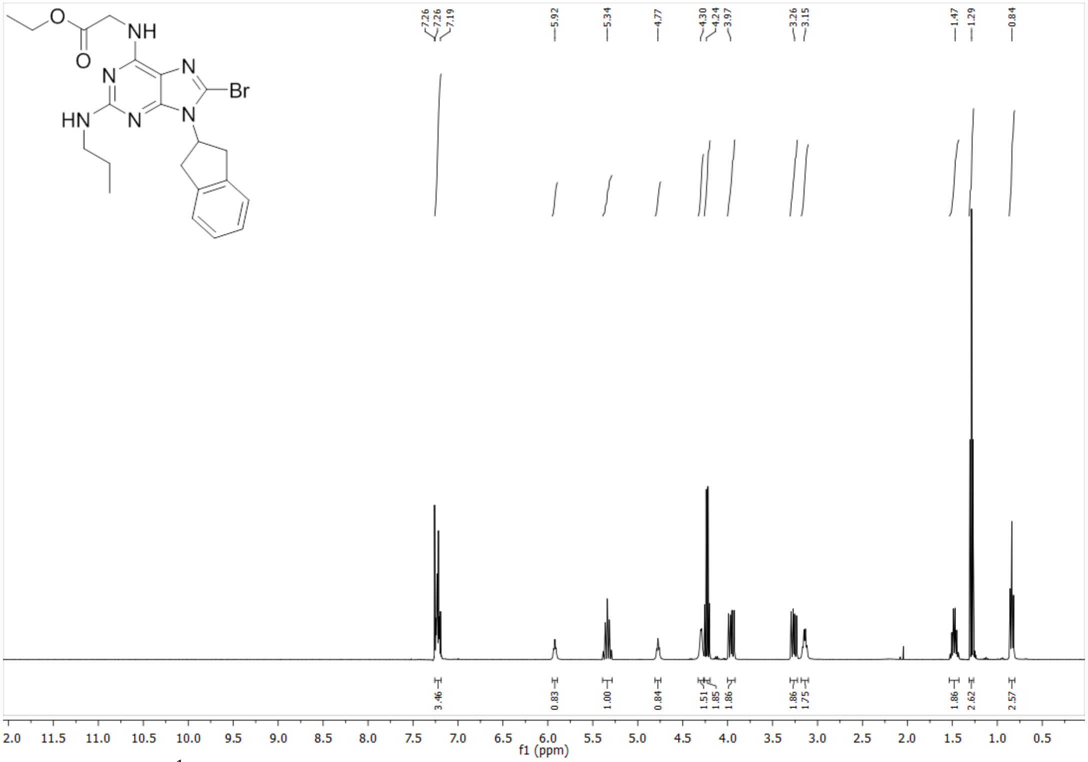

Fig. A66. ^{1}H NMR spectrum of 8-bromo-6-(ethoxy-carbonyl-methylamino)-9-(2-indanyl)-2-(1-propylamino)purine (**12e**).

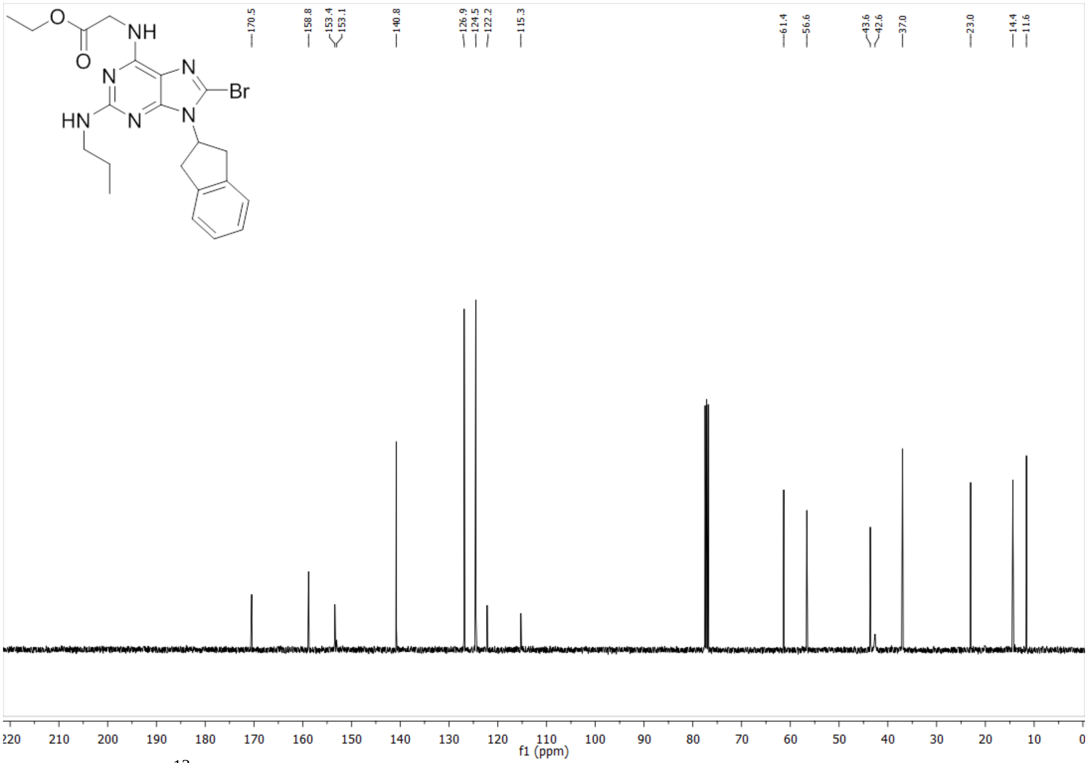

Fig. A67. ^{13}C NMR spectrum of 8-bromo-6-(ethoxy-carbonyl-methylamino)-9-(2-indanyl)-2-(1-propylamino)purine (**12e**).

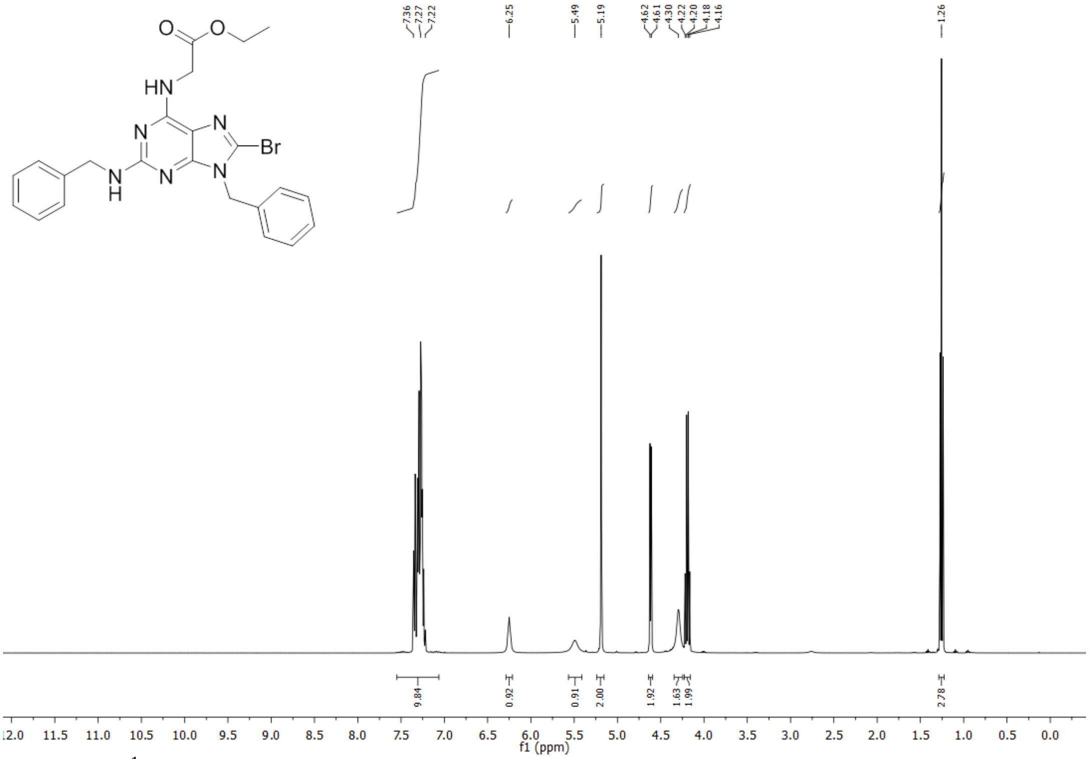

Fig. A68. ^{1}H NMR spectrum of 9-benzyl-2-benzylamino-8-bromo-6-(ethoxy-carbonyl-methylamino)-purine (**12f**).

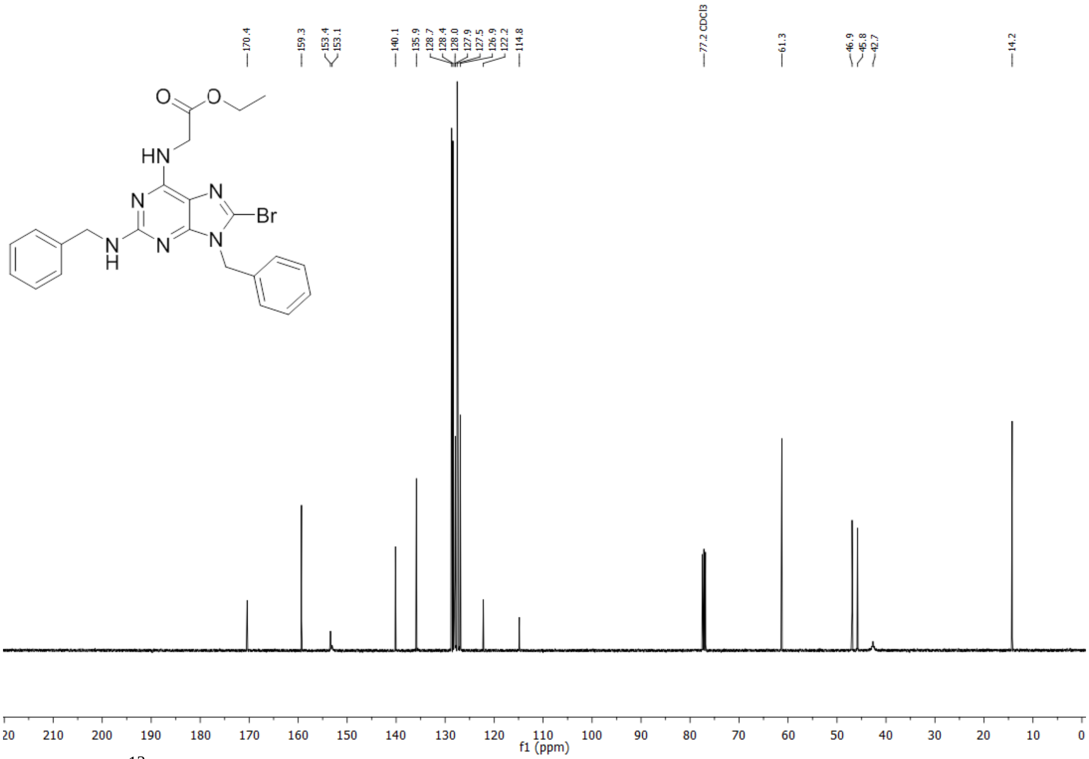

Fig. A69. ^{13}C NMR spectrum of 9-benzyl-2-benzylamino-8-bromo-6-(ethoxy-carbonyl-methylamino)-purine (**12f**).

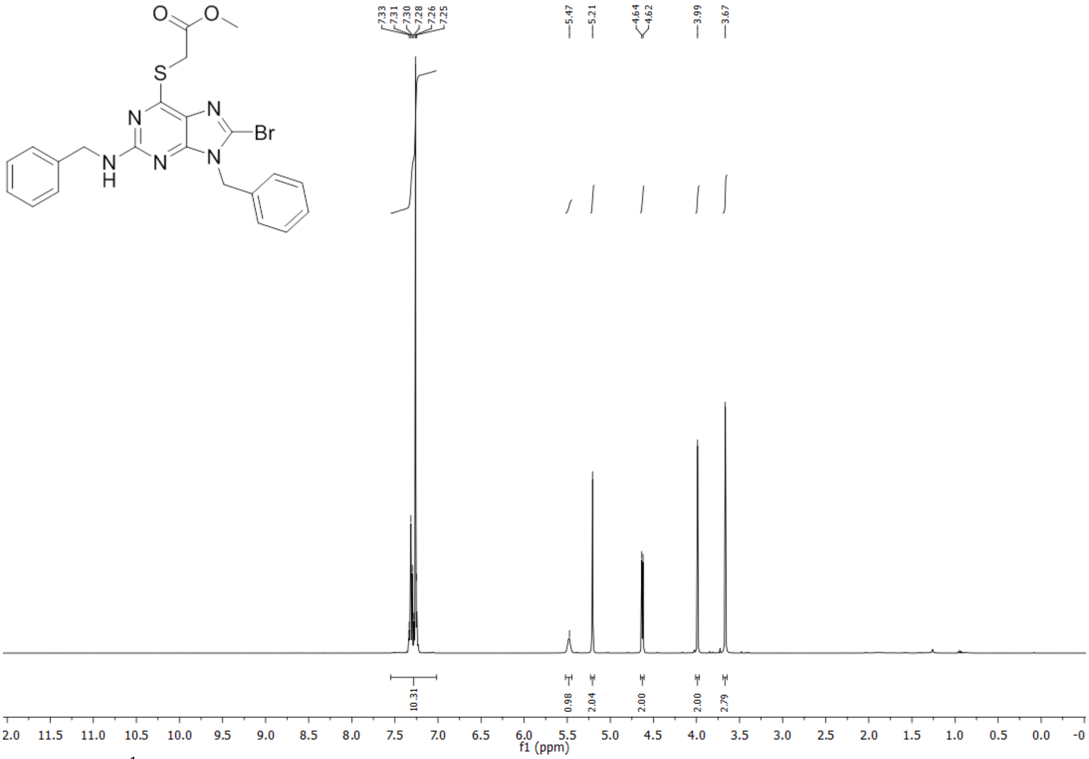

Fig. A70. ^{1}H NMR spectrum of 9-benzyl-2-benzylamino-8-bromo-6-(methoxy-carbonyl-methyl-thio)-purine (**12g**).

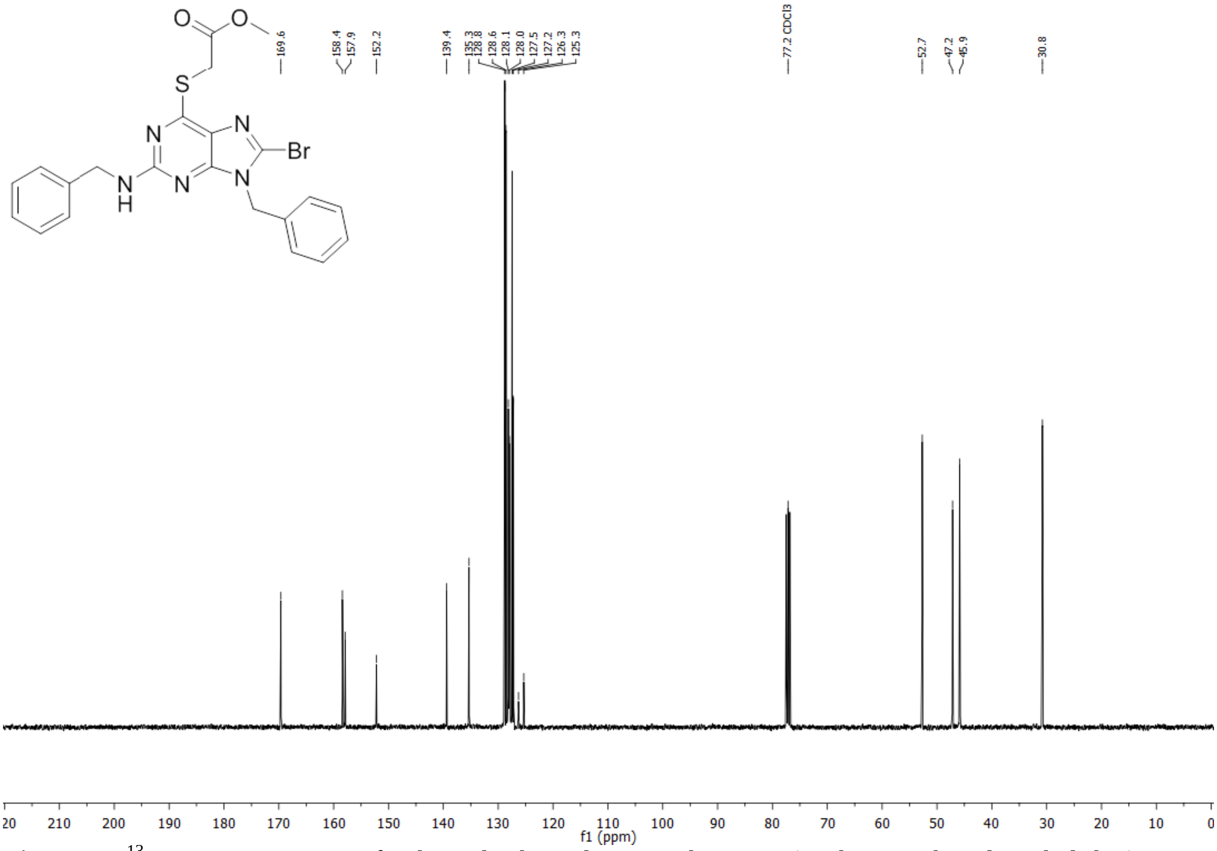

Fig. A71. ^{13}C NMR spectrum of 9-benzyl-2-benzylamino-8-bromo-6-(methoxy-carbonyl-methyl-thio)-purine (**12g**).

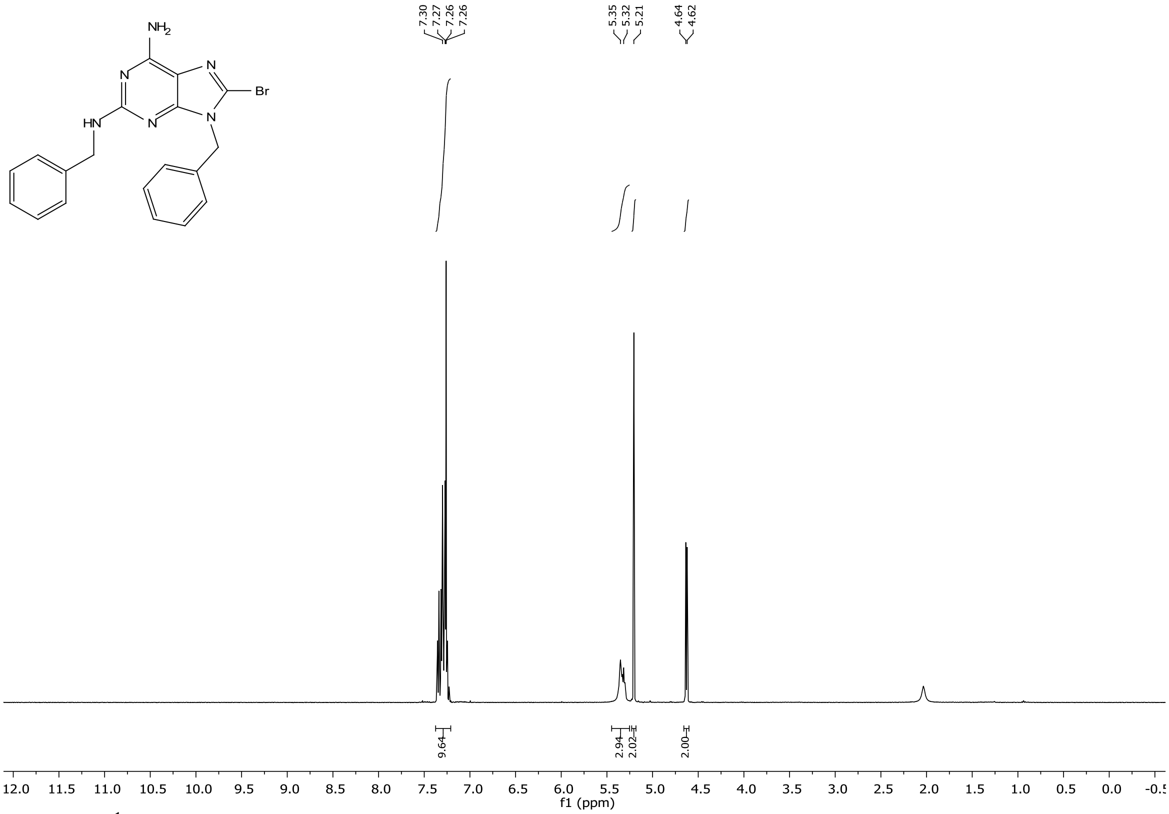

Fig. A72. ^{1}H NMR spectrum of 6-amino-9-benzyl-2-benzylamino-8-bromopurine (**12h**).

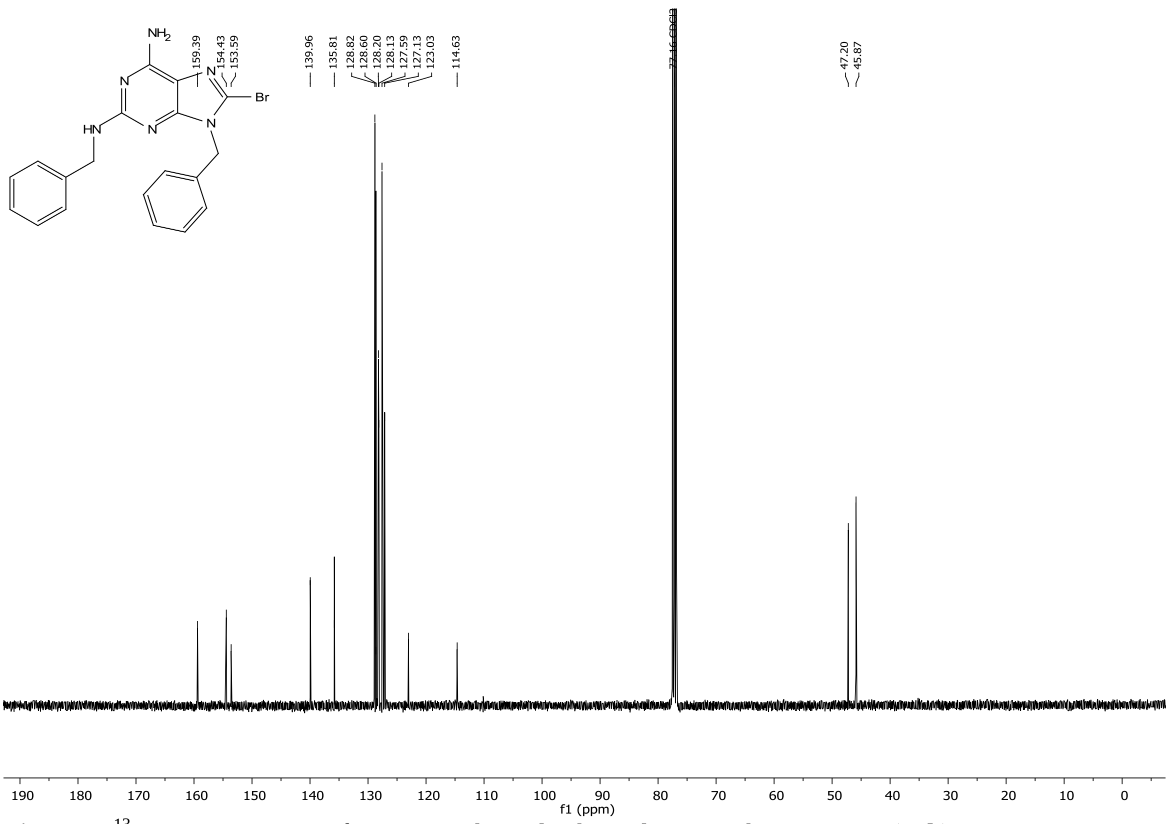

Fig. A73. ^{13}C NMR spectrum of 6-amino-9-benzyl-2-benzylamino-8-bromopurine (**12h**).

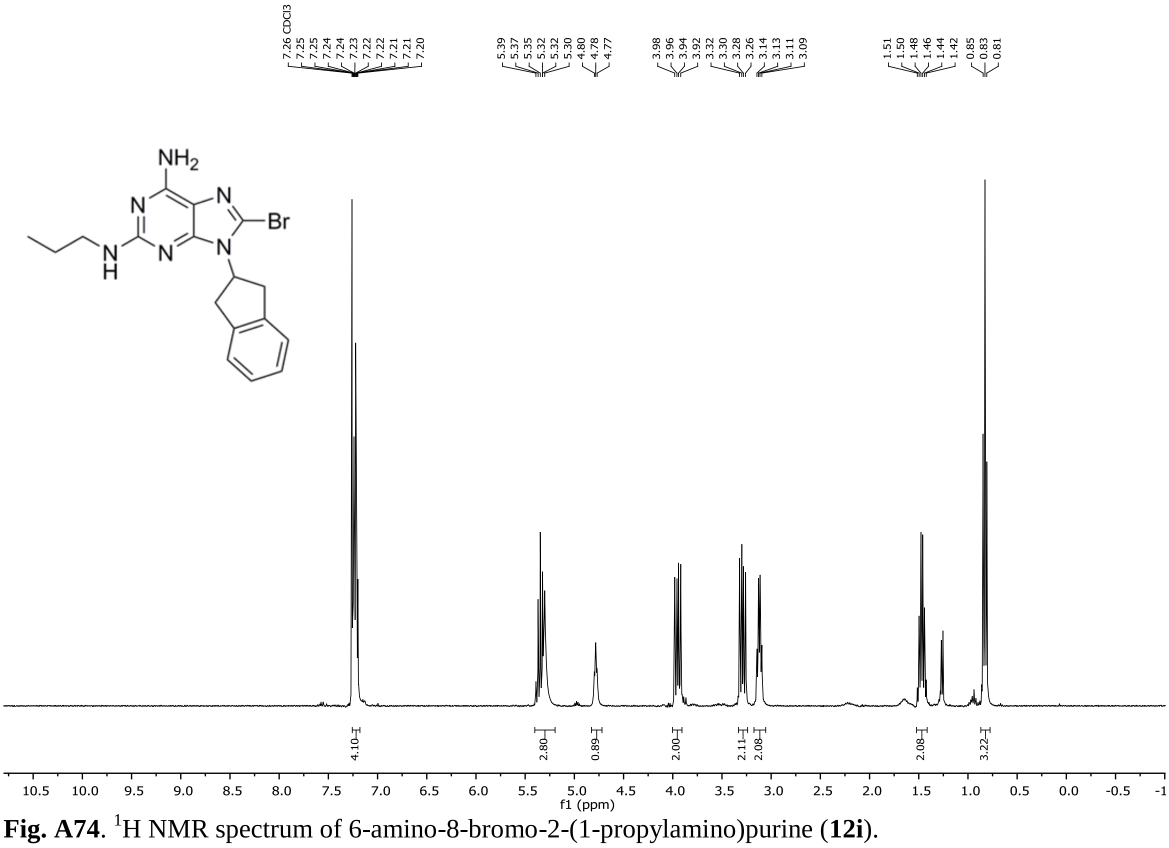

Fig. A74. ^{1}H NMR spectrum of 6-amino-8-bromo-2-(1-propylamino)purine (**12i**).

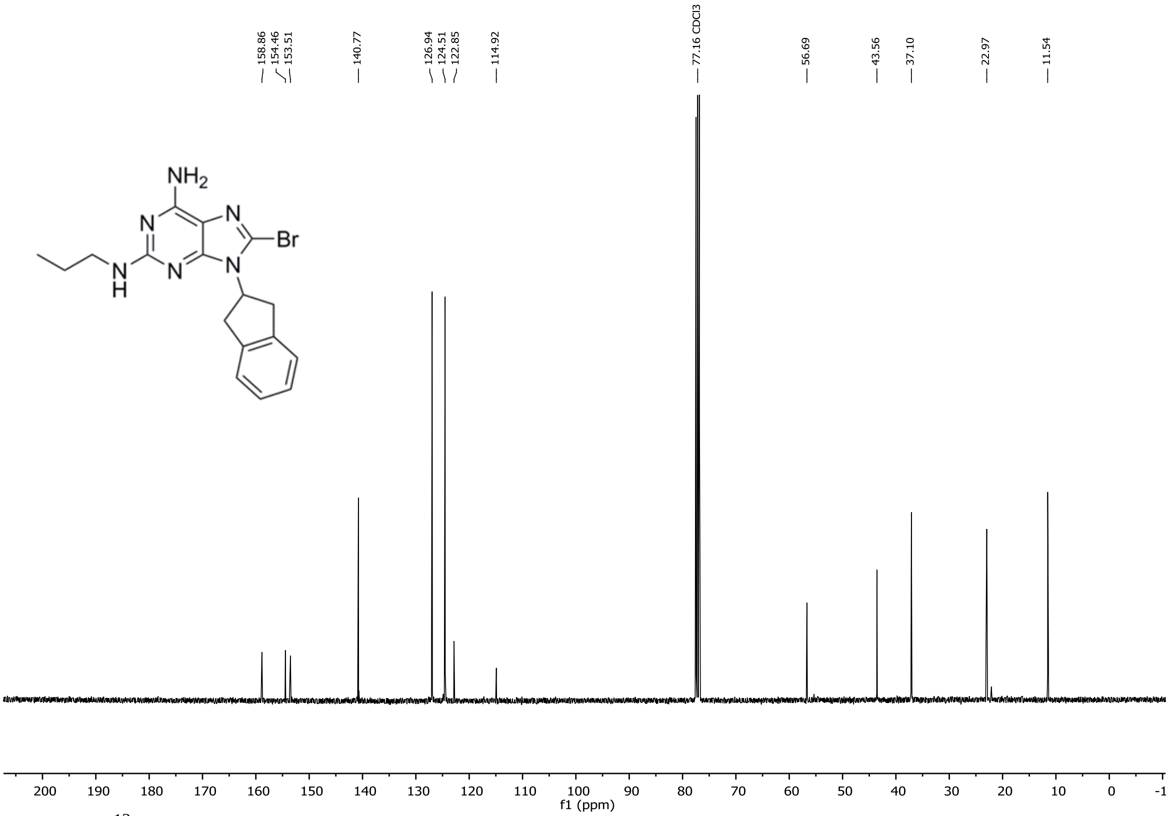

Fig. A75. ^{13}C NMR spectrum of 6-amino-8-bromo-2-(1-propylamino)purine (**12i**).

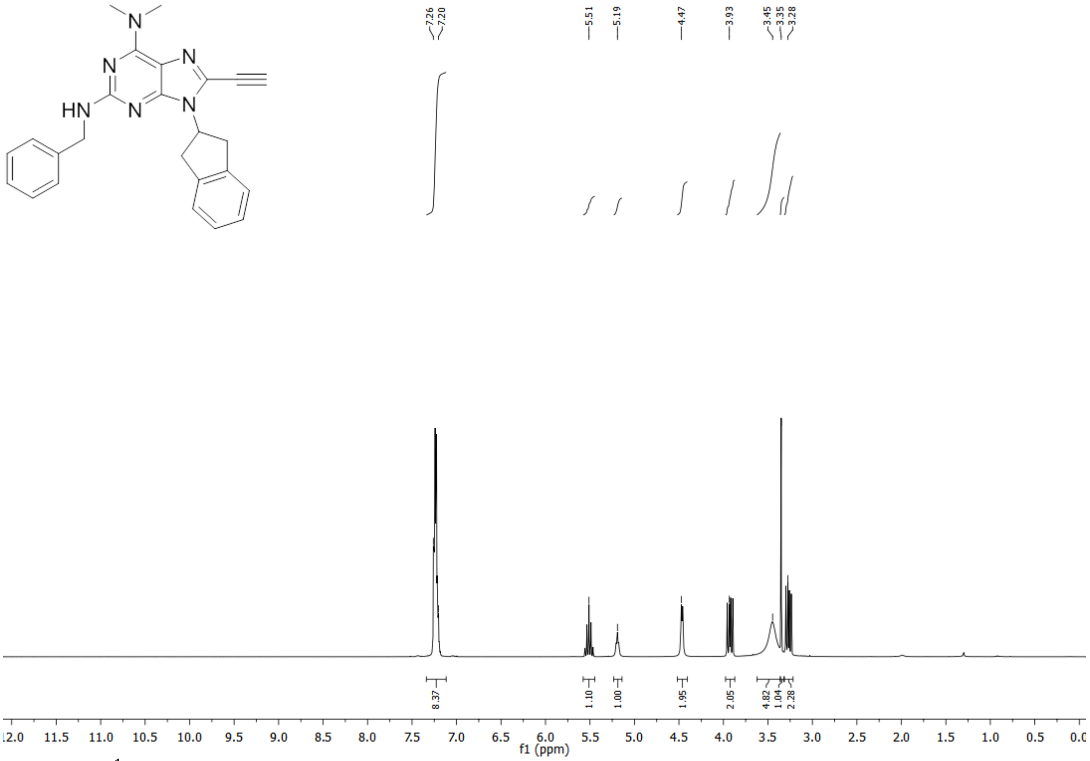

Fig. A76. ^{1}H NMR spectrum of 2-benzylamino-6-dimethylamino-8-ethynyl-9-(2-indanyl)purine (**13a**).

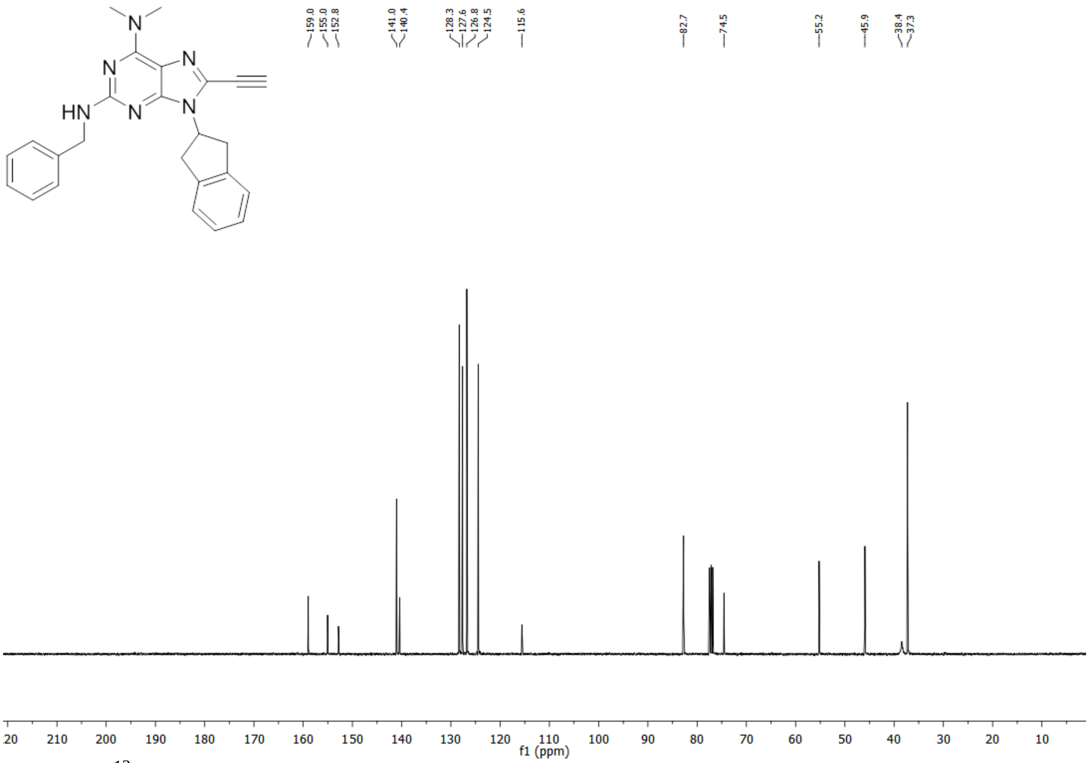

Fig. A77. ^{13}C NMR spectrum of 2-benzylamino-6-dimethylamino-8-ethynyl-9-(2-indanyl)purine (**13a**).

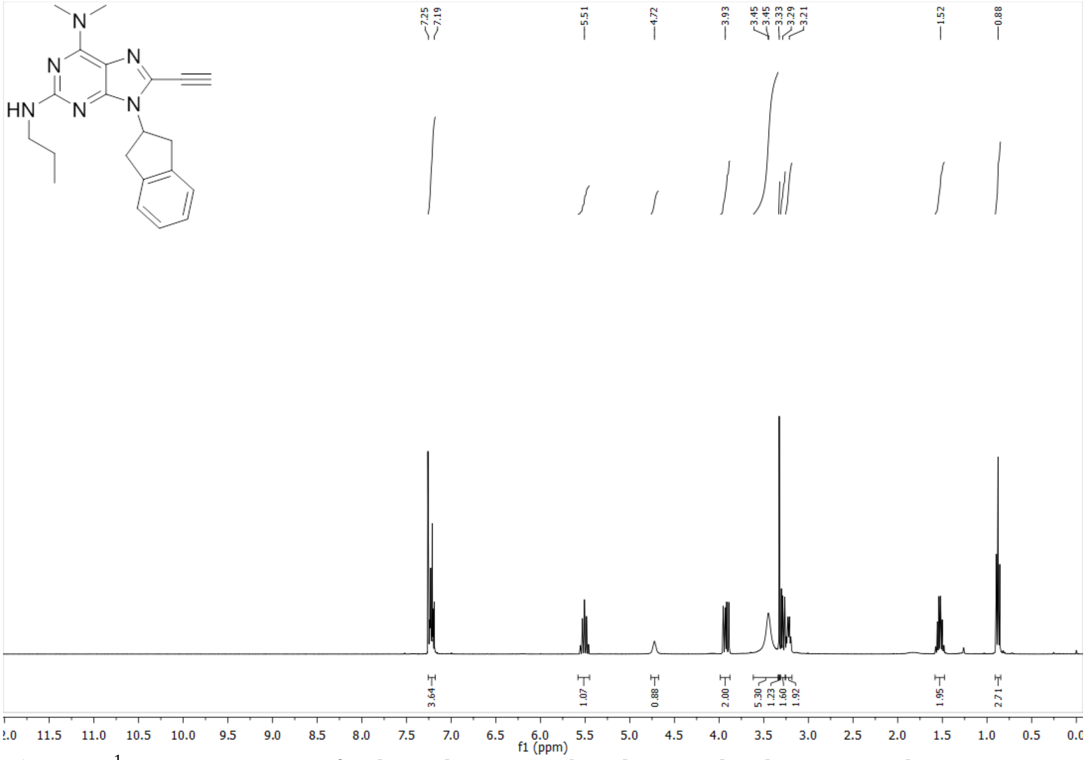

Fig. A78. ^{1}H NMR spectrum of 6-dimetylamino-8-ethynyl-9-(2-indanyl)-2-(1-propylamino)purine (**13b**).

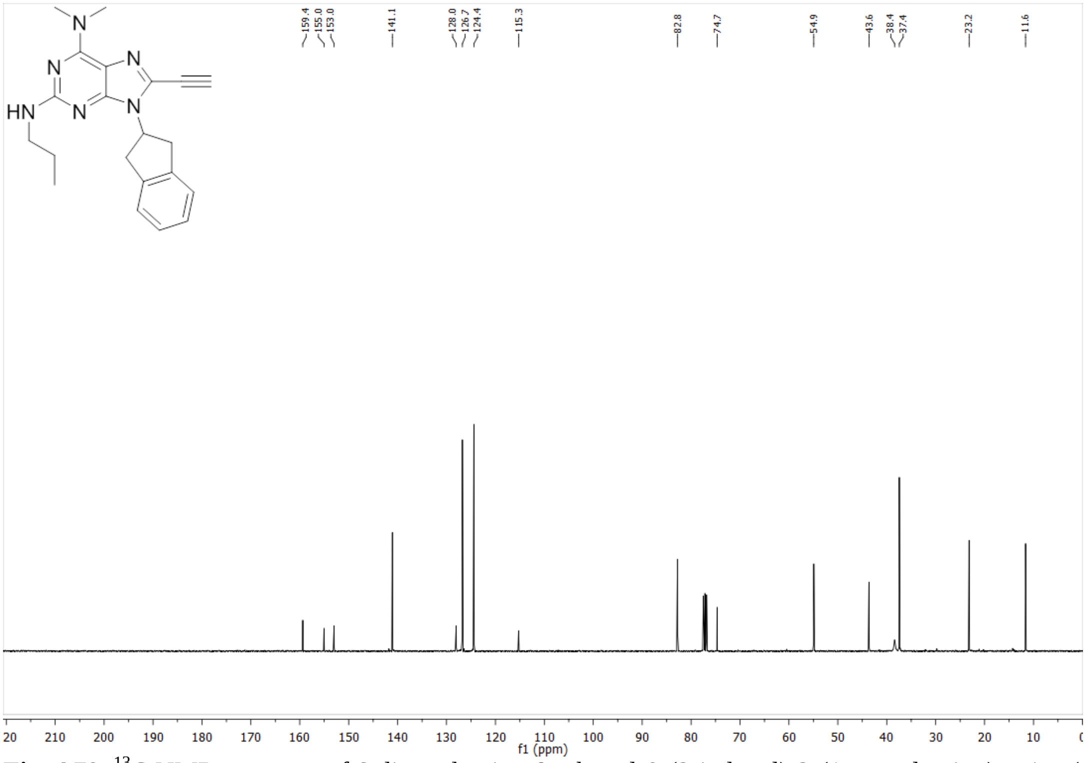

Fig. A79. ^{13}C NMR spectrum of 6-dimetylamino-8-ethynyl-9-(2-indanyl)-2-(1-propylamino)purine (**13b**).

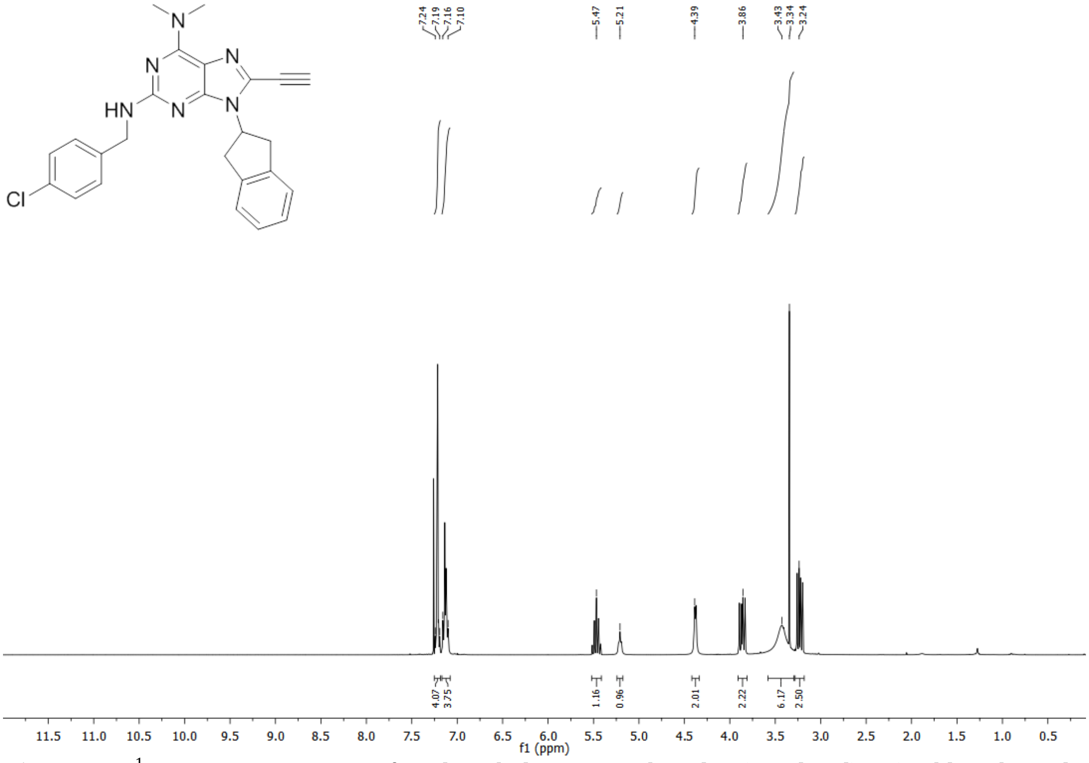

Fig. A80. ^{1}H NMR spectrum of 6-dimethylamino-8-ethynyl-9-(2-indanyl)-2-(*p*-chloro-benzylamino)purine (**13c**).

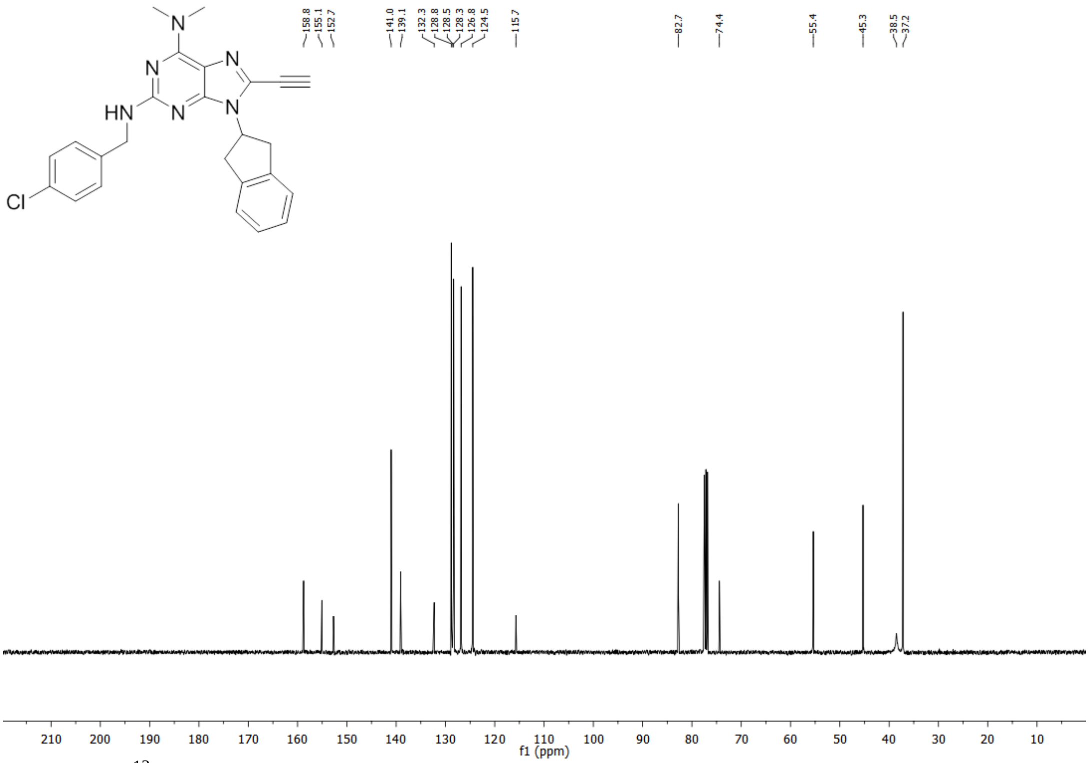

Fig. A81. ^{13}C NMR spectrum of 6-dimethylamino-8-ethynyl-9-(2-indanyl)-2-(*p*-chloro-benzylamino)purine (**13c**).

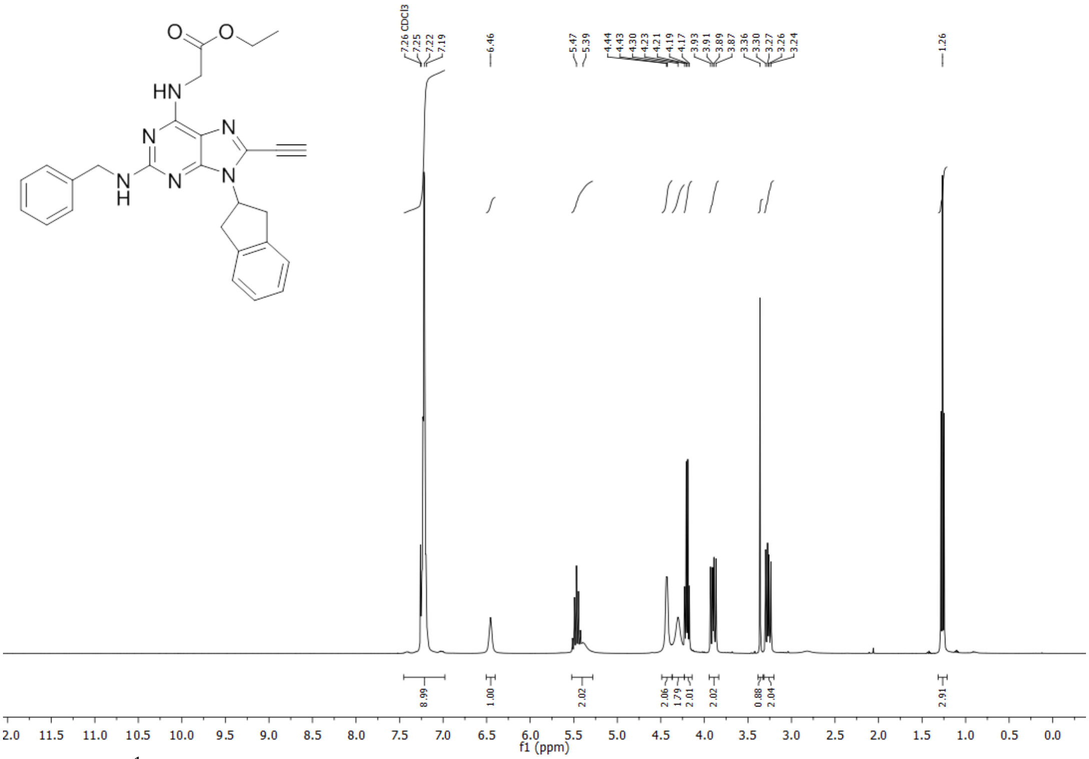

Fig. A82. ^{1}H NMR spectrum of 2-benzylamino-6-(ethoxy-carbonyl-methylamino)-8-ethynyl-9-(2-indanyl)-purine (**13d**).

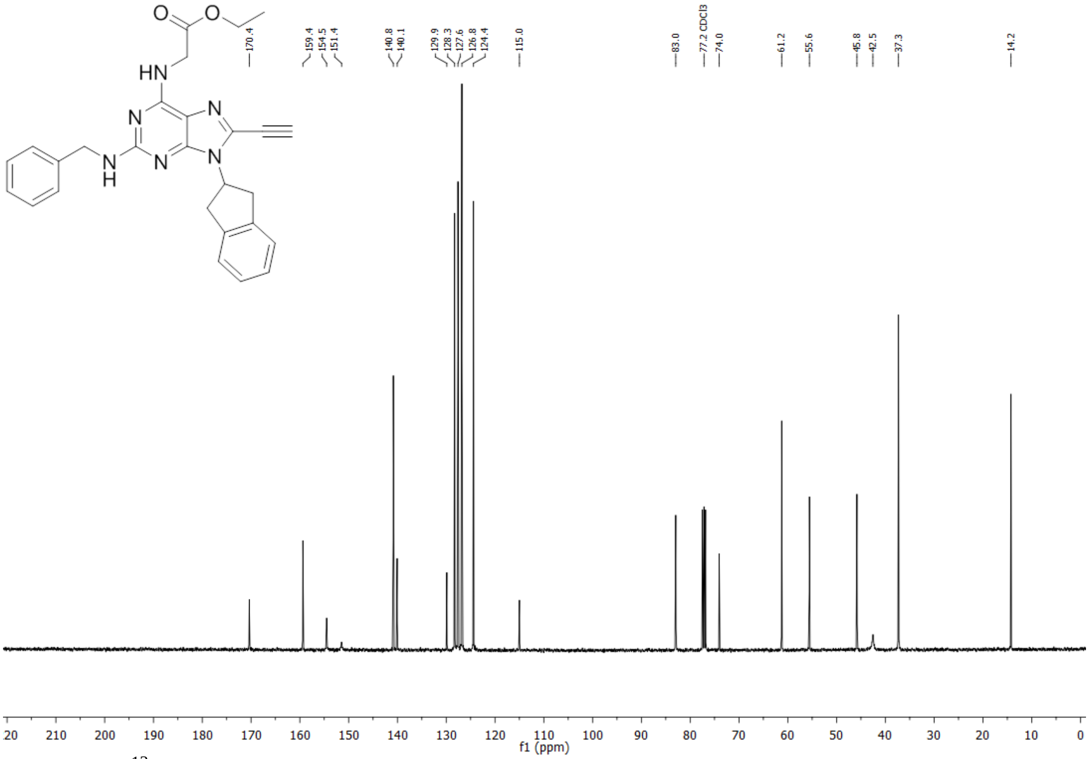

Fig. A83. ^{13}C NMR spectrum of 2-benzylamino-6-(ethoxy-carbonyl-methylamino)-8-ethynyl-9-(2-indanyl)-purine (**13d**).

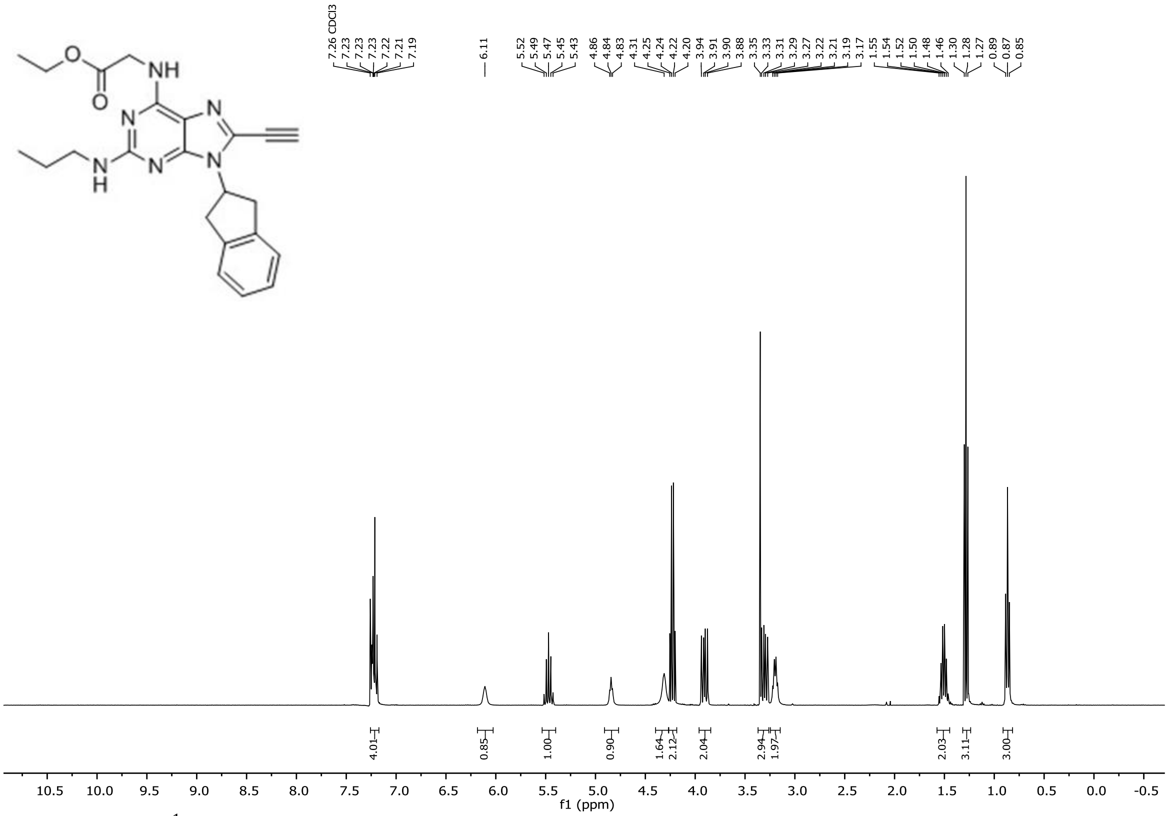

Fig. A84. ^{1}H NMR spectrum of 6-(ethoxy-carbonyl-methylamino)-8-ethynyl-9-(2-indanyl)-2-(1-propylamino)purine (**13e**).

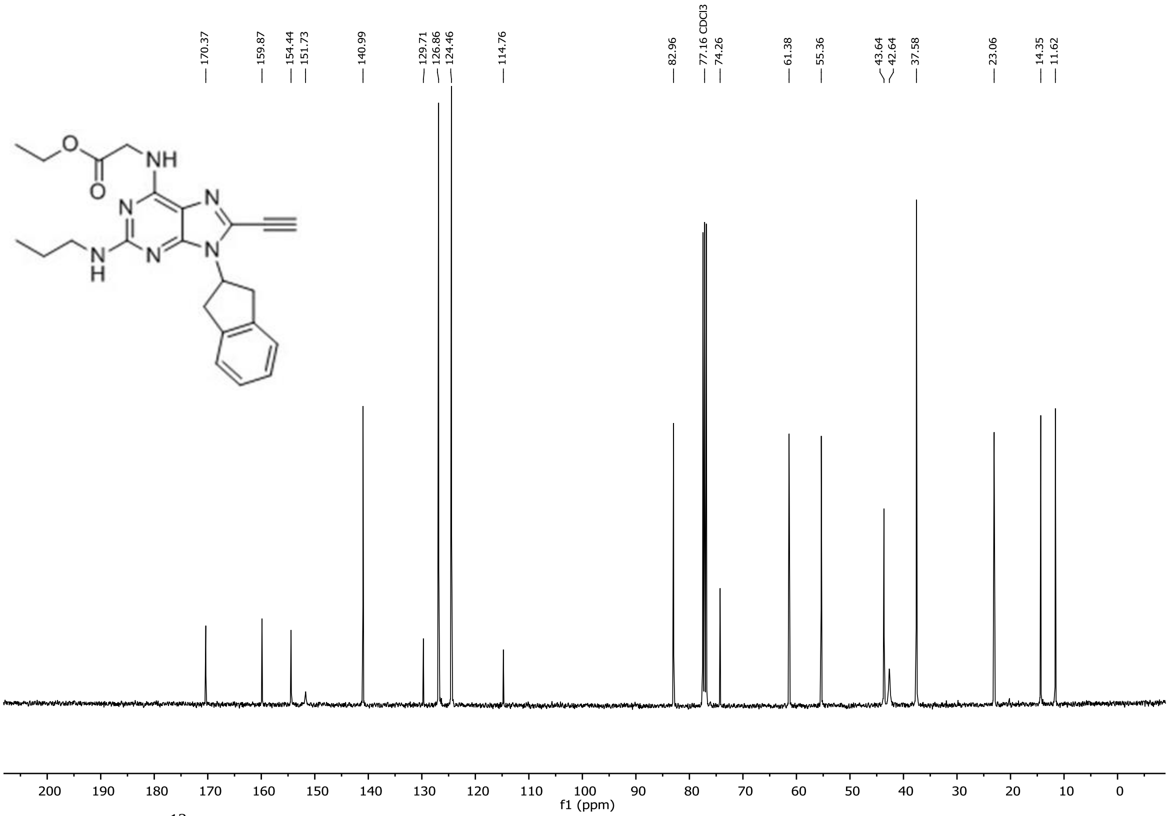

Fig. A85. ^{13}C NMR spectrum of 6-(ethoxy-carbonyl-methylamino)-8-ethynyl-9-(2-indanyl)-2-(1-propylamino)purine (**13e**).

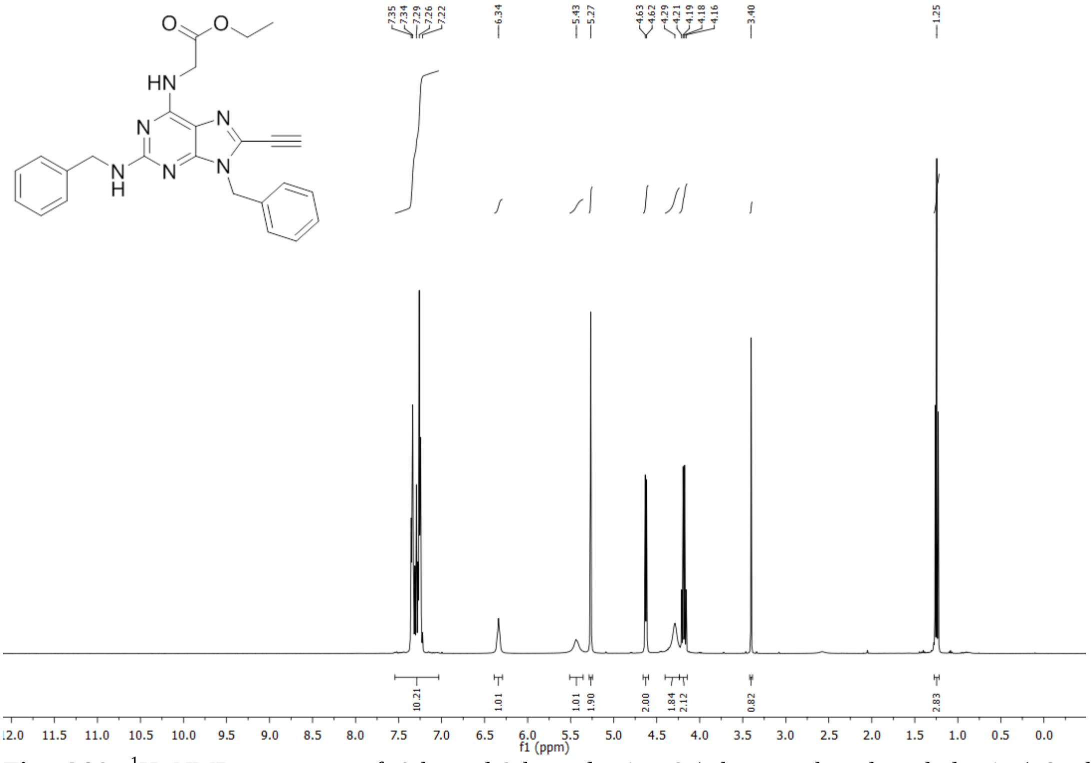

Fig. A86. ^{1}H NMR spectrum of 9-benzyl-2-benzylamino-6-(ethoxy-carbonyl-methylamino)-8-ethynylpurine (**13f**).

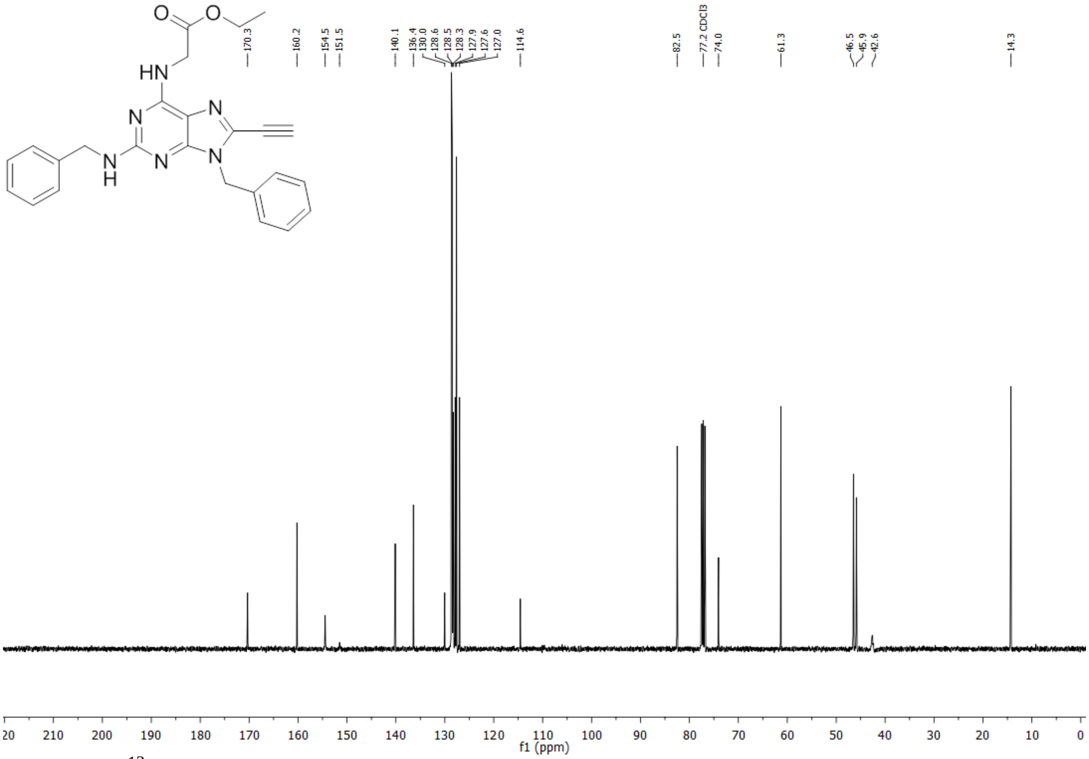

Fig. A87. ^{13}C NMR spectrum of 9-benzyl-2-benzylamino-6-(ethoxy-carbonyl-methylamino)-8-ethynylpurine (**13f**).

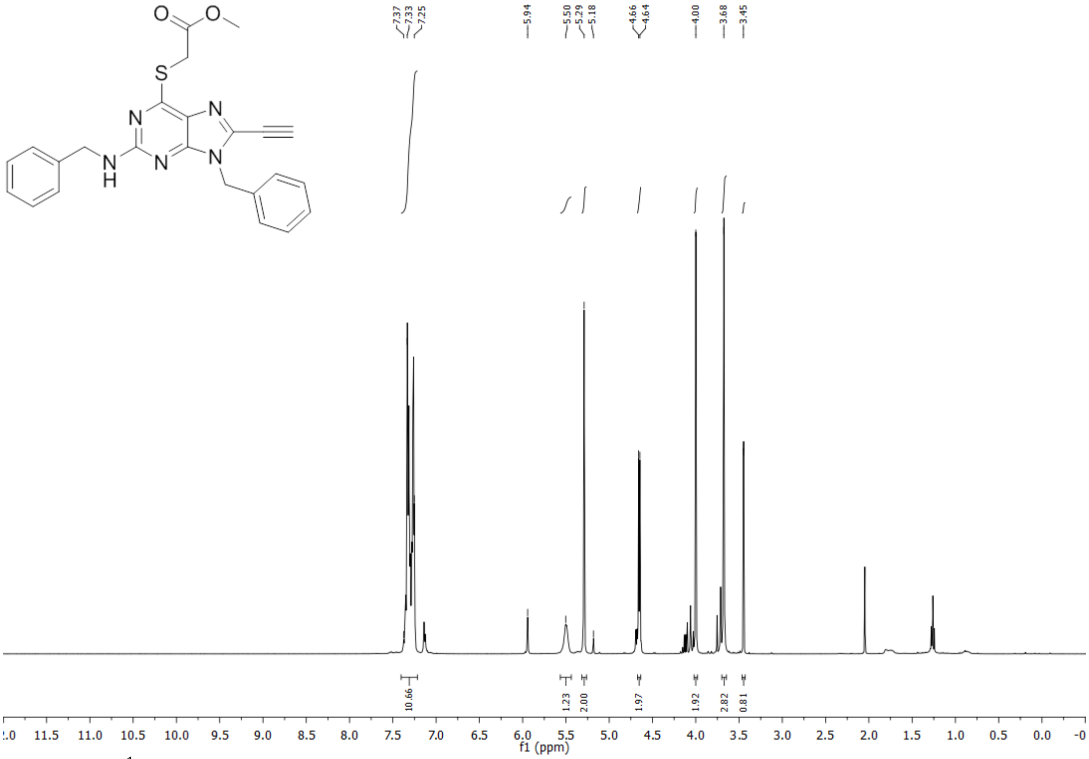

Fig. A88. ^{1}H NMR spectrum of 9-benzyl-2-benzylamino-8-ethynyl-6-(methoxy-carbonyl-methyl-thio)-purine (**13g**).

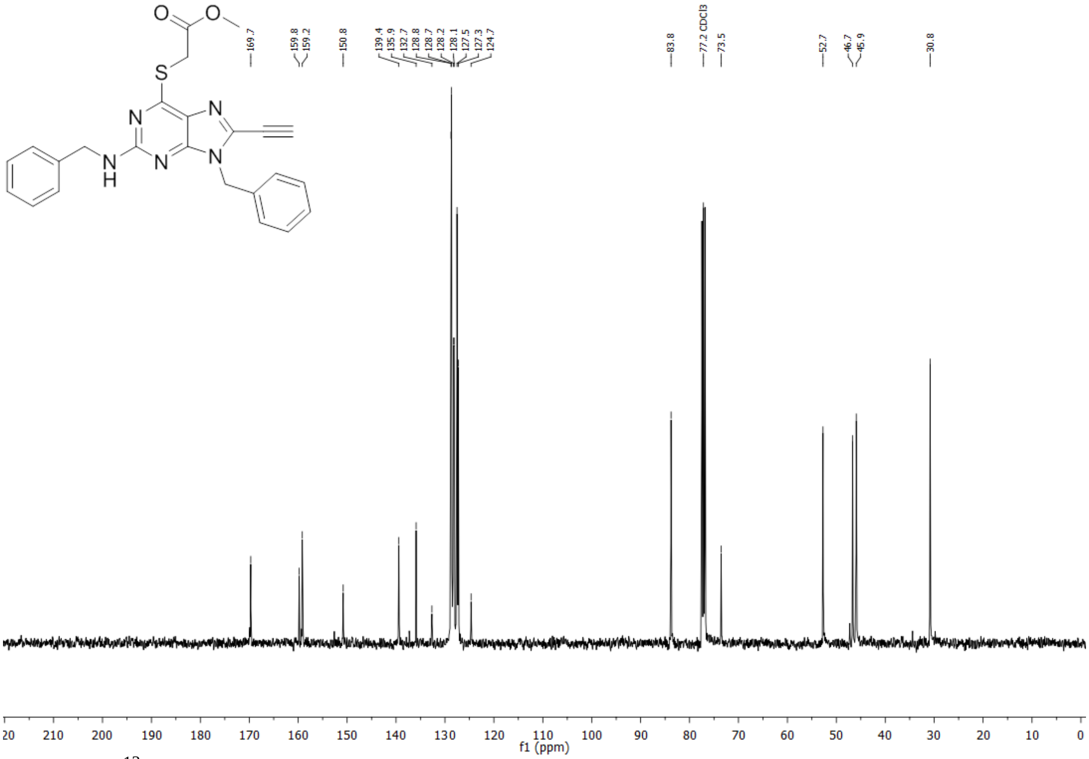

Fig. A89. ^{13}C NMR spectrum of 9-benzyl-2-benzylamino-8-ethynyl-6-(methoxy-carbonyl-methyl-thio)-purine (**13g**).

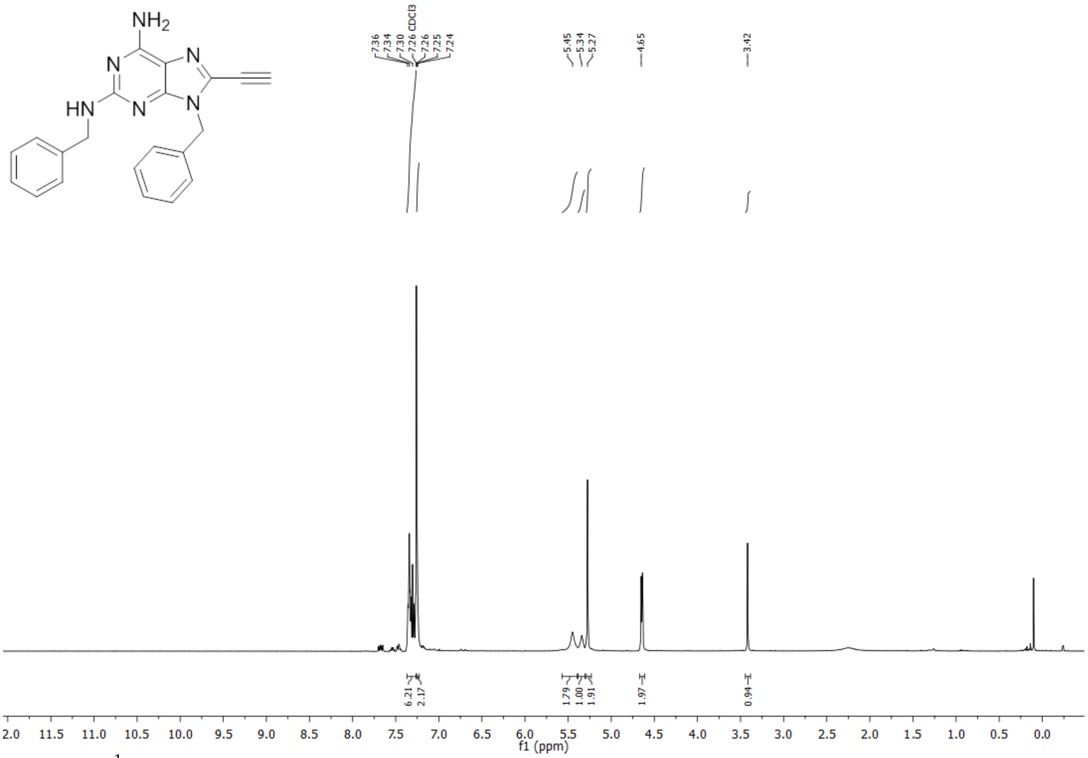

Fig. A90. ^{1}H NMR spectrum of 6-amino-9-benzyl-2-benzylamino-8-ethynylpurine (**13h**).

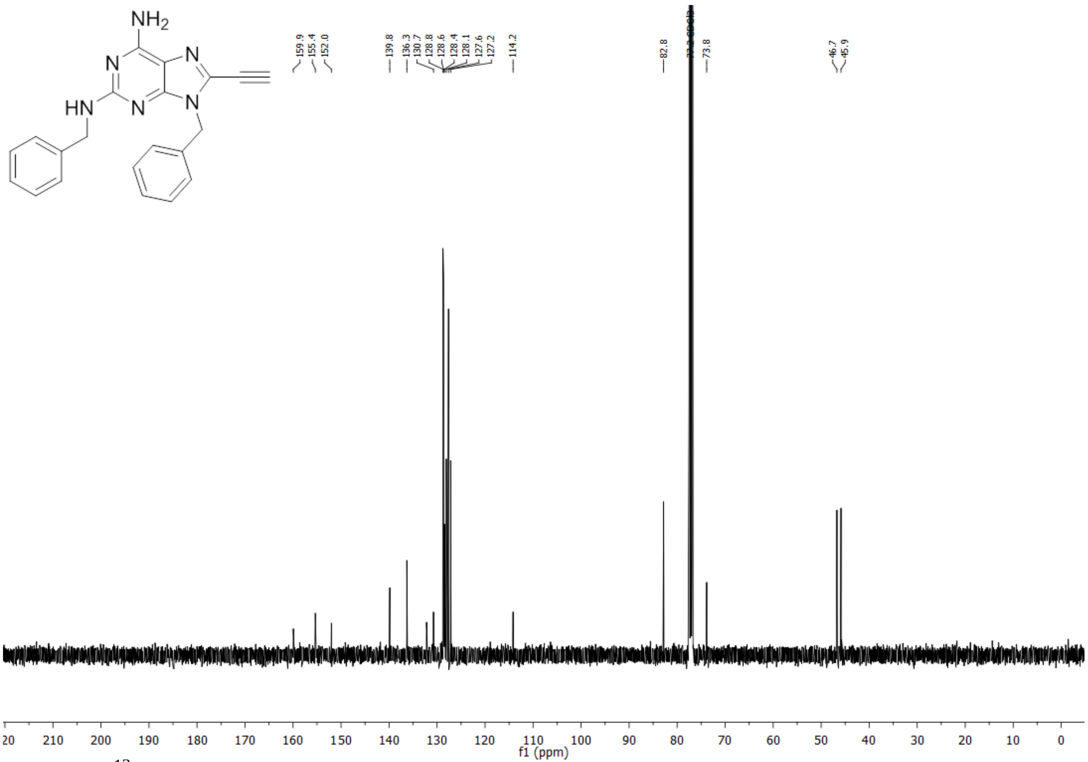

Fig. A91. ^{13}C NMR spectrum of 6-amino-9-benzyl-2-benzylamino-8-ethynylpurine (**13h**).

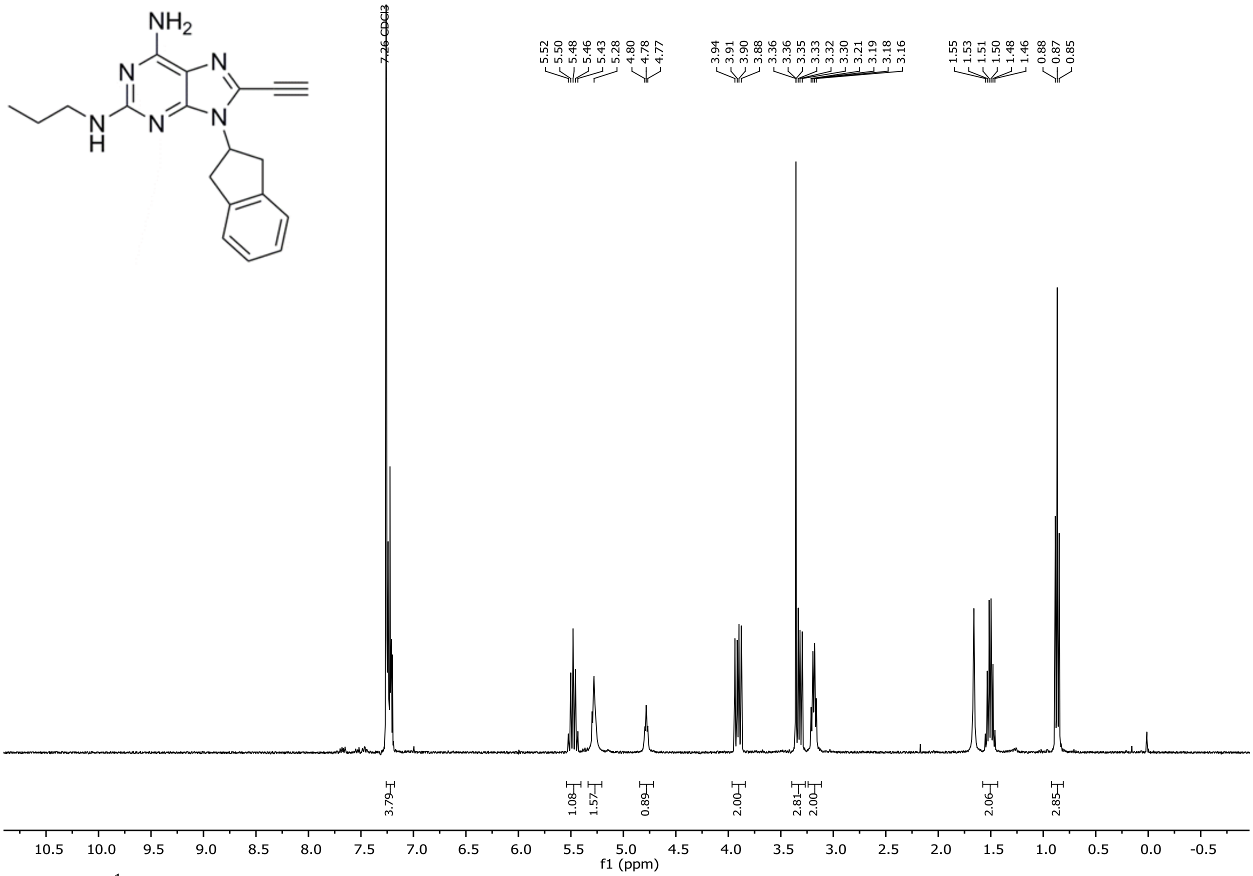

Fig. A92. ^{1}H NMR spectrum of 6-amino-8-ethynyl-9-(2-indanyl)-2-(1-propylamino)purine (**13i**).

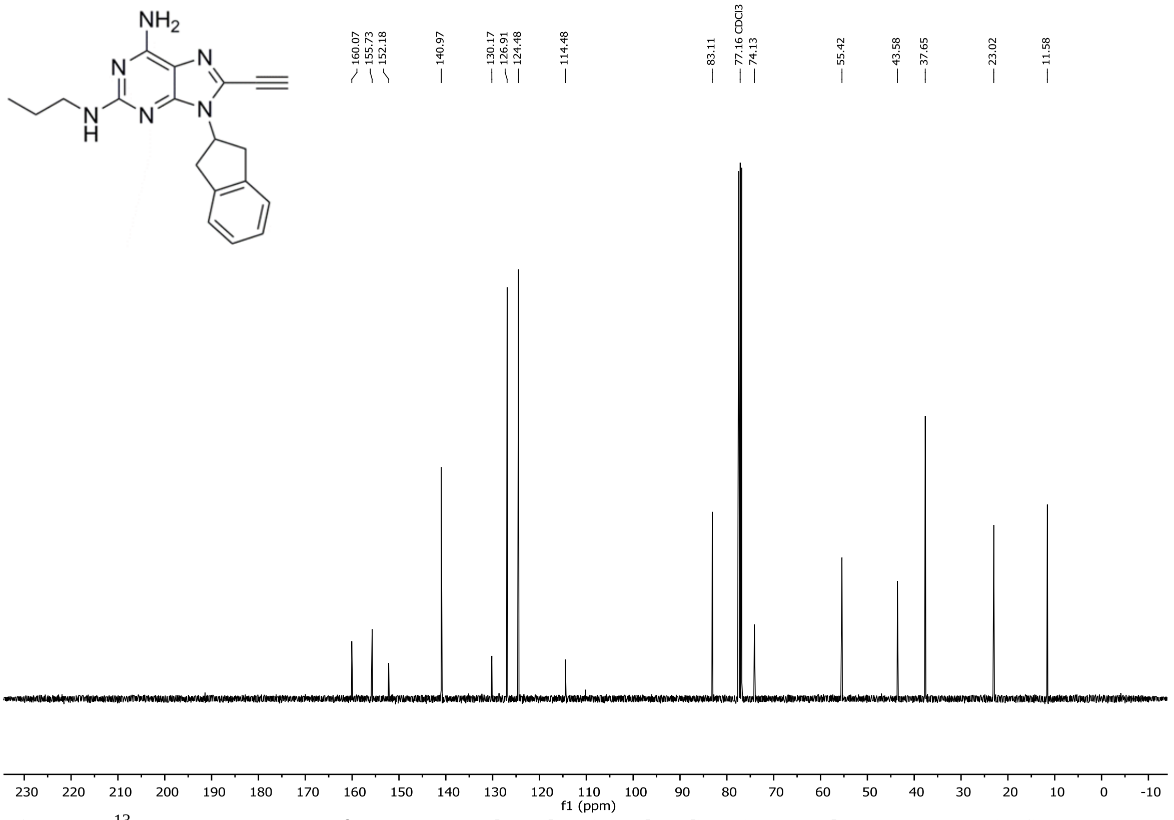

Fig. A93. ^{13}C NMR spectrum of 6-amino-8-ethynyl-9-(2-indanyl)-2-(1-propylamino)purine (**13i**).

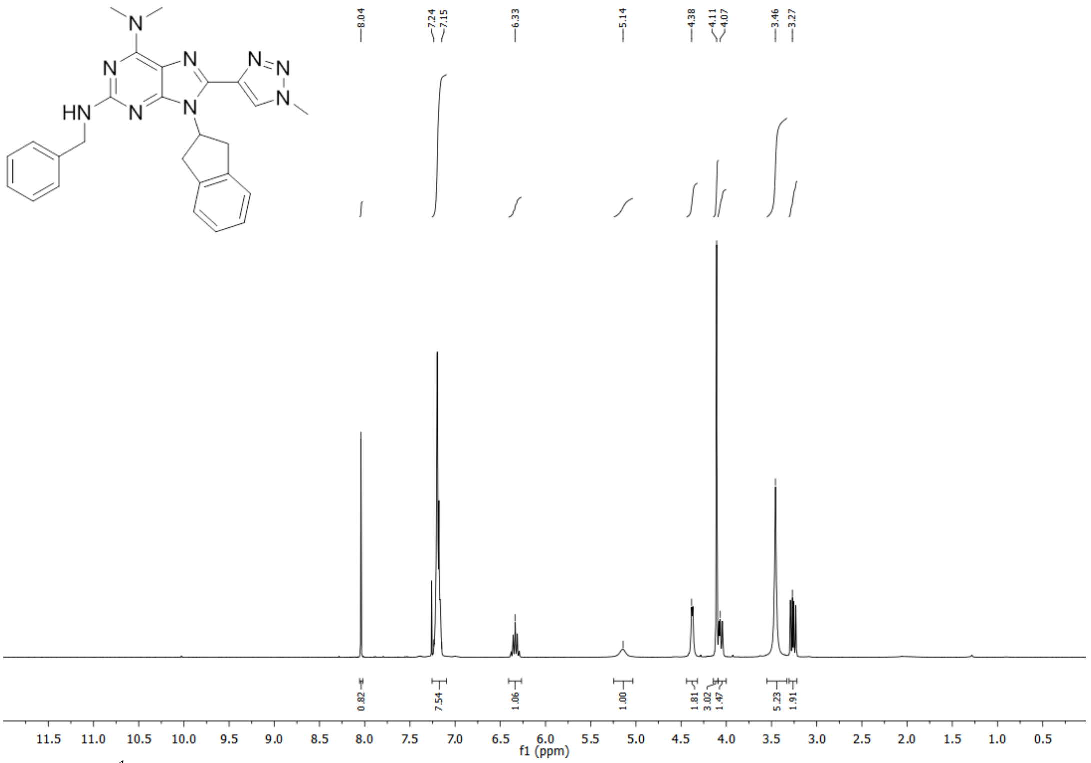

Fig. A94. ^{1}H NMR spectrum of 2-benzylamino-6-dimethylamino-9-(2-indanyl)-8-(1-methyl-1H-1,2,3-triazol-4-yl)-purine (**14a**).

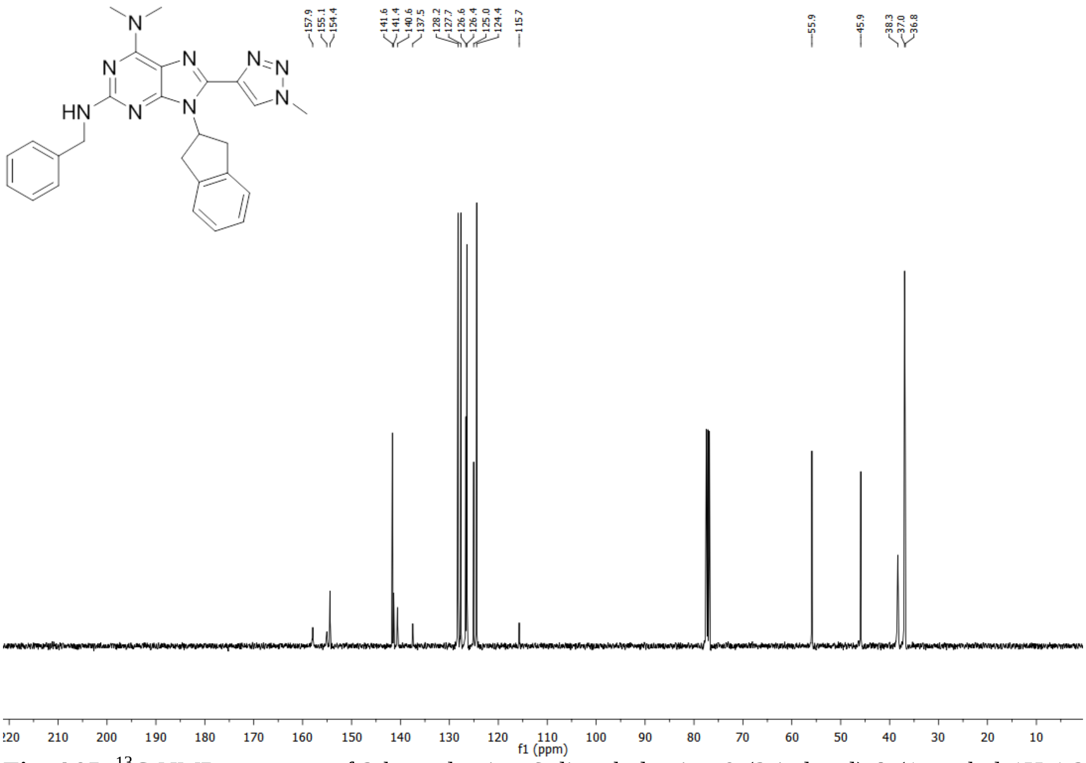

Fig. A95. ^{13}C NMR spectrum of 2-benzylamino-6-dimethylamino-9-(2-indanyl)-8-(1-methyl-1H-1,2,3-triazol-4-yl)-purine (**14a**).

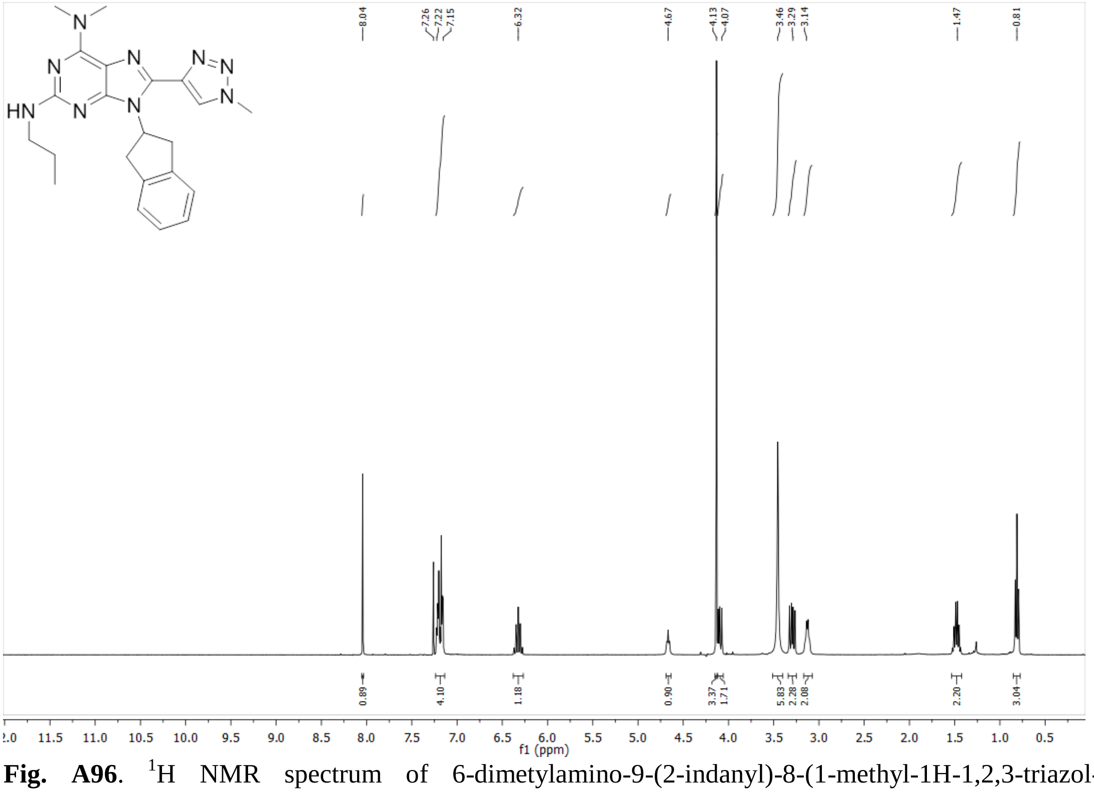

Fig. A96. ^{1}H NMR spectrum of 6-dimetylamino-9-(2-indanyl)-8-(1-methyl-1H-1,2,3-triazol-4-yl)-2-(1-propylamino)purine (**14b**).

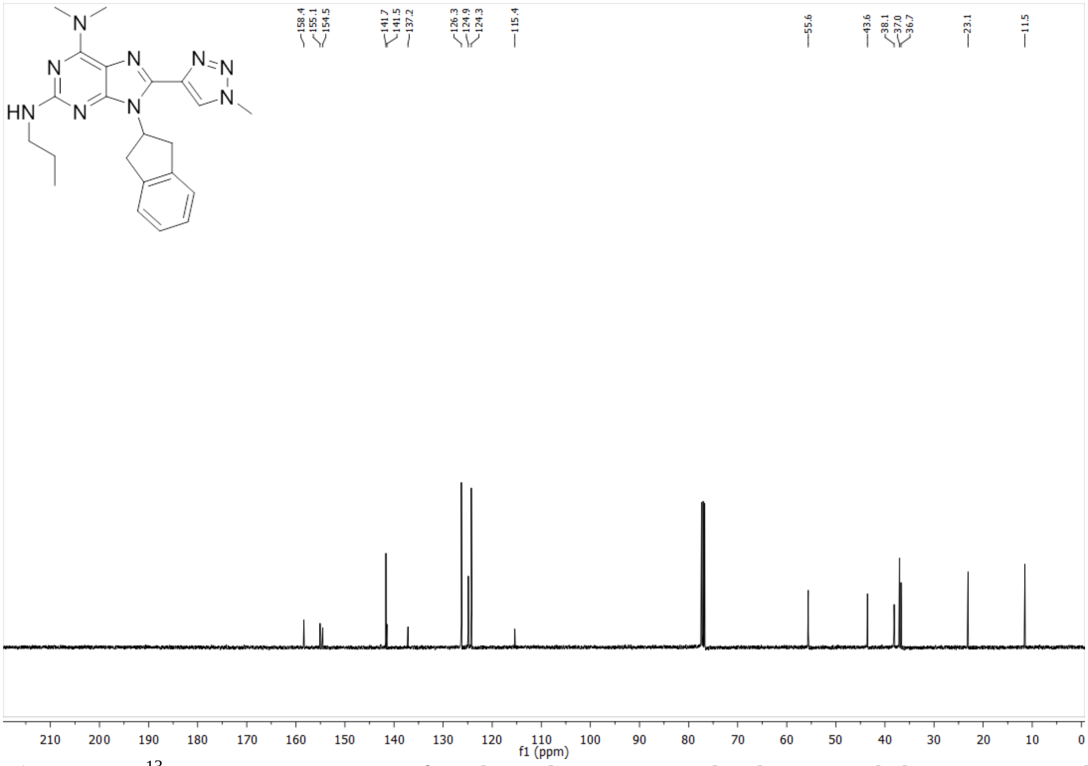

Fig. A97. ^{13}C NMR spectrum of 6-dimetylamino-9-(2-indanyl)-8-(1-methyl-1H-1,2,3-triazol-4-yl)-2-(1-propylamino)purine (**14b**).

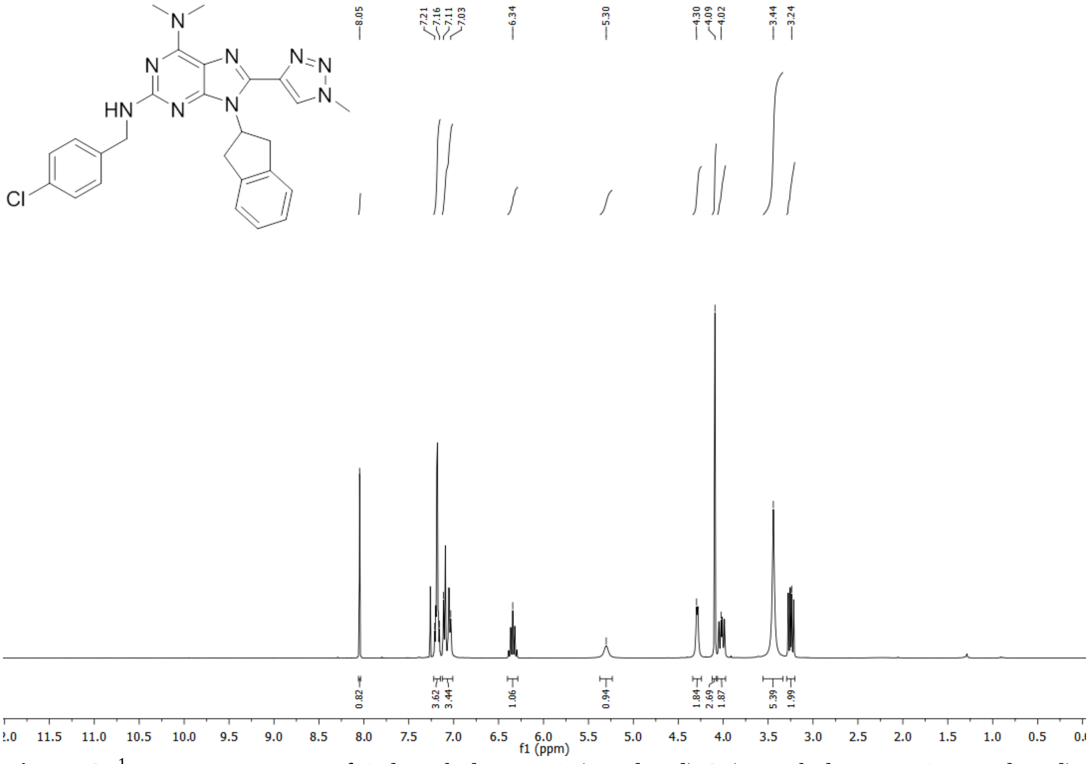

Fig. A98. ^{1}H NMR spectrum of 6-dimethylamino-9-(2-indanyl)-8-(1-methyl-1H-1,2,3-triazol-4-yl)-2-(*p*-chloro-benzylamino)purine (**14c**).

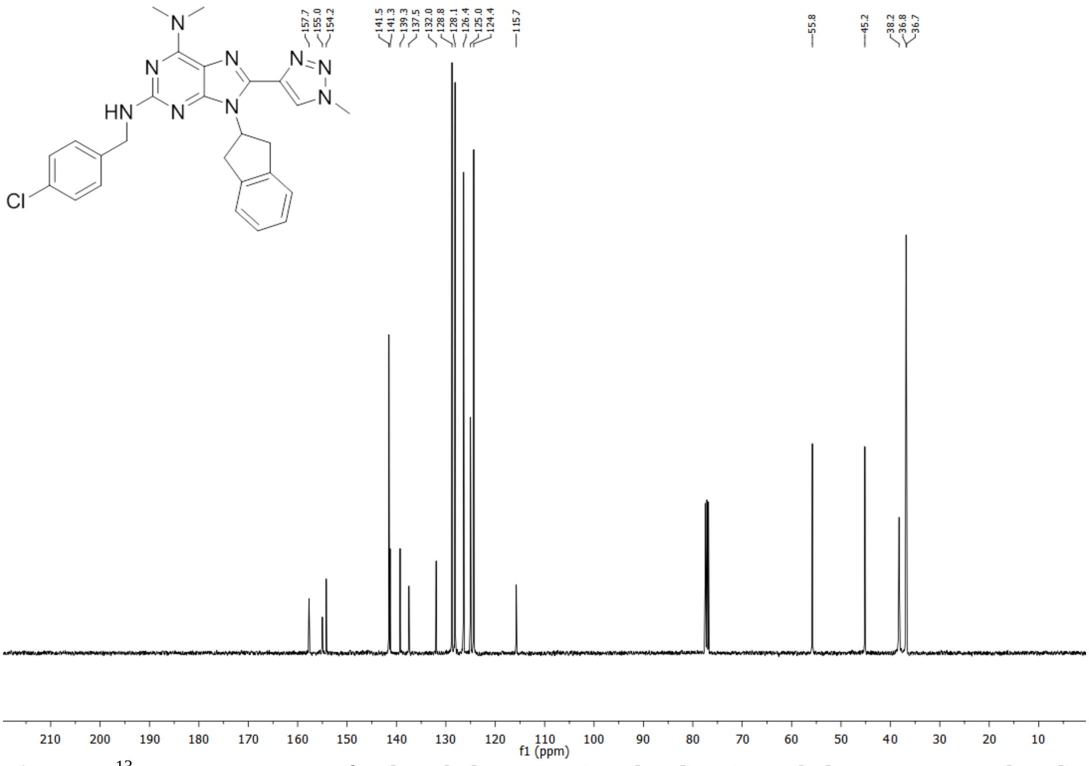

Fig. A99. ^{13}C NMR spectrum of 6-dimethylamino-9-(2-indanyl)-8-(1-methyl-1H-1,2,3-triazol-4-yl)-2-(*p*-chloro-benzylamino)purine (**14c**).

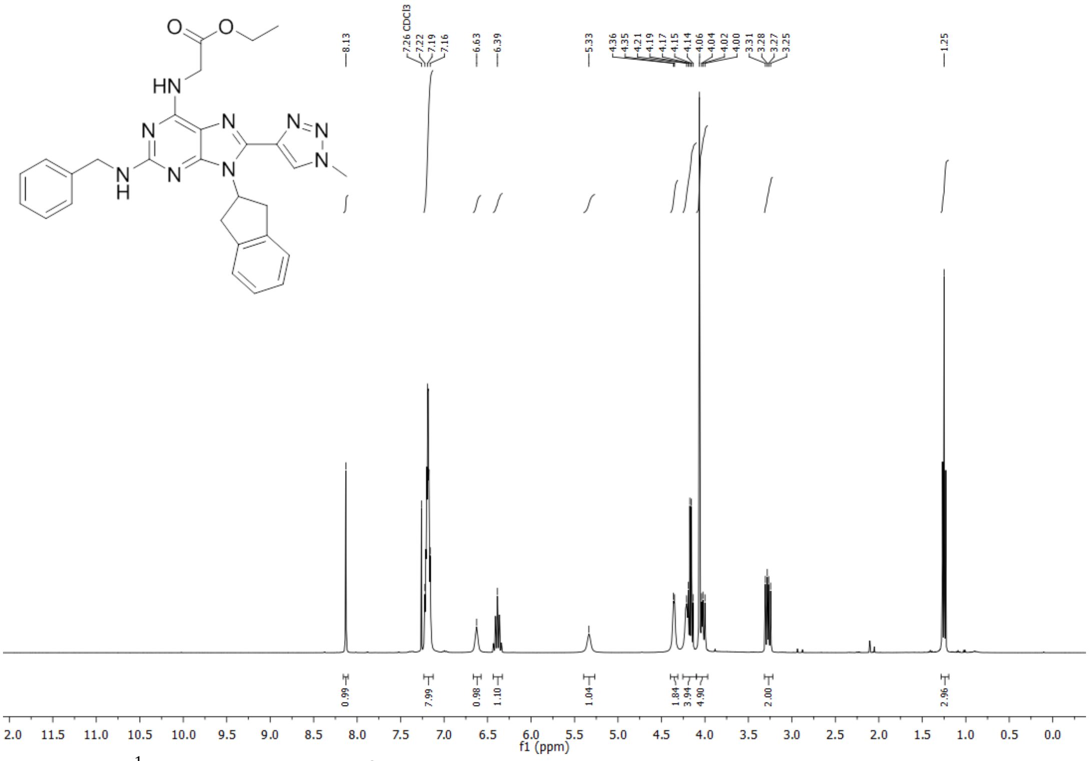

Fig. A100. ^{1}H NMR spectrum of 2-benzylamino-6-(ethoxy-carbonyl-methylamino)-9-(2-indanyl)-8-(1-methyl-1,2,3-triazol-4-yl)-purine (**14d**).

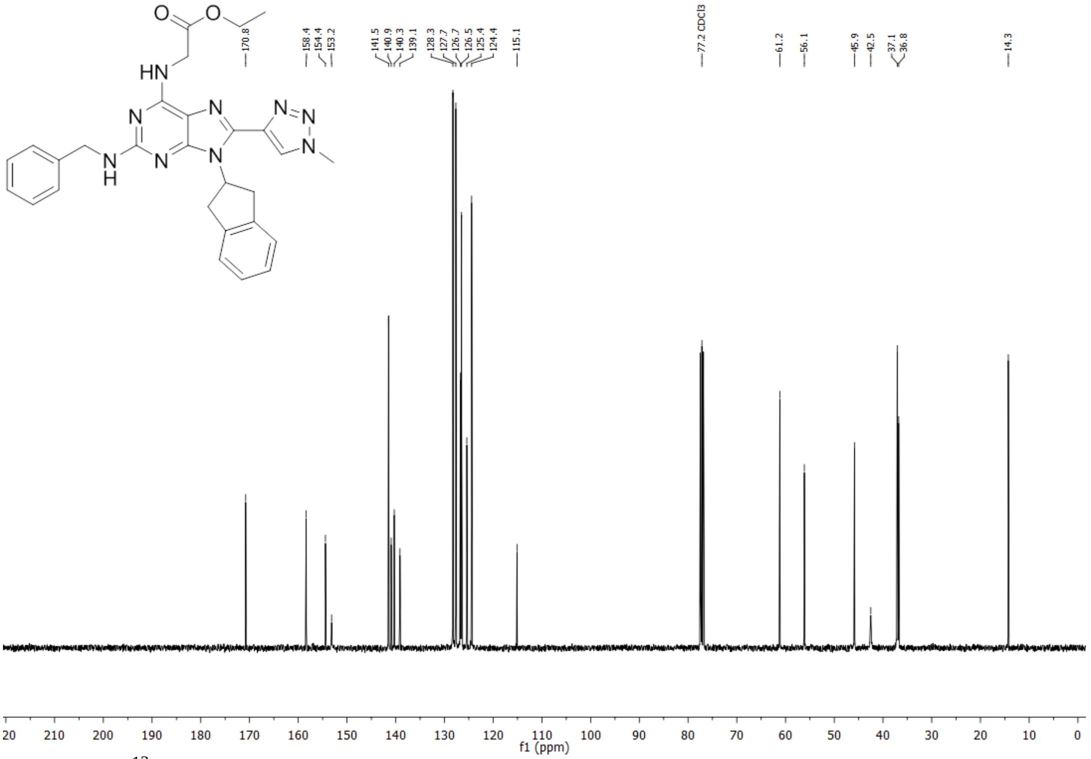

Fig. A101. ^{13}C NMR spectrum of 2-benzylamino-6-(ethoxy-carbonyl-methylamino)-9-(2-indanyl)-8-(1-methyl-1,2,3-triazol-4-yl)-purine (**14d**).

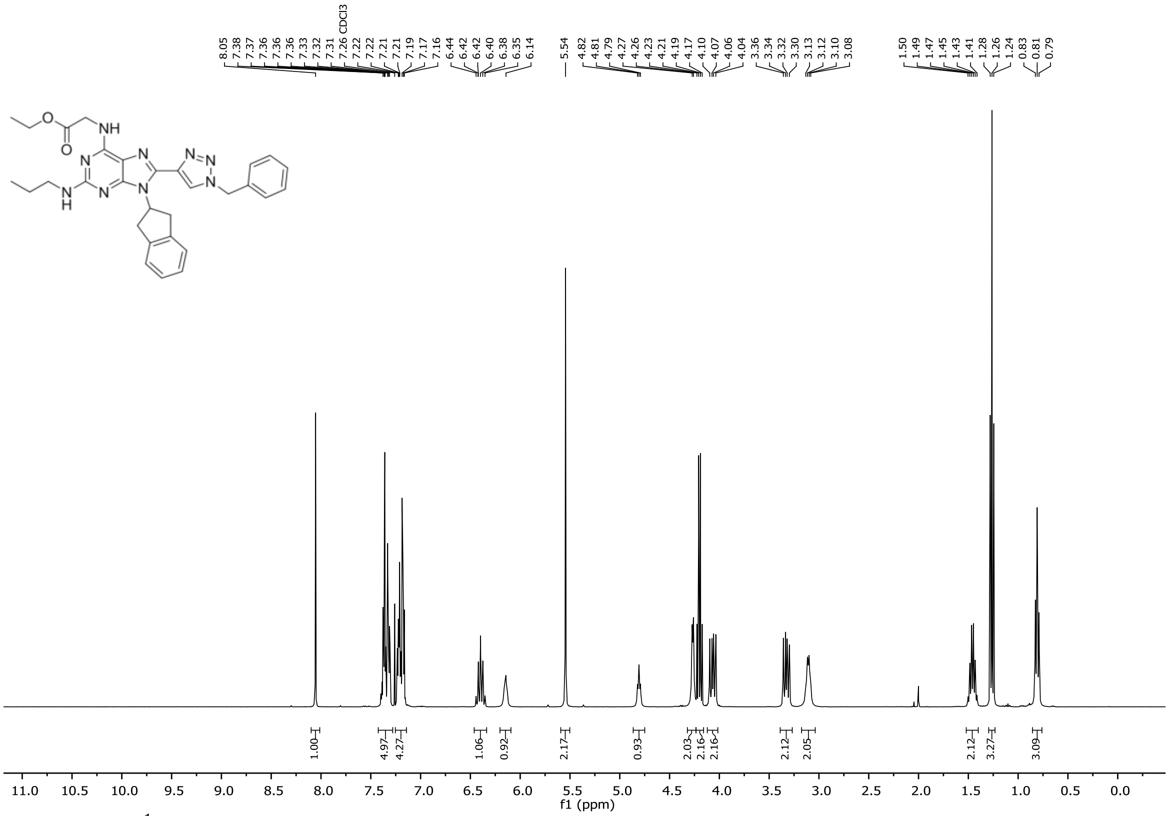

Fig. A102. ^{1}H NMR spectrum of 8-(1-benzyl-1H-1,2,3-triazol-4-yl)-6-(ethoxy-carbonyl-methylamino)-9-(2-indanyl)-2-(1-propylamino)purine (**14e**).

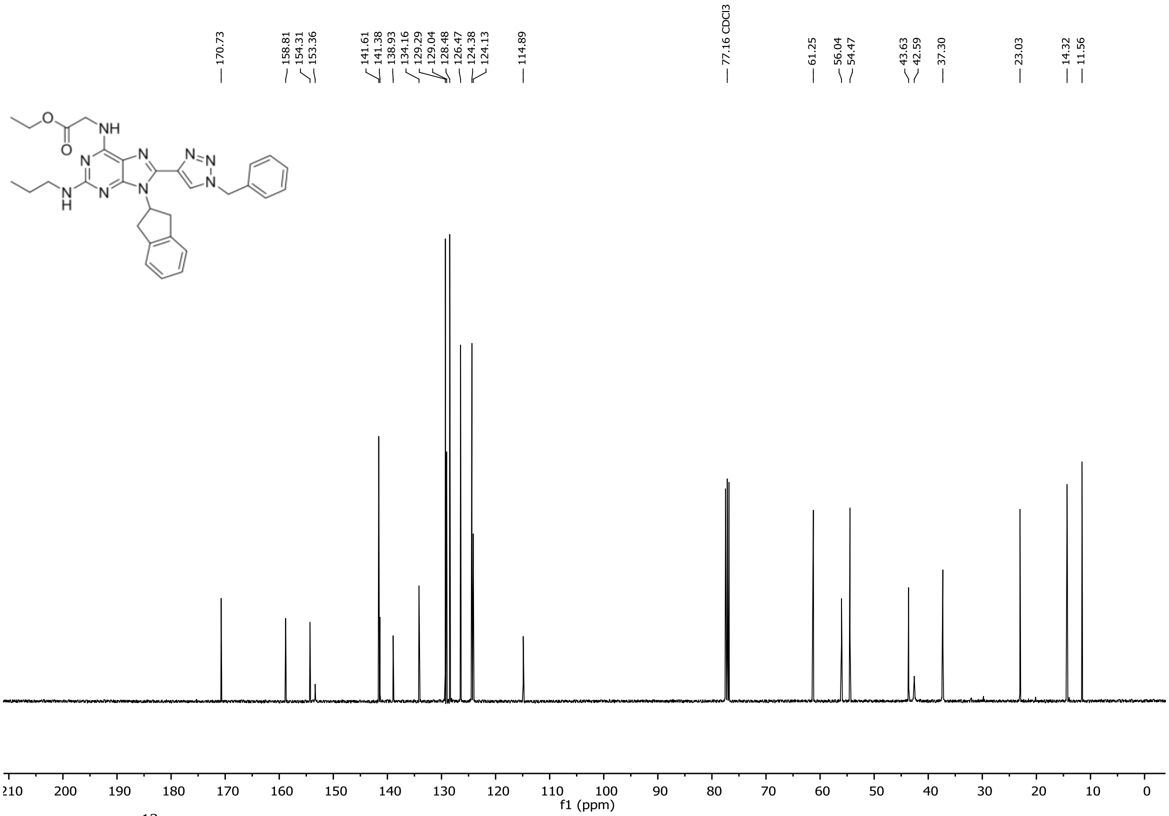

Fig. A103. ^{13}C NMR spectrum of 8-(1-benzyl-1H-1,2,3-triazol-4-yl)-6-(ethoxy-carbonyl-methylamino)-9-(2-indanyl)-2-(1-propylamino)purine (**14e**).

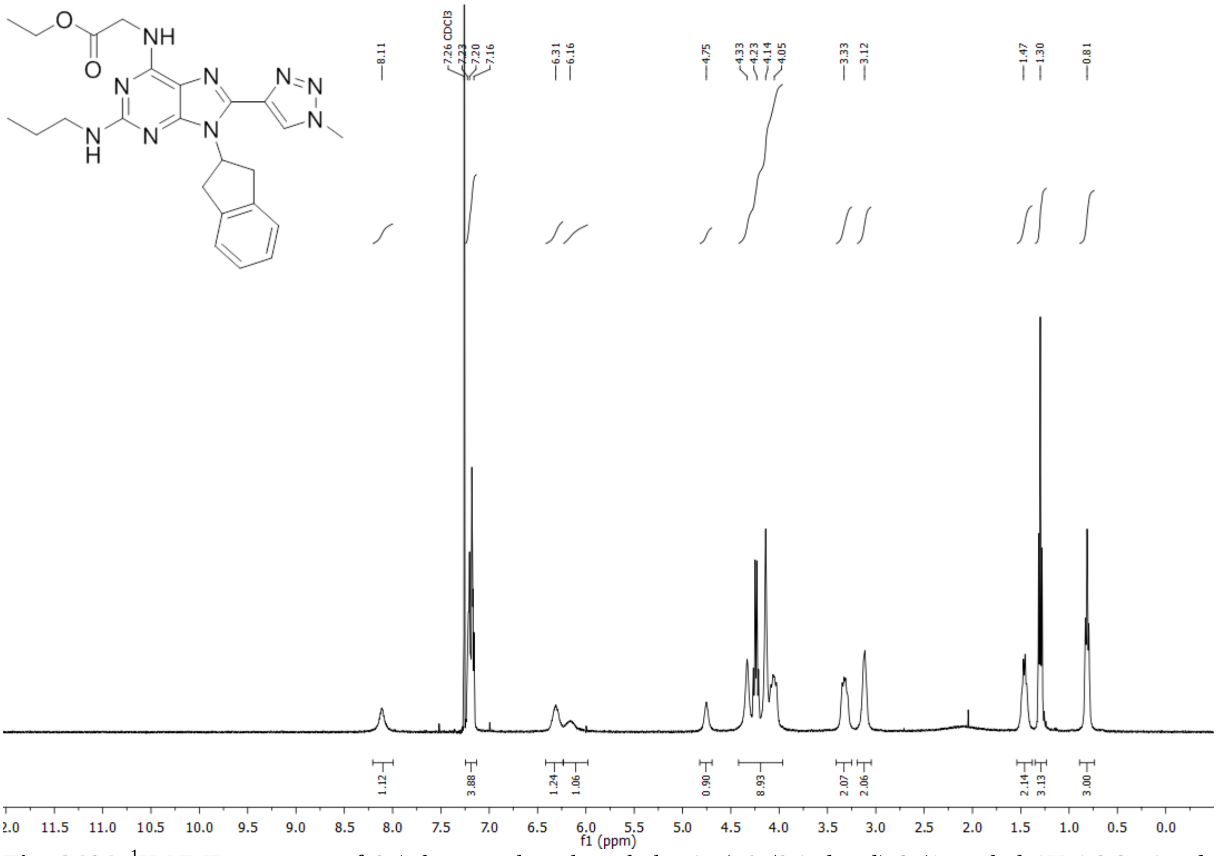

Fig. A104. ^{1}H NMR spectrum of 6-(ethoxy-carbonyl-methylamino)-9-(2-indanyl)-8-(1-methyl-1H-1,2,3-triazol-4-yl)-2-(1-propylamino)purine (**14f**).

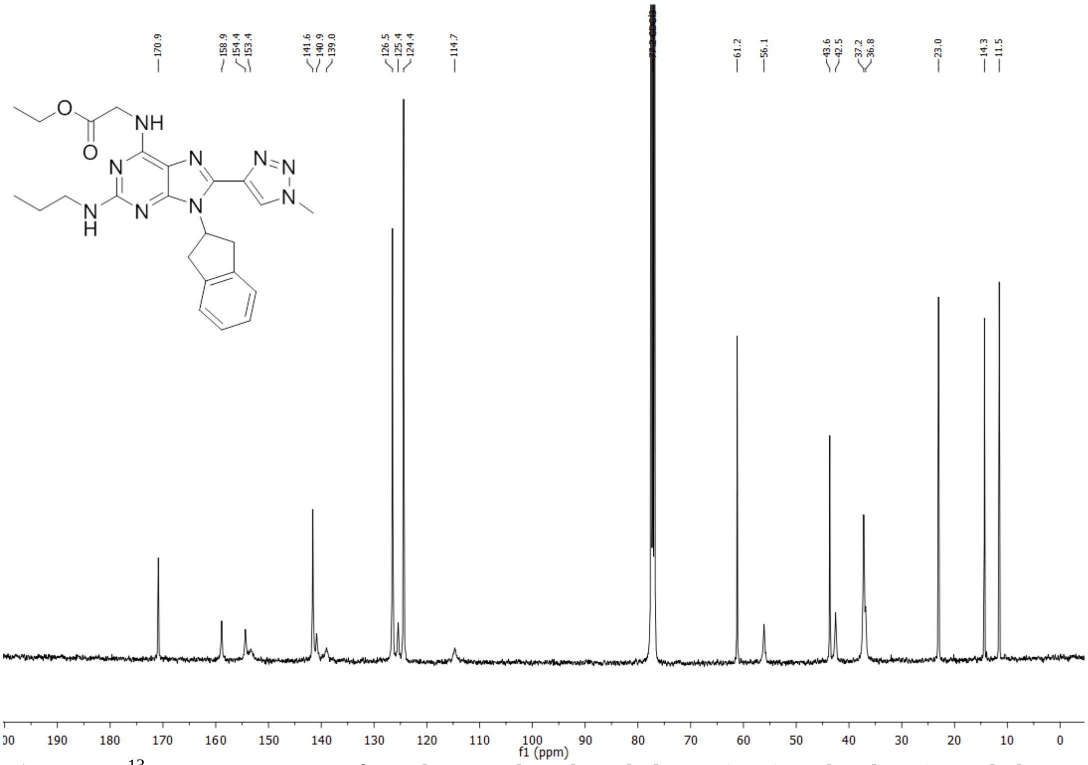

Fig. A105. ^{13}C NMR spectrum of 6-(ethoxy-carbonyl-methylamino)-9-(2-indanyl)-8-(1-methyl-1H-1,2,3-triazol-4-yl)-2-(1-propylamino)purine (**14f**).

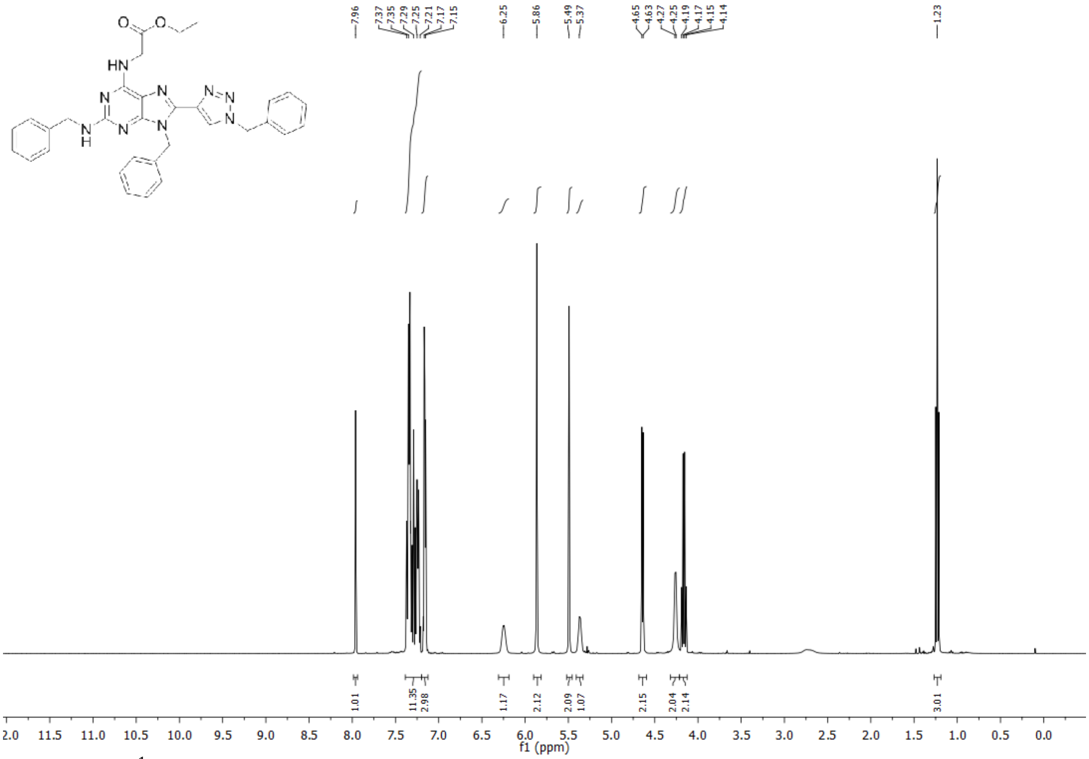

Fig. A106. ^{1}H NMR spectrum of 9-benzyl-2-benzylamino-8-(1-benzyl-1,2,3-triazol-4-yl)-6-(ethoxy-carbonyl-methylamino)purine (**14g**).

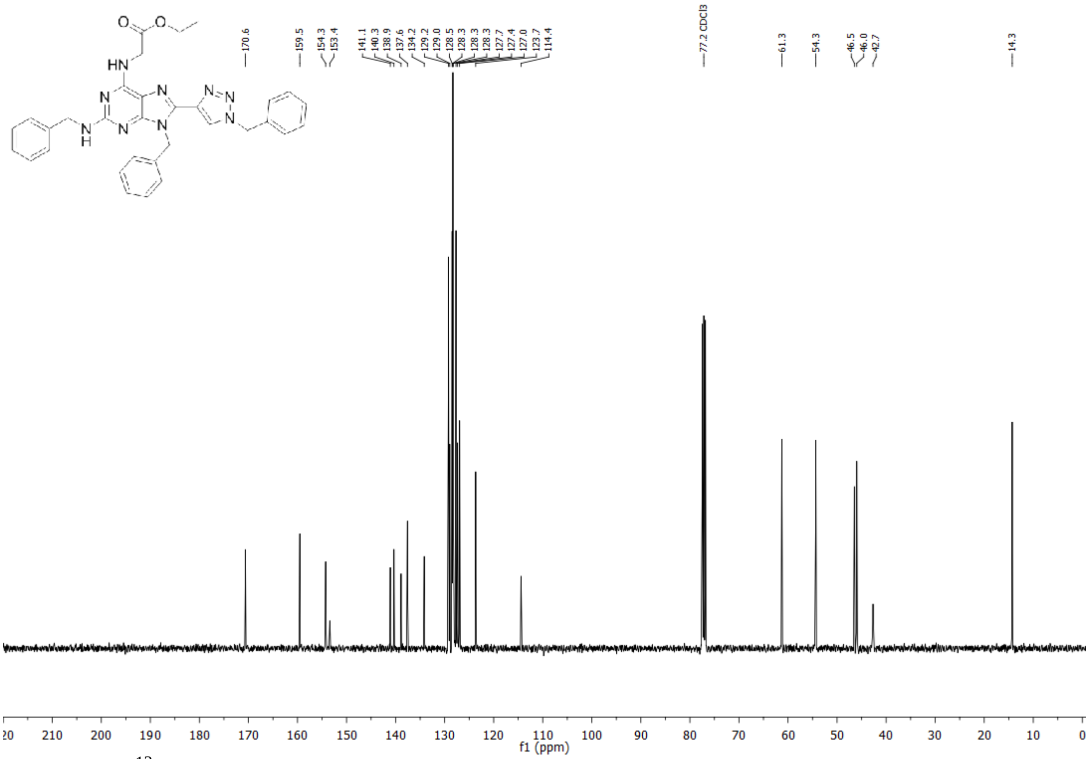

Fig. A107. ^{13}C NMR spectrum of 9-benzyl-2-benzylamino-8-(1-benzyl-1,2,3-triazol-4-yl)-6-(ethoxy-carbonyl-methylamino)purine (**14g**).

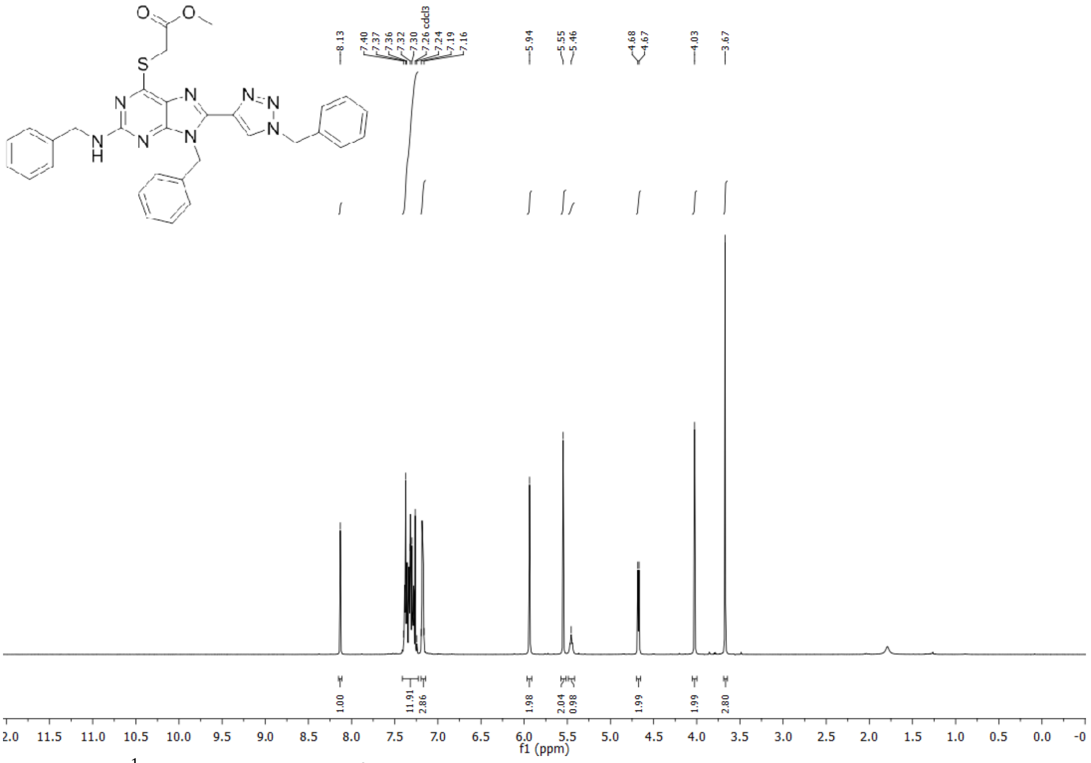

Fig. A108. ^{1}H NMR spectrum of 9-benzyl-2-benzylamino-8-(1-benzyl-1,2,3-triazol-4-yl)-6-(methoxy-carbonyl-methylthio)purine (**14h**).

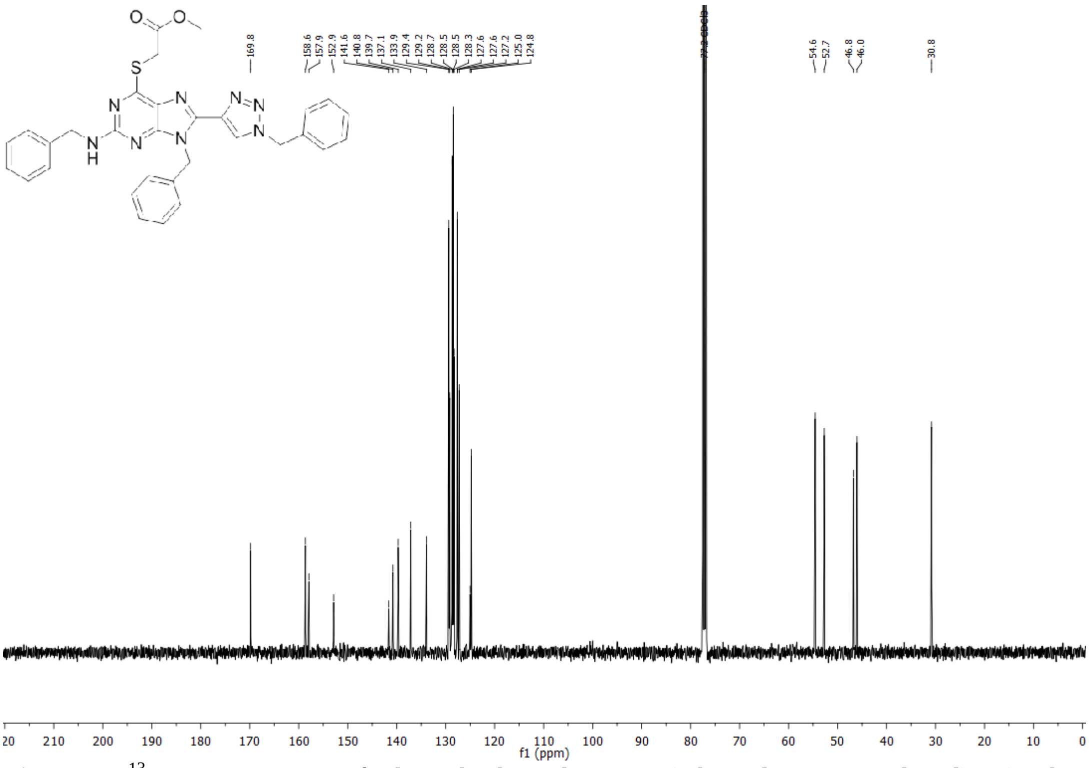

Fig. A109. ^{13}C NMR spectrum of 9-benzyl-2-benzylamino-8-(1-benzyl-1,2,3-triazol-4-yl)-6-(methoxy-carbonyl-methylthio)purine (**14h**).

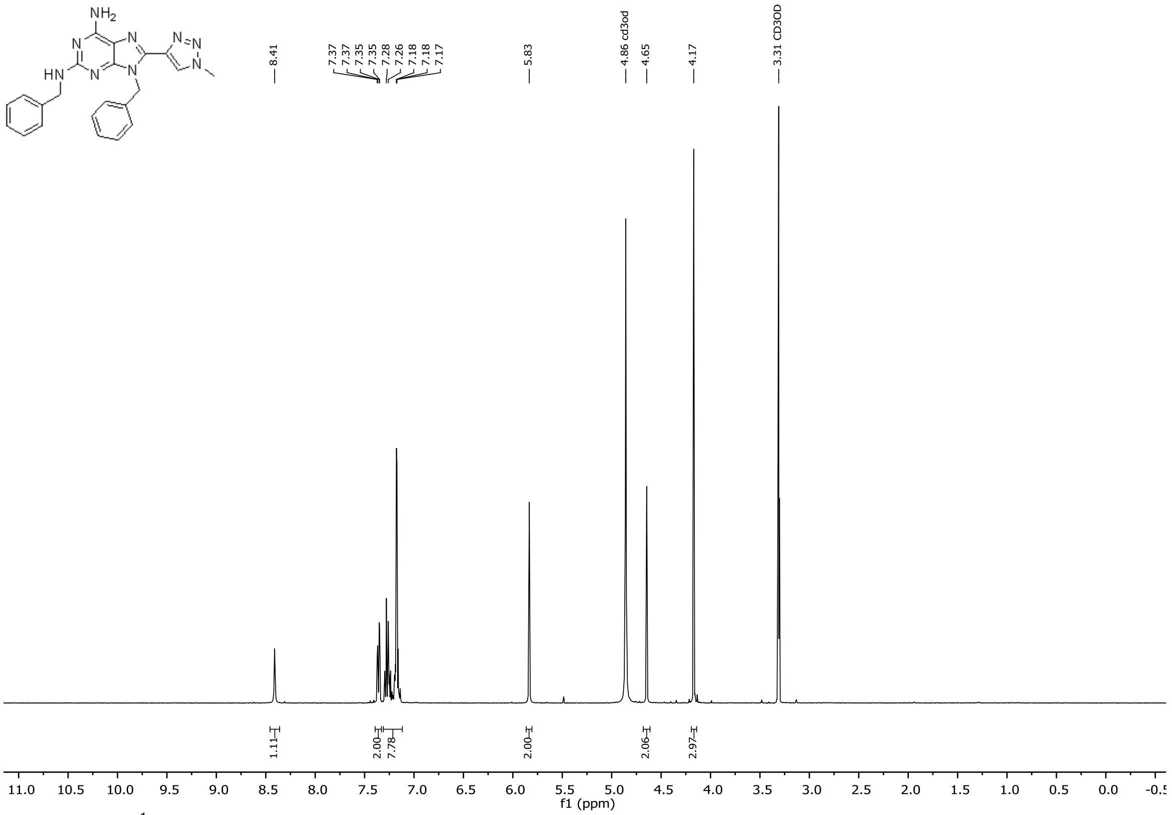

Fig. A110. ^{1}H NMR spectrum of 6-amino-9-benzyl-2-benzylamino-8-(1-methyl-1H-1,2,3-triazol-4-yl)-purine (**14i**).

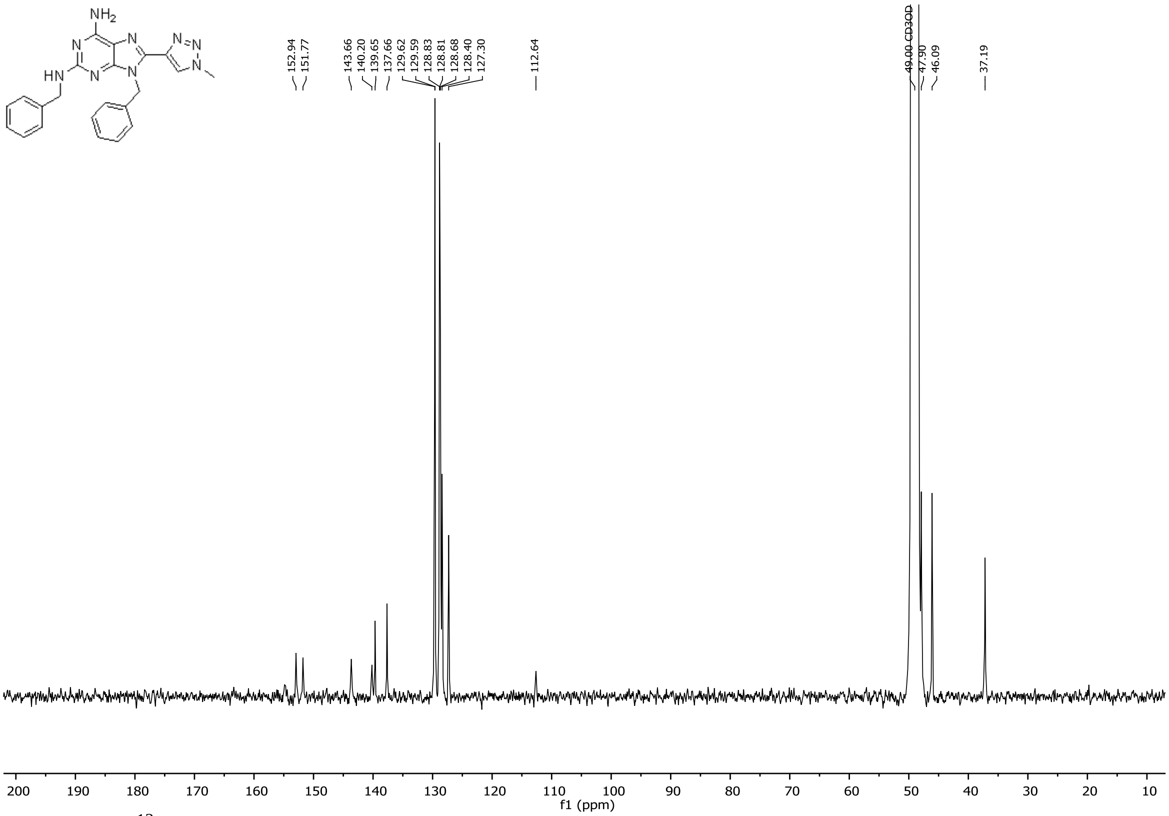

Fig. A111. ^{13}C NMR spectrum of 6-amino-9-benzyl-2-benzylamino-8-(1-methyl-1H-1,2,3-triazol-4-yl)-purine (**14i**).

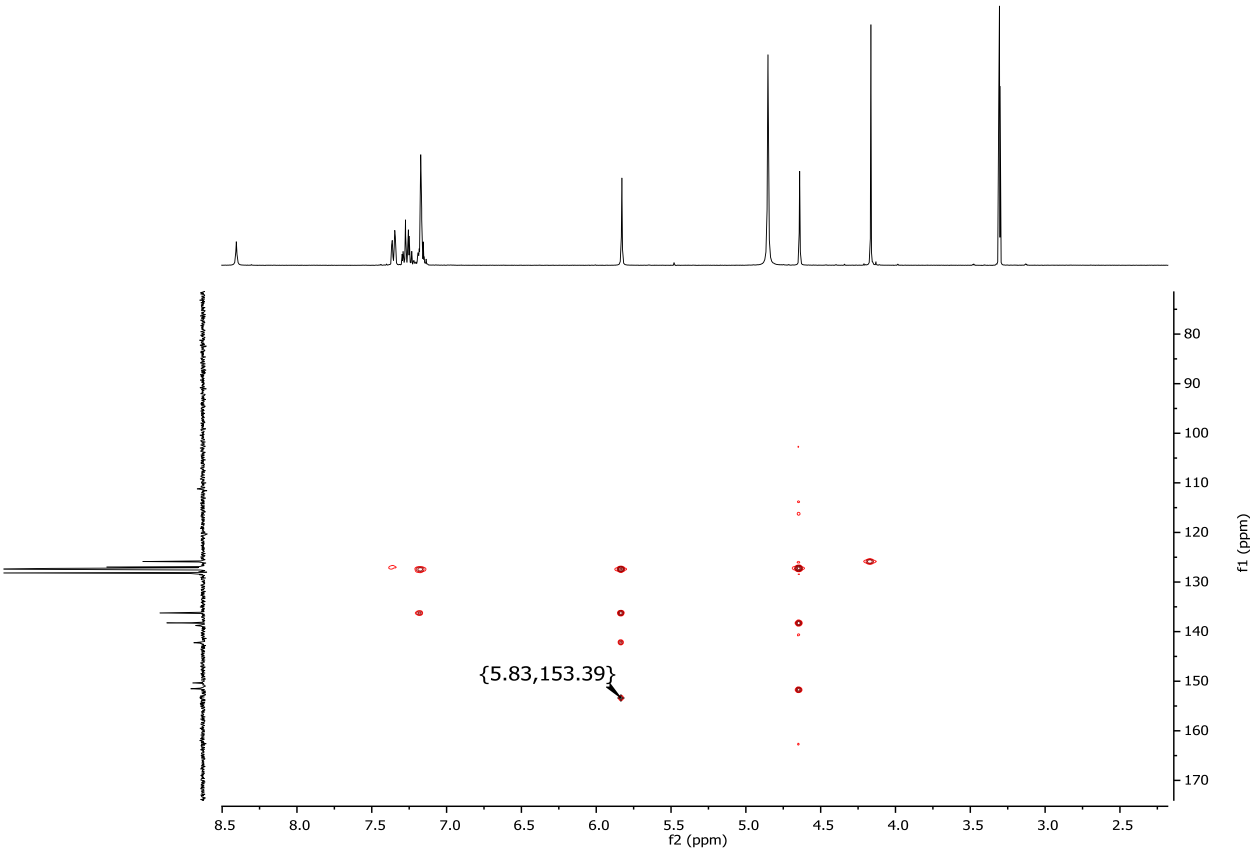

Fig. A112. gHMBC of 6-amino-9-benzyl-2-benzylamino-8-(1-methyl-1H-1,2,3-triazol-4-yl)-purine (**14i**).

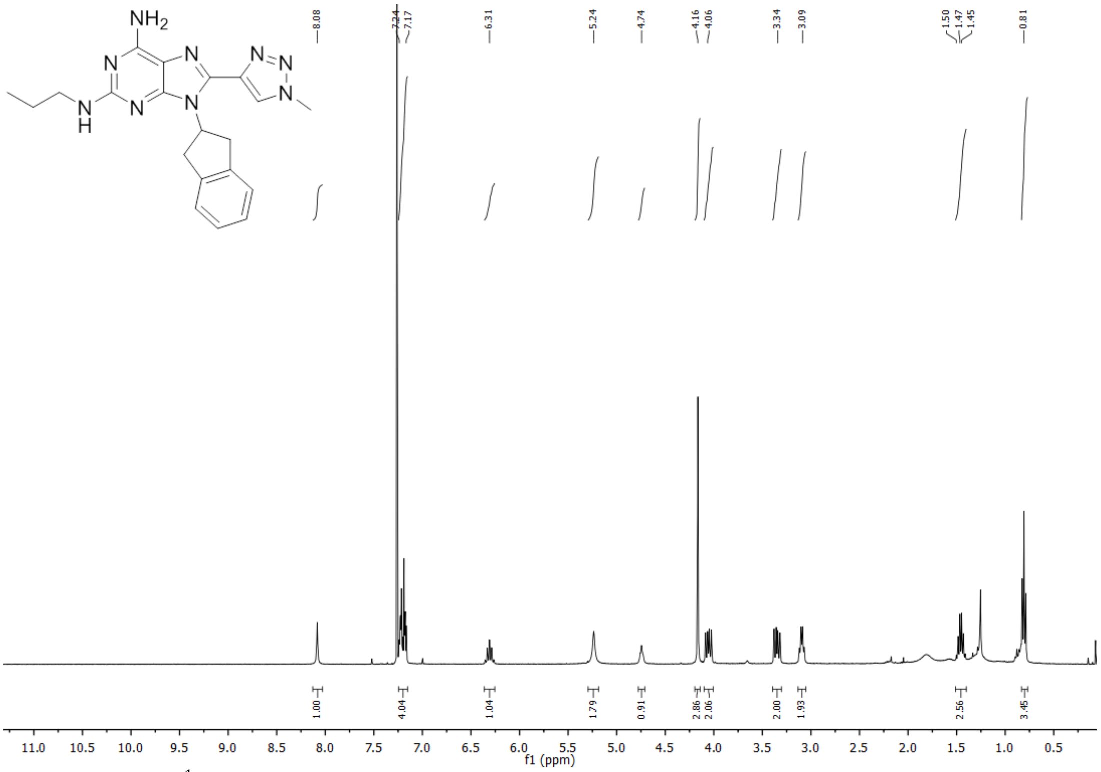

Fig. A113. ^{1}H NMR spectrum of 6-amino-9-(2-indanyl)-8-(1-methyl-1H-1,2,3-triazol-4-yl)-2-(1-propylamino)purine (**14j**).

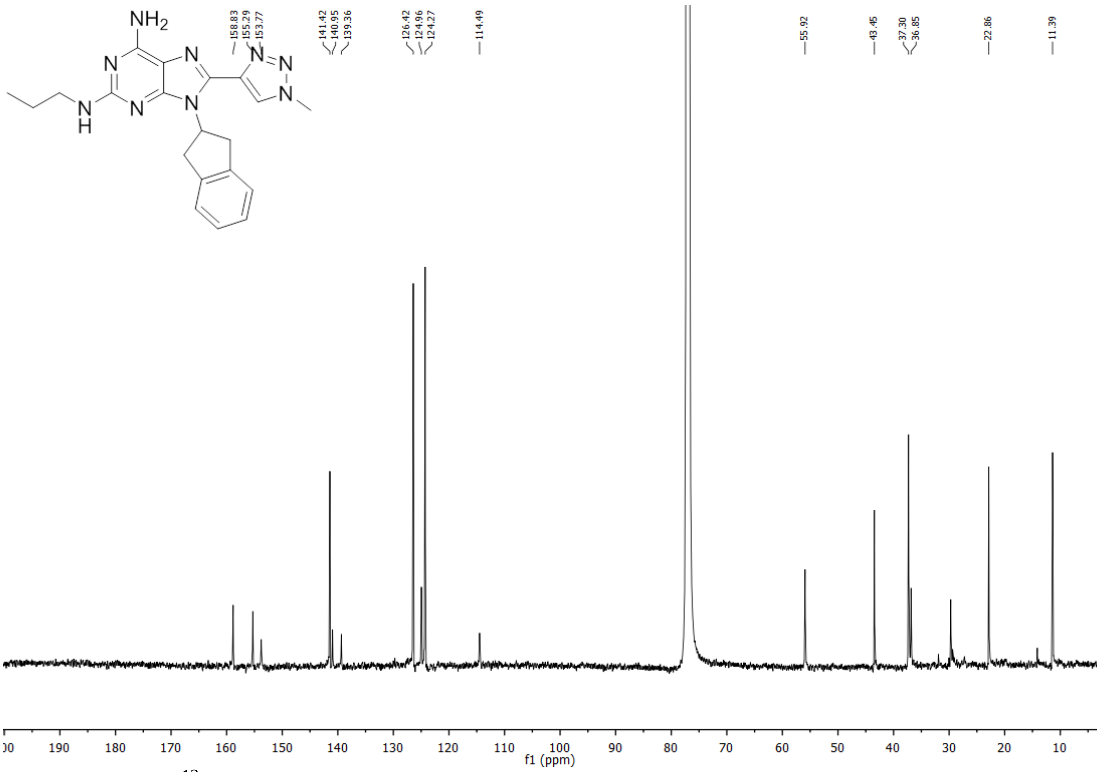

Fig. A114. ^{13}C NMR spectrum of 6-amino-9-(2-indanyl)-8-(1-methyl-1H-1,2,3-triazol-4-yl)-2-(1-propylamino)purine (**14j**).

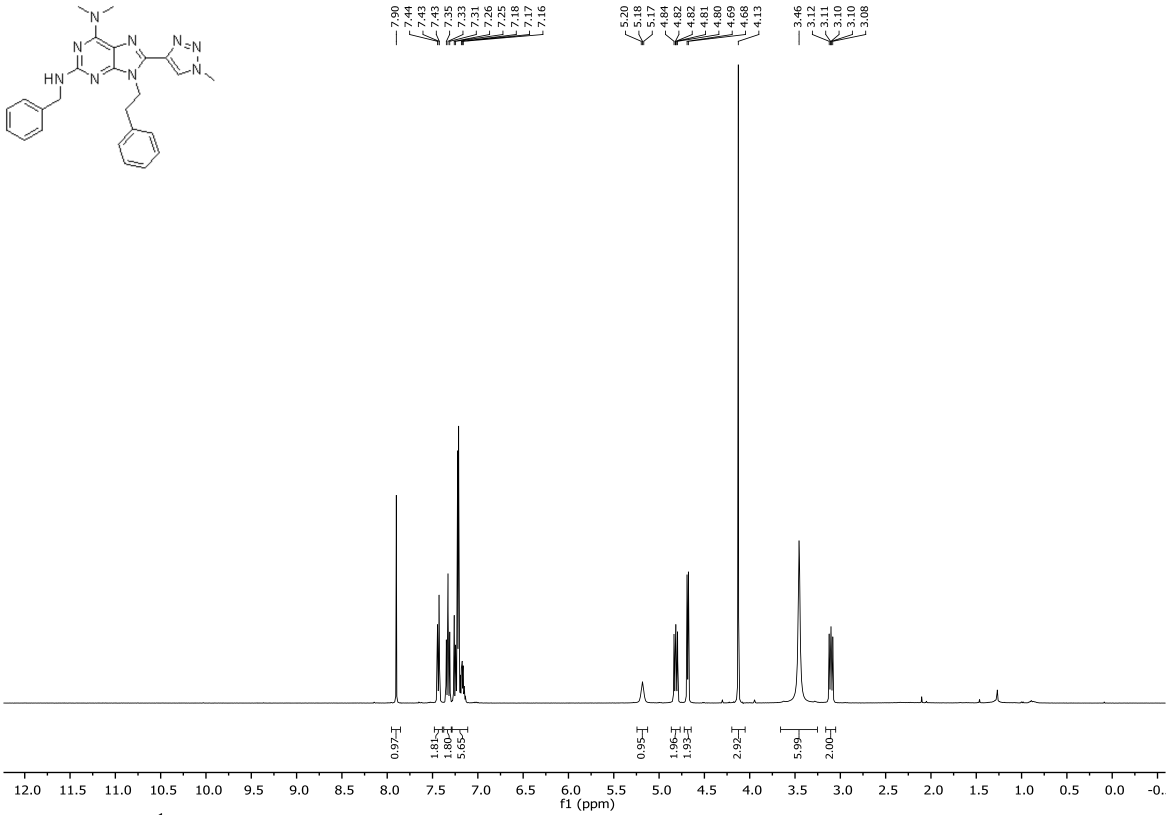

Fig. A115. ^{1}H NMR spectrum of 2-benzylamino-6-dimethylamino-8-(1-methyl-1H-1,2,3-triazol-4-yl)-9-phenethylpurine (**14k**).

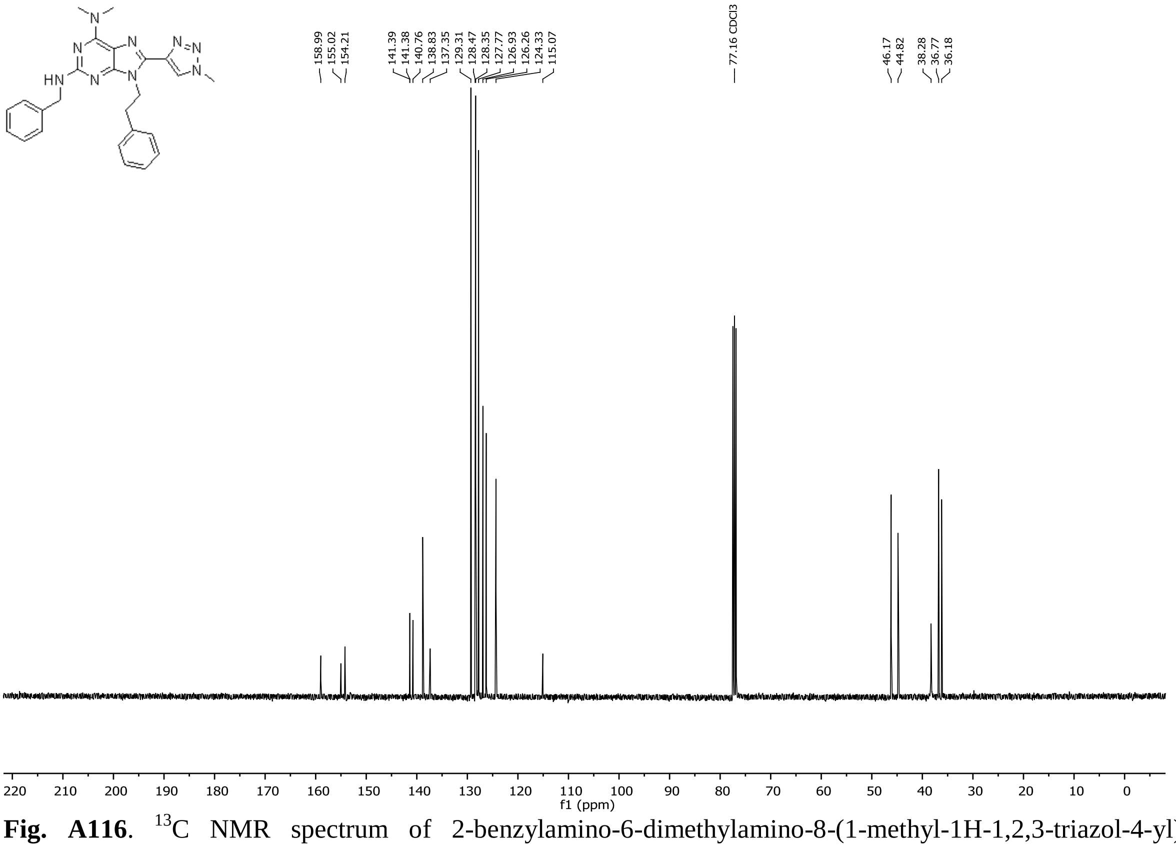

Fig. A116. ^{13}C NMR spectrum of 2-benzylamino-6-dimethylamino-8-(1-methyl-1H-1,2,3-triazol-4-yl)-9-phenethylpurine (**14k**).

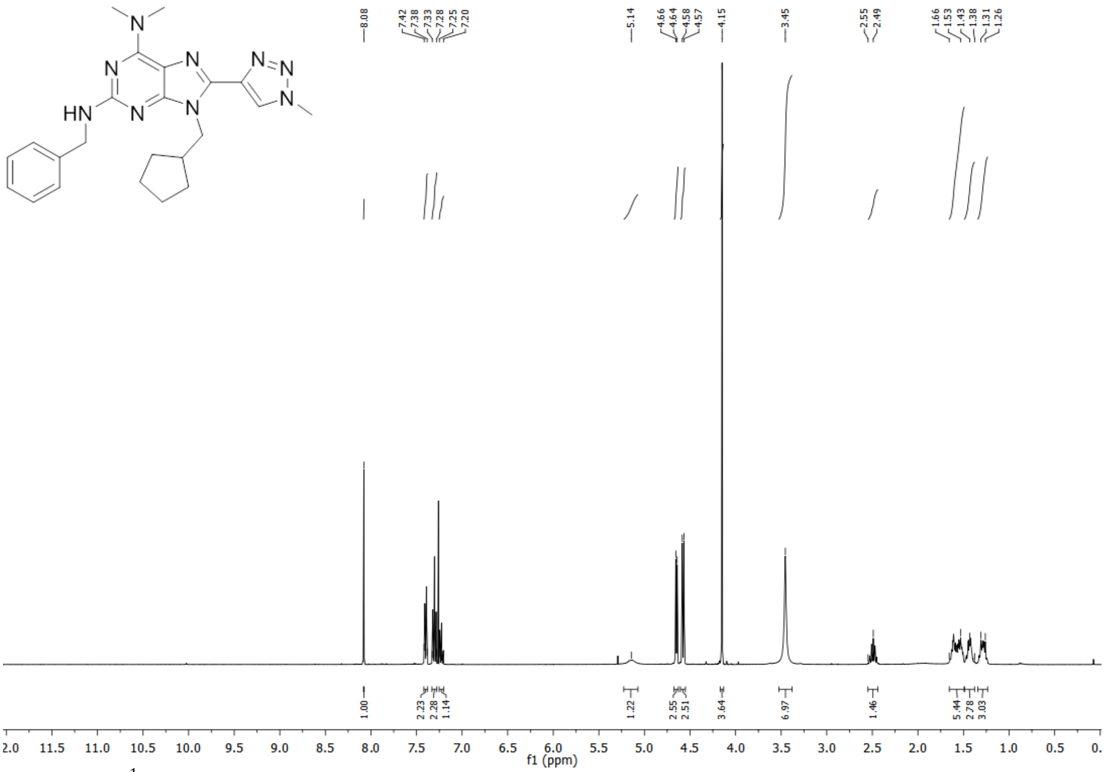

Fig. A117. ^{1}H NMR spectrum of 2-benzylamino-9-cyclophentylmethyl-6-dimethylamino-8-(1-methyl-1H-1,2,3-triazol-4-yl)purine **14l**).

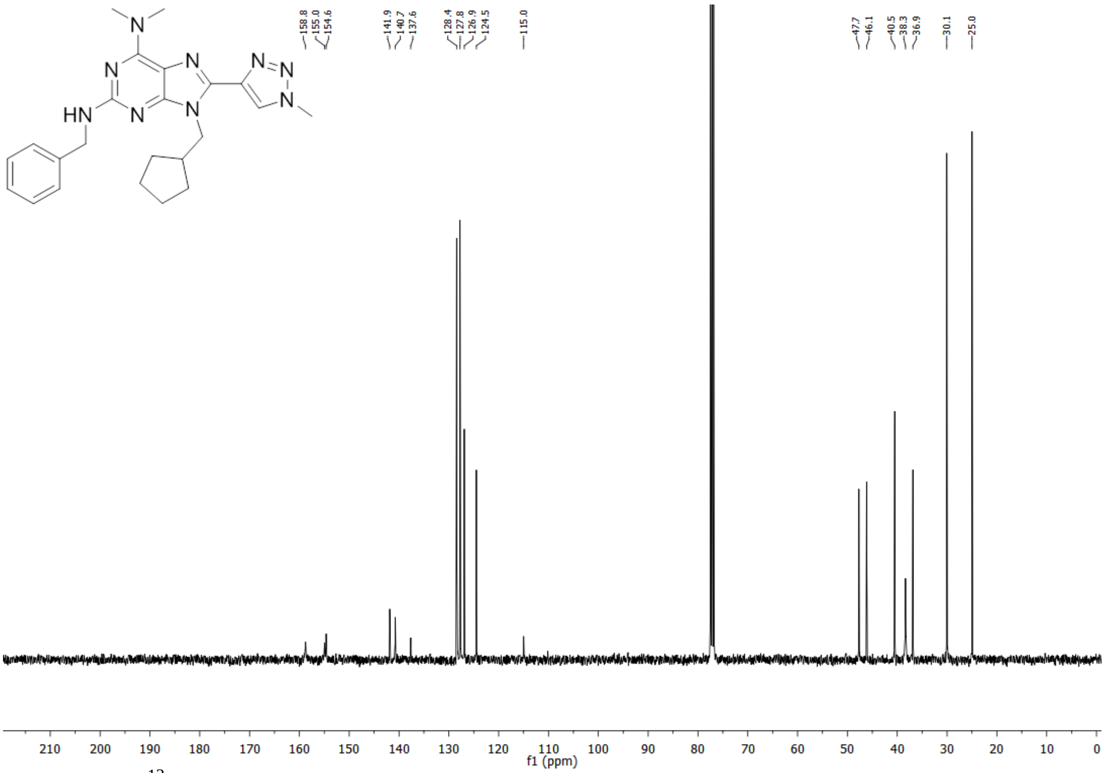

Fig. A118. ^{13}C NMR spectrum of 2-benzylamino-9-cyclophentylmethyl-6-dimethylamino-8-(1-methyl-1H-1,2,3-triazol-4-yl)purine (**14l**).

References

1. Bliman D, Pettersson M, Bood M and Grøtli M. 8-Bromination of 2,6,9-trisubstituted purines with pyridinium tribromide. Tetrahedron Lett. 2014; 55: 2929-2931.
2. Fletcher S, Shahani VM and Gunning PT. Facile and efficient access to 2,6,9-tri-substituted purines through sequential N9, N2 Mitsunobu reactions. Tetrahedron Lett. 2009; 50: 4258-4261.
3. Čechová L, Jansa P, Šála M, Dračínský M, Holý A, et al. The optimized microwave-assisted decomposition of formamides and its synthetic utility in the amination reactions of purines. Tetrahedron 2011; 67: 866-871.
4. Ju Y, Kumar D and Varma RS. Revisiting Nucleophilic Substitution Reactions: Microwave-Assisted Synthesis of Azides, Thiocyanates, and Sulfones in an Aqueous Medium. J. Org. Chem. 2006; 71: 6697-6700
5. MacroModel, version 9.9, Schrödinger, LLC, New York, NY, 2012.
6. Danelius E, Brath U and Erdélyi M. Insight into β-Hairpin Stability: Interstrand Hydrogen Bonding. Synlett 2013; 24: 2407-2410.
7. Erdélyi M, Pfeiffer B, Hauenstein K, Fohrer J, Gertsch J, et al. Conformational Preferences of Natural and C3-Modified Epothilones in Aqueous Solution. J. Med. Chem. 2008; 51: 1469-1473.
8. Dalvit C, Fogliatto G, Stewart A, Veronesi M and Stockman B. WaterLOGSY as a method for primary NMR screening: Practical aspects and range of applicability. J. Biomol. NMR. 2001; 21: 349-359.
9. MacroModel, version 9.9, Schrödinger, LLC, New York, NY, 2012.
10. Schrödinger Suite 2012 Protein Preparation Wizard; Epik version 2.3, Schrödinger, LLC, New York, NY, 2012; Impact version 5.8, Schrödinger, LLC, New York, NY, 2012; Prime version 3.1, Schrödinger, LLC, New York, NY, 2012.
11. Schrödinger, Glide version 5.8, LLC, New York, 2012.
12. LigPrep, version 2.5, Schrödinger, LLC, New York, NY, 2012.
